# Towards Translation of PqsR Inverse Agonists: From In Vitro Efficacy Optimization to In Vivo Proof‐of‐Principle

**DOI:** 10.1002/advs.202204443

**Published:** 2023-01-03

**Authors:** Mostafa M. Hamed, Ahmed S. Abdelsamie, Katharina Rox, Christian Schütz, Andreas M. Kany, Teresa Röhrig, Stefan Schmelz, Wulf Blankenfeldt, Alejandro Arce‐Rodriguez, José Manuel Borrero‐de Acuña, Dieter Jahn, Jessica Rademacher, Felix C. Ringshausen, Nina Cramer, Burkhard Tümmler, Anna K. H. Hirsch, Rolf W. Hartmann, Martin Empting

**Affiliations:** ^1^ Helmholtz‐Institute for Pharmaceutical Research Saarland (HIPS) Helmholtz Centre for Infection Research (HZI) Campus E8.1 66123 Saarbrücken Germany; ^2^ German Centre for Infection Research (DZIF) Partner Site Hannover‐Braunschweig Saarbrücken 66123 Saarbrücken Germany; ^3^ Department of Chemistry of Natural and Microbial Products Institute of Pharmaceutical and Drug Industries Research National Research Centre El‐Buhouth St. Dokki Cairo 12622 Egypt; ^4^ Department of Chemical Biology (CBIO) Helmholtz Centre for Infection Research (HZI) Inhoffenstr. 7 Braunschweig 38124 Saarbrücken Germany; ^5^ Department of Structure and Function of Proteins (SFPR) Helmholtz Centre for Infection Research (HZI) Inhoffenstr. 7 Braunschweig 38124 Saarbrücken Germany; ^6^ Institute for Biochemistry Biotechnology and Bioinformatics Technische Universität Braunschweig Braunschweig Germany; ^7^ Institute of Microbiology Technische Universität Braunschweig 38106 Braunschweig Germany; ^8^ Braunschweig Integrated Centre of Systems Biology (BRICS) Technische Universität Braunschweig 38106 Braunschweig Germany; ^9^ Departamento de Microbiología Facultad de Biología Universidad de Sevilla Av. de la Reina Mercedes no. 6 Sevilla CP 41012 Spain; ^10^ Department for Respiratory Medicine Medizinische Hochschule Hannover Carl‐Neuberg‐Str. 1 30625 Hannover Germany; ^11^ Biomedical Research in Endstage and Obstructive Lung Disease (BREATH) German Center for Lung Research (DZL) 30625 Hannover Germany; ^12^ European Reference Network on Rare and Complex Respiratory Diseases (ERN‐ LUNG) Frankfurt Germany; ^13^ Department for Pediatric Pneumology Allergology and Neonatology Medizinische Hochschule Hannover Carl‐Neuberg‐Str. 1 30625 Hannover Germany; ^14^ Department of Pharmacy Saarland University Campus E8.1 66123 Saarbrücken Germany

**Keywords:** bronchiectasis, in vivo proof‐of‐concept, pathoblocker, *Pseudomonas aeruginosa*, quorum sensing

## Abstract

*Pseudomonas aeruginosa* (PA) is an opportunistic human pathogen, which is involved in a wide range of dangerous infections. It develops alarming resistances toward antibiotic treatment. Therefore, alternative strategies, which suppress pathogenicity or synergize with antibiotic treatments are in great need to combat these infections more effectively. One promising approach is to disarm the bacteria by interfering with their quorum sensing (QS) system, which regulates the release of various virulence factors as well as biofilm formation. Herein, this work reports the rational design, optimization, and in‐depth profiling of a new class of *Pseudomonas* quinolone signaling receptor (PqsR) inverse agonists. The resulting frontrunner compound features a pyrimidine‐based scaffold, high in vitro and in vivo efficacy, favorable pharmacokinetics as well as clean safety pharmacology characteristics, which provide the basis for potential pulmonary as well as systemic routes of administration. An X‐ray crystal structure in complex with PqsR facilitated further structure‐guided lead optimization. The compound demonstrates potent pyocyanin suppression, synergizes with aminoglycoside antibiotic tobramycin against PA biofilms, and is active against a panel of clinical isolates from bronchiectasis patients. Importantly, this in vitro effect translated into in vivo efficacy in a neutropenic thigh infection model in mice providing a proof‐of‐principle for adjunctive treatment scenarios.

## Graphical abstract

This work presents a drug discovery campaign from an optimized hit until a potential candidate compound for adjunctive treatment of chronic *Pseudomonas aeruginosa* lung infections. The described quorum sensing inhibitor (QSI) acts with high anti‐virulence efficacy, favorable DMPK, and no overt safety pharmacology. Notably, this compound synergizes with antibiotic tobramycin in vitro and in vivo providing a compelling in vivo proof‐of‐concept.

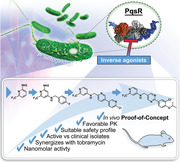

## 1 Introduction

*Pseudomonas aeruginosa* (PA) is a Gram‐negative opportunistic bacterium that is responsible for a wide range of acute and chronic infections in humans.^[^ ^1^, ^2^ ^]^ It is well‐known that the bacteriostatic or bactericidal action of antibiotics intrinsically leads to development of resistance resulting in the emergence of multi‐ or even pan‐resistant PA strains.^[^ ^3^ ^]^ In order to cope with this problem, there is a pressing need for new nontraditional and innovative therapy options beside the continuous search for “traditional” antibiotics.^[^ ^4^ ^]^ Also enhancing or rescuing the efficacy of antibiotics in clinical use is of interest, helping to circumvent or reduce resistance.^[^ ^5^ ^]^ One strategy, which could help to tackle PA infections via new modes‐of‐action while synergizing with antibiotics, is the so‐called pathoblocker approach, which aims to disrupt the virulence of the bacteria without killing them.^[^ ^5^, ^6^, ^7^, ^8^ ^]^ Working either as stand‐alone anti‐infective agents or via adjunctive treatment together with a backbone antibiotic, this strategy would also help to decrease the development of resistance.^[^ ^5^, ^6^, ^7^, ^8^ ^]^ To be more precise, the pharmacokinetic/pharmacodynamic (PK/PD) relationship of the clinical response to standard‐of‐care (SoC) aminoglycosides in patients is typically characterized by the ratio of exposure (expressed, e.g., as *C*_max_) to in vitro efficacy (commonly expressed as the minimal inhibitory concentration, MIC).^[^ ^9^ ^]^ Hence, boosting the antibacterial effect through potentiating agents has the potential to improve an efficient clinical response. In chronic infections, PA forms hard‐to‐eradicate biofilms, tremendously impairing antibiotic efficacy.^[^ ^10^ ^]^ Here, instead of the MIC value, which is measured against planktonic bacteria, the minimal biofilm‐eradicating concentration (MBEC) should rather be considered as a critical efficacy parameter.^[^ ^11^ ^]^ In short, enhancing the antibiotic‐mediated biofilm eradication should directly improve clinical response and, hence, most likely reduce the risk of resistance development.

**Figure 1:**
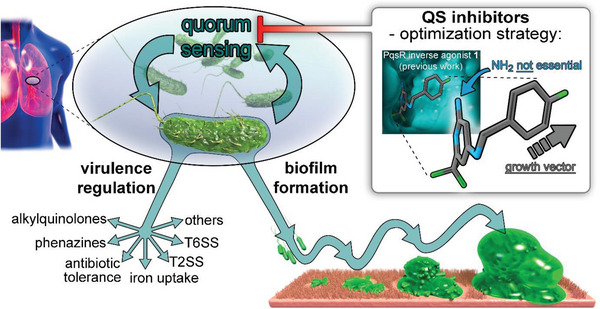
Generation of pathoblockers against PA by inhibition of quorum sensing via inverse agonists of the transcriptional regulator PqsR (MvfR). Multiple virulence mechanisms such as phenazine biosynthesis, AQ biosynthesis, antibiotic tolerance, iron uptake, T2SS, and T6SS as well as biofilm formation are under the control of PqsR.^[^ ^13^, ^14^ ^]^ The medicinal chemistry driven optimization strategy applied in this study is depicted in the inset in the top right corner.

Our strategy to disrupt bacterial virulence is based on the interfere with quorum sensing (QS)—a cell‐to‐cell communication system, which coordinates the release of the various virulence factors and co‐regulates biofilm formation (**Figure** **1**).^[^ ^2^, ^12^, ^13^, ^14^ ^]^ A promising target for QS inhibitors (QSI) in PA is *Pseudomonas* quinolone signaling receptor (PqsR), also called multiple virulence factor regulator (MvfR).^[^ ^15^ ^]^ PqsR is a transcriptional regulator that controls the Pseudomonas quinolone signaling (PQS) system and is considered as one of the master regulators of PA virulence.^[^ ^16^ ^]^ We and others have demonstrated the utility of PqsR as an attractive antivirulence target.^[^ ^15^, ^17^, ^18^, ^19^, ^20^, ^21^, ^22^, ^23^ ^]^

Previously, we have reported the first PqsR antagonists developed from the natural ligand HHQ,^[^ ^18^, ^19^ ^]^ but also the discovery of a structurally divergent hit scaffold as well as first successful lead‐generation efforts.^[^ ^20^, ^21^, ^23^ ^]^ Herein, we describe the rational hit‐to‐lead‐to‐candidate optimization of a new chemical class of potent PqsR antagonists, starting from the previously published optimized hit **1**.^[^ ^23^ ^]^ The frontrunner compound reported in this study potently impairs pyocyanin production in typical lab strains as well as clinical isolates from noncystic fibrosis bronchiectasis patients and enhances the eradication efficacy of SoC antibiotic tobramycin against PA biofilms. The binding mode to PqsR was elucidated by an X‐ray cocrystal structure. Furthermore, we include extensive data on biological profiling regarding in vitro and in vivo drug metabolism and pharmacokinetics (DMPK) characterization studies. Notably, we conducted an in vivo proof‐of‐concept study demonstrating the efficacy of an adjunctive treatment regimen in a neutropenic thigh infection model in mice.

## 2 Results and Discussion

### 2.1 Medicinal Chemistry‐Driven Optimization

Recently, we have reported compound **1** (Figure 1 and **Table** **1**) as a hit compound obtained during our fragment‐based drug discovery efforts.^[^ ^23^ ^]^ This compound showed a half maximal inhibitory concentration (IC_50_) of 2.3 × 10^−6^ m toward PqsR in a heterologous *lacZ* reporter gene assay in *E. coli* and served as our starting point in the optimization process.^[^ ^23^ ^]^ Activation of PqsR by its native agonist PQS leads to the direct and indirect transcriptional modulation of genes responsible for environmental adaptation, virulence factor expression, iron acquisition, redox signaling, antibiotic tolerance, cytotoxicity, and immune modulation/evasion.^[^ ^16^, ^24^ ^]^ One prominent phenotypic effect of disrupting PqsR‐dependent QS is the blockade of pyocyanin production as part of the phenazine biosynthesis (Figure 1).^[^ ^16^ ^]^ Therefore, all compounds synthesized were tested for the inhibition of this important virulence factor as well as activity in the reporter gene assay. Generally, a reasonable correlation was observed between inverse agonistic activity against PqsR (*E. coli* reporter‐gene assay) and pyocyanin inhibition in PA (Tables 1, 2, 3). Compound optimization was further guided by early in vitro ADME properties (Absorption, Distribution, Metabolism, Excretion), focusing particularly on kinetic solubility and metabolic stability (against mouse liver microsomes; MLM).

**Table 1:** *Pseudomonas* quinolone signaling receptor (PqsR) inverse agonistic activity and pyocyanin inhibitory activity as well as kinetic solubility and metabolic stability of compounds **1**–**6**

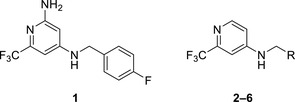					
	R	IC_50_ PqsR [×10^−9^ m]	IC_50_ Pyocyanin [×10^−6^ m]	Solubility [×10^−6^ m]	MLM *t*_1/2_ [min]^a)^
1	–	2309	107		
2	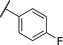	2207 ± 4038	176 ± 47.4	>200	54
3	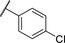	1376 ± 735	19.0 ± 1.2	>200	44
4	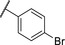	861 ± 162	14.4 ± 1.6	>200	61
5	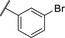	4955 ± 4850	71.4 ± 27.2	>200	20
6	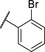	>10000	126 ± 10.6	>200	4

Importance of the NH2 group and identification of growth vector.

^a)^ Half‐life against mouse liver microsomes (MLM).

As a first step, we evaluated the importance of the amino substituent on the head group. Compound **2** lacking the amino group showed similar activity to that of **1**, indicating that the amino group does not contribute significantly to binding. The position of the CF_3_ and the type of substituent was previously evaluated during our initial fragment growing efforts.^[^ ^23^ ^]^ For further optimization, we kept the pyridine head lacking the amino group due to better chemical accessibility, enabling faster design‐make‐test cycles. For initial structure activity relationship (SAR) considerations we compared the potency based on the *E. coli* reporter‐gene assay. Replacing the 4‐fluoro substituent in the Eastern ring with Cl (**3**) and then Br (**4**) resulted in further improvement in activity. Varying the position of the Br to *meta* (**5**) or *ortho* (**6**) positions was detrimental for activity suggesting that the *para* position was the most suitable direction for further elongation.

Replacing the *p*‐Br in **4** with a *p*‐chlorophenyl (**7**) showed a significant boost in activity, clearly indicating the utility of installing a linear bi‐aromatic motif in this position (**Table** **2**). As a next step, we aimed to optimize this biphenyl ring system by introducing different heteroaryls. Firstly, we substituted the middle phenyl ring with different pyridines **8** and **9**, which resulted in variable potencies with the pyridine substitution pattern in **9** being the most potent. Accordingly, this pyridine core was kept for modification of the terminal phenyl ring (**10** and **11**). Compound **11** was the most potent and the positions of the nitrogen atoms in this compound seemed to be optimal among the other derivatives **8**–**11**. Although, the biphenyl compound **7** was more active than **11**, we continued our optimization with **11** due to better solubility and metabolic stability. We then tested the effect of the linker atom by replacing the nitrogen atom with oxygen (**12**) and sulfur (**13**). The oxygen linker (**12**) led to a significant loss in activity, while the sulfur (**13**) showed slightly lower activity as well as impaired solubility and metabolic stability compared to **11**.

**Table 2:** *Pseudomonas* quinolone signaling receptor (PqsR) inverse agonistic activity and pyocyanin inhibitory activity as well as kinetic solubility and metabolic stability of compounds **7**–**13**

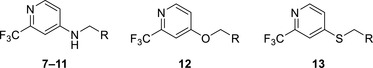					
	R	IC_50_ PqsR [×10^−9^ m]	IC_50_ Pyocyanin [×10^−6^ m]	Solubility [×10^−6^ m]	MLM *t*_1/2_ [min]^a)^
7	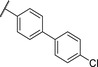	101 ± 37.3	0.449± 0.074	10	>40
8	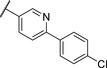	210 ± 125	3.3 ± 1.3	38.9	50
9	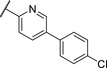	179 ± 103	2.9 ± 3.9	26.6	57
10	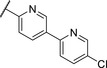	435 ± 198	35.1 ± 27.5	>200	75
11	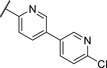	131 ± 9.3	7.2 ± 4.6	160.7	>480
12	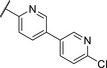	818 ± 436	>20	48.1	74
13	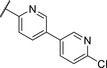	158 ± 36.1	3.1 ± 0.9	42.3	6

Bi‐aryl core optimization.

^a)^ Half‐life against mouse liver microsomes (MLM).

We used compound **11**, with the most favorable nitrogen linker, as a scaffold for further optimization of the Eastern part of the molecule (**Table** **3**). Replacing the Cl with a OMe (**14**) resulted in slight loss in activity. Adding an extra *meta* substituent like a Me (**15**) or CF_3_ (**16**) did not show any improvement, while adding a second Cl (**17**) resulted in a significant boost in activity. Combining the *p*‐OMe and the *m*‐Cl (**18**) showed comparable activity to monosubstituted *p*‐Cl (**11**) being around half as active than the dichloro motif (**17**). An effort to further elongate the molecule with a phenoxy moiety (**19**) did not show further improvement in activity compared to the chloro‐bearing compound **11** while being more potent than the methoxy derivative **14**.

**Table 3:** *Pseudomonas* quinolone signaling receptor (PqsR) inverse agonistic activity and pyocyanin inhibitory activity as well as kinetic solubility and metabolic stability of compounds **14**–**25**

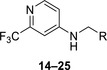					
	R	IC_50_ PqsR [×10^−9^ m]	IC_50_ Pyocyanin [×10^−6^ m]	Solubility [×10^−6^ m]	MLM *t*_1/2_ [min]^a)^
14	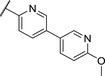	235 ± 13	2.9 ± 0.8	>200	77
15	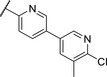	154 ± 79.8	1.9 ± 0.3	112.5	<5
16	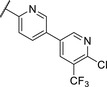	176 ± 9.8	2.1 ± 0.4	100.9	384
17	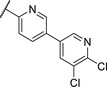	53.9 ± 11.8	0.802 ± 0.2	76.2	45
18	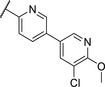	96.8 ± 12.4	1.5 ± 0.2	42.4	2
19	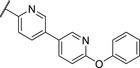	159 ± 13.6	4.2 ± 1.1	25.6	35
20	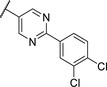	39.4 ± 2.6	0.234 ± 0.057	16.6	61
21	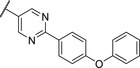	60.5 ± 11.4	0.32 ± 0.11	10.9	32
22	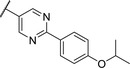	14.2 ± 4.0	0.521 ± 0.3	41.7	>85
23	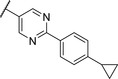	61.4 ± 34.9	0.591 ± 0.07	16.5	3
24	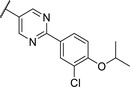	27.5 ± 4.7	0.245 ± 0.04	47.2	19
25	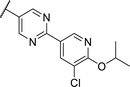	43.7 ± 12.7	0.523 ± 0.05	37.9	>480

Identification of the pyrimidine core.

^a)^ Half‐life against mouse liver microsomes (MLM).

**Figure 2:**
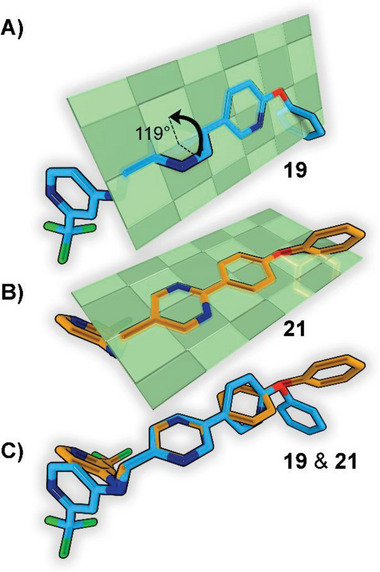
A) Energy‐minimized conformation of compound **19** showing the nonplanar biphenyl ring. b) Energy‐minimized conformation of compound **21** show the planar biphenyl ring. C) Overlay of compounds **19** and **21**.

By looking at the energy‐minimized conformation of **19** (**Figure** **2**), we concluded that the favored conformation of this bi‐aryl pyridine system was most likely in a tilted and nonplanar one. Clearly, this would not only impact the orientation of the terminal *meta* substituents but also influence the steric requirements and pi‐stacking characteristics of the scaffold. To test whether a planar system was beneficial for activity, we designed and synthesized pyrimidine analogues **20** and **21** of the two potent pyridine derivatives **17** and **19**, respectively) so far. By this means, we aimed to remove the steric *ortho*‐effect of the corresponding phenyl hydrogen atoms and, thereby, favor the planar conformation. Indeed, pyrimidines **20** and **21** turned out to be more potent than their dipyridine counterparts **17** and **19**. Therefore, this motif was kept constant for further optimization efforts.

Replacing the terminal phenoxy group by an isopropoxy substituent (**22**) was beneficial for potency in the reporter‐gene assay in contrast to installing a cyclopropyl (**23**) in this position hinting at the importance of the oxygen linker for target interaction. Adding an extra Cl besides the *para*‐isopropoxy **24** improved the antivirulence efficacy in PA despite being slightly less active in the reporter‐gene assay (cf. **22**). Finally, introducing an additional nitrogen in the disubstituted phenyl ring of **24** leading to pyridine‐bearing **25** was detrimental for activity.

In conclusion, we were able to identify and optimize a novel bi‐aryl scaffold resulting in nanomolar activity. The most potent compounds were pyrimidines **20** and **24**. However, compound **24** was chosen as our frontrunner as it showed the best compromise in the biological activities together with suitable solubility.

### 2.2 Structure of the Target–Inhibitor Complex

**Figure 3:**
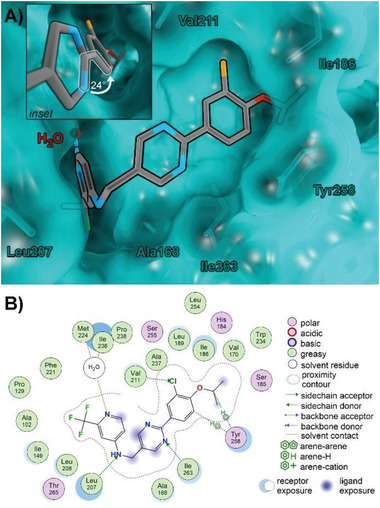
X‐ray cocrystal structure of **24** in complex with PqsR. A) 3D rendering of the ligand–receptor complex. Protein surface and carbons are shown in cyan, carbons of compound **24** are shown in gray, nitrogen is blue, oxygen is red, fluorine is green, and chlorine is orange. Hydrogens left out for clarity. The inset shows a close‐up of the bi‐aryl ring system, highlighting the decreased angle of the indicated dihedral (white markings). B) 2D interaction scheme of compound **24**.

We solved the crystal structure of **24** in complex with PqsR at a resolution of 2.74 Å (**Figure** **3**). Overall, the observed binding mode was in accordance with previously reported QSI bearing the trifluoromethylpyridine headgroup.^[^ ^20^, ^21^, ^23^ ^]^ Notably, the detected electron density confirmed a more planar conformation of the bi‐aryl ring system in **24**. Upon binding to PqsR, both rings tilted about 24° as observed by the corresponding dihedral angle (see the inset in Figure 3A). In comparison to the values shown in Figure 2, this would translate to an angle of 156° between both ring systems, which is closer to 180° (full planarity) than 119° (see Figure 3A). Investigating the complex more closely revealed that the *meta*‐chloro substituent of the Eastern ring fills a small pocket near Ile186, while the isopropoxy group protrudes into a tunnel‐like cavity framed by Ile186 and Tyr258. We conclude that the planar conformation promotes this space‐filling mode‐of‐interaction, rendering the pyrimidine scaffold beneficial for achieving high affinity.

### 2.3 In‐Depth In Vitro Activity Assessment

#### 2.3.1 Abolishing Alkylquiniolone Biosynthesis

**Figure 4:**
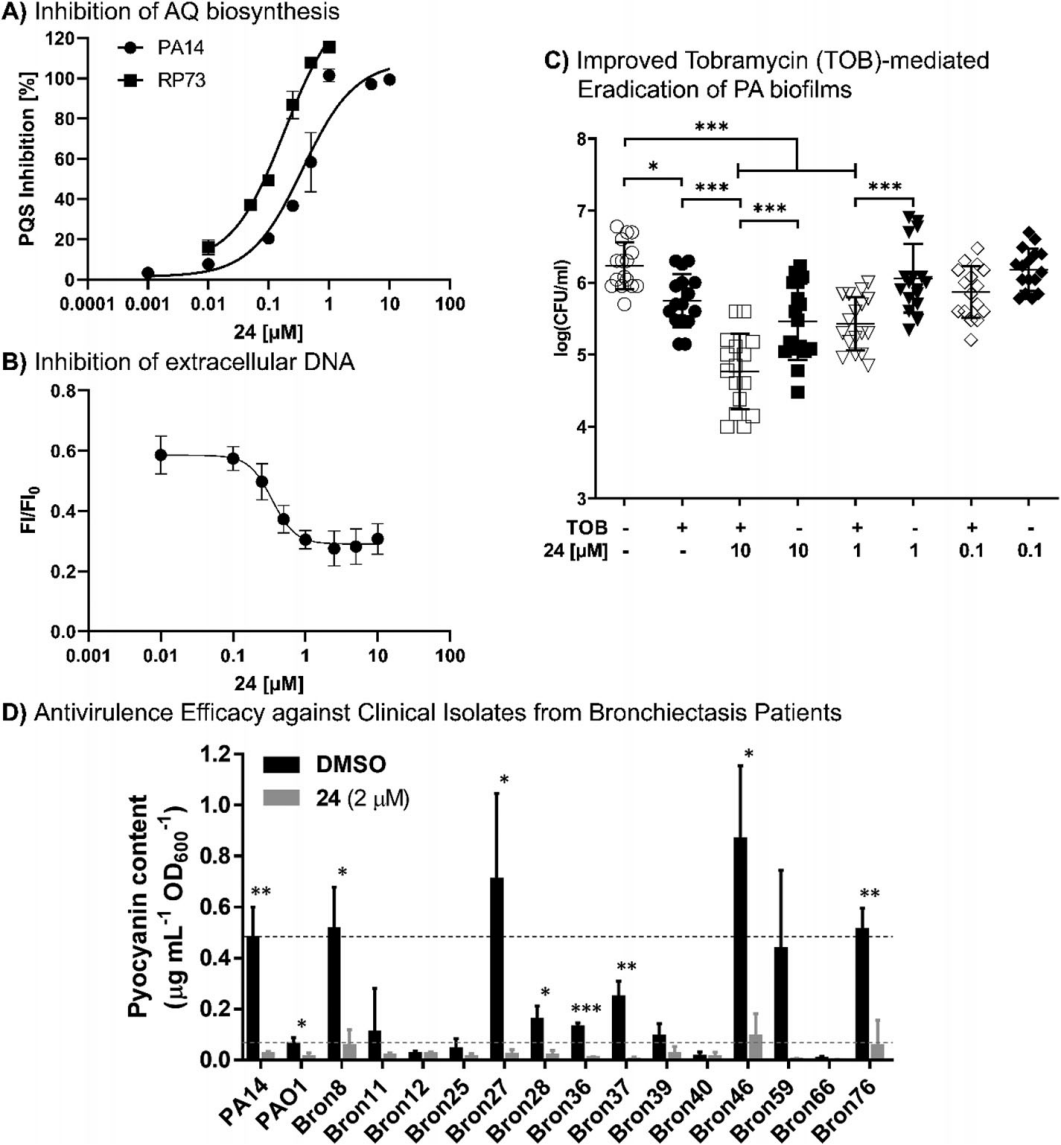
Detailed efficacy assessment of compound **24** in cell‐based assay systems. A) Dose‐dependent inhibition of the QS autoinducer molecule PQS in PA strains PA14 and RP73. B) Dose‐dependent reduction of PA14 eDNA release as determined via propidium iodine staining. IC_50_ 0.346 × 10^−6^ m (95%CI: 0.173–0.630). Mean ± SEM of at least three independent measurements are depicted. C) Combination of **24** and tobramycin (TOB) in the Calgary biofilm device. Biofilms were grown for 24 h in presence of **24**/DMSO, followed by treatment of indicated samples with TOB for another 24 h. Viable cells were determined after 48 h via colony‐forming units (CFU) count. Means and SD of three independent measurements are depicted ^(*^ *p* < 0.05, ^***^ *p* < 0.001 according to Tukey's range test). D) Inhibition of pyocyanin production in 14 PA clinical isolates from bronchiectasis patients and the laboratory strains PA14 and PAO1 (dashed lines) by compound **24**. Each strain was grown in LB‐medium for 16 h in presence of either 2 × 10^−6^ m **24**/DMSO or DMSO as control. Subsequently, pyocyanin was chloroform‐extracted from the supernatants and spectrophotometrically quantified by its absorbance at 520 nm. Means and SD of at least three independent measurements are depicted (^*^ *p* < 0.05, ^**^ *p* < 0.01, ^***^ *p* < 0.001 according to unpaired Student's *t* tests).

Biosynthesis of the alkylquinolone (AQ) autoinducers PQS and HHQ, as well as related derivatives HQNO and 2‐AA is mediated by PqsR‐dependent *pqs*A–E operon expression. These individual autoinducers are known to mediate further virulence traits such as persister‐cell formation, autolysis, eDNA release, etc.^[^ ^25^ ^]^ Inhibition of PqsR is expected to have a downregulating effect on the *pqsA–E* operon leading to decreased concentrations of AQs. In order to test whether compound **24** has an inhibitory effect on this operon, two PA strains, PA14 (typical lab strain) and RP73 (cystic‐fibrosis clinical isolate),^[^ ^26^ ^]^ were used to evaluate concentration‐dependent autoinducer suppression by compound **24**. These strains produce different AQ base levels in vitro (absolute concentrations PA14 < RP73). Incubation with compound **24** showed a strong dose‐dependent inhibition with IC_50_ values between 160 and 453 × 10^−9^ m for individual AQs (**Table** **4**, **Figure** **4**A) with no considerable differences between both strains.

**Table 4:** IC_50_ values of compound **24** on the biosynthesis of individual alkylquinolones (AQs) *Pseudomonas aeruginosa* strains PA14 and RP73

Alkylquinolone	IC_50_ PA14 [×10^−9^ m]	IC_50_ RP73 [×10^−9^ m]
PQS	349	160
HHQ	233	453
HQNO	347	230
2‐AA	386	213

#### 2.3.2 Impact on PA Biofilms

As a next step, we investigated the performance of our frontrunner **24** on PA biofilms. We previously reported on the impact of QSIs on the important biofilm‐matrix component eDNA (extracellular DNA).^[^ ^20^ ^]^ In line with these findings, **24** showed dose‐dependent inhibition of eDNA release in PA14, with a submicromolar IC_50_ of 0.346 × 10^−6^ m (Figure 4B).

Intrigued by the potency of **24** to reduce signal molecule release as well as eDNA in PA14, we investigated whether these effects would result in potentiation of antibiotic‐mediated biofilm eradication. Therefore, we employed the Calgary biofilm device,^[^ ^27^ ^]^ which allows straightforward analysis of biofilm susceptibility toward different agents. Briefly, biofilms were grown on pegs in presence/absence of **24** for 48 h. After that, biofilms were disrupted using sonication, followed by colony‐forming units (CFU) determination, which gave access to the amount of viable cells in the biofilm.^[^ ^27^, ^28^ ^]^

We observed that **24** significantly attenuates PA14 biofilm formation compared to a DMSO control when present in the growth medium from the beginning (Figure S1A, Supporting Information) and 8 h after the onset of biofilm growth (Figure S1B, Supporting Information). In a next step, we investigated the impact of this effect on antibiotic treatment of the biofilms. To this end, we used the SoC aminoglycoside tobramycin as the antibiotic backbone^[^ ^29^ ^]^ and analyzed its activity in the peg lid model after pretreating biofilms with different concentrations of **24** (Figure 4; see also Figure S1D,E, Supporting Information). Tobramycin was used at a concentration of 0.5 µg mL^−1^ and exerted only minor effects on untreated biofilms (*p* = 0.03). However, biofilms grown in presence of 1 and 10 × 10^−6^ m of **24** were much more susceptible to tobramycin treatment than untreated biofilms (*p* < 0.001). Strikingly, combination treatment caused a >3‐fold higher CFU reduction compared to single treatment with the antibiotic. Likewise, CFU reduction by 1 and 10 × 10^−6^ m of **24** was significantly higher in combination with tobramycin (*p* < 0.001). It is particularly intriguing that the subefficacious pathoblocker dose of 1 × 10^−6^ m (*p* = 0.93) still benefits tobramycin activity. This could be related to the submicromolar efficacy on eDNA release, which is a key mediator of tobramycin resistance.^[^ ^29^ ^]^ The 0.1 × 10^−6^ m **24** was not sufficient to do so (*p* = 0.21), demonstrating dose‐dependency of the observed effect. This effect could further be translated to the aminoglycoside amikacin, whose activity on PAO1 was also boosted in presence of 10 × 10^−6^ m **24** (see Figure S1C, Supporting Information). In total, these findings highlight the potential of combination treatment to enhance the efficiency of *P. aeruginosa* biofilm eradication by conventional antibiotics.

#### 2.3.3 Antivirulence Efficacy Against Clinical Isolates of PA from Bronchiectasis Patients

In order to test the efficacy of **24** to inhibit PqsR of other non‐CF *P. aeruginosa* strains, a set of 14 clinical isolates obtained from bronchiectasis patients were treated with the compound and the corresponding inhibition of pyocyanin production was quantified (Figure 4D). The collection contains representative isolates of the major clones in the *P. aeruginosa* population and pairs of clone B421 isolates from the same respiratory secretion (Table S2, Supporting Information). Moreover, the analyzed 14 *P. aeruginosa* strains revealed a high degree of variability in important phenotypical and virulence‐associated traits including the production of virulence factors (pyocyanin and pyoverdine), biofilm formation, and motility (swimming and swarming) (Figure S2, Supporting Information). The high strain variability observed between distinct clones and within clones from separate and matching habitats allowed for the parallel testing of high or low pyocyanin producers. Overall, treating the 14 clinical isolates with compound **24** caused a dramatic reduction in the production of pyocyanin by all strains, especially clear‐cut results were observed for *P. aeruginosa* strains that naturally produced high amounts of pyocyanin (Bron 8, 27, 46, 59, 76; Figure 4D). In summary, these results confirmed the broad activity of **24** in efficiently targeting the PQS signaling system of not only *P. aeruginosa* laboratory strains, but also of bronchiectasis‐derived clinical isolates.

In view of the promising in vitro efficacy data, frontrunner **24** was further profiled in terms of in vitro pharmacokinetics and safety.

### 2.4 In Vitro ADME Profiling

#### 2.4.1 Metabolism

Compound **24** showed acceptable metabolic stability in MLM (Table 3). Hence, it was further profiled using mouse liver S9 fractions (*t*_1/2_ 50 min) as well as hepatocytes (*t*_1/2_ 9 min, **Table** **5**). The results prompted us to investigate different routes of administration in order to assess the impact of drug metabolism on systemic compound availability.

**Table 5:** Primary in vitro pharmacokinetic (PK) data for **24**

Parameter	Value
Kinetic solubility [×10^−6^ m]	47.2
Calu‐3 (P_app_) [10–6 cm s^−1^]	3.1 ± 0.28
Mouse liver S9 *t*½ [min]	50
Mouse hepatocytes *t*½ [min]	9
Plasma binding human [%]	>99
Plasma binding murine [%]	>99

#### 2.4.2 Plasma Protein Binding

Plasma protein binding (PPB) was measured against human and murine plasma and determined to be very high (>99%) in both cases. High plasma protein binding can be favorable in terms of systemic exposure, as it can prolong circulation times. However, very high PPB might lead to disadvantages such as decreased availability of free drug for interaction with its target, unfavorably long elimination rates, and risk of drug accumulation. In order to understand whether the observed parameters are suitable for in vivo efficacy and reasonable clearance we conducted an in vivo PK study (vide infra).

#### 2.4.3 Permeability Across Lung Epithelial Cell Line Calu‐3

Transepithelial apparent permeability of compound **24** was assayed in the lung epithelial cell line Calu‐3, which were grown at an air–liquid interface until a stable transepithelial electrical resistance (TEER) was reached. Wells with a TEER < 300 Ω × cm^2^ were excluded from analysis. Reference compound ciprofloxacin exhibited an intermediate apparent permeability with 2.5 × 10^−6^ ± 8.9 × 10^−7^ cm × s^−1^ at 1 × 10^−6^ m, which is in accordance with literature.^[^ ^30^ ^]^ Compound **24** showed intermediate permeability with an apparent permeability value of 3.1 × 10^−6^ ± 2.8 × 10^−7^ cm × s^−1^ at 1 × 10^−6^ m, which was comparable to ciprofloxacin (Figure S2, Supporting Information).

### 2.5 In Vitro Safety Pharmacology

In order to assess potential safety risks of **24**, we subjected it to primary safety‐pharmacology assays, including hERG inhibition, CYP inhibition, AhR activation, Mini‐AMES, as well as a CEREP‐44 off‐target panel, which were conducted at the CROs Cyprotex and Eurofins (**Table** **6**).

**Table 6:** Primary safety‐pharmacology panel of **24** and functional assessment of CEREP off‐targets of **24** (de‐risking)

Assay target	IC50 [×10^−6^ m]	Assay method/note
hERG inhibition	15.8	Electrophysiology
fold AhR receptor induction	0.91 @1 × 10^−6^ m ^a)^	Luciferase reporter gene assay
CYP3A4 inhibition	>25^b)^	Metabolic activity
CYP2D6 inhibition	>25	Metabolic activity
CYP1A inhibition	>25	Metabolic activity
CYP2C9 inhibition	>25	Metabolic activity
CYP2C19 inhibition	>25	Metabolic activity
Mini‐AMES		Negative @62.25 µg mL^−1^ (147 × 10^−6^ m)
Ca2+ channel inhibition, Cerep 161	>30	Binding, antagonist
Norepinephrine transporter, Cerep 355	>10	Binding, antagonist
5‐HT2B receptor, Cerep 1333	>10	Binding, agonist
Na+ channel, Cerep 169	>30	Binding, antagonist

^a)^ fold activation at given concentration

^b)^ activation observed.

A functional electrophysiology hERG assay revealed an IC_50_ value of 15.8 × 10^−6^ m. In the context of the observed hERG activity, the aforementioned high PPB might lead to a suitable safety window for application humans as clinical outcome is correlating well with the margin provided by hERG IC_50_ and the peak free plasma exposure.^[^ ^31^ ^]^ With the aim to further explore potential ion‐channel inhibition by **24**, 13 additional ion channels were investigated at Charles River Laboratories. Functional inhibition was observed only for hK_v_1.5 (IC_50_ ≈30 × 10^−6^ m), while for all other targets tested IC_50_ values were >30 × 10^−6^ m (see Supporting Information).

Neither AhR induction nor significant inhibition of CYP2D6, CYP1A, CYP2C9, and CYP2C19 was observed up to 25 × 10^−6^ m. However, for CYP3A4 activation >50% was observed at 10 × 10^−6^ m. In the mini‐AMES test, no genotoxicity was observed for **24** at 62.25 µg mL^−1^. This was also the case in presence of rat liver S9 fractions, indicating that no toxic metabolites were formed under the assay conditions.

We also subjected **24** to the SafetyScreen44 (Eurofins) involving 38 binding assays and six functional assays. At 10 × 10^−6^ m compound **24** did not exert effects >80% on any target. However, we observed >50% binding to human norepinephrine transporter, Ca_v_1.2 L‐type rat calcium ion channel, 5‐HT_2B_ human serotonin receptor, nonselective rat sodium ion channel, and hERG human potassium ion channel. The latter is in accordance with the moderate activity already observed in the functional assay of the primary test panel (vide supra). For the other potential off‐targets, we also conducted functional assays, revealing that the observed binding events did not translate into functional inhibition.

While the overall safety pharmacology profile of this compound is balanced, the moderate hERG inhibition on a functional level should be taken into account in the context of plasma protein binding (vide infra), which is high and might, hence, provide a safety margin. Nevertheless, this should be investigated more closely, if the compound was moved to preclinical development.^[^ ^31^ ^]^

### 2.6 In Vivo Pharmacokinetics and Safety

#### 2.6.1 Pulmonary and Systemic In Vivo PK Studies

In order to assess distribution into the target compartments, namely epithelial lining fluid (ELF) and lung tissue, as well as systemic exposure in plasma, we conducted PK studies, starting with the intravenous route to determine absolute bioavailability. In addition, we assayed exposure after local, intratracheal (IT) administration. PK studies were performed for **24** and tobramycin to identify differences in exposure and kinetics. Compound **24** and tobramycin showed similar levels in lung tissue after administration of 0.25 mg kg^−1^ IT. However, **24** exhibited much higher levels as well as exposure in ELF than tobramycin. Of note, **24** also showed higher exposure in plasma compared to tobramycin after IT administration (Figure S6, Supporting Information). With respect to the IV route, **24** again showed higher exposure and a longer half‐life in plasma. Moreover, **24** harbored much higher levels in ELF than tobramycin after IV administration (Figure S6, Supporting Information). Additionally, **24** was also detected in lung tissue after IV administration, whereas no tobramycin was found in lung tissue after dosing 0.25 mg kg^−1^ IV. After IT administration, **24** and tobramycin showed similar exposures in lung tissue (Figure S4, Supporting Information). Based on the encouraging IV data of **24**, next, we conducted PK studies with the intraperitoneal (IP), subcutaneous (SC), and oral (per os; PO) route to determine the best route for a subsequent in vivo efficacy model (Figure S7, Supporting Information). The IP route gave the best exposure in plasma (bioavailability compared to the IV route of around 36%) followed by the SC route, which did not result in immediate peak concentrations and a delayed *T*_max_. The oral route resulted in only half of the exposure of the IP route. Given similar exposures in plasma, we selected the SC route for determination of the maximal tolerated dose.

#### 2.6.2 Maximum Tolerated Dose in Mice

In preparation for the testing of in vivo efficacy, the maximum tolerated dose of **24** in mice was determined at a CRO (Aurigon). After subcutaneous administration of 10, 30, and 60 mg kg^−1^, no mortality or loss of body weight were observed during 48 h. Mice treated with 10 and 30 mg kg^−1^ were devoid of any clinical findings. In case of the 60 mg kg^−1^ dose, only slight, transient findings were observed in single animals at 2 and 4 h, namely piloerection and decreased activity (3 of 10 mice). These observations were sex‐independent and not present after 4 h in any of the mice, which could be due to clearance of the compound. Analysis of plasma samples obtained at 0.5 and 4 h of treatment revealed generally higher compound levels at 4 h, supporting this hypothesis. This analysis further showed slightly higher plasma levels in male mice for the doses of 30 and 60 mg kg^−1^, whereas no differences between males and females were detected at 10 mg kg^−1^ (Figure S4, Supporting Information). In general, the study confirms that the compound is well tolerated both in male and female mice.

### 2.7 In Vivo Proof‐of‐Concept

**Figure 5:**
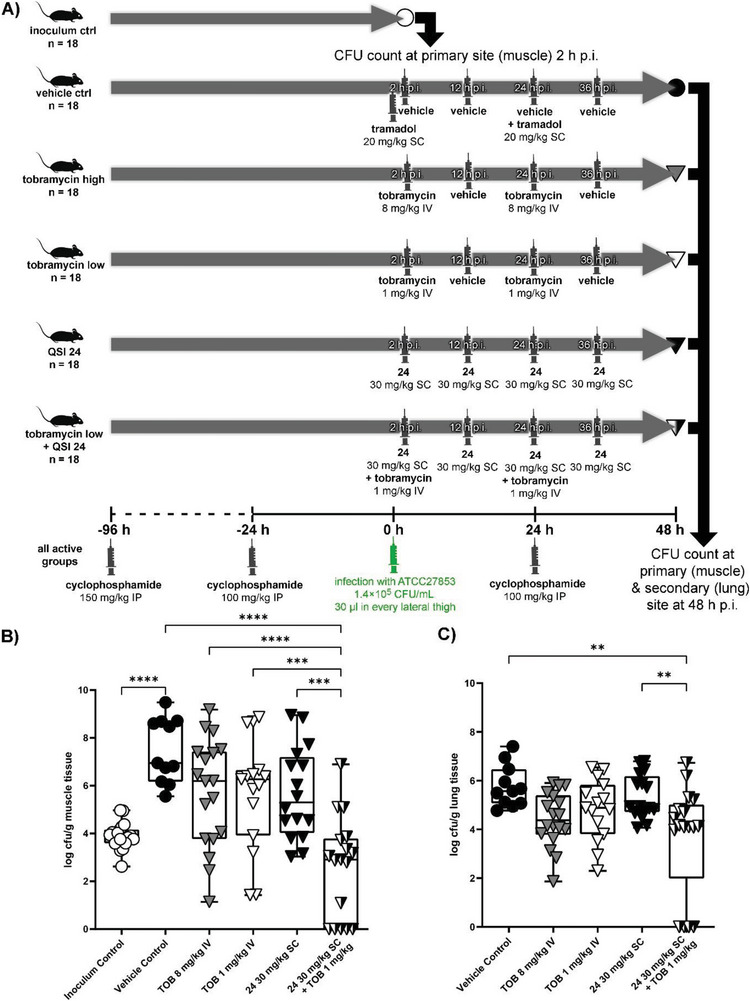
In vivo efficacy testing using a neutropenic thigh‐infection model in mice. A) Schematic overview of the experimental layout depicting groups and treatment regimens. B) Bacterial burden detected in the primary infection site (muscle). C) Bacterial burden detected in the secondary infection site (lung). ^****^ *p* < 0.0001, ^***^ *p* < 0.001, ^**^ *p* < 0.01. (CFU: colony‐forming unit; p.i.: post infection; IP: intraperitoneal; SC: subcutaneous; IV: intravenous; QSI: quorum sensing inhibitor; TOB: tobramycin).

After demonstrating favorable in vivo PK and safety characteristics as well as promising potency in in vitro activity assays, we were prompted to test compound **24** in a murine infection model. Selecting a suitable model for assessing the efficacy of a molecule with a pathoblocker‐type mode‐of‐action is key. A typical endpoint, for evaluating anti‐infectives is “bacterial load” measured as CFU at the respective site of infection.^[^ ^32^ ^]^ As compound **24** itself should not have a direct impact on bacterial viability and, hence, bacterial load, monotherapy was not expected to yield a dramatic reduction in CFU. For this reason, we opted for combination experiments together with backbone antibiotics. Considering the effects on PA biofilms shown before and in this study (Figure 4),^[^ ^20^ ^]^ the aminoglycoside tobramycin was the combination partner of choice. Moreover, we chose an ineffective dose of tobramycin (in our case 1 mg kg^−1^ IV) for the combination to assess if **24** in combination with tobramycin shows a synergistic effect and to determine if combination with **24** renders tobramycin more effective. Consequently, we also used a treatment group with **24** alone as well as with the, otherwise, ineffective dose of 1 mg kg^−1^ IV every 24 h tobramycin alone to show that possible effects are attributable to the combination. Furthermore, we decided to use a simple and robust test system to mitigate difficulties arising from intragroup variations when attempting PA infections in murine hosts.^[^ ^33^ ^]^ For these reasons, we opted for a thigh‐infection model in neutropenic mice as a surrogate model, which we extended to 48 h. To secure a maintained neutropenia for 48 h, animals were rendered neutropenic before infection and received an additional dose of cyclophosphamide 100 mg kg^−1^ intraperitoneally on day 1 postinfection. Although the neutropenic thigh‐infection model is not a chronic lung‐infection model, which might be closer to the clinical condition envisioned for our approach, it is accepted as an important test system for modeling PK/PD relationships of PA‐targeting antibiotics (**Figure** **5**).^[^ ^32^ ^]^ Based on the PK data for **24**, we chose a dose of 30 mg kg^−1^ SC every 12 h starting 2 h postinfection. The experimental layout is depicted in more detail in Figure 5a.

When analyzing the outcome at the primary infection site (thigh muscle, Figure 5b), we observed a defined colonization and growth of the bacterial burden when no treatment was applied (compare groups “inoculum control” versus “vehicle control”). Notably, treatment groups with only a singular active agent (“tobramycin high”, “tobramycin low”, and “QSI **24**”) did not exert significant effects over vehicle in terms of CFU reduction. This drastically changed when combining the low dose tobramycin (1 mg kg^−1^) with QSI **24** (30 mg kg^−1^). This group employing an aminoglycoside antibiotic and an optimized PqsR inverse agonist showed a significantly reduced bacterial burden in the muscle compared to all other groups (*p* < 0.001). In other words, the presence of QSI **24** enhanced the efficacy of tobramycin‐mediated eradication and even surpassed the higher single dose of the antibiotic. This notion provides evidence for synergistic action of the employed active agents against PA infections.

Looking at the secondary site of infection (lung, Figure 5c), we observed the following trends. Tobramycin treatment using the high dosing scheme seemed to affect dissemination, even if it did not reach significance (*p* > 0.05). Along the same lines, the groups treated either with the low dose of aminoglycoside antibiotic or with QSI **24** alone also did not show a significant effect (*p* < 0.05). Notably, the only group, which achieved a detectable inhibition of dissemination to the lung, was treated with a combination of the low tobramycin dose together with QSI **24**. Moreover, we performed LC–MS/MS analysis of plasma and lung tissue samples of all groups. Whereas we did not detect any tobramycin terminally, we detected similar levels of QSI **24** in plasma as well as lung tissue for both treatment groups (with combination of low dose tobramycin and without tobramycin, Figure S8, Supporting Information). Consequently, this proves that differences in bacterial burden between the QSI **24**/tobramycin and QSI **24** only group are solely attributable to the effect resulting from the combination therapy.

Dissecting CFU data for both primary and secondary infection sites (muscle and lung), we observed two animals, which were apparently cleared of the PA infection within the combination group and did not yield a detectable bacterial burden. This finding was not observed in any of the other groups.

Taken together, the presented results provide strong evidence for the superiority of administering a QSI–tobramycin combination treatment over using monotherapy even at higher doses of the antibiotic (compare Tob 8 mg kg^−1^ versus Tob 1 mg kg^−1^ + QSI **24**).

## 3 Conclusion

Chronic and acute infections by PA remain an ever‐growing threat and, especially, carbapenem‐resistant strains have been identified as priority pathogens for which new treatment options are urgently needed.^[^ ^34^ ^]^ In the case of chronic lung infections the use of aminoglycoside antibiotics such as tobramycin can be considered as SoC. However, failure to eradicate PA infections, e.g., in cystic‐fibrosis patients is reported regularly.^[^ ^35^ ^]^ It is known that PA biofilms play a major role in chronic infections and that tobramycin suffers from reduced efficacy against sessile PA colonies.^[^ ^8^, ^10^ ^]^ Enhancing tobramycin‐driven biofilm eradication by adjunctive agents such as QSIs might help to overcome this problem.

In this study, we conducted medicinal chemistry‐driven optimization of a fragment‐like hit **1** to an in vivo active precandidate (QSI **24**). This compound demonstrates antivirulence activity (pyocyanin inhibition) in the same potency range as other reported frontrunner molecules combined with promising PK properties and clean safety pharmacology profile.^[^ ^20^, ^21^, ^22^, ^36^ ^]^ The molecule is well‐tolerated in mice and achieves high exposures in the lung via various routes of application. It enhances the effect of the aminoglycoside antibiotic tobramycin on PA biofilms and, importantly, synergizes with backbone antibiosis in a neutropenic thigh‐infection model in mice. Important next steps in the profiling of this new promising compound are to establish PK/PD relationships in vivo and to include also chronic lung‐infection models as well as using various routes of administration in order to understand and learn how to optimally put the underlying mode‐of‐action to use in a preclinical setting. Hence, this study sets a cornerstone for enabling the exploration of the translational potential of PqsR‐targeting QSIs against PA infections.

Finally, we see great potential in further investigating nanocarrier‐based formulations as an additional innovative component. As we have shown recently, application of tailor‐made nanoparticles can provide further boosts in biofilm‐eradicating properties of tobramycin.^[^ ^20^, ^37^ ^]^ These delivery systems will be investigated for their compatibility with the chemical matter reported herein in future studies.

## 4 Experimental Section

Materials and experimental details are provided in the Supporting Information. The animal studies were conducted in accordance with the recommendations of the European Community (Directive 86/609/EEC, 24 November 1986). All animal procedures were performed in strict accordance with the German regulations of the Society for Laboratory Animal Science (GV‐ SOLAS) and the European Health Law of the Federation of Laboratory Animal Science Associations (FELASA). Animals were excluded from further analysis if sacrifice was necessary according to the humane endpoints established by the ethical board. Animal experiments were approved by the ethical board of the Niedersächsisches Landesamt für Verbraucherschutz und Lebensmittelsicherheit, Oldenburg, Germany. Animals were kept in individually ventilated cages with a 10 h/14 h dark/light cycle and had access to food and water ad libitum.

## Conflict of Interest

A.S.A., M.E., M.H., R.W.H., T.R., C.S., and A.K.H.H. have filed patent WO2020007938 (EP18181475) “PqsR Inverse Agonists”, which claims intellectual property for the structures published herein. F.C.R. reports grants from the German Center for Lung Research (DZL), the German Center for Infection Research (DZIF), the Innovative Medicines Initiative (IMI; EU/EFPIA), and the iABC Consortium […]—more info in the manuscript.

## Supporting information

Supporting Information

## Data Availability

The data that support the findings of this study are available from the corresponding author upon reasonable request.
