# Supplementary material for: Towards Translation of PqsR Inverse Agonists: From In Vitro Efficacy Optimization to In Vivo Proof‐of‐Principle
Source: Adv Sci (Weinh). 2023 Jan 3;10(5):2204443. doi: 10.1002/advs.202204443 (PMC9929129; doi:10.1002/advs.202204443)
Supplement: Supplementary file 1 — Supporting Information [file ADVS-10-2204443-s001.pdf]

ADVANCED SCIENCE
Open Access

Supporting Information

for *Adv. Sci.*, DOI 10.1002/advs.202204443

Towards Translation of PqsR Inverse Agonists: From In Vitro Efficacy Optimization to In Vivo Proof-of-Principle

*Mostafa M. Hamed, Ahmed S. Abdelsamie, Katharina Rox, Christian Schütz, Andreas M. Kany, Teresa Röhrig, Stefan Schmelz, Wulf Blankenfeldt, Alejandro Arce-Rodriguez, José Manuel Borrero-de Acuña, Dieter Jahn, Jessica Rademacher, Felix C. Ringshausen, Nina Cramer, Burkhard Tümmler, Anna K. H. Hirsch, Rolf W. Hartmann and Martin Empting**

Towards translation of PqsR inverse agonists: from *in vitro* efficacy optimization to *in vivo* proof-of-principle

*Mostafa M. Hamed, Ahmed S. Abdelsamie, Katharina Rox, Christian Schütz, Andreas M. Kany, Teresa Röhrig, Stefan Schmelz, Wulf Blankenfeldt, Alejandro Arce-Rodriguez, José Manuel Borrero-de Acuña, Dieter Jahn, Jessica Rademacher, Felix C. Ringshausen, Nina Cramer, Burkhard Tümmler, Anna K. H. Hirsch, Rolf W. Hartmann, Martin Empting**

M. M. Hamed, A. S. Abdelsamie, C. Schütz, A. M. Kany, T. Röhrig, A. K. H. Hirsch, R. W. Hartmann, M. Empting
Helmholtz-Institute for Pharmaceutical Research Saarland (HIPS)-Helmholtz Centre for Infection Research (HZI) Campus E8.1, Saarbrücken 66123, Germany.
E-mail:Martin.empting@helmholtz-hzi.de

M. M. Hamed, A S. Abdelsamie, K. Rox, C Schütz, A. M. Kany, T Röhrig, A. K. H. Hirsch, R. W. Hartmann, M. Empting
German Centre for Infection Research (DZIF) Partner Site Hannover-Braunschweig Saarbrücken 66123, Germany.

A. S. Abdelsamie
Department of Chemistry of Natural and Microbial Products, Institute of Pharmaceutical and Drug Industries Research, National Research Centre, El-Buhouth St., Dokki, P.O. Box 12622, Cairo, Egypt.

K. Rox
Department of Chemical Biology (CBIO) Helmholtz Centre for Infection Research (HZI) Inhoffenstr. 7 Braunschweig 38124, Germany.

S. Schmelz, W. Blankenfeldt
Department of Structure and Function of Proteins (SFPR) Helmholtz Centre for Infection Research (HZI) Inhoffenstr. 7 Braunschweig 38124, Germany.

W. Blankenfledt
Institute for Biochemistry, Biotechnology and Bioinformatics, Technische Universität Braunschweig, Braunschweig, Germany.

A. Arce-Rodriguez, J. M. Borrero-de Acuña, D. Jahn

Institute of Microbiology, Technische Universität Braunschweig, Braunschweig 38106, Germany

J. M. Borrero-de Acuña, D. Jahn

Braunschweig Integrated Centre of Systems Biology (BRICS), Technische Universität Braunschweig, Braunschweig 38106, Germany

J. M. Borrero-de Acuña

Departamento de Microbiología, Facultad de Biología, Universidad de Sevilla, Av. de la Reina Mercedes, no. 6, Sevilla, CP 41012, Spain

N. Cramer, B. Tümmler

Department for Pediatric Pneumology, Allergology and Neonatology, Medizinische Hochschule Hannover, Carl-Neuberg-Str. 1, Hannover 30625, Germany

J. Rademacher, F.C. Ringshausen

Department for Respiratory Medicine, Medizinische Hochschule Hannover, Carl-Neuberg-Str. 1, Hannover 30625, Germany;

J. Rademacher, F.C. Ringshausen, B. Tümmler

Biomedical Research in Endstage and Obstructive Lung Disease (BREATH), German Center for Lung Research (DZL), Hannover 30625, Germany

F.C. Ringshausen

European Reference Network on Rare and Complex Respiratory Diseases (ERN- LUNG), Frankfurt, Germany

C. Schütz, A. K. H. Hirsch, R. W. Hartmann, M. Empting

Department of Pharmacy, Saarland University Campus E8.1, Saarbrücken 66123, Germany

Table of Contents

1 EXPERIMENTAL 5

1.1 CHEMISTRY 5

1.1.1 Materials and Methods 5

1.1.2 General Information 5

1.1.3 Synthetic Strategy 6

1.1.4 Preparative RP-HPLC purifications 9

1.1.5 Experimental procedures 9

1.2 BIOLOGY 27

1.2.1 PqsR reporter gene assay in *Escherichia coli* 27

1.2.2 Pyocyanin assay in *Pseudomonas aeruginosa* 27

1.2.3 Alkylquinolone quantification 27

1.2.4 Biofilm-formation assay 28

1.2.5 Biofilm-eradication assay 29

1.2.6 eDNA assay 31

1.2.7 *P. aeruginosa* clinical isolates from bronchiectasis patients – phenotypic characterization and inhibition of pyocyanin production 31

1.3 DMPK 36

1.3.1 Apparent permeability in Calu-3 cell line 36

1.3.2 Metabolic-Stability Tests in Mouse Liver Microsomes (MLM). 37

1.3.3 PK and PD Sample Preparation and Analysis 37

1.3.4 Kinetic Turbidimetric Aqueous Solubility 40

1.4 Safety Pharmacology of Compound 24 41

1.4.1 Cerep panel (Eurofins) 41

1.4.2 Further functional off-target assays (Eurofins) 43

1.4.3 Ion Channel Profiling at Charles River 43

1.4.4 Pharmacokinetic and off-target functional assays (Cyprotex) 44

1.4.5 Mini-AMES (Cyprotex) 44

1.4.6 Mouse Maximum tolerated dose (MTD) study (Aurigon) 45

1.4.7 *In vivo* PK studies 46

1.4.8 *In vivo* thigh infection model with *P. aeruginosa* 50

1.5 X-ray crystallography 52

1.5.1 Expression and Purification of PqsR$^{91-319}$ 52
1.5.2 Protein Crystallization, Data Collection and Structure Solution 52
1.6 Molecular modeling 54
1.7 Statistics 55
2 ^{1}H- AND ^{13}C-NMR SPECTRA OF THE DESCRIBED COMPOUNDS 57
3 HRMS SPECTRA OF THE DESCRIBED COMPOUNDS 81
4 HPLC traces 93
5 REFERENCES 105

1 EXPERIMENTAL

1.1 CHEMISTRY

1.1.1 Materials and Methods

Starting materials and solvents were purchased from commercial suppliers, and used without further purification. All chemical yields refer to purified compounds and were not optimized. Reaction progress was monitored using TLC silica gel 60 F_{254} aluminum sheets, and visualization was accomplished by UV at 254 nm. Column chromatography was performed using the automated flash chromatography system CombiFlash® Rf (Teledyne Isco) equipped with RediSepRf silica columns. Preparative RP-HPLC was was perofmed using an UltiMate 3000 Semi-Preparative System (Thermo Fisher Scientific) with nucleodur® C18 Gravity (250 mm × 10 mm, 5 µm) column. ^{1}H and ^{13}C NMR spectra were recorded as indicated on a Bruker Avance Neo 500 MHz (^{1}H, 500 MHz; ^{13}C, 126 MHz) with prodigy cryoprobe system or a Bruker Fourier 300 (^{1}H, 300 MHz; ^{13}C, 75 MHz) instrument. Chemical shifts were recorded as δ values in ppm units and referenced against the residual solvent peak ($CDCl_3$, δ = 7.26, 77.16; DMSO-d_6, δ = 2.50, 39.52, acetone-d_6: δ = 2.05, 29.84). Splitting patterns describe apparent multiplicities and are designated as s (singlet), br s (broad singlet), d (doublet), dd (doublet of doublet), t (triplet), q (quartet), m (multiplet). Coupling constants (*J*) are given in hertz (Hz). Low resolution mass analytics and purity control of final compounds was carried out either using an Ultimate 3000-MSQ LCMS system (Thermo Fisher Scientific) consisting of a pump, an autosampler, MWD detector and a ESI quadrupole mass spectrometer. Purity of all compounds used in biological assays was ≥ 95%. High resolution mass spectra were recorded on a ThermoFisher Scientific (TF, Dreieich, Germany) Q Exactive Focus system equipped with heated electrospray ionization (HESI)-II source. Final products were dried at high vacuum.

1.1.2 General Information

Chemical and Analytical Methods. ^{1}H- and ^{13}C-NMR spectra were recorded as indicated on a Bruker Avance Neo 500 MHz with prodigy cryoprobe system or a Bruker Fourier 300 instrument. Chemical shifts are given in parts per million (ppm), and referenced against the residual solvent peak. Coupling constants (J) are given in Hertz. Low-resolution mass analytics and purity control of final compounds were carried out either using an Ultimate 3000-MSQ LCMS system (Thermo Fisher Scientific), consisting of a pump, an autosampler, MWD detector and an ESI quadrupole mass spectrometer.

High resolution mass spectra were recorded on a ThermoFisher Scientific (TF, Dreieich, Germany) Q Exactive Focus system equipped with heated electrospray ionization (HESI)-II source. Reagents were used as obtained from commercial suppliers without further purification. Procedures were not optimized regarding yield. Column chromatography was performed using the automated flash chromatography system CombiFlash® Rf (Teledyne Isco) equipped with RediSepRf silica columns. Preparative HPLC was performed using an UltiMate 3000 Semi-Preparative System (Thermo Fisher Scientific) with nucleodur® C18 Gravity (250 mm × 10 mm, 5 µm) column. Final products were dried at high vacuum.

General Method A: General S_N2 Reaction. To *tert*-butyl(2-(trifluoromethyl)pyridin-4-yl)carbamate (**27**) (1 mmol) in DMF (5 mL) at 0 °C was added NaH (60wt%) (1.2 mmol). The reaction was stirred for 30 min followed by the addition of the corresponding benzyl bromide derivative (**28a–28h**) (1.2 mmol). The reaction mixture was stirred at room temp. for 2 h. The reaction was then added to ice water followed by extraction with ethyl acetate. The combined organic layers were dried over $MgSO_4$, filtered and concentrated under reduced pressure. Purification was done using automated column chromatography to yield compounds (**29a–29h**).

General Method B: General Deprotection Reaction. To the corresponding derivative (**29a–29h**) (1 mmol) dissolved in DCM (5 mL) was added TFA (1 mL). After stirring at r.t. for 5 h, the mixture was cooled to 0 °C followed by the addition of saturated $NaHCO_3$ solution and extraction with DCM. The combined organic layers were dried over $MgSO_4$, filtered and concentrated under reduced pressure. Purification was done using automated column chromatography or preparative HPLC to yield compounds (**2**–**6, 30**–**32**).

General Method C: General Suzuki Coupling. In a flask containing the corresponding derivative (**4, 30**–**32, 34**–**35**), the corresponding boronic acid derivative (1.3 mmol) and $Pd(PPh_3)_4$ (0.05 mmol) were added 25 mL of the dioxane/H2O mixture (4:1) and Na2CO3 (4 mmol). The reaction was refluxed at 110 °C for 3 h under a N2 atmosphere. Excess solvent was evaporated under reduced pressure, and then water (30 mL) was added followed by extraction with ethyl acetate. The combined organic layers were dried over $MgSO_4$, filtered and concentrated under reduced pressure. Purification was done using preparative HPLC to yield compounds (**7**–**25**).

1.1.3 **Synthetic Strategy**

Synthesis of compounds **2–6** and the intermediates **30–32** was achieved *via* the route depicted in Scheme S1. Notably, a double Boc-protection and subsequent mono-deprotection strategy was

essential to prepare the scaffold for successful mono benzylation. In detail, amino protection of the 2-(trifluoromethyl)pyridin-4-amine using Boc anhydride yielded compound **26**. Then, we generated mono-deprotected derivative **27** by applying TFA. Benzylation of **27** was achieved using different benzyl bromide derivatives **28a**–**28h** in DMF in presence of NaH, affording compounds **29a**–**29h**. The different benzyl bromide derivatives **28a**–**28h** were either commercially available (**28a**–**28f**) or prepared directly before reaction (**28g**–**28h**, see also the Supporting Information). Final deprotection was conducted by applying a more concentrated TFA solution to yield target molecules **2**–**6** and the intermediates **30**–**32**.

We synthesized compounds **7–11** and **14–25** by reacting intermediates **4**, **30–32** with various boronic acids under Suzuki_Miyaura conditions using $Pd(PPh_3)_4$ as catalyst (Scheme S2).

Reacting 2-(trifluoromethyl)pyridin-4-ol with Lawesson's reagent yielded thiol derivative **33**. Compound **28g** served as the benzylating agent for the generation of intermediates **34** and **35** using 2-(trifluoromethyl)pyridin-4-ol or **33**, respectively. Finally, compounds **12** and **13** were obtained by Suzuki–Miyaura coupling of (5-chloropyridin-2-yl)boronic acid with compounds **34** and **35**, respectively (Scheme S3).

Scheme S1. General scheme for the synthesis of compounds 2–6 and the intermediates 30–32

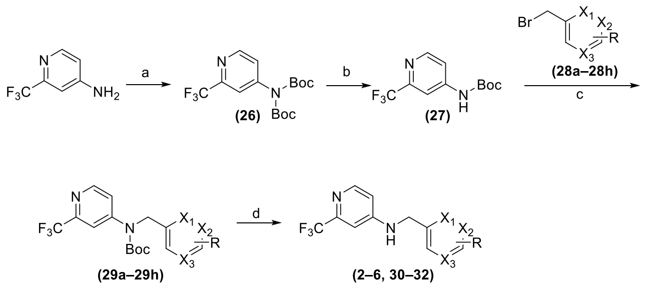

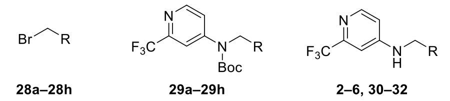

**28a, 29a, 2**	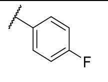	**28e, 29e, 6**	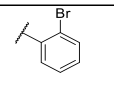
**28b, 29b, 3**	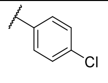	**28f, 29f, 30**	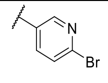
**28c, 29c, 4**	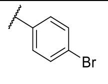	**28g, 29g, 31**	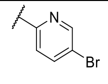
**28d, 29d, 5**	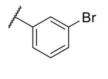	**28h, 29h, 32**	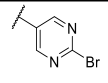

Reagents and Conditions: (a) Boc anhydride, DMAP, TEA, THF, rt; (b) TFA/DCM 1:10, rt, 15min; (c) Method A: NaH, DMF, rt; (d) Method B. TFA/DCM 2:10, rt, 5h.

Scheme S2. General scheme for the synthesis of compounds 7–11 and 14–25.

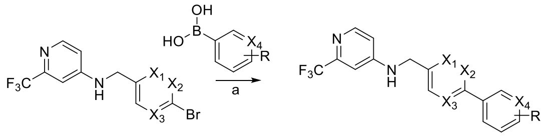

(4, 30-32) (7-11, 14-25)

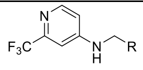

7-11, 14-25

**7**	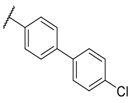	**18**	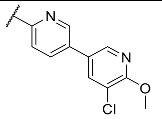
**8**	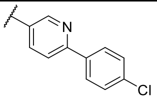	**19**	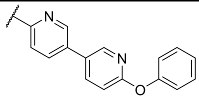
**9**	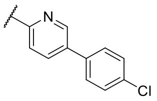	**20**	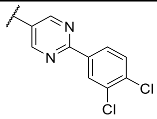
**10**	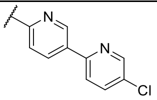	**21**	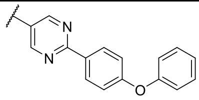
**11**	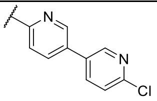	**22**	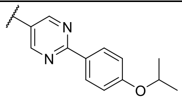
**14**	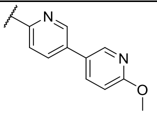	**23**	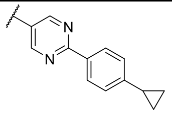
**15**	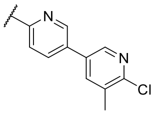	**24**	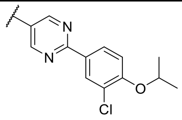
**16**	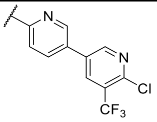	**25**	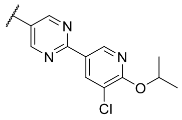
**17**	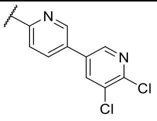		

Reagents and Conditions: (a) Method C: boronic acid derivatives, $Pd(PPh_3)_4$, Na_2CO_3, dioxane/H_2O 4:1, 110 °C.

Scheme S3. Synthesis of compounds 12 and 13.

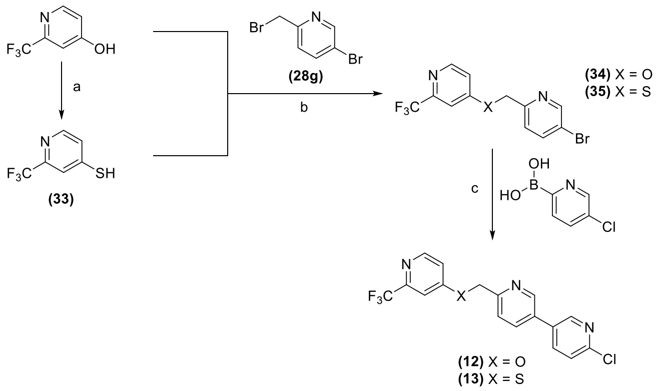

Reagents and Conditions: (a) Lawesson's reagent, toluene; (b) 5-bromo-2-(bromomethyl)pyridine, K_2CO_3, acetone or DMF; (c) Method C: (5-chloropyridin-2-yl)boronic acid, $Pd(PPh_3)_4$, Na_2CO_3, dioxane/H_2O 4:1, 110 °C.

1.1.4 **Preparative RP-HPLC purifications**

Purifications *via* preparative RP-HPLC were carried out using two possible conditions:

Condition A: gradient 5–100% CH_3CN + 0.05% HCOOH in water + 0.05% HCOOH in 53 min at a flow rate of 5 mL/min. The sample was dissolved in DMSO and manually injected to the HPLC system.

Condition B: gradient 10–100% CH_3CN + 0.05% HCOOH in water + 0.05% HCOOH in 35 min at a flow rate of 5 mL/min. The sample was dissolved in DMSO and manually injected to the HPLC system.

1.1.5 **Experimental procedures**

N-(4-Fluorobenzyl)-2-(trifluoromethyl)pyridin-4-amine (**2**)

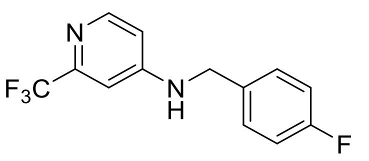

Compound **2** was synthesized from compound (**28a**) according to the general method B. The crude product was purified by preparative HPLC (Condition A, eluted at 50% ACN, 80 % yield). ^{1}H NMR (500 MHz, $(CD_3)_2CO$) δ 8.18 (d, *J* = 5.7 Hz, 1H), 7.47 – 7.42 (m, 2H), 7.14 – 7.09 (m, 2H), 7.02 (d, *J* = 2.1 Hz, 1H), 6.86 (s, 1H), 6.75 (dd, *J* = 5.6, 2.1 Hz, 1H), 4.51 (d, *J* = 5.8 Hz, 2H); ^{13}C NMR (126 MHz, $(CD_3)_2CO$) δ 162.9 (d, *J* = 243.0 Hz), 155.6, 150.9, 148.9 (q, *J* = 32.9 Hz), 135.3 (d, *J* = 3.3 Hz), 130.1 (d, *J* = 8.2 Hz), 123.1 (q, *J* = 273.7 Hz), 116.1 (d, *J* = 21.8 Hz), 110.0, 105.25, 46.2; MS (ESI+) *m/z* 271 $[M+H]^+$; HRMS (ESI+) *m/z* calcd for $C_{13}H_{11}F_4N_2$ $[M+H]^+$: 271.08584 found 271.08384.

N-(4-Chlorobenzyl)-2-(trifluoromethyl)pyridin-4-amine (**3**)

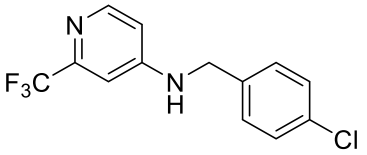

Compound **3** was synthesized from compound (**28b**) according to the general method B. The crude product was purified by preparative HPLC (Condition A, eluted at 55% ACN, 71% yield). ^{1}H NMR (500 MHz, $(CD_3)_2CO$) δ 8.18 (d, *J* = 5.7 Hz, 1H), 7.45 – 7.41 (m, 2H), 7.40 – 7.36 (m, 2H), 7.02 (d, *J* = 2.1 Hz, 1H), 6.89 (s, 1H), 6.74 (dd, J = 5.6, 2.1 Hz, 1H), 4.53 (d, *J* = 6.0 Hz, 2H); ^{13}C NMR (126 MHz, $(CD_3)_2CO$) δ 155.6, 151.0, 148.9 (q, *J* = 32.8 Hz), 138.3, 133.3, 129.8, 129.5, 123.1 (q, *J* = 273.7 Hz), 110.0, 105.3, 46.2; MS (ESI+) *m/z* 287 [*M*+H]$^+$; HRMS (ESI+) *m/z* calcd for $C_{13}H_{11}ClF_3N_2$ [*M*+H]$^+$: 287.05629 found 287.05429.

N-(4-Bromobenzyl)-2-(trifluoromethyl)pyridin-4-amine (**4**)

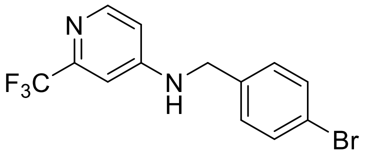

Compound **4** was synthesized from compound (**28c**) according to the general method B. The crude product was purified by preparative HPLC (Condition A, eluted at 60% ACN, 93% yield). ^{1}H NMR (500 MHz, $(CD_3)_2CO$ e) δ 8.18 (d, *J* = 5.7 Hz, 1H), 7.55 – 7.51 (m, 2H), 7.37 (d, *J* = 8.4 Hz, 2H), 7.02 (d, *J* = 2.0 Hz, 1H), 6.90 (s, 1H), 6.74 (dd, *J* = 5.6, 2.0 Hz, 1H), 4.52 (d, *J* = 5.9 Hz, 2H); ^{13}C NMR (126 MHz, $(CD_3)_2CO$) δ 155.6, 150.9, 148.9 (q, *J* = 32.9 Hz), 138.8, 132.5, 130.2, 123.1 (q, *J* = 273.6 Hz), 121.4, 110.0, 105.3, 46.2; MS (ESI+) *m/z* 331 [*M*+H]$^+$; HRMS (ESI+) *m/z* calcd for $C_{13}H_{11}BrF_3N_2$ [*M*+H]$^+$: 331.00577 found 331.00383.

N-(3-Bromobenzyl)-2-(trifluoromethyl)pyridin-4-amine (**5**)

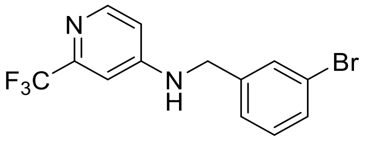

Compound **5** was synthesized from compound (**28d**) according to the general method B. The crude product was purified by preparative HPLC (Condition B, eluted at 65% ACN, 60% yield). ^{1}H NMR (500 MHz, $(CD_3)_2CO$) δ 8.19 (d, *J* = 5.7 Hz, 1H), 7.60 (s, 1H), 7.46 (d, *J* = 7.9 Hz, 1H), 7.42 (d, *J* = 7.7 Hz, 1H), 7.31 (t, *J* = 7.8 Hz, 1H), 7.04 (d, *J* = 2.0 Hz, 1H), 6.92 (s, 1H), 6.76 (dd, *J* = 5.6, 2.0 Hz, 1H), 4.55 (d, *J* = 6.0 Hz, 2H); ^{13}C NMR (126 MHz, $(CD_3)_2CO$) δ 155.6, 151.0, 149.0 (q, *J* = 32.9 Hz), 142.2, 131.5, 131.1,

131.0, 127.0, 123.4, 123.1 (q, *J* = 273.5 Hz), 110.0, 105.4, 46.2; MS (ESI+) *m/z* 331 [*M*+H]$^+$; HRMS (ESI+) *m/z* calcd for $C_{13}H_{11}BrF_3N_2$ [*M*+H]$^+$: 331.00577 found 331.00378.

N-(2-Bromobenzyl)-2-(trifluoromethyl)pyridin-4-amine (**6**)

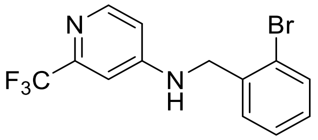

Compound **6** was synthesized from compound (**28e**) according to the general method B. The crude product was purified by preparative HPLC (Condition B, eluted at 65% ACN, 56% yield). ^{1}H NMR (500 MHz, $(CD_3)_2CO$) δ 8.21 (d, *J* = 5.7 Hz, 1H), 7.65 (dd, *J* = 8.0, 1.1 Hz, 1H), 7.44 (dd, *J* = 7.7, 1.4 Hz, 1H), 7.36 (td, *J* = 7.5, 1.1 Hz, 1H), 7.25 (td, *J* = 7.8, 1.7 Hz, 1H), 7.05 (s, 1H), 6.88 (s, 1H), 6.74 (d, *J* = 3.8 Hz, 1H), 4.56 (d, *J* = 5.9 Hz, 2H); ^{13}C NMR (126 MHz, $(CD_3)_2CO$) δ 155.5, 151.1, 149.0 (q, *J* = 32.8 Hz), 137.8, 133.8, 130.2, 130.0, 128.8, 123.8, 123.1 (q, *J* = 273.7 Hz), 109.9, 105.3, 47.3; MS (ESI+) *m/z* 331 [*M*+H]$^+$; HRMS (ESI+) *m/z* calcd for $C_{13}H_{11}BrF_3N_2$ [*M*+H]$^+$: 331.00577 found 331.00356.

N-((4'-Chloro-[1,1'-biphenyl]-4-yl)methyl)-2-(trifluoromethyl)pyridin-4-amine (**7**)

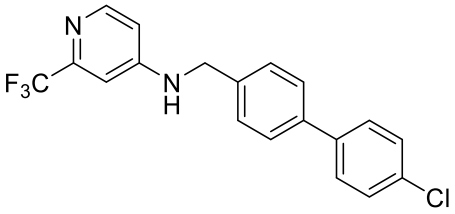

Compound **7** was synthesized according to Method C by reacting compound (**4**) with (4-chlorophenyl)boronic acid. The crude product was purified by preparative HPLC (Condition A, eluted at 70% ACN, 41% yield). ^{1}H NMR (500 MHz, $(CD_3)_2CO$) δ 8.19 (d, *J* = 5.7 Hz, 1H), 7.69 – 7.65 (m, 4H), 7.49 (ddd, *J* = 9.3, 7.7, 5.6 Hz, 4H), 7.06 (d, *J* = 1.8 Hz, 1H), 6.92 (s, 1H), 6.78 (dd, *J* = 5.6, 1.9 Hz, 1H), 4.57 (d, *J* = 5.9 Hz, 2H); ^{13}C NMR (126 MHz, $(CD_3)_2CO$) δ 155.7, 151.0, 148.9 (q, *J* = 32.6 Hz), 140.2, 139.5, 138.9, 133.8, 129.8, 129.3, 128.8, 127.9, 123.1 (q, *J* = 273.4 Hz), 109.9, 105.3, 46.5; MS (ESI+) *m/z* 363 [*M*+H]$^+$; HRMS (ESI+) *m/z* calcd for $C_{19}H_{15}ClF_3N_2$ [*M*+H]$^+$: 363.08759 found 363.08525.

N-((6-(4-Chlorophenyl)pyridin-3-yl)methyl)-2-(trifluoromethyl)pyridin-4-amine (**8**)

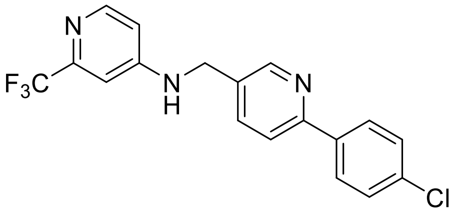

Compound **8** was synthesized according to Method C by reacting compound (**30**) with (4-chlorophenyl)boronic acid. The crude product was purified by preparative HPLC (Condition A, eluted at 60% ACN, 49% yield). ^{1}H NMR (500 MHz, $(CD_3)_2CO$) δ 8.73 (d, *J* = 1.6 Hz, 1H), 8.21 (d, *J* = 5.7 Hz, 1H), 8.15 – 8.12 (m, 2H), 7.94 (d, *J* = 7.8 Hz, 1H), 7.90 (dd, *J* = 8.2, 2.2 Hz, 1H), 7.52 – 7.49 (m, 2H), 7.08 (d, *J* = 2.2 Hz, 1H), 6.93 (s, 1H), 6.82 (dd, *J* = 5.7, 2.3 Hz, 1H), 4.64 (d, *J* = 5.9 Hz, 2H); ^{13}C NMR (126 MHz, $(CD_3)_2CO$) δ 155.5, 155.5, 151.1, 150.0, 149.0 (q, *J* = 32.9 Hz), 138.6, 137.1, 135.4, 133.8, 129.1, 129.1, 123.1 (q, *J* = 273.5 Hz), 120.7, 110., 105.4, 44.3; MS (ESI+) *m/z* 364 [*M*+H]$^+$; HRMS (ESI+) *m/z* calcd for $C_{18}H_{14}ClF_3N_3$ [*M*+H]$^+$: 364.08283 found 364.08066.

N-((5-(4-Chlorophenyl)pyridin-2-yl)methyl)-2-(trifluoromethyl)pyridin-4-amine (**9**)

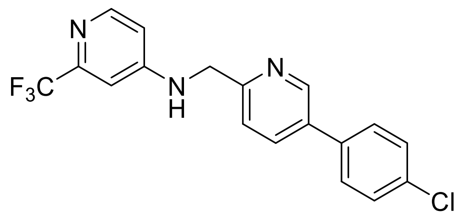

Compound **9** was synthesized according to Method C by reacting compound (**31**) with (4-chlorophenyl)boronic acid. The crude product was purified by preparative HPLC (Condition A, eluted at 60% ACN, 71% yield). ^{1}H NMR (500 MHz, $(CD_3)_2CO$) δ 8.86 (d, *J* = 2.2 Hz, 1H), 8.21 (d, *J* = 5.7 Hz, 1H), 8.05 (dd, *J* = 8.1, 2.4 Hz, 1H), 7.75 – 7.72 (m, 2H), 7.55 – 7.51 (m, 3H), 7.12 (s, 1H), 7.04 (s, 1H), 6.82 (d, *J* = 4.0 Hz, 1H), 4.66 (d, *J* = 5.8 Hz, 2H); ^{13}C NMR (126 MHz, $(CD_3)_2CO$) δ 157.9, 155.6, 151.0, 148.9 (q, *J* = 33.3 Hz), 148.2, 137.2, 135.8, 134.7, 134.6, 130.0, 129.5, 123.1 (q, *J* = 273.2 Hz), 122.3, 110.1, 105.4, 48.2; MS (ESI+) *m/z* 364 [*M*+H]$^+$; HRMS (ESI+) *m/z* calcd for $C_{18}H_{14}ClF_3N_3$ [*M*+H]$^+$: 364.08283 found 364.08054.

N-((5-Chloro-[2,3'-bipyridin]-6'-yl)methyl)-2-(trifluoromethyl)pyridin-4-amine (**10**)

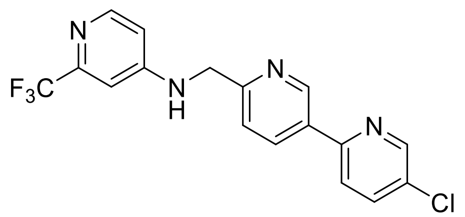

Compound **10** was synthesized according to Method C by reacting compound (**31**) with (5-chloropyridin-2-yl)boronic acid. The crude product was purified by preparative HPLC (Condition A, eluted at 50% ACN, 40% yield). ^{1}H NMR (500 MHz, $(CD_3)_2CO$) δ 9.25 (d, *J* = 1.7 Hz, 1H), 8.69 (d, *J* = 2.0 Hz, 1H), 8.43 (dd, *J* = 8.2, 2.3 Hz, 1H), 8.20 (d, *J* = 5.7 Hz, 1H), 8.06 (d, *J* = 8.5 Hz, 1H), 7.98 (dd, *J* = 8.5, 2.5 Hz, 1H), 7.56 (d, *J* = 8.2 Hz, 1H), 7.11 (s, 1H), 7.03 (s, 1H), 6.82 (d, *J* = 4.0 Hz, 1H), 4.68 (d, *J* = 5.8

Hz, 2H); ^{13}C NMR (126 MHz, $(CD_3)_2CO$) δ 159.4, 155.6, 153.7, 151.0, 149.4, 149.0 (q, J = 32.9 Hz), 148.5, 137.8, 135.7, 133.2, 132.0, 123.1 (q, J = 273.9 Hz), 122.4, 122.2, 110.1, 105.5, 48.4; MS (ESI+) *m/z* 365 $[M+H]^+$; HRMS (ESI+) *m/z* calcd for $C_{17}H_{13}ClF_3N_4$ $[M+H]^+$: 365.07808 found 365.07603.

N-((6'-Chloro-[3,3'-bipyridin]-6-yl)methyl)-2-(trifluoromethyl)pyridin-4-amine (**11**)

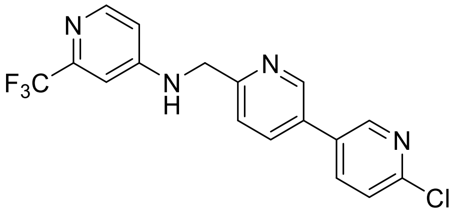

Compound **11** was synthesized according to Method C by reacting compound (**31**) with (6-chloropyridin-3-yl)boronic acid. The crude product was purified by preparative HPLC (Condition A, eluted at 45% ACN, 55% yield). ^1H NMR (500 MHz, $(CD_3)_2CO$) δ 8.92 (d, J = 2.0 Hz, 1H), 8.74 (dd, J = 2.6, 0.5 Hz, 1H), 8.21 (d, J = 5.7 Hz, 1H), 8.18 (dd, J = 8.3, 2.6 Hz, 1H), 8.13 (dd, J = 8.1, 2.4 Hz, 1H), 7.60 – 7.57 (m, 2H), 7.12 (d, J = 2.0 Hz, 1H), 7.03 (s, 1H), 6.82 (dd, J = 5.5, 1.9 Hz, 1H), 4.69 (d, J = 5.8 Hz, 2H); ^{13}C NMR (126 MHz, $(CD_3)_2CO$) δ 158.7, 155.6, 151.5, 151.0, 149.0 (q, J = 32.3 Hz), 148.9, 148.4, 138.5, 136.1, 133.4, 131.7, 125.3, 123.1 (q, J = 273.5 Hz), 122.5, 110.1, 105.4, 48.2; MS (ESI+) *m/z* 365 $[M+H]^+$; HRMS (ESI+) *m/z* calcd for $C_{17}H_{13}ClF_3N_4$ $[M+H]^+$: 365.07808 found 365.07617.

6-Chloro-6'-(((2-(trifluoromethyl)pyridin-4-yl)oxy)methyl)-3,3'-bipyridine (**12**)

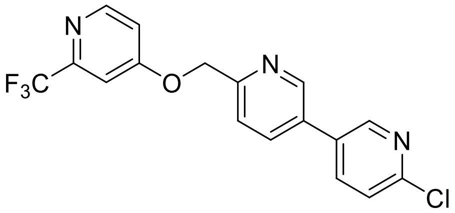

Compound **12** was synthesized according to Method C by reacting compound (**34**) with (6-chloropyridin-3-yl)boronic acid. The crude product was purified by preparative HPLC (Condition B, eluted at 70% ACN, 66% yield). ^1H NMR (500 MHz, $(CD_3)_2CO$) δ 8.97 (d, J = 1.9 Hz, 1H), 8.78 (d, J = 2.4 Hz, 1H), 8.60 (d, J = 5.7 Hz, 1H), 8.24 – 8.20 (m, 2H), 7.76 (d, J = 8.1 Hz, 1H), 7.61 (d, J = 8.3 Hz, 1H), 7.54 (d, J = 2.4 Hz, 1H), 7.36 (dd, J = 5.6, 2.4 Hz, 1H), 5.50 (s, 2H); ^{13}C NMR (126 MHz, $(CD_3)_2CO$) δ 166.7, 156.6, 152.6, 151.7, 150.0 (q, J = 34.0 Hz), 149.0, 148.5, 138.7, 136.3, 133.2, 132.5, 125.4, 122.9, 122.6 (q, J = 273.5 Hz), 113.9, 109.14 (q, J = 2.7 Hz), 71.7; MS (ESI+) *m/z* 366 $[M+H]^+$; HRMS (ESI+) *m/z* calcd for $C_{17}H_{12}ClF_3N_3O$ $[M+H]^+$: 366.06210 found 366.06101.

6-Chloro-6'-(((2-(trifluoromethyl)pyridin-4-yl)thio)methyl)-3,3'-bipyridine (**13**)

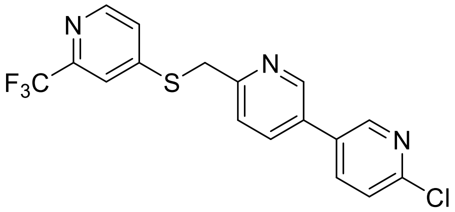

Compound **13** was synthesized according to Method C by reacting compound (**35**) with (6-chloropyridin-3-yl)boronic acid. The crude product was purified by preparative HPLC (Condition A, eluted at 65% ACN, 55% yield). ^{1}H NMR (500 MHz, $(CD_3)_2CO$) δ 8.90 (d, *J* = 2.3 Hz, 1H), 8.75 (d, *J* = 2.6 Hz, 1H), 8.51 (d, *J* = 5.3 Hz, 1H), 8.19 (dd, *J* = 8.3, 2.6 Hz, 1H), 8.15 (dd, *J* = 8.1, 2.4 Hz, 1H), 7.90 (d, *J* = 1.6 Hz, 1H), 7.74 (d, *J* = 8.1 Hz, 1H), 7.69 (dd, *J* = 5.3, 1.7 Hz, 1H), 7.59 (d, *J* = 8.3 Hz, 1H), 4.66 (s, 2H); ^{13}C NMR (126 MHz, $(CD_3)_2CO$) δ 157.7, 152.5, 151.6, 150.4, 148.9, 148.3, 148.2 (q, *J* = 34.0 Hz), 138.6, 136.4, 133.2, 131.9, 125.3, 124.3, 124.2, 122.6 (q, *J* = 273.8 Hz), 118.2 (q, *J* = 2.9 Hz), 37.1; MS (ESI+) *m/z* 382 $[M+H]^+$; HRMS (ESI+) *m/z* calcd for $C_{17}H_{12}ClF_3N_3S$ $[M+H]^+$: 382.03926 found 382.03833.

N-((6'-Methoxy-[3,3'-bipyridin]-6-yl)methyl)-2-(trifluoromethyl)pyridin-4-amine (**14**)

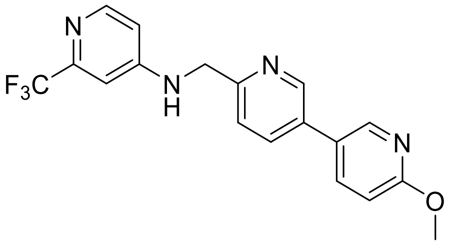

Compound **14** was synthesized according to Method C by reacting compound (**31**) with (6-methoxypyridin-3-yl)boronic acid. The crude product was purified by preparative HPLC (Condition A, eluted at 45% ACN, 62% yield). ^{1}H NMR (500 MHz, $(CD_3)_2CO$) δ 8.84 (d, *J* = 1.8 Hz, 1H), 8.49 (dd, *J* = 2.5, 0.5 Hz, 1H), 8.21 (d, *J* = 5.7 Hz, 1H), 8.02 (ddd, *J* = 8.7, 6.3, 2.5 Hz, 2H), 7.52 (d, *J* = 8.1 Hz, 1H), 7.12 (d, *J* = 1.8 Hz, 1H), 7.01 (s, 1H), 6.89 (dd, *J* = 8.6, 0.7 Hz, 1H), 6.83 (dd, *J* = 5.4, 1.7 Hz, 1H), 4.65 (d, *J* = 5.8 Hz, 2H), 3.94 (s, 3H); ^{13}C NMR (126 MHz, $(CD_3)_2CO$) δ 165.0, 157.4, 155.6, 151.0, 149.0 (q, *J* = 32.6 Hz), 147.9, 146.0, 138.3, 135.5, 133.0, 127.4, 123.1 (d, *J* = 273.4 Hz), 122.4, 111.8, 110.1, 105.4, 53.8, 48.2; MS (ESI+) *m/z* 361 $[M+H]^+$; HRMS (ESI+) *m/z* calcd for $C_{18}H_{16}F_3N_4O$ $[M+H]^+$: 361.12762 found 361.12511.

N-((6'-Chloro-5'-methyl-[3,3'-bipyridin]-6-yl)methyl)-2-(trifluoromethyl)pyridin-4-amine (**15**)

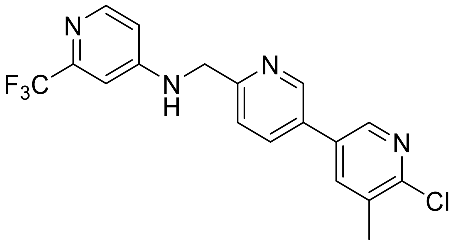

Compound **15** was synthesized according to Method C by reacting compound (**31**) with (6-chloro-5-methylpyridin-3-yl)boronic acid. The crude product was purified by preparative HPLC (Condition A, eluted at 50% ACN, 44% yield). ^{1}H NMR (500 MHz, $(CD_3)_2CO$) δ 8.91 (d, *J* = 2.3 Hz, 1H), 8.57 (d, *J* = 2.4 Hz, 1H), 8.20 (d, *J* = 5.7 Hz, 1H), 8.13 – 8.10 (m, 2H), 7.57 (d, *J* = 8.1 Hz, 1H), 7.11 (s, 1H), 7.05 (s, 1H), 6.82 (d, *J* = 4.1 Hz, 1H), 4.68 (d, *J* = 5.9 Hz, 2H), 2.45 (s, 3H); ^{13}C NMR (126 MHz, $(CD_3)_2CO$) δ 158.6, 155.6, 151.6, 151.0, 148.8 (q, *J* = 34.2 Hz), 148.4, 146.0, 138.8, 136.1, 133.5, 131.8, 123.1 (q, *J* = 273.5 Hz), 122.4, 110.1, 105.3, 48.2, 19.6; MS (ESI+) *m/z* 379 $[M+H]^+$; HRMS (ESI+) *m/z* calcd for $C_{18}H_{15}ClF_3N_4$ $[M+H]^+$: 379.09373 found 379.09155.

N-((6'-Chloro-5'-(trifluoromethyl)-[3,3'-bipyridin]-6-yl)methyl)-2-(trifluoromethyl)pyridin-4-amine (**16**)

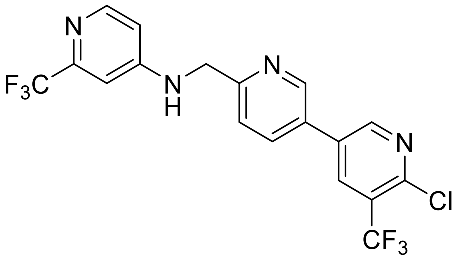

Compound **16** was synthesized according to Method C by reacting compound (**31**) with (6-chloro-5-(trifluoromethyl)pyridin-3-yl)boronic acid. The crude product was purified by preparative HPLC (Condition A, eluted at 60% ACN, 35% yield). ^{1}H NMR (500 MHz, $(CD_3)_2CO$) δ 9.04 – 9.00 (m, 2H), 8.59 (d, *J* = 2.3 Hz, 1H), 8.25 (dd, *J* = 8.2, 2.4 Hz, 1H), 8.21 (d, *J* = 5.7 Hz, 1H), 7.62 (d, *J* = 8.2 Hz, 1H), 7.11 (s, 1H), 7.07 (s, 1H), 6.82 (d, *J* = 4.1 Hz, 1H), 4.70 (d, *J* = 5.9 Hz, 2H); ^{13}C NMR (126 MHz, $(CD_3)_2CO$) δ 159.5, 155.6, 155.5, 151.8, 151.0, 149.0 (q, *J* = 32.9 Hz), 148.7, 148.1, 136.6, 133.8, 130.5, 125.4 (q, *J* = 33.1 Hz), 123.4 (q, *J* = 272.2 Hz), 123.1 (q, *J* = 273.4 Hz), 122.5, 110.1, 105.4, 48.2; MS (ESI+) *m/z* 433 $[M+H]^+$; HRMS (ESI+) *m/z* calcd for $C_{18}H_{12}ClF_6N_4$ $[M+H]^+$: 433.06547 found 433.06429.

N-((5',6'-dichloro-[3,3'-bipyridin]-6-yl)methyl)-2-(trifluoromethyl)pyridin-4-amine (**17**)

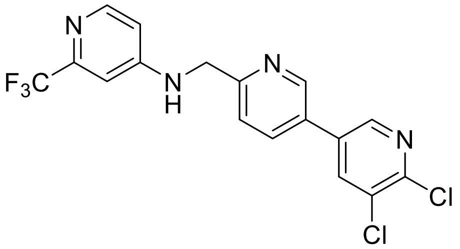

Compound **17** was synthesized according to Method C by reacting compound (**31**) with (5,6-dichloropyridin-3-yl)boronic acid. The crude product was purified by preparative HPLC (Condition A, eluted at 55% ACN, 42% yield. ^{1}H NMR (500 MHz, $(CD_3)_2CO$) δ 8.85 (d, *J* = 1.9 Hz, 1H), 8.60 (d, *J* = 2.2 Hz, 1H), 8.28 (d, *J* = 2.2 Hz, 1H), 8.09 – 8.05 (m, 2H), 7.47 (d, *J* = 8.1 Hz, 1H), 6.99 (s, 1H), 6.95 (s, 1H), 6.69 (d, *J* = 3.9 Hz, 1H), 4.56 (d, *J* = 5.9 Hz, 2H); ^{13}C NMR (126 MHz, $(CD_3)_2CO$) δ 159.3, 159.3, 155.6, 149.0 (q, *J* = 32.8 Hz), 148.6, 148.5, 146.7, 138.2, 136.4, 135.0, 131.0, 130.5, 123.1 (q, *J* = 273.5 Hz), 122.5, 110.2, 105.4, 48.2; MS (ESI+) *m/z* 399 $[M+H]^+$; HRMS (ESI+) *m/z* calcd for $C_{17}H_{12}Cl_2F_3N_4$ $[M+H]^+$: 399.03911 found 399.03794.

N-((5'-Chloro-6'-methoxy-[3,3'-bipyridin]-6-yl)methyl)-2-(trifluoromethyl)pyridin-4-amine (**18**)

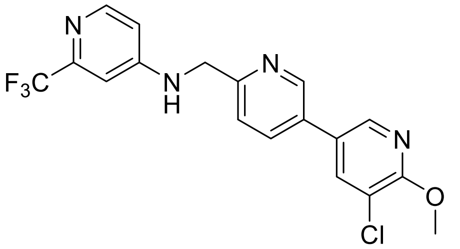

Compound **18** was synthesized according to Method C by reacting compound (**31**) with (5-chloro-6-methoxypyridin-3-yl)boronic acid. The crude product was purified by preparative HPLC (Condition B, eluted at 65% ACN, 59% yield). ^{1}H NMR (300 MHz, $CDCl_3$) δ 8.75 (d, *J* = 1.9 Hz, 1H), 8.31 (d, *J* = 5.7 Hz, 1H), 8.28 (d, *J* = 2.2 Hz, 1H), 7.85 (d, *J* = 2.3 Hz, 1H), 7.85 – 7.80 (m, 1H), 7.38 (d, *J* = 8.1 Hz, 1H), 6.93 (d, *J* = 2.2 Hz, 1H), 6.65 (dd, *J* = 5.6, 2.2 Hz, 1H), 5.95 (s, 1H), 4.55 (d, *J* = 4.8 Hz, 2H), 4.08 (s, 3H); ^{13}C NMR (75 MHz, $CDCl_3$) δ 159.5, 155.0, 153.9, 150.4, 149.0 (q, *J* = 33.7 Hz), 147.2, 142.9, 136.9, 135.2, 131.8, 127.6, 121.96, 121.92 (q, *J* = 274.2 Hz), 119.0, 109.5, 104.9, 54.8, 47.2; MS (ESI+) *m/z* 395 $[M+H]^+$; HRMS (ESI+) *m/z* calcd for $C_{18}H_{15}ClF_3N_4O$ $[M+H]^+$: 395.08865 found 395.08644.

N-((6'-Phenoxy-[3,3'-bipyridin]-6-yl)methyl)-2-(trifluoromethyl)pyridin-4-amine (**19**)

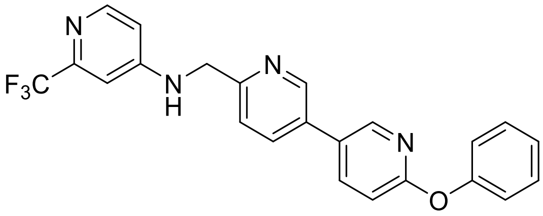

Compound **19** was synthesized according to Method C by reacting compound (**31**) with (6-phenoxypyridin-3-yl)boronic acid. The crude product was purified by preparative HPLC (Condition A, eluted at 60% ACN, 55% yield). ^{1}H NMR (500 MHz, $(CD_3)_2CO$) δ 8.86 (s, 1H), 8.45 (d, J = 2.3 Hz, 1H), 8.20 (d, J = 5.6 Hz, 1H), 8.16 (dd, *J* = 8.5, 2.5 Hz, 1H), 8.05 (dd, *J* = 8.1, 2.1 Hz, 1H), 7.53 (d, *J* = 8.1 Hz, 1H), 7.44 (t, *J* = 7.8 Hz, 2H), 7.23 (t, *J* = 7.4 Hz, 1H), 7.19 (d, *J* = 7.8 Hz, 2H), 7.11 (d, *J* = 8.7 Hz, 2H), 7.03 (s, 1H), 6.82 (d, *J* = 4.1 Hz, 1H), 4.66 (d, *J* = 5.7 Hz, 2H). ^{13}C NMR (126 MHz, $(CD_3)_2CO$) δ 164.5, 157.8, 155.6, 155.3, 151.0, 149.0 (q, *J* = 32.8 Hz), 148.0, 146.5, 139.2, 135.7, 132.6, 130.4, 129.3, 125.4, 123.1 (q, *J* = 273.7 Hz), 122.4, 122.2, 112.5, 110.1, 105.4, 48.3. MS (ESI+) *m/z* 423 $[M+H]^+$; HRMS (ESI+) *m/z* calcd for $C_{23}H_{18}F_3N_4O$ $[M+H]^+$: 423.14327 found 423.14078.

N-((2-(3,4-Dichlorophenyl)pyrimidin-5-yl)methyl)-2-(trifluoromethyl)pyridin-4-amine (**20**)

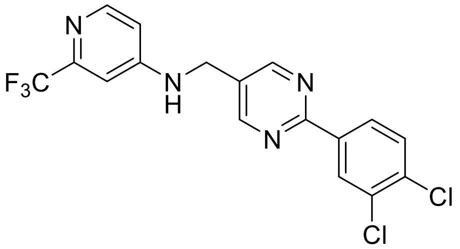

Compound **20** was synthesized according to Method C by reacting compound (**32**) with (3,4-dichlorophenyl)boronic acid. The crude product was purified by preparative HPLC (Condition A, eluted at 70% ACN, 35% yield). ^{1}H NMR (500 MHz, $(CD_3)_2CO$) δ 8.97 (s, 2H), 8.57 (d, *J* = 2.0 Hz, 1H), 8.40 (dd, *J* = 8.5, 2.0 Hz, 1H), 8.23 (d, *J* = 5.7 Hz, 1H), 7.73 (d, *J* = 8.5 Hz, 1H), 7.10 (d, *J* = 2.3 Hz, 1H), 7.00 (s, 1H), 6.85 (dd, *J* = 5.7, 2.3 Hz, 1H), 4.72 (d, *J* = 5.9 Hz, 2H); ^{13}C NMR (126 MHz, $(CD_3)_2CO$) δ 161.9, 157.9, 155.3, 151.2, 149.1 (q, *J* = 32.9 Hz), 138.8, 135.0, 133.1, 131.8, 131.5, 130.4, 128.4, 123.0 (q, *J* = 273.5 Hz), 110.1, 105.44, 42.2; MS (ESI+) *m/z* 399 $[M+H]^+$; HRMS (ESI+) *m/z* calcd for $C_{17}H_{12}Cl_2F_3N_4$ $[M+H]^+$: 399.03911 found 399.03842.

N-((2-(4-Phenoxyphenyl)pyrimidin-5-yl)methyl)-2-(trifluoromethyl)pyridin-4-amine (**21**)

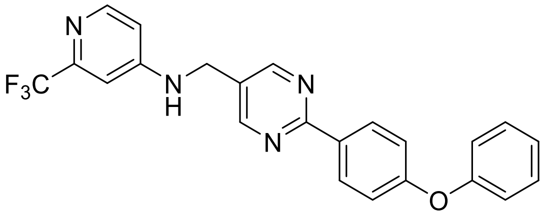

Compound **21** was synthesized according to Method C by reacting compound (**32**) with (4-phenoxyphenyl)boronic acid. The crude product was purified by preparative HPLC (Condition A, eluted at 75% ACN, 68% yield). ^{1}H NMR (500 MHz, $CDCl_3$) δ 8.75 (s, 2H), 8.41 (d, *J* = 8.1 Hz, 2H), 8.33 (d, *J* = 5.6 Hz, 1H), 7.38 (t, *J* = 7.5 Hz, 2H), 7.17 (t, *J* = 7.4 Hz, 1H), 7.08 (d, *J* = 8.1 Hz, 4H), 6.90 (s, 1H), 6.61 (d, *J* = 5.6 Hz, 1H), 4.94 (s, 1H), 4.47 (d, *J* = 5.4 Hz, 2H); ^{13}C NMR (126 MHz, $CDCl_3$) δ 164.3, 160.4, 156.7, 156.4, 153.7, 150.7, 149.2 (q, *J* = 33.9 Hz), 131.8, 130.2, 130.1, 127.4, 124.2, 121.7 (q, *J* = 274.2 Hz), 119.8, 118.3, 109.4, 104.9, 42.6; MS (ESI+) *m/z* 423 $[M+H]^+$; HRMS (ESI+) *m/z* calcd for $C_{23}H_{18}F_3N_4O$ $[M+H]^+$: 423.14327 found 423.14081.

N-((2-(4-Isopropoxyphenyl)pyrimidin-5-yl)methyl)-2-(trifluoromethyl)pyridin-4-amine (**22**)

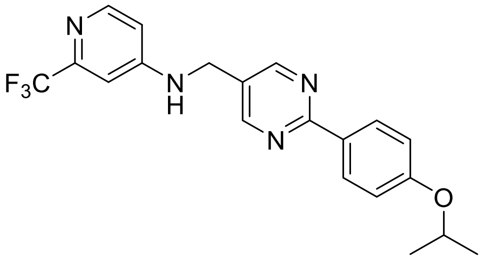

Compound **22** was synthesized according to Method C by reacting compound (**32**) with (4-isopropoxyphenyl)boronic acid. The crude product was purified by preparative HPLC (Condition B, eluted at 70% ACN, 86% yield). ^{1}H NMR (500 MHz, $(CD_3)_2CO$) δ 8.85 (s, 2H), 8.41 – 8.38 (m, 2H), 8.23 (d, J = 5.7 Hz, 1H), 7.09 (d, J = 2.3 Hz, 1H), 7.03 – 7.00 (m, 2H), 6.89 (s, 1H), 6.85 (dd, J = 5.7, 2.3 Hz, 1H), 4.73 (hept, J = 6.0 Hz, 1H), 4.63 (d, J = 5.9 Hz, 2H), 1.34 (d, J = 6.0 Hz, 6H); ^{13}C NMR (126 MHz, $CDCl_3$) δ 164.6, 160.7, 156.6, 153.7, 150.7, 149.2 (q, *J* = 33.8 Hz), 130.0, 129.4, 126.9, 121.8 (q, *J* = 274.3 Hz), 115.8, 109.4, 104.9, 70.1, 42.6, 22.1; MS (ESI+) *m/z* 389 $[M+H]^+$; HRMS (ESI+) *m/z* calcd for $C_{20}H_{20}F_3N_4O$ $[M+H]^+$: 389.15892 found 389.15650.

N-((2-(4-Cyclopropylphenyl)pyrimidin-5-yl)methyl)-2-(trifluoromethyl)pyridin-4-amine (**23**)

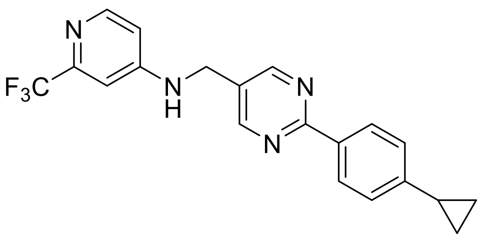

Compound **23** was synthesized according to Method C by reacting compound (**32**) with (4-cyclopropylphenyl)boronic acid. The crude product was purified by preparative HPLC (Condition B, eluted at 70% ACN, 81% yield). ^{1}H NMR (500 MHz, $CDCl_3$) δ 8.73 (s, 2H), 8.32 (t, *J* = 6.3 Hz, 3H), 7.17 (d, *J* = 8.0 Hz, 2H), 6.89 (s, 1H), 6.60 (d, *J* = 5.6 Hz, 1H), 4.89 (s, 1H), 4.44 (d, *J* = 5.5 Hz, 2H), 1.99 – 1.93 (m, 1H), 1.06 – 1.02 (m, 2H), 0.80 – 0.77 (m, 2H); ^{13}C NMR (126 MHz, $CDCl_3$) δ 164.8, 156.6, 153.6, 150.7, 149.2 (q, *J* = 33.7 Hz), 148.0, 134.3, 128.3, 127.4, 125.9, 121.7 (q, *J* = 274.5 Hz), 109.4, 104.9, 42.6, 15.7, 10.2; MS (ESI+) *m/z* 371 $[M+H]^+$; HRMS (ESI+) *m/z* calcd for $C_{20}H_{18}F_3N_4$ $[M+H]^+$: 371.14836 found 371.14602.

N-((2-(3-Chloro-4-isopropoxyphenyl)pyrimidin-5-yl)methyl)-2-(trifluoromethyl)pyridin-4-amine (**24**)

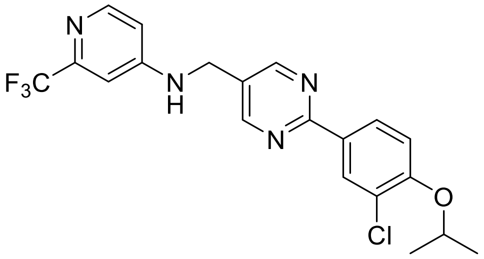

Compound **24** was synthesized according to Method C by reacting compound (**32**) with (3-chloro-4-isopropoxyphenyl)boronic acid. The crude product was purified by preparative HPLC (Condition A, eluted at 70% ACN, 68% yield). ^{1}H NMR (500 MHz, $(CD_3)_2CO$) δ 8.88 (s, 2H), 8.46 (d, *J* = 2.1 Hz, 1H), 8.36 (dd, *J* = 8.7, 2.2 Hz, 1H), 8.23 (d, *J* = 5.7 Hz, 1H), 7.25 (d, *J* = 8.8 Hz, 1H), 7.09 (d, *J* = 2.3 Hz, 1H), 6.90 (s, 1H), 6.85 (dd, *J* = 5.7, 2.3 Hz, 1H), 4.81 (hept, *J* = 6.1 Hz, 1H), 4.66 (d, *J* = 5.9 Hz, 2H), 1.39 (d, *J* = 6.0 Hz, 6H); ^{13}C NMR (126 MHz, $(CD_3)_2CO$) δ 162.9, 157.8, 156.6, 155.4, 151.2, 149.1 (q, *J* = 33.0 Hz), 131.6, 130.5, 130.2, 128.7, 124.2, 123.1 (q, *J* = 273.4 Hz), 115.6, 110.1, 105.4, 72.4, 42.3, 22.2; MS (ESI+) *m/z* 423 $[M+H]^+$; HRMS (ESI+) *m/z* calcd for $C_{20}H_{19}ClF_3N_4O$ $[M+H]^+$: 423.11995 found 423.11898.

N-((2-(5-Chloro-6-isopropoxypyridin-3-yl)pyrimidin-5-yl)methyl)-2-(trifluoromethyl)pyridine-4-amine (**25**)

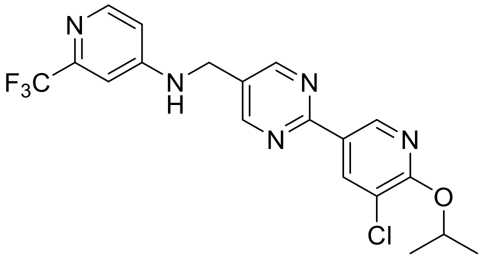

Compound **25** was synthesized according to Method C by reacting compound (**32**) with (5-chloro-6-isopropoxypyridin-3-yl)boronic acid. The crude product was purified by preparative HPLC (Condition B, eluted at 75% ACN, 63% yield). ^{1}H NMR (500 MHz, $(CD_3)_2CO$) δ 9.08 (d, *J* = 2.1 Hz, 1H), 8.92 (s, 2H), 8.64 (d, *J* = 2.1 Hz, 1H), 8.23 (d, *J* = 5.7 Hz, 1H), 7.10 (d, *J* = 2.3 Hz, 1H), 6.94 (s, 1H), 6.86 (dd, *J* = 5.7, 2.3 Hz, 1H), 5.47 (hept, *J* = 6.2 Hz, 1H), 4.69 (d, *J* = 5.9 Hz, 2H), 1.40 (d, *J* = 6.2 Hz, 6H); ^{13}C NMR (126 MHz, $(CD_3)_2CO$) δ 161.7, 160.9, 157.9, 155.4, 151.2, 149.1 (q, *J* = 33.1 Hz), 146.1, 138.2, 130.9, 128.5, 123.1 (q, *J* = 273.5 Hz), 118.8, 110.1, 105.5, 71.0, 42.3, 22.2; MS (ESI+) *m/z* 424 $[M+H]^+$; HRMS (ESI+) *m/z* calcd for $C_{19}H_{18}ClF_3N_5O$ $[M+H]^+$: 424.11520 found 424.11437.

Ditert-butyl(2-(trifluoromethyl)pyridin-4-yl)dicarbamate (**26**)

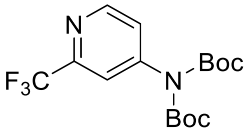

To a solution of 2-trifluoromethyl-4-aminopyridine (0.81 g, 5 mmol) in THF (30 mL) were added Boc_2O (3.27 g, 15 mmol), triethylamine (1.75 mL, 12.5 mmol) and DMAP (122 mg, 1 mmol). The reaction mixture was stirred for 18 h at r.t. under argon atmosphere. Excess solvent was evaporated under reduced pressure and to the remaining residue, 1 N HCl (25 mL) was added followed by extraction with ethyl acetate. The organic solvent was then dried over $MgSO_4$ and evaporated under reduced pressure. The crude product was used without any further purification.

^{1}H NMR (300 MHz, $CDCl_3$): δ [ppm] = 8.72 (d, *J* = 5.4 Hz, 1 H), 7.52 (d, *J* = 1.7 Hz, 1 H), 7.31 (dd, *J* = 5.3 Hz, *J* = 1.8 Hz, 1 H), 1.47 (s, 18 H); MS (ESI+) *m/z* 363 $[M+H]^+$.

Tert-butyl(2-(trifluoromethyl)pyridin-4-yl)carbamate (**27**)

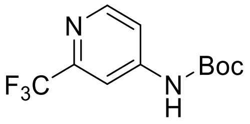

To compound **26** (1.7 g, 4.7 mmol) dissolved in DCM (20 mL) was added TFA (2 mL). The reaction was stirred for 30 min at room temp until the starting material was fully consumed. Saturated $NaHCO_3$ was added followed by extraction with DCM. The organic solvent was then dried over $MgSO_4$ and evaporated under reduced pressure. Purification was done using automated column chromatography.

Yield 91%; ^{1}H NMR (300 MHz, $CDCl_3$): δ [ppm] = 8.53 (d, *J* = 5.6 Hz, 1 H), 7.78 (d, *J* = 2.0 Hz, 1 H), 7.44 (dd, *J* = 5.5 Hz, *J* = 2.1 Hz, 1 H), 6.92 (bs, 1 H), 1.53 (s, 9 H); MS (ESI+) *m/z* 263 [*M*+H]$^+$.

Compounds **28a–28f** are commercially available.

Synthesis of compounds **28g** and **28h** was done according to the following scheme.

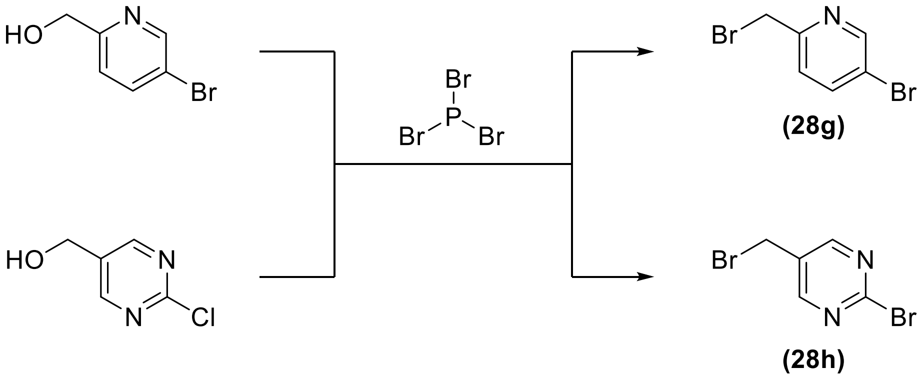

5-Bromo-2-(bromomethyl)pyridine (**28g**)

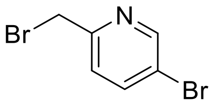

To a stirred solution of (5-bromopyridin-2-yl)methanol (350 mg, 1.86 mmol) in DCM (20 mL) at 0 °C, was added phosphorus tribromide (2.519 g, 9.30 mmol). The reaction was stirred at room temp. for 18 h. The reaction was then quenched by dropwise addition of $NaHCO_3$ at 0 °C followed by extraction with DCM. The organic solvent was then dried over $MgSO_4$, filtered and concentrated under reduced pressure. Purification was done using automated column chromatography to yield compound (**28g**).

Yield 77%; MS (ESI+) *m/z* 252 [*M*+H]$^+$

2-Bromo-5-(bromomethyl)pyrimidine (**28h**)

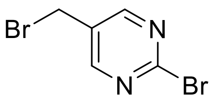

To a stirred solution of (2-chloropyrimidin-5-yl)methanol (1 g, 6.91 mmol) in DCM (20 mL) at 0 °C was added the Phosphorus tribromide (9.37 g, 34.58 mmol). The reaction was stirred at room temp. for 18 h. The reaction was then quenched by dropwise addition of $NaHCO_3$ at 0 °C followed by extraction with DCM. The organic solvent was then dried over $MgSO_4$ and concentrated under reduced pressure. Purification was done using automated column chromatography to yield compound (**28h**). Yield 49%; MS (ESI+) *m/z* 253 [*M*+H]$^+$.

Tert-butyl (4-fluorobenzyl)(2-(trifluoromethyl)pyridin-4-yl)carbamate (**29a**)

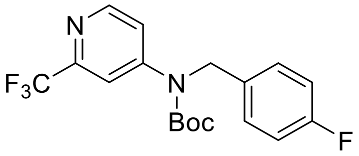

Compound **29a** was synthesized according to Method A by reacting compound (**27**) with 1-(bromomethyl)-4-fluorobenzene (**28a**). The crude product was used without any further purification. Yield 92%; MS (ESI+) *m/z* 371 [*M*+H]$^+$.

Tert-butyl (4-chlorobenzyl)(2-(trifluoromethyl)pyridin-4-yl)carbamate (**29b**)

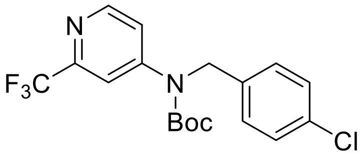

Compound **29b** was synthesized according to Method A by reacting compound (**27**) with 1-(bromomethyl)-4-chlorobenzene (**28b**). The crude product was used without any further purification. Yield 75%; MS (ESI+) *m/z* 387 [*M*+H]$^+$.

Tert-butyl (4-bromobenzyl)(2-(trifluoromethyl)pyridin-4-yl)carbamate (**29c**)

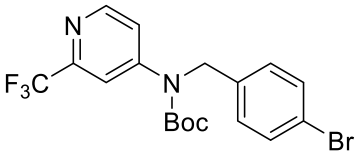

Compound **29c** was synthesized according to Method A by reacting compound (**27**) with 1-bromo-4-(bromomethyl)benzene (**28c**). The crude product was used without any further purification. Yield 79%; MS (ESI+) *m/z* 431 [*M*+H]$^+$.

Tert-butyl (3-bromobenzyl)(2-(trifluoromethyl)pyridin-4-yl)carbamate (**29d**)

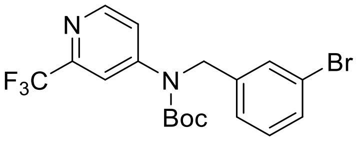

Compound **29d** was synthesized according to Method A by reacting compound (**27**) with 1-bromo-3-(bromomethyl)benzene (**28d**). The crude product was used without any further purification. Yield 70%; MS (ESI+) *m/z* 431 $[M+H]^+$.

Tert-butyl (2-bromobenzyl)(2-(trifluoromethyl)pyridin-4-yl)carbamate (**29e**)

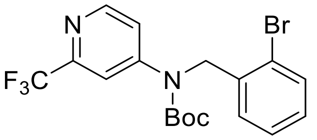

Compound **29e** was synthesized according to Method A by reacting compound (**27**) with 1-bromo-2-(bromomethyl)benzene (**28e**). The crude product was used without any further purification. Yield 91%; MS (ESI+) *m/z* 431 $[M+H]^+$.

Tert-butyl ((6-bromopyridin-3-yl)methyl)(2-(trifluoromethyl)pyridin-4-yl)carbamate (**29f**)

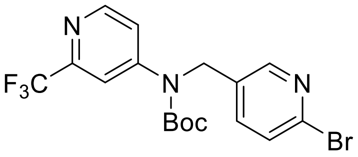

Compound **29f** was synthesized according to Method A by reacting compound (**27**) with 2-bromo-5-(bromomethyl)pyridine (**28f**). Purification was done using automated column chromatography and used directly in the next step. Yield 73%; MS (ESI+) *m/z* 432 $[M+H]^+$.

Tert-butyl ((5-bromopyridin-2-yl)methyl)(2-(trifluoromethyl)pyridin-4-yl)carbamate (**29g**)

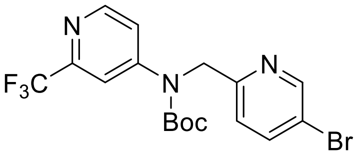

Compound **29g** was synthesized according to Method A by reacting compound (**27**) with (**28g**). Purification was done using automated column chromatography and used directly in the next step. Yield 90%; MS (ESI+) *m/z* 432 $[M+H]^+$.

Tert-butyl ((2-bromopyrimidin-5-yl)methyl)(2-(trifluoromethyl)pyridin-4-yl)carbamate (**29h**)

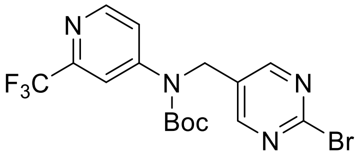

Compound **29h** was synthesized according to Method A by reacting compound (**27**) with (**28h**). Purification was done using automated column chromatography and used directly in the next step. Yield 82%; MS (ESI+) *m/z* 433 $[M+H]^+$.

N-((6-Bromopyridin-3-yl)methyl)-2-(trifluoromethyl)pyridin-4-amine (**30**)

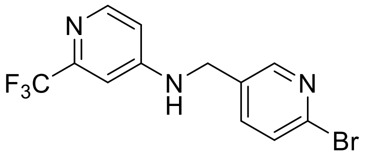

Compound **30** was synthesized from compound (**29f**) according to the general method B. Purification was done using automated column chromatography. Yield 91%; ^{1}H NMR (500 MHz, $(CD_3)_2CO$) δ 8.46 (d, *J* = 2.2 Hz, 1H), 8.32 (d, *J* = 6.1 Hz, 1H), 7.79 (dd, *J* = 8.2, 2.5 Hz, 1H), 7.60 (d, *J* = 8.2 Hz, 1H), 7.52 (s, 1H), 7.20 (d, *J* = 2.3 Hz, 1H), 6.94 (dd, *J* = 6.1, 2.3 Hz, 1H), 4.69 (s, 2H). ^{13}C NMR (126 MHz, $(CD_3)_2CO$) δ 156.7, 150.5, 148.9, 146.5 (q, *J* = 34.1 Hz), 141.6, 139.2, 134.2, 128.9, 122.4 (q, *J* = 273.7 Hz) 110.0, 106.2, 43.9; MS (ESI+) *m/z* 332 $[M+H]^+$.

N-((5-Bromopyridin-2-yl)methyl)-2-(trifluoromethyl)pyridin-4-amine (**31**)

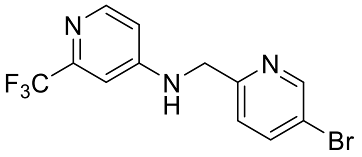

Compound **31** was synthesized from compound (**29g**) according to the general method B. Purification was done using automated column chromatography. Yield 97%; ^{1}H NMR (500 MHz, $(CD_3)_2CO$) δ 8.65 (d, *J* = 2.3 Hz, 1H), 8.19 (d, *J* = 5.8 Hz, 1H), 7.97 (dd, *J* = 8.4, 2.4 Hz, 1H), 7.42 (d, *J* = 8.4 Hz, 1H), 7.07 (s, 1H), 7.01 (s, 1H), 6.77 (d, J = 3.7 Hz, 1H), 4.59 (d, *J* = 5.9 Hz, 2H). ^{13}C NMR (126 MHz, $(CD_3)_2CO$) δ 157.7, 155.5, 151.0, 150.9, 148.9 (q, *J* = 31.0 Hz), 140.3, 124.0, 123.1 (q, *J* = 273.4 Hz), 119.7, 110.1, 105.4, 48.0; MS (ESI+) *m/z* 332 $[M+H]^+$.

N-((2-Bromopyrimidin-5-yl)methyl)-2-(trifluoromethyl)pyridin-4-amine (**32**)

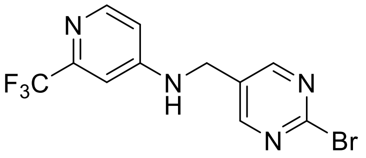

Compound **32** was synthesized from compound (**29h**) according to the general method B. Purification was done using automated column chromatography. Yield 80%; ^{1}H NMR (500 MHz, $(CD_3)_2CO$) δ 8.73 (s, 2H), 8.22 (d, *J* = 5.7 Hz, 1H), 7.08 (d, *J* = 2.2 Hz, 1H), 6.96 (s, 1H), 6.83 (dd, *J* = 5.6, 2.1 Hz, 1H), 4.67 (d, *J* = 6.0 Hz, 2H). ^{13}C NMR (126 MHz, $(CD_3)_2CO$) δ 160.1, 155.3, 152.4, 151.1, 149.0 (q, *J* = 33.0 Hz), 132.5, 123.0 (q, *J* = 273.5 Hz), 110.1, 105.5, 41.7; MS (ESI+) *m/z* 333 $[M+H]^+$.

2-(Trifluoromethyl)pyridine-4-thiol (**33**)

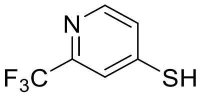

A mixture of 2-(trifluoromethyl)pyridin-4-ol (163 mg, 1 mmol) and Lawesson's reagent (202 mg, 0.5 mmol) in toluene (10 mL) was refluxed for 2 h. Excess solvent was evaporated under reduced pressure and the remaining residue was purified using automated column chromatography. Yield 55%; MS (ESI+) *m/z*) 180 $[M+H]^+$.

4-((5-Bromopyridin-2-yl)methoxy)-2-(trifluoromethyl)pyridine (34)

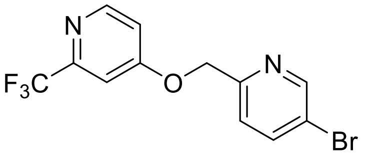

A mixture of 2-(trifluoromethyl)pyridin-4-ol (50 mg, 0.31 mmol) and K_2CO_3 (50.86 mg, 0.39 mmol) in acetone (5 mL) was stirred at room temperature for 30 min. Compound (**28g**) (92.35 mg, 0.39 mmol) was then added and the reaction was heated at 60 °C for 1 h. The reaction was then added to iced water followed by extraction with ethyl acetate. The organic solvent was then dried over $MgSO_4$, filtered and evaporated under reduced pressure. Purification was done using automated column chromatography. Yield 56%; ^{1}H NMR (500 MHz, $(CD_3)_2CO$) δ 8.70 (d, J = 2.3 Hz, 1H), 8.58 (d, J = 5.7 Hz, 1H), 8.07 (dd, J = 8.4, 2.4 Hz, 1H), 7.61 (d, J = 8.4 Hz, 1H), 7.50 (d, J = 2.4 Hz, 1H), 7.32 (dd, J = 5.7, 2.4 Hz, 1H), 5.41 (s, 2H). ^{13}C NMR (126 MHz, $(CD_3)_2CO$) δ 166.6, 155.5, 152.6, 151.1, 150.1 (q, J = 34.1 Hz), 140.5, 124.5, 122.6 (q, J = 273.5 Hz), 120.6, 113.9, 109.1 (q, J = 2.7 Hz), 71.3; MS (ESI+) *m/z* 333 $[M+H]^+$.

4-(((5-Bromopyridin-2-yl)methyl)thio)-2-(trifluoromethyl)pyridine (**35**)

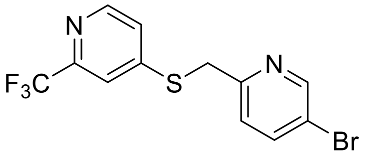

To compound (**33**) (89.6 mg, 0.5 mmol) and K_2CO_3 (83 mg, 0.6 mmol) in acetone (10 mL) was added compound (**28g**) (150.6 mg, 0.6 mmol). The reaction was stirred for 2 h at room temp. Excess solvent was evaporated under reduced pressure and to the remaining residue, water (20 mL) was added followed by extraction with ethyl acetate. The organic solvent was then dried over $MgSO_4$, filtered and evaporated under reduced pressure. The crude product was purified by preparative HPLC (Condition A, eluted at 60% ACN, 65% yield). ^{1}H NMR (500 MHz, $(CD_3)_2CO$) δ 8.63 (d, *J* = 2.3 Hz, 1H), 8.50 (d, *J* = 5.3 Hz, 1H), 8.00 (dd, *J* = 8.4, 2.4 Hz, 1H), 7.84 (d, *J* = 1.6 Hz, 1H), 7.63 (dd, *J* = 5.3, 1.6 Hz, 1H), 7.59 (d, *J* = 8.4 Hz, 1H), 4.58 (s, 2H). ^{13}C NMR (126 MHz, $(CD_3)_2CO$) δ 156.6, 152.3, 150.9, 150.4, 148.2 (q, *J* = 34.0 Hz), 140.6, 125.8, 124.2, 122.6 (q, *J* = 273.8 Hz), 120.0, 118.2 (q, *J* = 2.8 Hz), 36.7; MS (ESI+) *m/z* 349 $[M+H]^+$.

1.2 BIOLOGY

1.2.1 **PqsR reporter gene assay in *Escherichia coli***

The agonistic and antagonistic activity of PqsR ligands was evaluated *via* β-galactosidase reporter gene assay utilizing ortho-Nitrophenyl-β-galactoside (ONPG). As previously described,[1] *Escherichia coli* Dh5α containing plasmid pEAL08-2 expressing *pqsR* under *tac* promoter and *lacZ* (encoding β-galactosidase LacZ) under *pqsA* promoter was incubated with the test compounds in presence of 50 nM PQS. IC_{50} values were determined from dose-response curves of compounds using at least six different concentrations and fitting with non-linear regression analysis (OriginPro 2017–2020, Originlab Corporation).

1.2.2 **Pyocyanin assay in *Pseudomonas aeruginosa***

Inhibition of pyocyanin biosynthesis of compounds was measured spectrophotometrically as previously described.[2] *Pseudomonas aeruginosa* PA14 was grown in rich medium for 16 h under agitation with and without compound. Pyocyanin was extracted from cultures with chloroform, then re-extracted with 0.2 M HCl and quantified photometrically (520 nm). IC_{50} values were determined from dose-response curves of compounds using at least six different concentrations and fitting with non-linear regression analysis (OriginPro 2017–2020, Originlab Corporation).

1.2.3 **Alkylquinolone quantification**

Inhibition of alkylquinolone (AQ) signal molecule biosynthesis of compound 24 was analyzed *via* LC-ESI-MS/MS. Extracellular concentrations of the AQs PQS (2-heptyl-3-hydroxy-4-quinolone), HHQ (2-heptyl-4-quinolone), HQNO (N-oxo-2-heptyl-4-Hydroxyquinoline), 2-AA (2-aminoacetophenone) were measured from supernatants of cultures incubated with or without compound for 17 h. 5,6,7,8-Tetradeutero-2-heptyl-4(1H)-quinolone (HHQ-d4) was added as internal standard before sample preparation. IC_{50} values were calculated according to the procedure of the pyocyanin assay (see above). LC-ESI-MS/MS conditions were as follows: Dionex Ultimate 3000 HPLC instrument parameters: Zorbax Eclipse XDB 80 Å C18 5 µm 4.6 x 50 mm (Agilent, Santa Clara, CA, USA); eluent A – H2O with 0.1% trifluoroacetic acid (TFA), 0.1% pentafluoropropionic acid (PFPA), 0.1% heptafluorobutyric acid (HFBA); eluent B – acetonitrile with 0.1% TFA, 0.1% PFPA, 0.1% HFBA; isocratic 50% A; flow – 0.7 mL/min. TSQ Quantum Access Max (Thermo Fisher Scientific, Waltham,

MA, USA) instrument parameters: Spray voltage 3500 V, vaporizer temperature 370 °C, sheath gas pressure 35 psi, aux gas pressure 30 psi, capillary temperature 270 °C, collision pressure 1.5 mTorr, positive ionization mode. For AQ analyte reaction monitoring parameters please see Table S1.

Table S1. LC-ESI-MS/MS reaction monitoring parameters of AQ analytes in positive ionization mode.

	**precursor ion m/z**	**product ion m/z**	**collision energy (V)**	**tube lens offset (V)**	**retention time (min)**
**PQS**	**260.048**	**145.958**	**44**	**110**	**2.4**
		**174.927**	**30**	**110**	
**HHQ**	**244.050**	**158.944**	**31**	**100**	**2.3**
		**171.943**	**33**	**100**	
**HQNO**	**260.036**	**158.908**	**28**	**110**	**3.0**
**2-AA**	**136.016**	**91.048**	**24**	**68**	**1.5**
		**117.998**	**13**	**68**	
**d4HHQ**	**248.081**	**162.965**	**32**	**100**	**2.3**
		**175.982**	**34**	**100**	

1.2.4 **Biofilm-formation assay**

Biofilm formation was determined using a MBEC Assay® Biofilm Inoculator with 96-well base (Innvotech, Edmonton, CA). An overnight culture of PA14 was centrifuged (10 min, 5000 x g). The supernatant including extracellular polymers was removed and the pellet washed with 10 mL of LB medium. Centrifugation was repeated, the supernatant discarded, and the pellet resuspended in 5 mL of LB. OD was adjusted to 0.2. The bacteria were further diluted in LB medium, followed by addition of the target compound or DMSO control to give OD 0.1 and 1% DMSO. 150 µL were added to each well of the 96-well plate. The outer wells were used as blanks containing 150 µL of 1% DMSO in LB. Six replicates per sample were distributed evenly across the plate, with every row containing one replicate of each sample. The well plate was covered with the peg lid. One layer of parafilm was wrapped around the biofilm inoculator. The inoculator was incubated at 37 °C, static for 24 h with a beaker of H_2O ensuring humidity. Alternatively, the plate was incubated using only LB for 8 h prior to addition of DMSO/**24** to give 1% DMSO, followed by another 16 h incubation under the same conditions.

After 24 h, the plate was removed from the incubator and the peg lid was transferred to a fresh 96 well plate (Nunc U bottom, Thermo Fisher, Dreieich, Germany) containing the respective target

compound, 150 µL 1% DMSO in LB. The inoculator was wrapped with parafilm again and incubated for another 24 h. After that, the peg lid was transferred for 1 min to a 96-well plate containing 200 µL sterile MΩ in each well, followed by another 96 well plate containing 200 µL LB in each well. The system was sealed with parafilm and sonicated for 15 min in an ultrasonic bath with tray. Immediately after sonication, 20 µL were removed from each well using a multi-channel pipette and diluted 1:10 (stepwise, until 10-6). After each dilution step, 10 µL were directly transferred to cetrimide agar plates (spot plating) using fresh pipette tips. Agar plates were incubated for 16 h at 37 °C and then used for cell counting. Statistical analysis was performed with GraphPad Prism 8.4.1 (GraphPad Software, San Diego, CA) using one-way ANOVA with Tukey's multiple comparisons test. Statistical outliers were determined using the Nalimov test, applying a 95% cut-off.
Results are shown in Figure S1.

1.2.5 **Biofilm-eradication assay**

The assay was carried out as described in section 1.2.4, with the following modifications: In the first incubation step only QSI or DMSO (DMSO control and tobramycin control) were added to the wells at time 0. The well plate used for the second incubation step contained QSI/DMSO as before, in presence/absence of tobramycin/amikacin. Aminoglycoside antibiotics were diluted from a 10x stock solution in LB medium to a final concentration of 0.5 µg/mL (TOB) or 4 µg/mL (AMK). In the assays with amikacin, *P. aeruginosa* PAO1 was used.

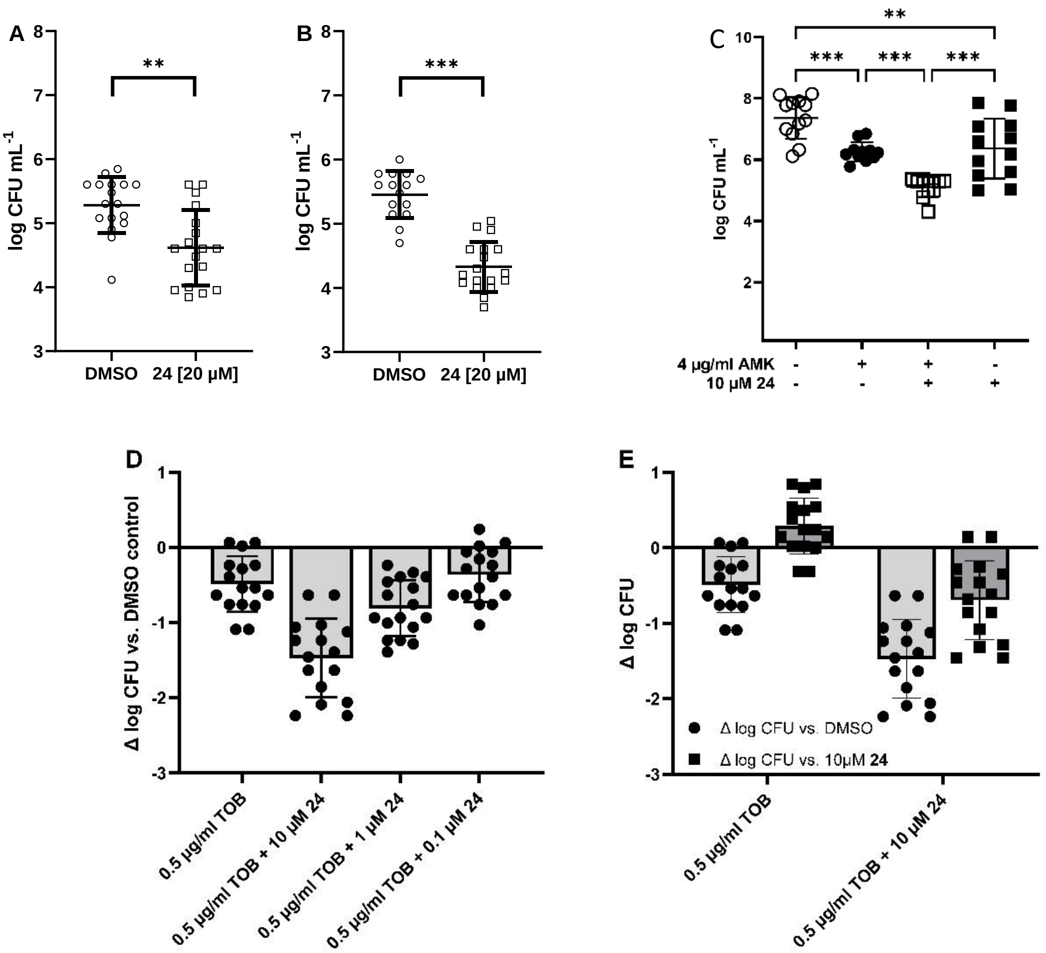

Figure S1. PA14 biofilm assay in the Calgary Biofilm Device. **(A)** Cp. **24**/DMSO were added at the beginning of the assay or **(B)** to previously untreated cultures after 8 h. Viable cells were determined after 48 *via* CFU count. **(C)** Combination of 10 µM **24** with 4 µg/mL amikacin (AMK) leads to a significant boost in activity against *P. aeruginosa* PAO1 compared to treatment with **24**/antibiotic individually. Means and SD of three independent measurements are depicted ((** = $p < 0.01$, *** = $p < 0.001$ according to Tukey's range test). **(D & E)** Data from PA14 biofilm assay in the Calgary Biofilm Device as shown in Figure 4C from the main manuscript depicted using Δ log CFU values calculated with regard to DMSO control **(D)** as well as with regard to DMSO control and 10 µM of compound 24 alone **(E)**. Biofilms were grown for 24 h in presence of **24**/DMSO, followed by treatment of indicated samples with TOB for another 24 h. Viable cells were determined after 48 h via CFU count. Means and SD of three independent measurements are depicted.

1.2.6 eDNA assay

The eDNA release assay was performed as described previously[3] with slight modifications. The impact on eDNA was assessed by incubation of biofilm with propidium iodine solution (0.025 mg mL^{-1}) for 3 h and detection of specific florescence at 620 nm after a thorough washing step with 18MΩH_2O.

1.2.7 *P. aeruginosa* clinical isolates from bronchiectasis patients – phenotypic characterization and inhibition of pyocyanin production

Clinical isolates. The *P. aeruginosa* strains had been isolated from respiratory secretions collected from patients with bronchiectasis seen at the bronchiectasis out-patient clinic of Hannover Medical School. The clinical isolates were genotyped by a custom-made multilocus microarray.[4] The hexadecimal SNP genotype was converted into the multilocus sequence genotype (MLST)[5] by comparison with the sequence of the seven selected housekeeping genes in genome sequences of the same clone deposited in our in-house[6] or the International Pseudomonas Genome Database.[7]

Table S2. Multimarker array genotype and MLST sequence type of *P. aeruginosa* clinical isolates from bronchiectasis patients

**Strain**	**Array-Code**	**MLST**
Bron 8	3C2A	ST179
Bron 11	B421	*
Bron 12	B421	*
Bron 19	F46D	ST235
Bron 25	EA0A	ST27
Bron 27	0C2E	ST395
Bron 28	2F82	ST245
Bron 36	239A	ST1320
Bron 37	2C22	ST274
Bron 39	F429	ST2770
Bron 40	C40A	ST17
Bron 46	D421	ST253
Bron 59	C40A	ST17
Bron 66	B421	*
Bron 76	B421	*

Bron 11 and Bron 12: clonal variants of the same sputum sample collected from subject A

Bron 66 and Bron 76: clonal variants of the same sputum sample collected from subject B

Bron 40 and Bron 59: clonal variants collected from different subjects at separate dates four months apart

* no assigment to MLST scheme feasible because of the 8 - 10 % sequence diversity to the reference genome

(common intraclonal sequence diversity 0.5 %)

C40A, ST17, clone C; clade 1; most abundant clone in the *P. aeruginosa* population

D421, ST253, clone PA14; clade 2; second most abundant clone in the *P. aeruginosa* population

F46D, ST235, clade 2; ST235; most abundant clone in acute infections and in ICUs

B421, most common clone in the inanimate environment; clade 3; T3SS negative

Biofilm formation assay. Biofilm quantification was conducted using the crystal violet staining procedure as described before.[8] Briefly, overnight grown cultures of each clinical *P. aeruginosa* isolate (CI) were diluted 1:100 in LB medium, and 100 µL of the dilutions were inoculated for 48 h at 37°C in a 96-well microtiter plate in a humid chamber. After incubation, the culture medium from each well was carefully discarded and the plate was washed twice using ddH_20. Biofilm staining was carried out by the addition of 125 µL of 0.1 % crystal violet to each well, followed by 10 min incubation at room temperature. The crystal violet solution was discarded and the plate was twice with ddH_20. After the plate was completely dry, the remaining crystal violet from each well attached to the grown biofilm was dissolved in 150 µL of 30 % acetic acid. Finally, 125 µL of the crystal violet solution was transferred into a new 96-well microtiter plate and absorbance at 550 nm was measured in a Tecan Infinite® M Nano reader (Männedorf, Switzerland). The experiments were carried out at least in triplicates.

Swimming motility. The swimming assay was carried out in square petri dishes containing 50 mL of BM2 medium (40 mM K_2HPO_4; 22 mM KH_2PO_4; 7 mM $(NH_4)_2SO_4$) supplemented with 0.4% Glucose, 2 mM $MgSO_4$, 10 µM $FeSO_4$, 0.1% casamino acids and 0.3% Bacto agar. For this purpose, LB-grown overnight cultures of each strain were diluted 1:30 in 5 ml of pre-warmed LB-medium and grown for 6 h at 37°C with constant shaking. An aliquot of each culture was adjusted in its cell density to obtain 0.5 mL of cells with an OD_{600} of 1.5. Next, a sterile toothpick was soaked into the tube containing the diluted cells and immediately used to stab the agar plate. The petri dishes were incubated for at least 16 h at 37°C in a humid chamber and the diameter of the swimming halo was measured with a ruler. The laboratory strains PA14 and PAO1 were always assayed as control. The motility assays were run in triplicates.

Swarming motility. To determine the swarming motility of the cultures, a protocol similar to the swimming assay (*vide supra*) was carried out with the following modifications. The BM2 medium was slightly modified by removing the $(NH_4)_2SO_4$ from the stock solution and adjusting the agar concentration to 0.5%. The agar petri dishes were inoculated with 2 µL of diluted cells (OD_{600} of 1.5) prepared as indicated above, and were incubated for at least 16 h at 30°C in a humid chamber. Finally, the plates were photographed and the swarming area for each strain was determined using ImageJ Fiji.[9] Once again, the PA14 and PAO1 strains were assayed as control. The motility assays were run in triplicates.

Pyoverdine determinations. Precultures of each strain were grown 20 h at 37°C in M9 minimal medium (33.7 mM $Na_2HPO_4•2H_2O$; 22 mM KH_2PO_4; 8.55 mM NaCl; 9.35 mM NH_4Cl) supplemented with 30 mM succinate, 2 mM $MgSO_4$, 10 µM $FeSO_4$ and 2.5 mL L^{-1} of trace elements solution.[10] Next, 5 µl of each culture were transferred to 10 ml of M9 minimal medium devoid of iron and trace element solution, and cultured again for 24 h at 37°C with constant shaking. After incubation, the OD_{600} of the cultures was determined: The cell contained in 1 mL medium were harvested by centrifugation (5 min; 14,000 rpm) and the absorbance at 405 nm (A_{405nm}) of 0.8 ml of the supernatant was determined spectrophotometrically and normalized to the OD_{600} of the culture measured before (i.e. A_{405nm}, OD_{600}^{-1}). PA14 and PAO1 were also assayed as controls. The experiments were carried out at least in triplicates.

Pyocyanin inhibition assay. The inhibition of pyocyanin production in the clinical isolates (CIs) and the type strains (PA14 and PAO1) was tested by the treatment of LB-grown cultures incubated for 16 h at 37°C with constant shaking with 2 µM of **24**/DMSO or with 2 µl of DMSO as control. Pyocyanin extraction and quantification were carried out as described in section 1.2.2. Pyocyanin concentrations (µg $mL_{supernatant}^{-1}$) were calculated by multiplication of its absorbance at 520 nm with a correction factor of 17.072, and normalized to the OD_{600} of the culture.

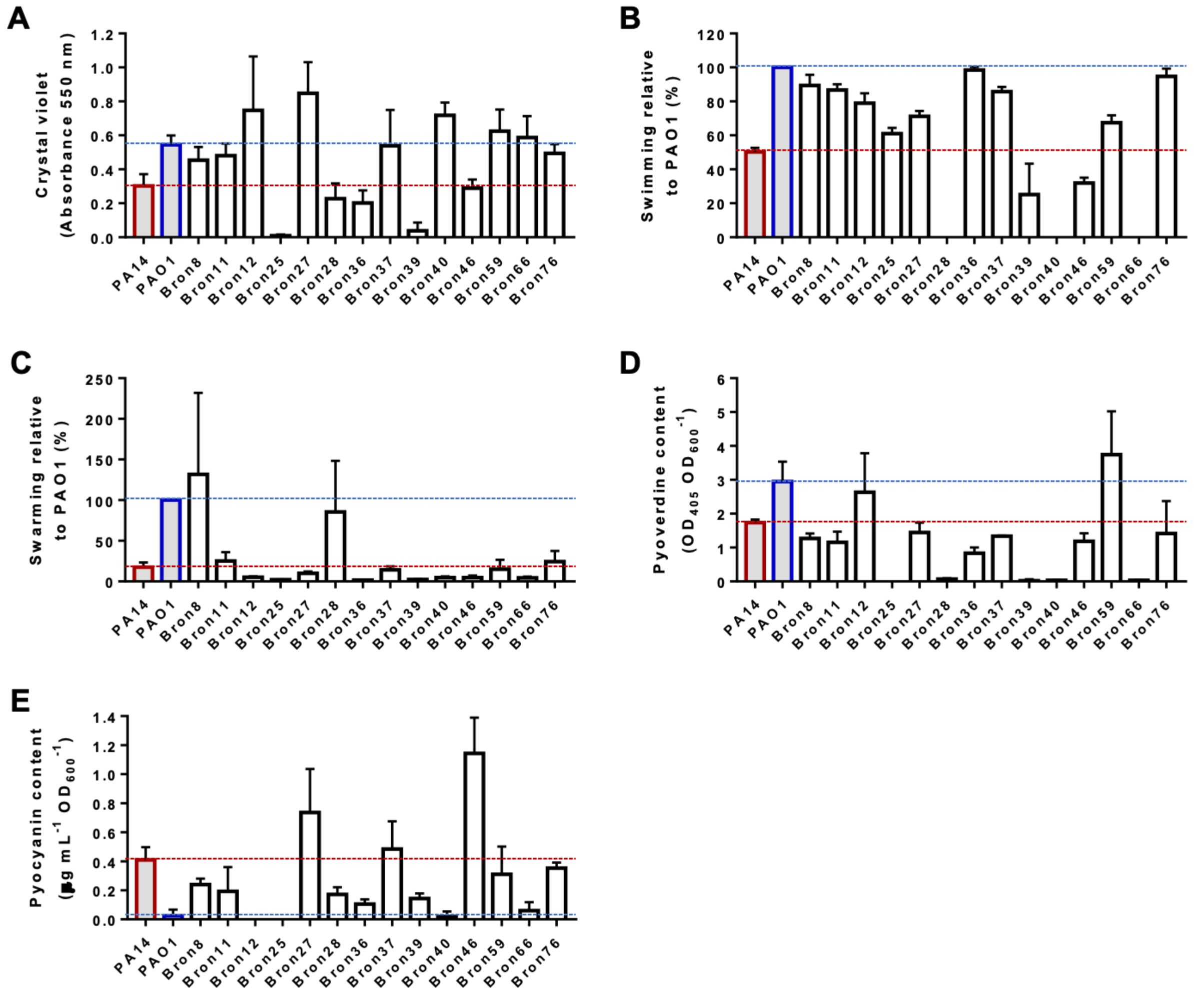

Figure S2. Phenotypic characterization of *P. aeruginosa* clinical isolates from Bronchiectasis patients. Shown are **(A)** biofilm formation of the CIs and type strains determined by crystal violet staining, **(B)** swimming motility of the CIs shown as percentage relative to those of the PAO1 strain, **(C)** swarming motility of the CIs shown as percentage relative to those of the PAO1 strain, **(D)** pyoverdine production in the supernatants of CIs and type strains' cultures grown in minimal medium devoid of iron**,** **(E)** pyocyanin production of CIs and type strains cultured in rich LB-medium. Dashed lines represent the measurements in PA14 and PAO1 laboratory strains for each phenotypic trait. In all cases, the mean and SD of at least three independent measurements are shown.

Determination of antimicrobial minimal inhibitory concentrations (MIC). A microdilution assay was performed to determine the MIC of several classes of antibiotics as follows. Bacterial pre-cultures were grown overnight at 37°C in 3 ml of Mueller-Hinton II broth (MHB-II). On the next day, a 1:30 dilution of each culture was prepared in 3 mL of MHB-II medium and grown at 37°C for 4 h in order to obtain exponentially-grown cells. The bacterial density was measured in the spectrophotometer and each culture was diluted in MHB-II medium to obtain 5 mL of cells at an approximate cell density

of 10^6 CFU/mL (which corresponds approximately to an OD_{600} of 0.001). Next, 100 µL of each bacterial preparation were mixed with 100 µL of each antibiotic dilution previously prepared in MHB-II medium and aliquoted into each well of a 96-well plate. The plates were incubated into a humid chamber at 37°C, and the bacterial growth were monitored after 20 h of incubation. For each antimicrobial, the MIC value corresponds to the lowest antibiotic concentration at which no visible growth was observed. The classification of MIC breakpoints was performed according to the Clinical and Laboratory Standards Institute (CLSI) guidelines.

Table S3. Antimicrobial susceptibility of the Bronchiectasis clinical isolates

Antimicrobial classification	Antibiotic (µg/mL)	Bron 8	Bron 11	Bron 12	Bron 25	Bron 27	Bron 28	Bron 36	Bron 37	Bron 39	Bron 40	Bron 46	Bron 59	Bron 66	Bron 76	PA14	PAO1
aminoglycoside	Tobramycin	1	1	≤ 0.5	≤ 0.5	≤ 0.5	≤ 0.5	≤ 0.5	2	1	≤ 0.5	≤ 0.5	≤ 0.5	≤ 0.5	≤ 0.5	≤ 0.5	≤ 0.5
fluoroquinolone	Ciprofloxacin	1	1	1	1	1	≤ 0.5	4	1	16	1	1	1	≤ 0.5	1	1	1
β-lactam	Ceftazidim	2	4	2	≤ 0.5	2	1	≤ 0.5	4	≤ 0.5	16	≤ 0.5	2	≤ 0.5	4	4	2
polymyxin	Colistin	2	2	1	≤ 0.5	1	2	1	2	2	1	1	2	1	1	1	2

MIC breakpoints according to the CLSI guidelines (in µg/mL)

	Sens	Inter	Resis
Tobramycin	≤ 4	8	≥ 16
Ciprofloxacin	≤ 1	2	≥ 4
Ceftazidim	≤ 8	16	≥ 32
Colistin	≤ 2	4	≥ 8

1.3 DMPK

1.3.1 Apparent permeability in Calu-3 cell line

Permeability of compound **24** was assessed *in vitro* with Calu-3 HTB-55 cell line (ATCC). Before the permeability experiment, cytotoxicity towards the cell line of compound **24** at relevant concentrations was excluded. Cells were cultivated in Minimum Essential Medium supplemented with Earle´s salts, L-glutamine, 10% FCS, 1% non-essential amino acids (NEAA) and 1mM sodium pyruvate. Passages between 35 and 55 were used, medium was changed every 2–3 days. For experiments, cells were harvested using trypsin/EDTA and $1x10^5$ cells seeded on Transwell® inserts 3460. Cells were grown in air-liquid interface beginning day 3 and used for transport studies on day 11–13. TEER values exceeded 300 Ω*cm^2 before beginning transport studies. For experiments, Krebs-Ringer solution with 1% BSA was used and cells were accommodated to the buffer for at least 1 h with no decrease in TEER. 200 µL samples were taken in regular intervals from the apical side (time intervals 0, 20, 40, 60, 90, 120, 180, 240 minutes) and replenished with fresh buffer. TEER was monitored during the experiment and epithelial barriers were considered compromised if the TEER

fell below 300 Ω*cm^2 during 4 h of experiment duration. Sodium-fluorescein and ciprofloxacin-HCl were used as a control. 50 µL of sample was mixed with 150 µL of ice-cold acetonitrile containing internal standard diphenhydramine (1 µM) and the concentration of compound **24** was analyzed with HPLC-ESI-MS/MS in SRM mode tuned to compound **24** and positive ionization mode with the following mass transition: precursor ion m/z 423.048, product ions m/z 191.880 and m/z 218.866.
Results are depicted in Figure S3.

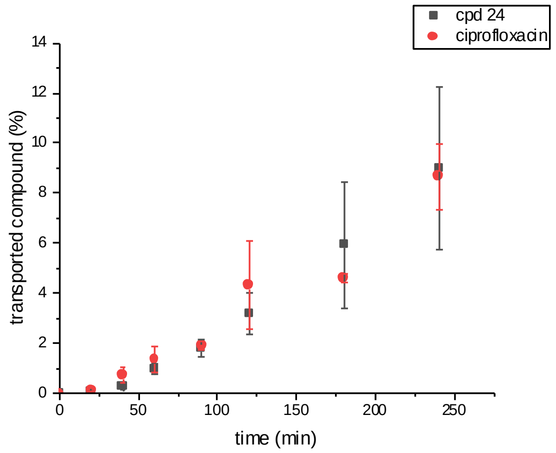

Figure S3. Basolateral compound levels of **24** in comparison to ciprofloxacin in the Transwell® model.

1.3.2 **Metabolic-Stability Tests in Mouse Liver Microsomes (MLM).**

Phase I metabolic stability was evaluated by incubating the compound (1 µM) with 0.5 mg/mL MLM (Corning) and 1 mM NADPH at 37 °C for 0, 5, 10, 15 and 30 min. The enzymatic activity of the MLM was determined by measuring the metabolic stability of Verapamil, Diphenhydramine and Benzydamine (1 µM each). To stop the incubation, 2 volumes of acetonitrile were added containing internal standard (1 µM Leucine Enkephaline) followed by centrifugation of the samples (15 min, 3,500 rpm). The half-life ($t_{1/2}$) was determined by analyzing the concentrations of the compounds at the different time points by HPLC-MS/MS.

1.3.3 **PK and PD Sample Preparation and Analysis**

All PK and PD samples were analyzed *via* HPLC-MS/MS using an Agilent 1290 Infinity II HPLC system coupled to an AB Sciex QTrap 6500plus mass spectrometer. First, a calibration curve was prepared by spiking different concentrations of compound **24** or tobramycin (Fa. Sigma) into plasma, homogenized lung from CD-1 mice or 0.9% sodium chloride solution as matrix for bronchoalveolar lavage fluid (BALF). The lower limits of quantification are indicated in Table S2. Caffeine was used as

internal standard. In addition, quality control samples (QCs) were prepared for **24** and tobramycin in plasma, homogenized lung and 0.9% sodium chloride solution/BALF. The following extraction procedure was used for **24** and tobramycin: 7.5 µL of a plasma sample (calibration samples, QCs or PK samples) was extracted with 10 µL of water containing 10% formic acid and 15 µL of acetonitrile containing 12.5 ng/mL caffeine as internal standard for 5 min at 2000 rpm on an Eppendorf MixMate® vortex mixer. 50 µL of a lung sample (calibration samples, QCs or PK samples) was extracted with 15 µL water containing 10 % formic acid and 35 µL of acetonitrile containing 12.5 ng/mL caffeine as internal standard for 5 min at 2000 rpm on an Eppendorf MixMate® vortex mixer. BALF: 200 µL of methanol were added to 100 µL of a BALF sample (calibration samples, QCs or PK samples). Samples were concentrated in an Eppendorf concentrator until dryness at room temperature. 90 µL of water containing 10% formic acid and 10 µL of acetonitrile containing 125 ng/mL caffeine were added to the dried samples and dissolved for 20 min at 2000 rpm on an Eppendorf MixMate® vortex mixer. All samples were centrifuged for 10 min at 4 °C at 13,000 rpm. Supernatants were transferred to standard HPLC-glass vials. HPLC conditions were as follows: column: Agilent Zorbax Eclipse Plus C18, 50x2.1 mm, 1.8 µm; temperature: 30 °C; injection volume: 10 µL; flow rate: 700 µL/min; solvent A: water + 0.1% formic acid; solvent B: acetonitrile + 0.1% formic acid; gradient for 1121 and tobramycin: 99% A at 0 min, 99% – 0% A from 0.1 min to 4.00 min, 0% A until 4.50 min, 0 % – 99% A from 4.50 to 4.70 min. Mass spectrometric conditions were as follows: Scan type: MRM, positive mode; Q1 and Q3 masses for caffeine, **24** and tobramycin can be found in Table S4; peak areas of each sample and of the corresponding internal standard were analyzed using MultiQuant 3.0 software (AB Sciex). Peak areas of the respective sample of 1121 and tobramycin were normalized to the internal standard peak area. Peaks of PK samples were quantified using the calibration curve. The accuracy of the calibration curve was determined using QCs independently prepared on different days (Table S4). PK parameters were determined using a non-compartmental analysis with PKSolver.[11] ELF concentrations were calculated using the following formulae:

(1) $V_{ELF} = V_{BALF} \times \frac{Urea_{BALF}}{Urea_{Plasma}}$

(2) $c_{ELF} = c_{BALF} \times \frac{V_{BALF}}{V_{ELF}}$

Table S4. Information on the calibration curve for compound **24** and Tobramycin.

ID	limits of quantification [ng/mL]	lower limit of qualification [ng/mL]	accuracy [%]
**24**			
**Plasma**	25–8000	10	111.8–114.45
**lung**	4–8000	2.5	89.28–113.05
**BALF**	250–8000	100	100.00–110.65
**Tobramycin**			
**Plasma**	40–8000	25	88.23–114.15
**lung**	40–8000	25	86.51–109.87
**BALF**	4–8000	2.5	86.55–109.38

Table S5. Information on the tuning for compound **24** and Tobramycin as well as urea and caffeine references.

ID	Q1 Mass [Da]	Q3 Mass [Da]	time [msec]	CE [volts]	CXP [volts]	DP [volts]
**24**	423.021	219.000	30	35.0	26.0	166.0
**24**	423.021	261.100	30	25.0	12.0	166.0
**Tobramycin**	468.164	163.000	30	1.0	29.0	18.0
**Tobramycin**	468.164	324.000	30	1.0	21.0	36.0
**Tobramycin**	468.164	205.000	30	1.0	29.0	22.0
**Urea**	60.915	43.800	30	17.0	16.0	56.0
**Urea**	60.915	43.100	30	53.0	12.0	56.0
**Urea**	60.915	29.100	30	111.0	6.0	56.0
**Caffeine**	195.024	138.000	30	25.0	14.0	130.0
**Caffeine**	195.024	110.000	30	31.0	18.0	130.0

1.3.4 **Kinetic Turbidimetric Aqueous Solubility**

The aqueous solubility assay was performed at room temperature for 5 min. Different concentrations of the test compound (0.4, 2, 4, 20, 40, 100, and 200 µM, final DMSO concentration 2%) were prepared by dilution in PBS buffer followed by a 5 min incubation at room temperature. Absorbance of the samples was measured then at a wavelength of 620 nm. The solubility of the compound was determined as the concentration that shows increased absorbance above the vehicle control (*i.e.*, 1% DMSO in buffer). Analysis of the data involved the fitting of the measured and normalized absorbances in a four-parameter sigmoidal function. The LogS (decadic logarithm of the solubility) was calculated from the AUC.

1.4 Safety Pharmacology of Compound 24

1.4.1 Cerep panel (Eurofins)

Compound **24** was further profiled for its off-target interactions against a panel of 44 targets. The screening was done by Eurofins Pharma Discovery within the SafetyScreen44 Panel (Cerep, ITEM P270). In all assays binding/functional inhibition was determined at 10 µM.

1.4.1.1 Binding Assays

Table S6. List of binding assays within the CEREP44 off-target panel and binding data for compound **24**.

ASSAY NAME	BINDING (%)
Ca^{2+} channel (L, dihydropyridine site) (antagonist radioligand)	75.3
Norepinephrine transporter (h) (antagonist radioligand)	74.7
Potassium Channel hERG (human) [3H] Dofetilide	73.1
5-HT_{2B} (h) (agonist radioligand)	62.2
Na^{+} channel (site 2) (antagonist radioligand)	57.2
5-HT transporter (h) (antagonist radioligand)	49.8
CB_2 (h) (agonist radioligand)	31.0
MAO-A (antagonist radioligand)	24.9
kappa (h) (KOP) (agonist radioligand)	23.6
dopamine transporter (h) (antagonist radioligand)	22.7
CB_1 (h) (agonist radioligand)	16.3
GR (h) (agonist radioligand)	16.2
delta (DOP) (h) (agonist radioligand)	14.5
KV channel (antagonist radioligand)	14.2
AR (h) (agonist radioligand)	12.7
D_1 (h) (antagonist radioligand)	12.2
5-HT_{1A} (h) (agonist radioligand)	11.3
$alpha_{2A}$ (h) (antagonist radioligand)	8.4
H1 (h) (antagonist radioligand)	8.2
mu (MOP) (h) (agonist radioligand)	8.2
$beta_2$ (h) (antagonist radioligand)	7.9
V1a (h) (agonist radioligand)	7.6

$beta_1$ (h) (agonist radioligand)	7.1
5-HT_3 (h) (antagonist radioligand)	4.4
$alpha_{1A}$ (h) (antagonist radioligand)	3.4
A2A (h) (agonist radioligand)	2.2
NMDA (antagonist radioligand)	0.9
M2 (h) (antagonist radioligand)	0
ETA (h) (agonist radioligand)	–0.3
D2S (h) (agonist radioligand)	–0.6
M3 (h) (antagonist radioligand)	–2.0
5-HT_{2A} (h) (agonist radioligand)	–2.8
N neuronal $alpha_4beta_2$ (h) (agonist radioligand)	–4.7
M1 (h) (antagonist radioligand)	–5.9
5-HT_{1B} (h) (antagonist radioligand)	–6.7
CCK1 (CCKA) (h) (agonist radioligand)	–12.0
BZD (central) (agonist radioligand)	–27.1
H2 (h) (antagonist radioligand)	–30.0

1.4.1.2 Functional Assays

Table S7. List of functional assays within the CEREP44 off-target panel and inhibition by compound **24**.

**ASSAY NAME**	**INHIBITION (%)**
COX-2 (h)	19.1
COX-1 (h)	8.8
PDE4D2 (h)	6.7
PDE3A (h)	0.3
acetylcholinesterase (h)	–1.9
Lck kinase (h)	–6.8

1.4.2 **Further functional off-target assays (Eurofins)**

Table S8. Compound 24 was further tested in the following functional assays at Eurofins.

ASSAY NAME	ESTIMATED IC_{50} [µM]
NET human norepinephrine transporter functional antagonist uptake assay (302000)	>10 µM
Cav1.2 (L-type) human Ca ion channel cell based automated patch clamp assay (CYL8051QP2DR)	>30 µM
Nav1.5 human sodium ion channel cell based automated patch clamp CiPA assay (CYL8004QP2DR)	>30 µM
HTR2B Human 5-Hydroxytryptamine GPCR calcium assay in arrestin cell line in agonist mode, DiscoverX (86-0030P-2091AG)	>10 µM
HTR2B Human 5-Hydroxytryptamine GPCR calcium assay in arrestin cell line in agonist mode, DiscoverX (86-0030P-2091AN)	>10 µM

1.4.3 **Ion Channel Profiling at Charles River**

Table S9. Compound **24** was tested against the following ion channels at Charles River Laboratories.

ASSAY TYPE	ION CHANNEL AND EXPRESSION SYSTEM	ESTIMATED IC_{50} [µM]
FASTPatch Assay	BK (K_{Ca}1.1/β1) calcium-activated potassium channel (human KCNMA1 and KCNMB1 genes) expressed in CHO cells	>30
	IK (K_{Ca}3.1) calcium-activated potassium channel (human KCNN4 gene) expressed in CHO cells	>30
	Cloned hKv1.5 potassium channels (encoded by the human KCNA5 gene, expressed in CHO cells)	32.6
ScreenPatch Assay	CFTR cystic fibrosis transmembrane conductance regulator (Cl^- channel encoded by human CFTR gene) expressed in CHO cells	>30
	Kv1.3 potassium channel (human KCNA3 gene) expressed in CHO cells.	>30
FLIPR Assay	ENaC channel (human SCNN1A, SCNN1B and SCNN1G genes, expressed in HEK293 cells	>30
	$GABA_A$ α5/β3/γ2 ionotropic receptor (human GABRA5, GABRB3, and GABRG2 genes, expressed in HEK293 cells	>30

	P2X4 receptor (human P2RX4 gene) expressed in HEK293 cells	>30
	TRPA1 channel (human TRPA1 gene) expressed in CHO cells	>30
	TRPC6 channel (human TRPC6 gene) expressed in HEK293 cells	>30
	TRPV1 channel (human TRPV1 gene) expressed in HEK293 cells	>30
	TRPV4 channel (human TRPV4 gene) expressed in CHO cells	>30

1.4.4 **Pharmacokinetic and off-target functional assays (Cyprotex)**

Compound **24** was assayed at Cyprotex in functional assays for interference with important off-targets as well as important pharmacokinetic parameters. With the exception of plasma protein binding, a dose-response relationship was studied in order to calculate IC_{50} values.

Table S10. Information on in vitro ADMET assays performed at Cyprotex.

Assay	Assay type
Cytochrome P450 (CYP) inhibition assay (IC_{50})	Isoform specific substrate metabolization rate
Plasma protein binding assay (human and murine)	Equilibrium dialysis
AhR Nuclear Receptor Activation	Luciferase reporter gene assay
hERG safety	Single cell electrophysiology

1.4.5 **Mini-AMES (Cyprotex)**

Approximately ten million bacteria were exposed in triplicate to **24** (1.9, 3.9, 7.8, 15.6, 31.2, 62.5 µg/mL), a negative control (vehicle) and positive controls for 90 min in medium containing a low concentration of histidine (sufficient for about 2 doublings.) The cultures were then diluted into indicator medium lacking histidine, and dispensed into 48 wells of 384-well plates (micro-plate format, MPF). The plates were incubated for 48 h at 37 °C, and cells that underwent a reversion grew, resulting in a color change in the wells with growth. The wells showing growth were counted and compared to the vehicle control. An increase in the number of colonies of at least two-fold over baseline (mean + SD of the vehicle control) and a dose response indicate a positive response. An unpaired, one-sided Student's T-test was used to identify the conditions that were significantly different from the vehicle control.

Where indicated, S9 fraction from the livers of Aroclor 1254-treated rats were included in the incubation at a final concentration of 4.5%. A NADPH-regenerating system was also included to ensure a steady supply of reducing equivalents.

Strains used in this study:

Salmonella typhimurium TA98: hisD3052, rfa, uvrB / pKM101; detects frame-shift mutations.

Salmonella typhimurium TA100: hisG45, rfa, uvrB / pKM101; detects base-pair substitutions.

1.4.6 **Mouse Maximum tolerated dose (MTD) study (Aurigon)**

The maximum tolerated dose of **24** in NMRI mice was conducted and analyzed at CRO ATRC Aurigon Toxicological Research Center Ltd, Hungary. The study was conducted in accordance with Standard Operating Procedures of ATRC and the requirements of EEC Directive 2001/83/EC of the European Parliament and of the Council of November 6, 2001 on the Community Code relating to Medicinal Products for human use (Official Journal L -311,28/11/2004, p.67–128) as amended by EEC Directive 2004/27/EC of the European Parliament and the Council of March 31, 2004 amending Directive 2001/83/EC on the Community code relating to medicinal products for human use (Official Journal L - 136, 30/04/2004, p. 34-57), ICH Guideline M3(R2) "Guidance on Nonclinical Safety Studies for the Conduct of Human Clinical Trials and Marketing Authorization for Pharmaceuticals", ICH Guideline S3A "Toxicokinetics: The Assessment of Systemic Exposure in Toxicity Studies", OECD Guideline for the Testing of Chemicals: Toxicokinetics, (OECD 417, 2010). This study was conducted in compliance with the principles of Hungarian Act 1998: XXVIII regulating animal protection (latest modified by Act 2011 CLVIII) and in Government Decree 40/2013 on animal experiments. Handling and care of the animals will be conducted according to the Guide for the Care and Use of Laboratory Animals, NRC, 2011.

Animals were 6 weeks old and weighed 27–34 g. **24** was applied subcutaneously in a formulation containing 5% DMSO, 10% Cremophor EL® and 85% PBS first at 10 mg/kg and mice were observed for 48 h. As no clinical findings were observed, 30 mg/kg was then applied to the next group. Likewise, 60 mg/kg were applied 48 h after. Mortality was observed twice a day. Clinical observation was performed directly after dosing, 1 h, 2 h, 4 h, 24 h and 48 h after administration. Body weight was registered prior to treatment and daily after the dosing for 48 h. For serum concentration determination, 100 µL blood were collected from each animal at 0.5 h and 4 h time points after the administration, processed to plasma and analyzed using HPLC-MS/MS at HZI. Each group consisted of 5 male and 5 female mice, 2 of which were used for blood collection.

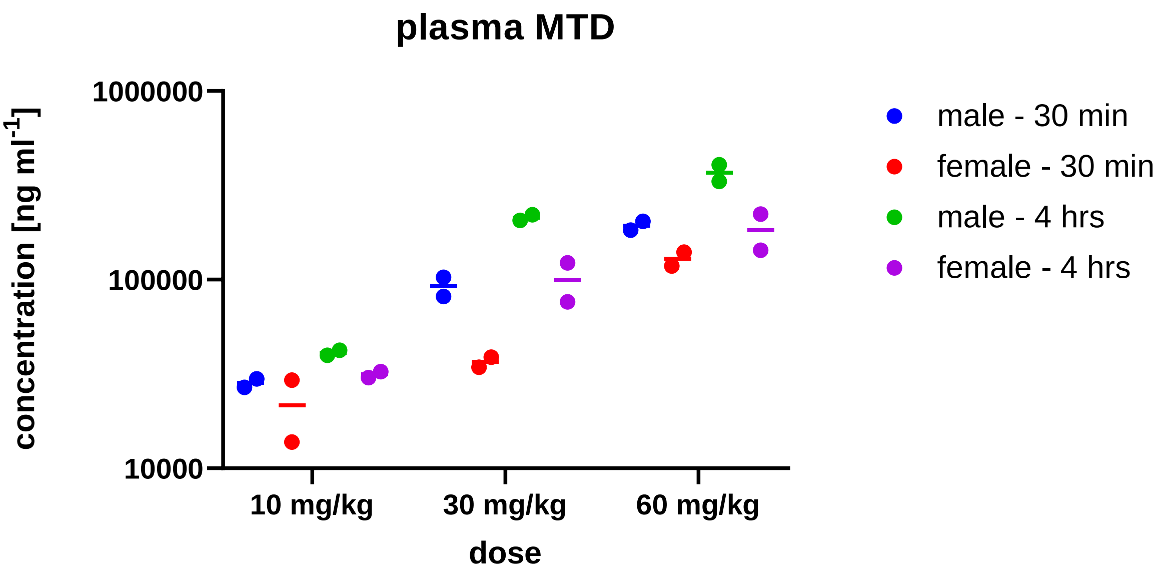

Figure S4. Plasma levels at 0.5 and 4 h after 30 and 60 mg/kg SC administration of **24**.

1.4.7 *In vivo* PK studies

The in live part for the IV and IT PK studies were performed at CRO Saretius (UK). Groups of male CD1 mice (25–30 g, Charles River UK) were housed in groups of up to 6 and maintained under a 12 h light/dark cycle with free access to food and water. Temperature and humidity were controlled according to U.K. HO regulations. On the day of the study, mice were briefly anesthetized with isoflurane before suspending the mouse by its upper incisors on a board held at an angle of approx. 45 degrees. Mice were then dosed intratracheally with the compound (**24** or Tob) at the designated does formulated in ethanol/tyloxapol/glycerol/phosphate-buffered saline (PBS) [10:1:5:84] in a 1 mL/kg dosing volume up to a maximum of 30 µL per mouse. A laryngoscope was used to facilitate dosing. Animals were allowed to recover before replacing into their home cages. Terminal blood samples were then taken from groups of 3 mice at each of eight time points post-dose by cardiac puncture under CO2. Blood samples were placed in tubes on ice containing EDTA anticoagulant. After gentle mixing, samples were centrifuged (10,000 rpm x 3 min) and two 50 µL aliquots taken into fresh tubes that were immediately frozen on dry ice. Following blood sample collection, tracheas were exposed and a small incision made. An 18 Gauge plastic tube was then inserted through the incision into the trachea and tied in place. Aliquots of 0.8 mL of sterile saline were then infused into and then withdrawn from the lungs and the collected bronchoalveolar lavage (BALF) sample placed into a tube. This process was repeated two more times, and the samples from each mouse pooled.

The resulting samples were weighed and mixed, before a 1 mL aliquot was taken for analysis and frozen on dry ice. Immediately after collecting the final BALF sample, lung samples were excised and excess tissue removed, before rinsing in saline, blotting and weighing. Samples were then snap frozen by immersion in liquid nitrogen. All samples were stored at –20 °C until sent for analysis on dry ice. Analysis of the samples was done as described below.

For pharmacokinetic experiments *via* the intraperitoneally (IP), subcutaneously (SC) and perorally (PO) route 4 weeks old outbred male CD-1 mice (Charles River, Netherlands) were used. All animal experiments were performed in compliance with the German animal welfare law (TierSchG BGBl. S. 1105; 25.05.1998). The mice were housed and handled in accordance with good animal practice as defined by the Federation of Laboratory Animal Science Associations (FELASA). All animal experiments were approved by the Lower Saxony State Office of Consumer Protection and Food Safety (Oldenburg, Germany). **24** was dissolved in 5% DMSO, 10% Cremophor EL and 85 % PBS. Mice were administered **24** at 20 mg/kg intraperitoneally (IP), subcutaneously (SC) and perorally (PO). About 20 µL of whole blood was collected serially from the lateral tail vein at time points t = 0.25 (only IP and SC), 0.5, 1, 2, 4, and 8 h post administration. After 24 h mice were euthanized, and blood was collected from the heart. Whole blood was collected into Eppendorf tubes coated with 0.5 M EDTA and immediately spun down at 13,000 rpm for 10 min at 4 °C. The plasma was transferred into a new Eppendorf tube and then stored at −80 °C until analysis. Spontaneous urine was also collected at the indicated time points.

Table S11. PK parameter plasma 24 and tobramycin 0.25 mg/kg IT and IV

	**24** IT	Tobramycin IT	**24** IV	Tobramycin IV
t1/2 [h]	0.81 ± 0.0	0.81 ± 0.2	1.05 ± 0.1	0.25 ± 0.1
Tmax [h]	0.67 ± 0.3	0.67 ± 0.3		
Cmax [ng/mL]	4,183.67 ± 930.7	272.90 ± 165.6		
C0 [ng/mL]			36,449.95 ± 8344.2	588.92 ± 222.9
AUC 0-t [ng/mL*h]	5,146.17 ± 650.8	306.45 ± 154.0	19,300.89 ± 719.1	141.70 ± 11.7
MRT [h]	1.32 ± 0.1	1.50 ± 0.1	1.08 ± 0.3	0.28 ± 0.1
Vz/F_obs [L/kg]	0.06 ± 0.0	1.02 ± 0.4	0.02 ± 0.0	0.60 ± 0.2
Cl/F_obs [mL/min/kg]	0.80 ± 0.1	15.20 ± 8.0	0.21 ± 0.0	27.99 ± 1.8

Table S12. PK parameter ELF and lung tissue 24 and tobramycin 0.25 mg/kg IT and IV

	**24** IT	Tobramycin IT	**24** IV	Tobramycin IV
ELF Tmax [h]	0.5 ± 0.	0.67 ± 0.3	0.67 ± 0.9	0.14 ± 0.1
ELF Cmax [ng/mL]	898,155.34 ± 288,396.28	171,532.68 ± 133,180.1	725,227.13 ± 201,930.8	520.23 ± 210.6
ELF AUC 0-t [ng/mL*h]	1,602,310.98 ± 169,233.3	190,112.59 ± 125,195.6	1,607,478.49 ± 270,147.7	135.28 ± 84.3
ELF/plasma AUC ratio	311.4	620.37	83.3	0.95
Lung Tmax [h]	0.67 ± 0.3	0.83 ± 0.3	0.08 ± 0.0	-
lung Cmax [ng/g]	859.15 ± 292.1	478.52 ± 66.5	2,201.11 ± 254.6	-
lung AUC 0-t [ng/g*h]	962.85 ± 252.3	1,020.00 ± 258.9	1,280.59 ± 73.5	-

Table S13. PK parameter plasma 24 20 mg/kg IP, SC and PO

	**24** IP	**24** SC	24 PO
t1/2 [h]	1.68 ± 0.0	1.51 ± 0.0	1.90 ± 0.5
Tmax [h]	1.00 ± 0.00	2.00 ± 0.00	0.83 ± 0.2
Cmax [ng/mL]	65,775.00 ± 6,427.6	44,2210 ± 5,317.1	45,616.67 ± 10,554.5
AUC 0-t [ng/mL*h]	557,069.64 ± 242,342.5	363,325.17 ± 58,722.2	213,075,71 ± 45,802,5
Vz/F_obs [L/kg]	0.25 ± 0.3	0.12 ± 0.0	0.25 ± 0.0
Cl/F_obs [mL/min/kg]	1.70 ± 1.8	0.93 ± 0.1	1.58 ± 0.4
Bioavailability [%]	36.1	23.5 %	13.8 %

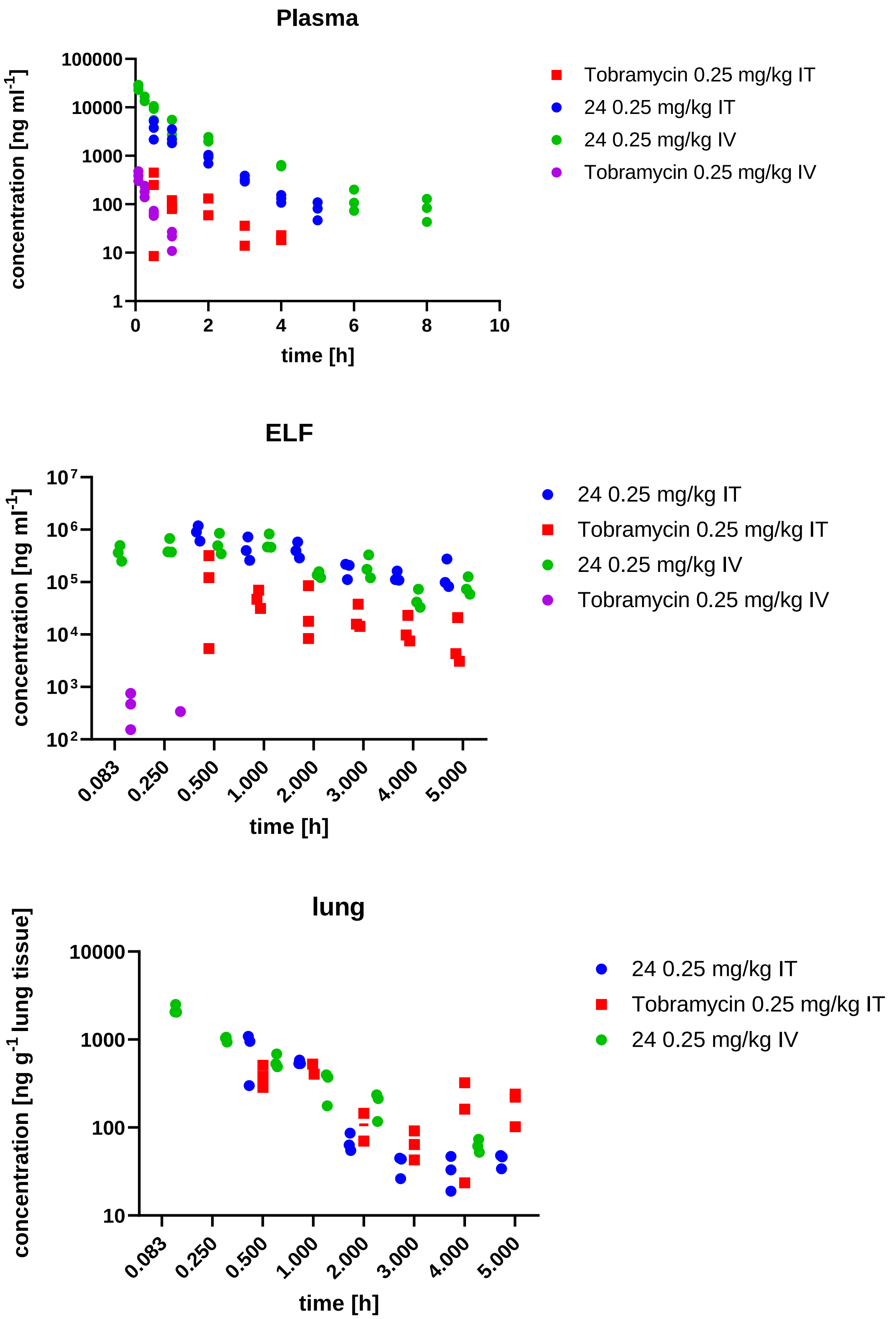

Figure S5. Plasma, ELF and lung tissue levels at varying time points after 0.25 mg/kg IV and IT administration of tobramycin and **24**.

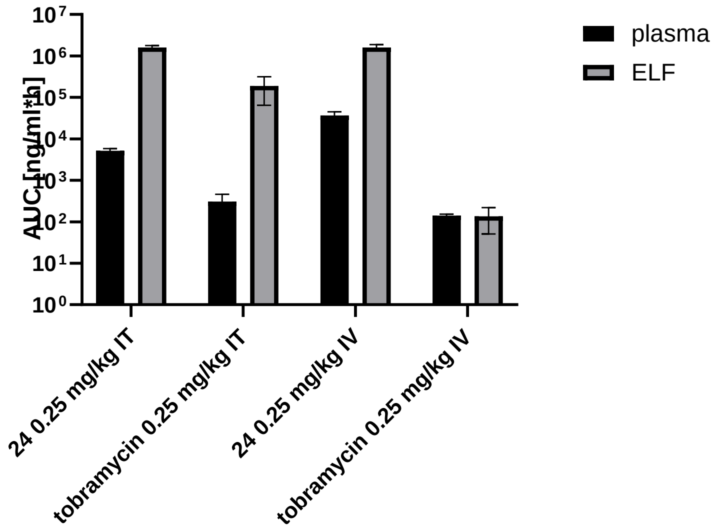

Figure S6. AUC levels after IV and IT administration

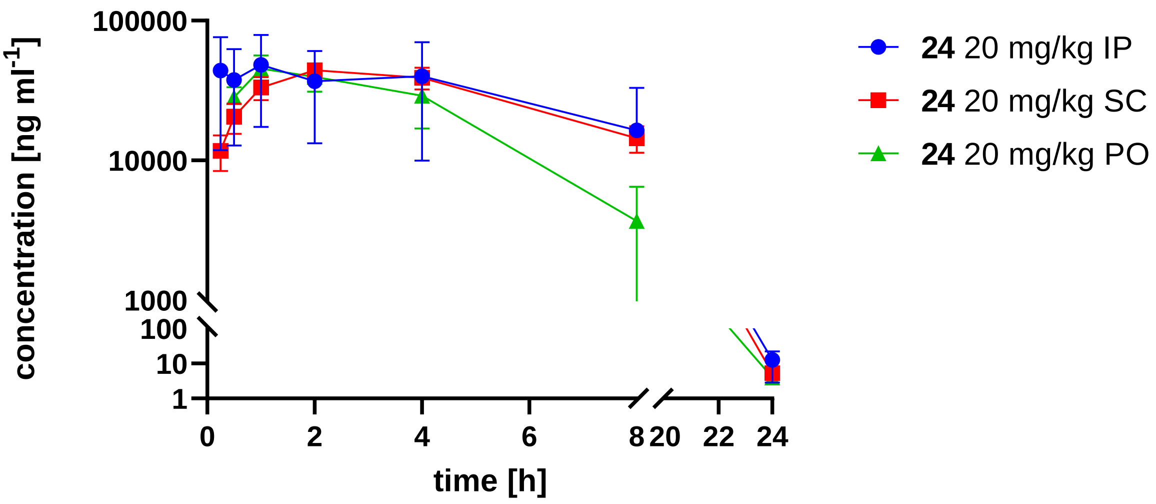

Figure S7. Plasma levels after 20 mg/kg IP, SC and PO administration of **24**.

1.4.8 ***In vivo* thigh infection model with *P. aeruginosa***

Male, 6 weeks-old, CD-1 mice (Charles River, Netherlands) were rendered neutropenic by administration of 150 mg/kg and 100 mg/kg cyclophosphamide i.p. on day –4, –1 and +1, respectively. The *P. aeruginosa* strain ATCC 27853 was used. The inoculum was prepared as follows: on day –1 the *P. aeruginosa* strain was streaked out onto a blood agar plate and incubated at 37 °C. Then one single colony was inoculated into MHB medium (diluted 1:2 in water) and incubated at 120 rpm and 37 °C. On day 0, bacteria were centrifuged for 15 min at 4,000 rpm and washed twice in

PBS. Then they were adjusted to an OD of 1 and diluted 1:5000 in PBS. The inoculum was plated onto MHB agar plates in serial dilutions and incubated at 37 °C. Mice were grouped into the following (n=18 for each group): (1) baseline inoculum group, sacrificed 2 h post infection; (2) vehicle control group (receiving vehicle t= 2, 12, 24 and 36 h post infection as well as 20 mg/kg tramadol SC t= 0 and 24 h post infection); (3) tobramycin group I (8 mg/kg tobramycin IV at t= 2 and 24 h post infection); (4) tobramycin group II (1 mg/kg tobramycin IV at t= 2 and 24 h post infection); (5) **24** group (30 mg/kg **24** SC at t=2, 12, 24 and 36 h post infection); (6) **24** + tobramycin group (30 mg/kg **24** SC at t=2, 12, 24 and 36 h and tobramycin 1 mg/kg IV at t= 2 and 24 h post infection). On the day of infection (day 0), mice received 30 µL of the *P. aeruginosa* strain ATCC 27853 into each lateral thigh (inoculum: $1x10^5$ cfu/mL). 48 h after infection, mice were euthanized, blood was removed from the heart, lung and thighs were aseptically removed. Whole blood was collected into Eppendorf tubes coated with 0.5 M EDTA and immediately spun down at 13,000 rpm for 10 min at 4 °C. The plasma was transferred into a new Eppendorf tube and then stored at −80 °C until analysis. Organs were homogenized in 0.9% NaCl-solution and plated onto MHB agar plates in duplicate in serial dilutions and incubated at 37 °C for 24 h. Blood, lung and thighs were also subjected to LC-MS/MS analysis.

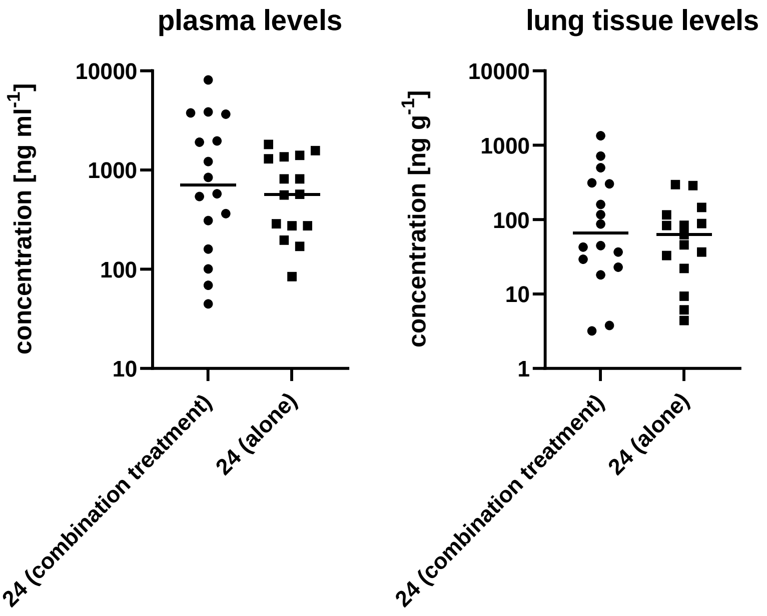

Figure S8. Plasma and lung-tissue levels after the *in vivo* infection model.

1.5 X-ray crystallography

1.5.1 Expression and Purification of PqsR$^{91\text{-}319}$

The plasmid pOPIN-His$_6$-SUMO-PqsR$^{91\text{-}319}$ was transformed into *E. coli* BL21-CodonPlus(DE3)-RIL and protein expression in TB (+Amp+Cm) was induced with 0.5 mM IPTG for 16 h at 20 °C when the culture reached an OD600 of 0.6-0.8. After centrifugation, the cells were resuspended in buffer A (20 mM HEPES 8.0, 300 mM NaCl, 5mM BME) supplemented with one EDTA-free protease inhibitor cocktail tablet (Roche Life Science). The cells were lysed with an Emulsiflex-C3 homogenizer (Avestin) with two cycles. Supernatant was separated from pellet by centrifugation in a SA-60 rotor at 16000 rpm for 30 min. The supernatant was applied onto a 5 mL HisTrap HP column (GE Healthcare). His$_6$-SUMO-PqsR$^{91\text{-}319}$ was eluted with a linear gradient (20 mM HEPES pH 8.0, 300 mM NaCl, 0–250 mM imidazole pH 8.0). His$_6$-SUMO-PqsR91-319 containing fractions were pooled and after cleavage o.n. of the His$_6$-SUMO tag by SUMO protease (1%, *e.g.* 0.01 mg SUMO protease per 1 mg of His$_6$-SUMO-PqsR91-319; dialyzed in parallel against buffer without imidazole) and a second (reverse) nickel affinity chromatography, PqsR$^{91\text{-}319}$ was finally purified by size exclusion chromatography in 20 mM HEPES, 150 mM NaCl, 1 mM DTT, pH 8.0. The protein was concentrated and flash-frozen in liquid nitrogen and stored at –80 °C.

1.5.2 Protein Crystallization, Data Collection and Structure Solution

Crystals of PqsR$^{91\text{-}319}$ in complex with compound **24** were obtained by co-crystallization in a sitting drop vapor diffusion set-up. Prior crystallization 10 mg/mL PqsR$^{91\text{-}319}$ was mixed with 1.0 mM compound **24** incubated for 10 min prior centrifugation at 14k rpm for 5 min. Equal amounts of protein were mixed with 30 mM magnesium formate, 120 mM lithium citrate, 20% (v/v) MPD and 0.1 M MES pH 5.6. Crystals grew to full size (140x140x110 µm) within 27 days at 20 °C. The crystals were cryoprotected with 20% (v/v) MPD and flash-frozen in liquid nitrogen. X-ray diffraction data were collected at 100 K at the Swiss Light Source (SLS) synchrotron beamline X06DA (PSI Villigen, Switzerland) on a Dectris Pilatus 2 M CCD detector (Table S13). Diffraction data were processed with Autoproc[12] including STARANISO package.[13] The data was corrected for anisotropy with STARANISO. The structure was determined by molecular replacement with PHASER[14] using the PDB entry 2Q7V. Model building and refinement were performed with COOT4 and with phenix.refine[15] from the PHENIX suite,[16] respectively.

**Table S14**: Data collection and refinement statistics	
	$PqsR^{91\text{-}319}$ – 24
**Data collection statistics**	
Beamline[a]	SLS BEAMLINE X06DA, DECTRIS PILATUS 2M
Wavelength (Å)	1.0
Space group	$P6_522$
Unit cell dimensions	
a, b, c (Å)	120.3, 120.3, 115.2
α, β, γ (°)	90.0, 90.0, 120.0
Resolution range (Å)	60.1–2.74 (2.99–2.74)
Ellipsoidal[a] resolution (Å) (direction)[b]	3.31 (a*)<br>3.31 (b*)<br>2.62 (c*)
Mosaicity (°)	0.11
Total No. of	
measured reflections	335556 (18279)** (ellipsoidal)
unique reflections	9271 (464) (ellipsoidal)
Mean I/σ(I)	32.6 (1.3) (ellipsoidal)
Multiplicity	36.2 (39.4)
Completeness (spherical) (%)	69.2 (15.9)
Completeness (ellipsoidal) (%)	95.2 (81.9)
R_{meas}[c] (%)	9.2 (363.4)
$R_{p.i.m.}$[d] (%)	1.5 (57.3)
$CC_{1/2}$[e]	100.0 (61.0)
Solvent content (%)	73.7
Monomers/ASU	1
**Refinement statistics**	
Resolution range (Å)	60.16 –2.74 (2.99–2.74)
R_{work} (%)	24.8
R_{free} (%)	27.5
No. of non H-atoms	
Protein	1553
24	29
Water	7
R.m.s. deviations	
Bonds (Å)	0.005
Angles (°)	0.806
Ramachandran plot (%)	
Favored regions	92.6
Outliers	0.0
MolProbity score	1.64
PDB ID	7P4U

*Data for PqsR91-319 in complex with compound 9a were processed anisotropically using unmerged data *via* the STARANISO server.[13]

**Values in parentheses refer to the highest resolution shell.

[a] The statistics are for data that were corrected for anisotropy by STARANISO.[13]

[b] The resolution limits for three directions in reciprocal space (a*, b*, c*) are indicated here.

[c] $R_{meas} = \Sigma_{hkl} [N / (N - 1)]^{1/2} \Sigma_i |I_i(hkl) - \langle I(hkl)\rangle| / \Sigma_{hkl} \Sigma_i I_i(hkl)$, where N is the multiplicity, $I_i(hkl)$ is the intensity of the measurement of the reflection hkl and $\langle I(hkl)\rangle$ is the mean intensity of multiple observations of the reflection hkl.

[d] $R_{p.i.m.} = \Sigma_{hkl} [1 / (N - 1)]^{1/2} \Sigma_i |I_i(hkl) - \langle I(hkl)\rangle| / \Sigma_{hkl} \Sigma_i I_i(hkl)$.

[e] $CC_{1/2}$: Correlation coefficient between the intensities of two random half data sets.

1.6 Molecular modeling

Modeling of compound conformations was performed with MOE 2021.09 (Molecular Operating Environment, Chemical Computing Group),[17] while graphic processing for manuscript figures was done using YASARA structure (YASARA Biosciences GmBH)[18] and POV-Ray 3.7.0.

First, the structures of compounds **19** and **21** were assembled in MOE in a superimposed orientation using the built-in “Builder” function. Then both compounds were energy-minimized in vacuum using Amber10:EHT as force field. Angles between aromatic systems were measured by investigating the corresponding dihedral angles.

1.7 Statistics

Statistical Analysis

Results of biological experiments are reported as means of at least three independent experiments, unless otherwise stated.

For MBEC determination, One-way ANOVA was performed using GraphPad Prism 8.4.2.

For animal studies, unpaired t-test (one-sided, p-value) was performed with Microsoft Excel.

A summary of data processing and statistical analysis is shown in Table S15. Detailed information can be found in the respective experimental section.

The CEREP panel, functional off-target assays, determination of CYP and hERG inhibition, mini-AMES, as well as Mouse MTD were performed at CROs.

Table S15. Details on the analysis for different assays used.

**Test**	**Preprocessing**	**Presentation***	**N**	**Statistical Method**	**Software**
Cytotoxicity Assays	Normalization to DMSO control, mean of 2 technical replicates	Mean % viable cells	2	-	Microsoft Excel
Kinetic Turbidimetric Solubility	Blank subtraction	Mean	3	AUC Determination	GraphPad Prism 8.4.2
Metabolic Stability	Mean of 2 technical replicates	Mean ± SD	2	Linear regression of semi-logarithmic plot	Microsoft Excel
Permeability	mass correction for cumulative sampling	Mean P_{app} ± standard deviation	N = 3 individual experiments with each 3-4 replicates	none	Excel
Biofilm formation and eradication	Four independent experiments with four technical replicates	Scatter plot with mean and SEM	4	one-way ANOVA with Tukey's multiple	GraphPad Prism 8.4.2

(MBEC)	(n=4)			comparisons test	
Pyocyanin assay	Blank subtraction, Normalization to cell growth	Dose response curve, mean ± SD/CI	3	Non-linear regression	Microsoft Excel, OriginPro 2020
Pharmaco-kinetics	Normalization of peak area to internal standard peak area, calculation of conc. based on calibration curve and quality control samples	Scatter plot including mean concentration vs. time; mean ± SE	3	-	Microsoft Excel<br>PK Solver
Alkylquinolone Inhibition Assay	Normalization of peak area to internal standard, mean of 3 technical replicates, calculation of concentration based on a calibration curve	Dose response curve, mean w/o error	3	Non-linear regression	Microsoft Excel, OriginPro 2020
In vivo CFU	Normalization to volume used for counting, normalized to g/tissue for every individual animal	Single values, mean box plot	n=18 per group	ordinary one-way ANOVA	GraphPad Prism 9.4.1
Reporter Gene Assay	Normalization to solvent control	Mean IC_{50}/CI	≥2	Non-linear regression	OriginPro 2020
X-ray	See Table S14				

*SD = Standard deviation; CI = confidence interval of 95%

2 ^{1}H- AND ^{13}C-NMR SPECTRA OF THE DESCRIBED COMPOUNDS

Compound **2**

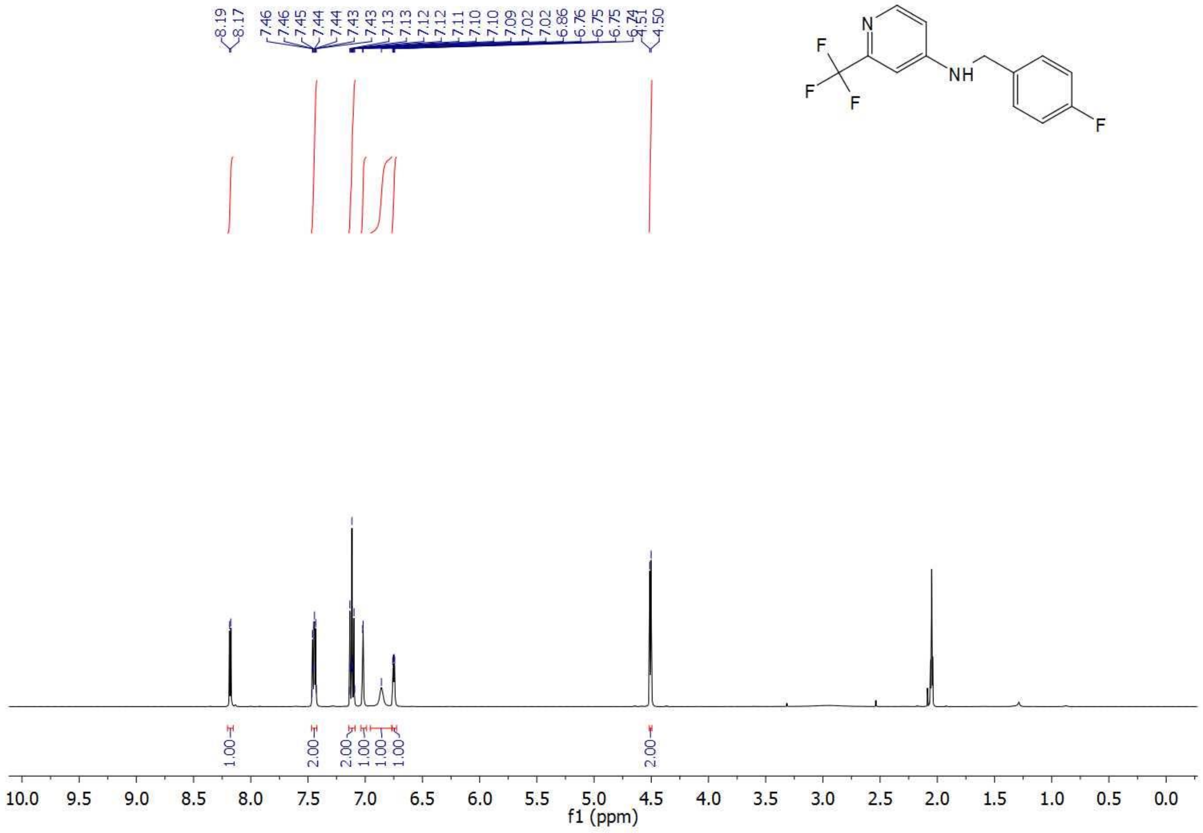

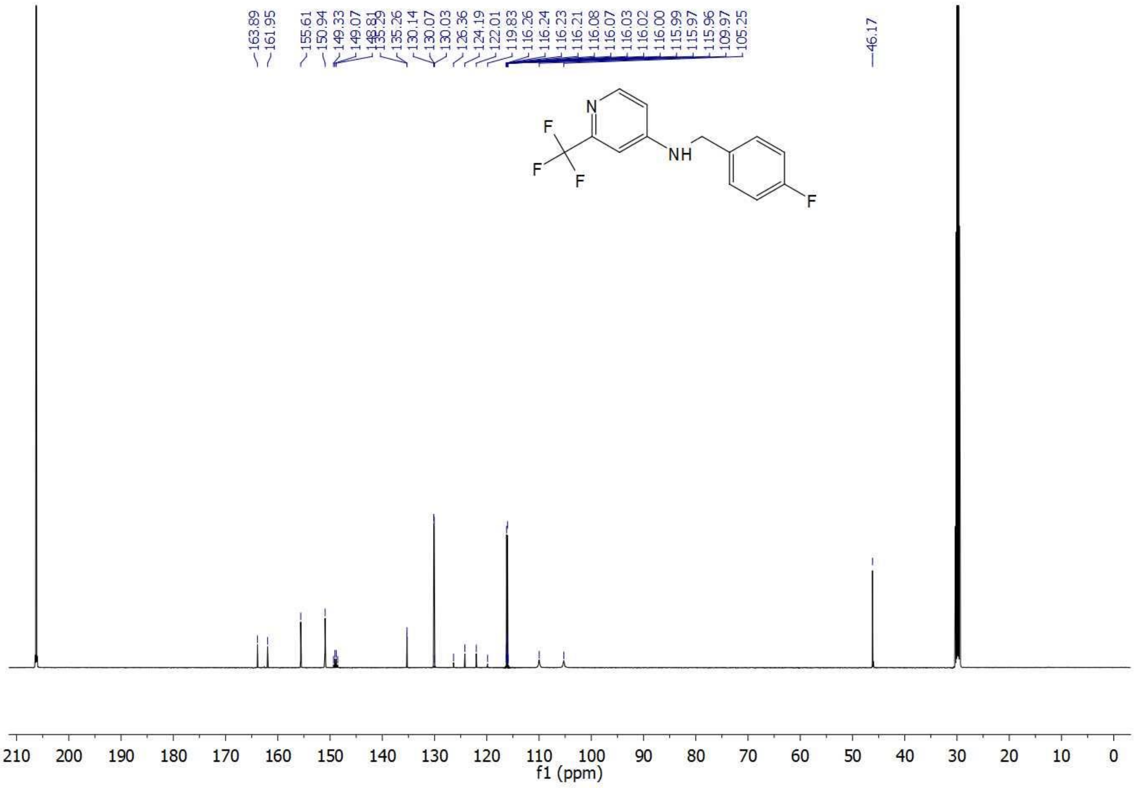

Compound **3**

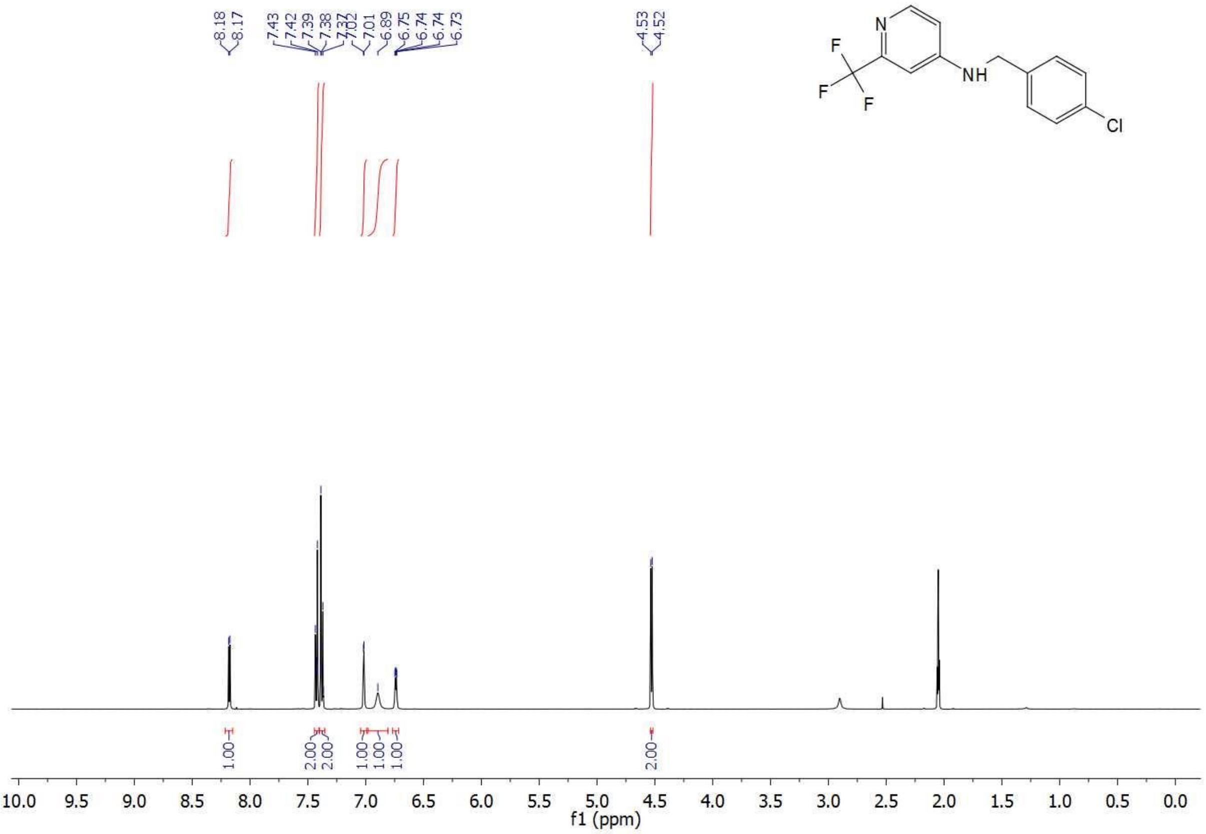

8.18
8.17
7.43
7.42
7.39
7.38
7.37
7.02
7.01
6.89
6.75
6.74
6.74
6.73
4.53
4.52
N
F
F
F
NH
Cl
1.00
2.00
2.00
1.00
1.00
1.00
2.00
10.0 9.5 9.0 8.5 8.0 7.5 7.0 6.5 6.0 5.5 5.0 4.5 4.0 3.5 3.0 2.5 2.0 1.5 1.0 0.5 0.0
f1 (ppm)

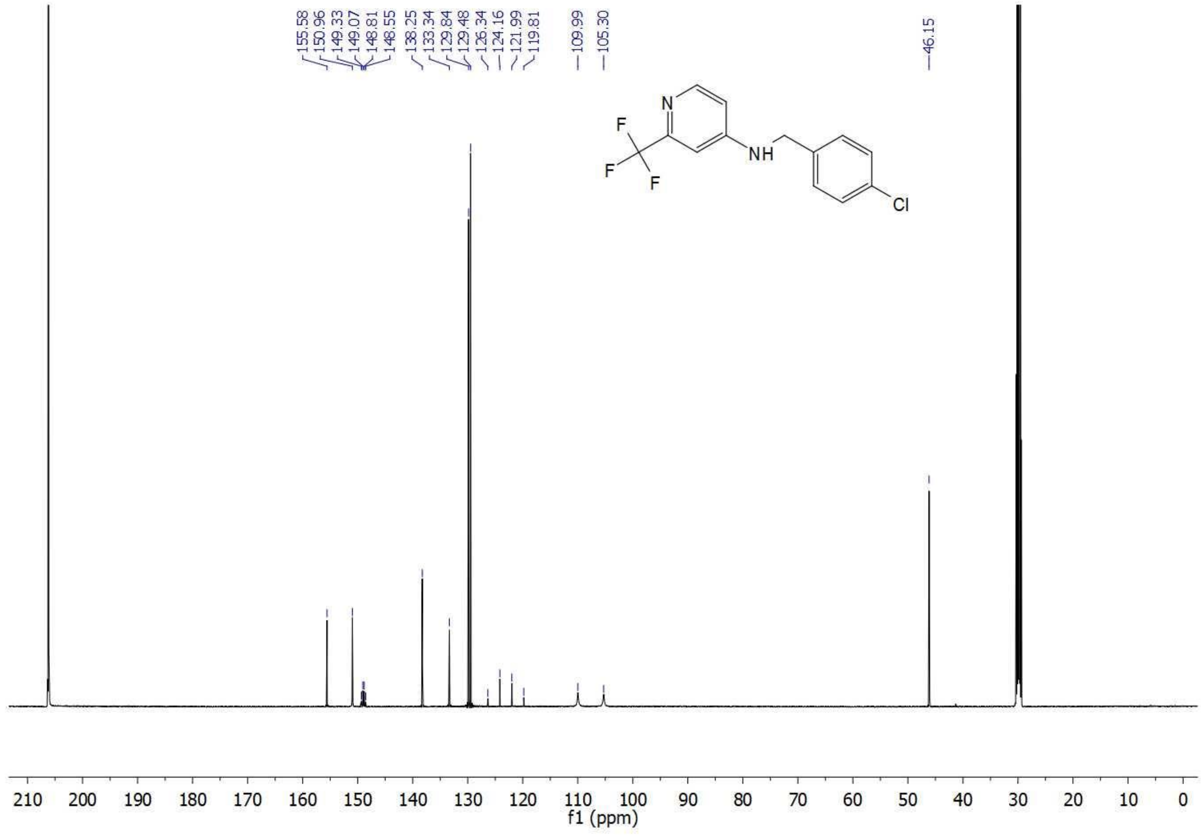

155.58
150.96
149.33
149.07
148.81
148.55
138.25
133.34
129.84
129.48
126.34
124.16
121.99
119.81
109.99
105.30
46.15
N
F
F
F
NH
Cl
210 200 190 180 170 160 150 140 130 120 110 100 90 80 70 60 50 40 30 20 10 0
f1 (ppm)

Compound **4**

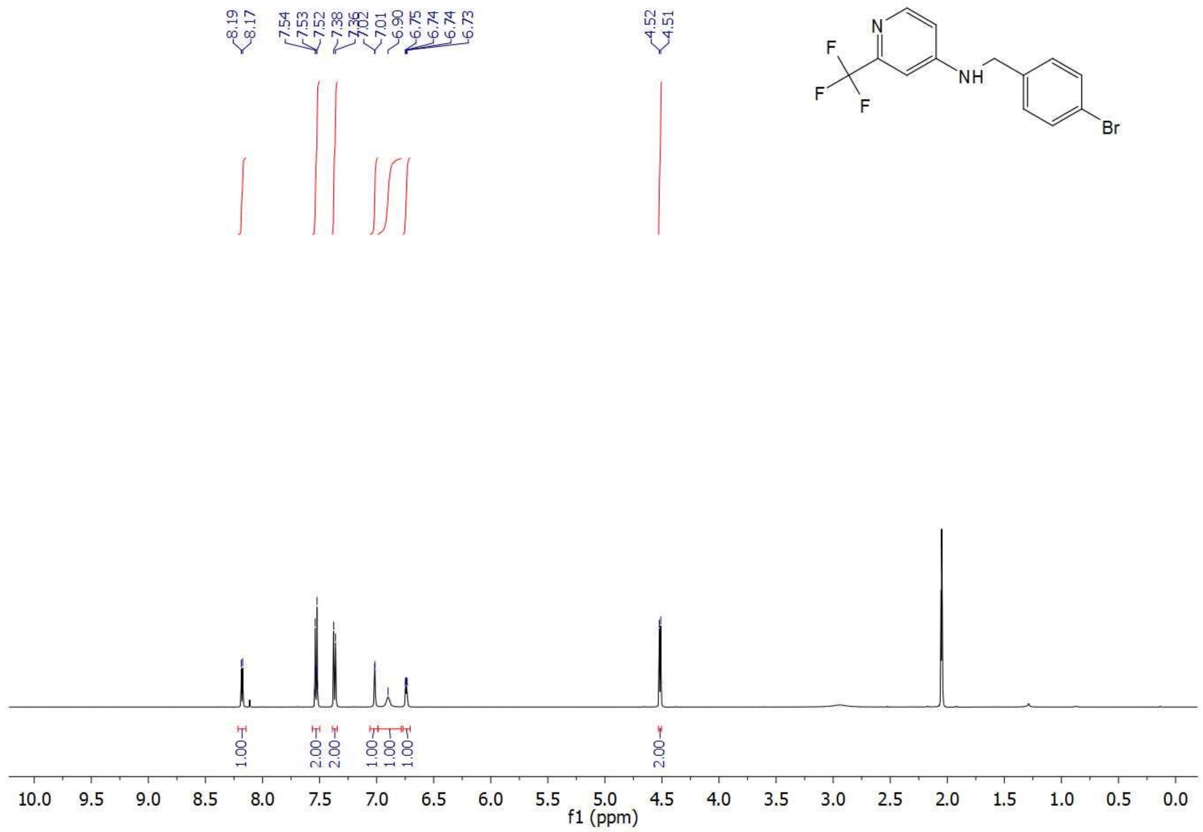
8.19
8.17
7.54
7.53
7.52
7.38
7.36
7.02
7.01
6.90
6.75
6.74
6.74
6.73
4.52
4.51
N
F
F
F
NH
Br
1.00
2.00
2.00
1.00
1.00
1.00
2.00
10.0 9.5 9.0 8.5 8.0 7.5 7.0 6.5 6.0 5.5 5.0 4.5 4.0 3.5 3.0 2.5 2.0 1.5 1.0 0.5 0.0
f1 (ppm)

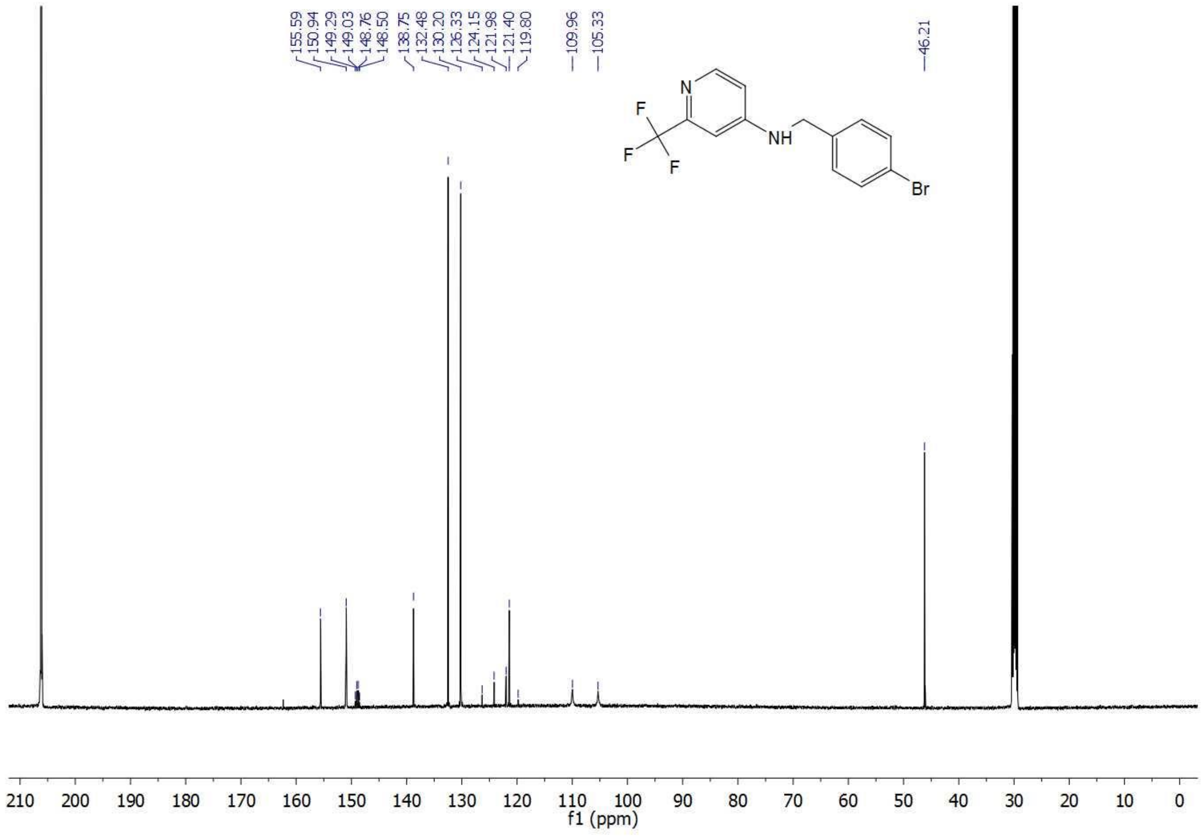
155.59
150.94
149.29
149.03
148.76
148.50
138.75
132.48
130.20
126.33
124.15
121.98
121.40
119.80
109.96
105.33
46.21
N
F
F
F
NH
Br
210 200 190 180 170 160 150 140 130 120 110 100 90 80 70 60 50 40 30 20 10 0
f1 (ppm)

Compound **5**

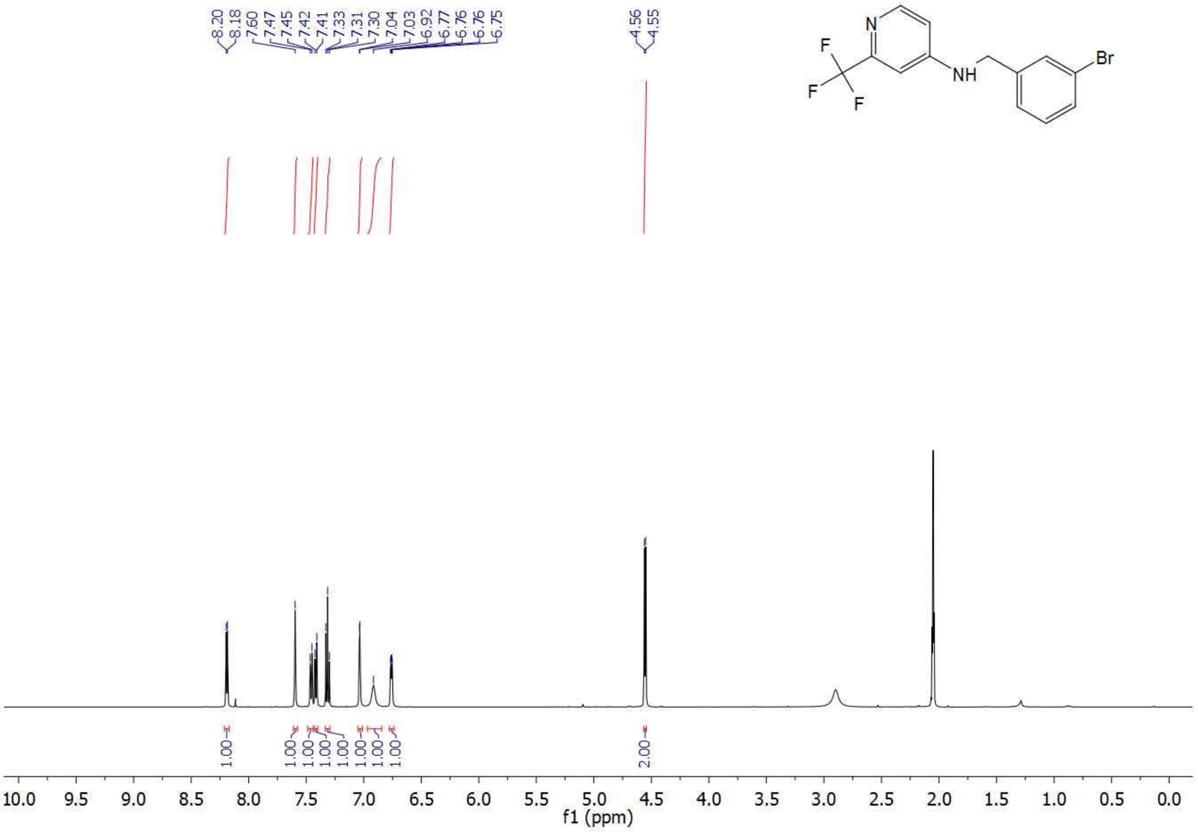
8.20
8.18
7.60
7.47
7.45
7.42
7.41
7.33
7.31
7.30
7.04
7.03
6.92
6.77
6.76
6.76
6.75
4.56
4.55
F
F
F
N
NH
Br
1.00
1.00
1.00
1.00
1.00
1.00
1.00
1.00
2.00
10.0 9.5 9.0 8.5 8.0 7.5 7.0 6.5 6.0 5.5 5.0 4.5 4.0 3.5 3.0 2.5 2.0 1.5 1.0 0.5 0.0
f1 (ppm)

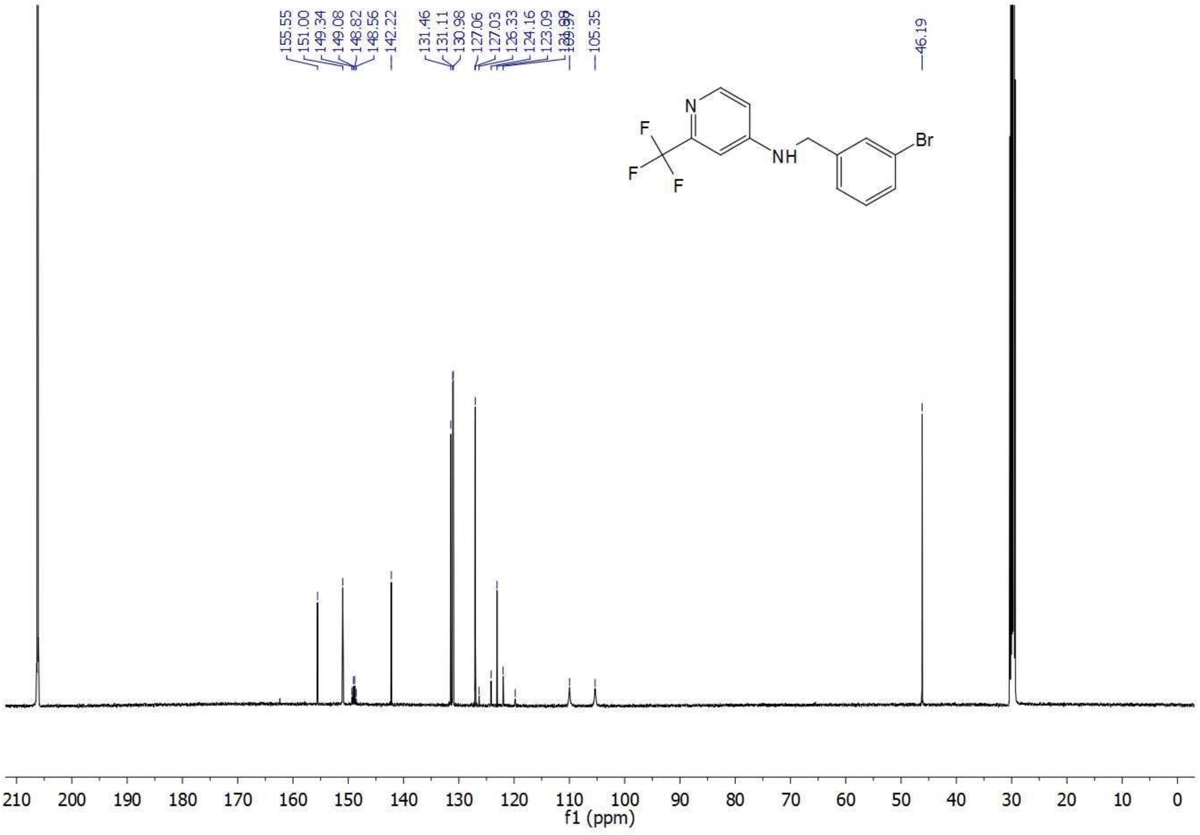
155.55
151.00
149.34
149.08
148.82
148.56
142.22
131.46
131.11
130.98
127.06
127.03
126.33
124.16
123.09
105.35
46.19
F
F
F
N
NH
Br
210 200 190 180 170 160 150 140 130 120 110 100 90 80 70 60 50 40 30 20 10 0
f1 (ppm)

Compound **6**

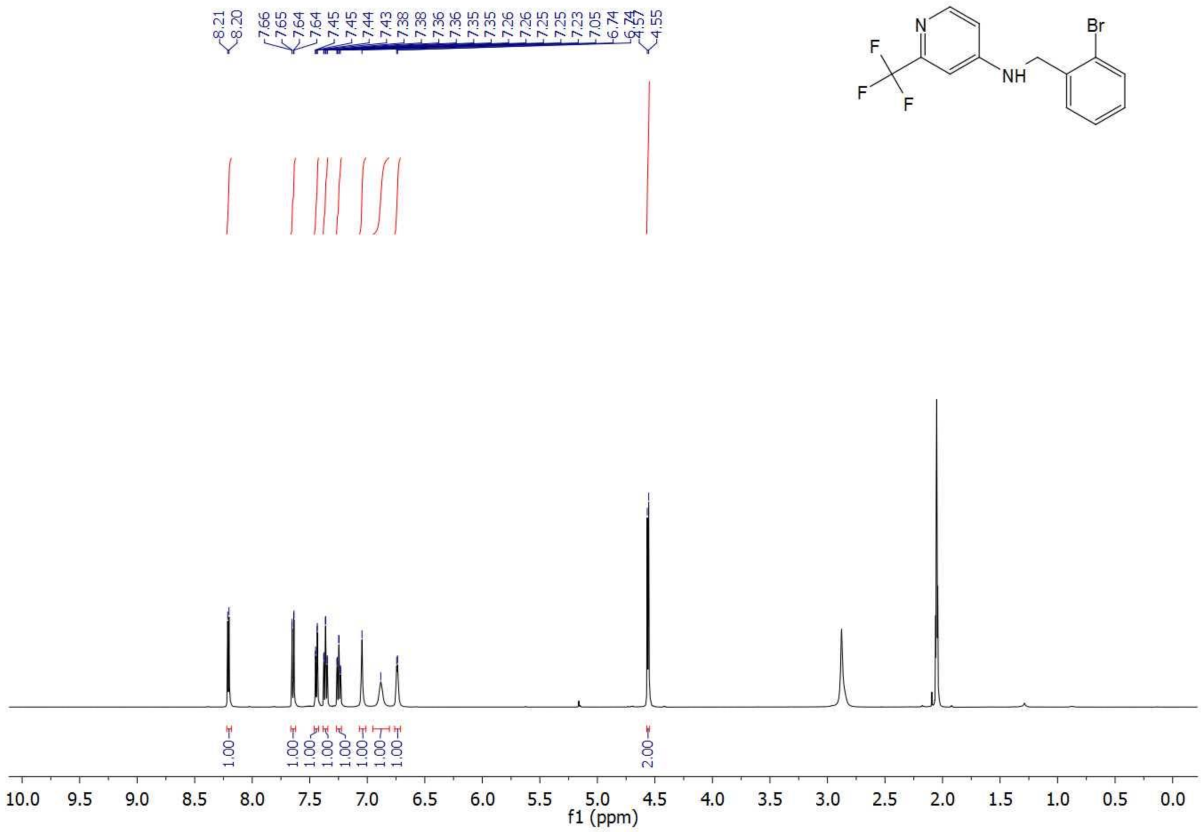

8.21
8.20
7.66
7.65
7.64
7.45
7.45
7.44
7.43
7.38
7.38
7.36
7.36
7.35
7.35
7.26
7.26
7.25
7.25
7.23
7.05
6.74
4.57
4.55
1.00
1.00
1.00
1.00
1.00
1.00
1.00
1.00
2.00
f1 (ppm)

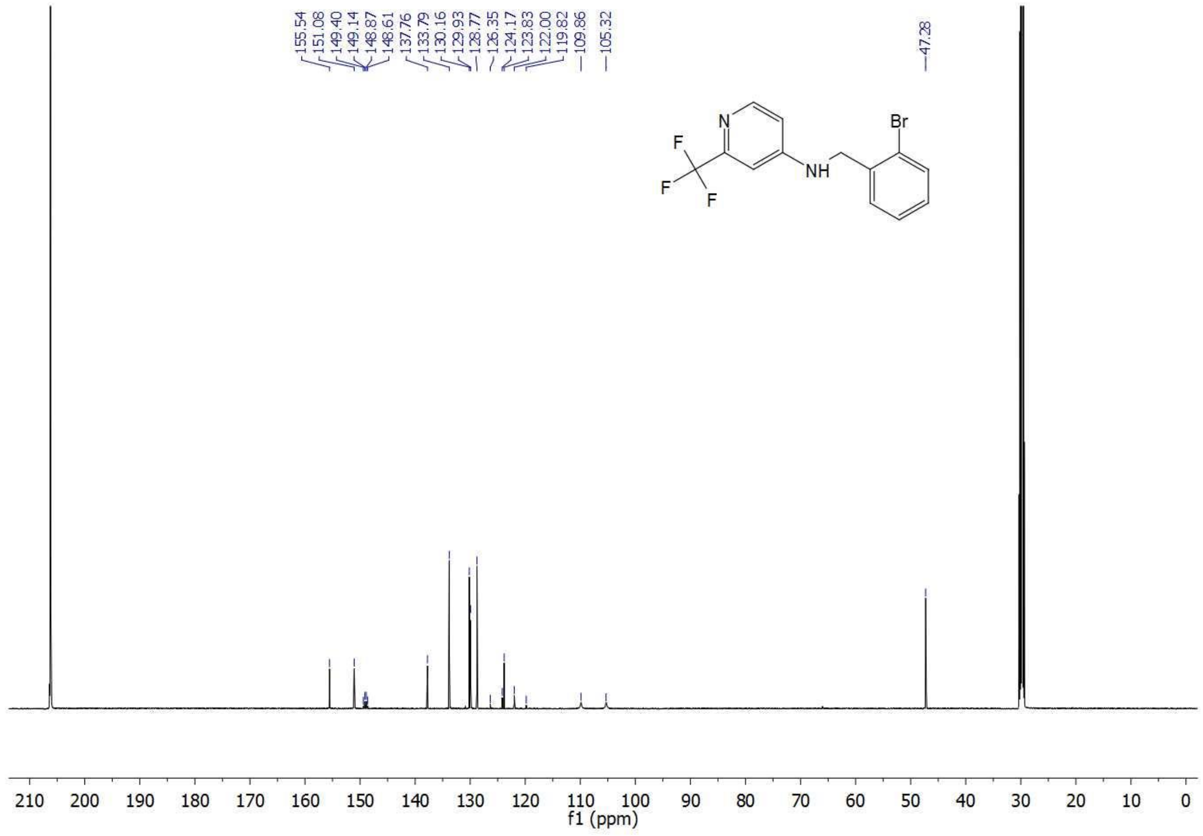

155.54
151.08
149.40
149.14
148.87
148.61
137.76
133.79
130.16
129.93
128.77
126.35
124.17
123.83
122.00
119.82
109.86
105.32
47.28
f1 (ppm)

Compound **7**

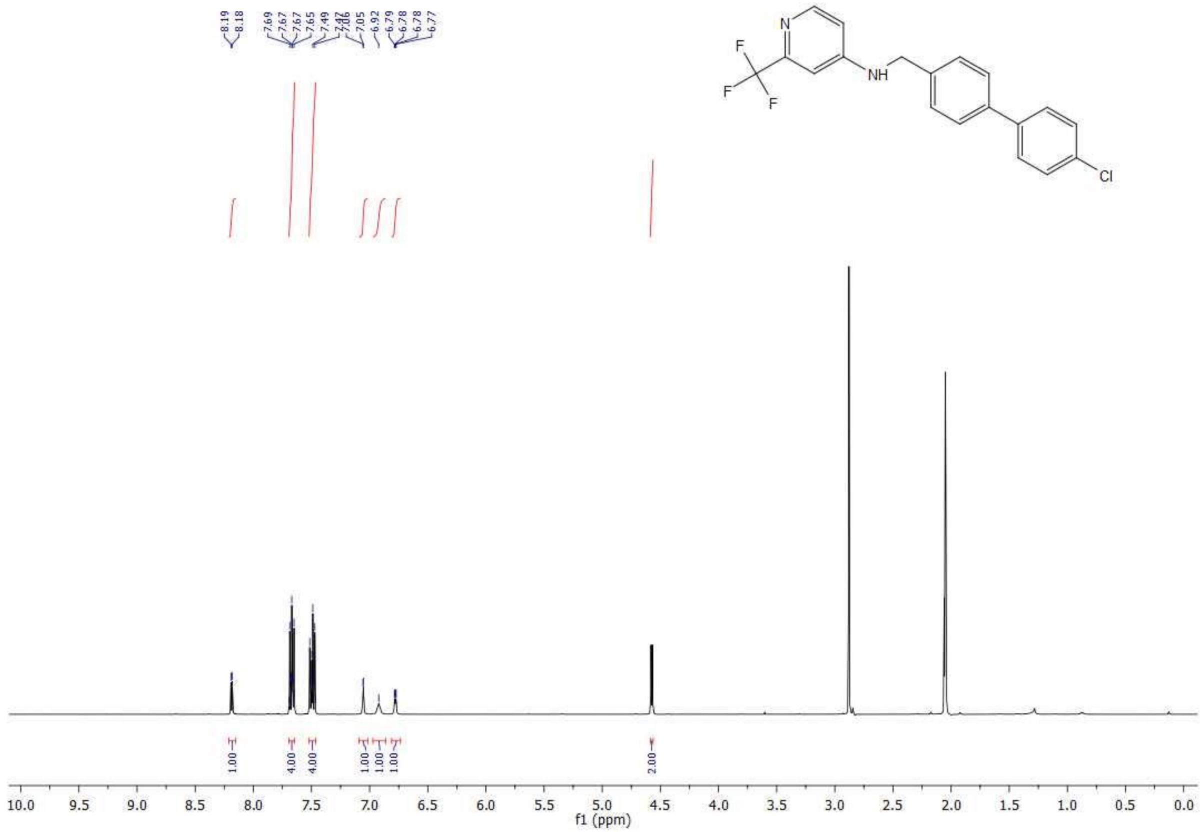

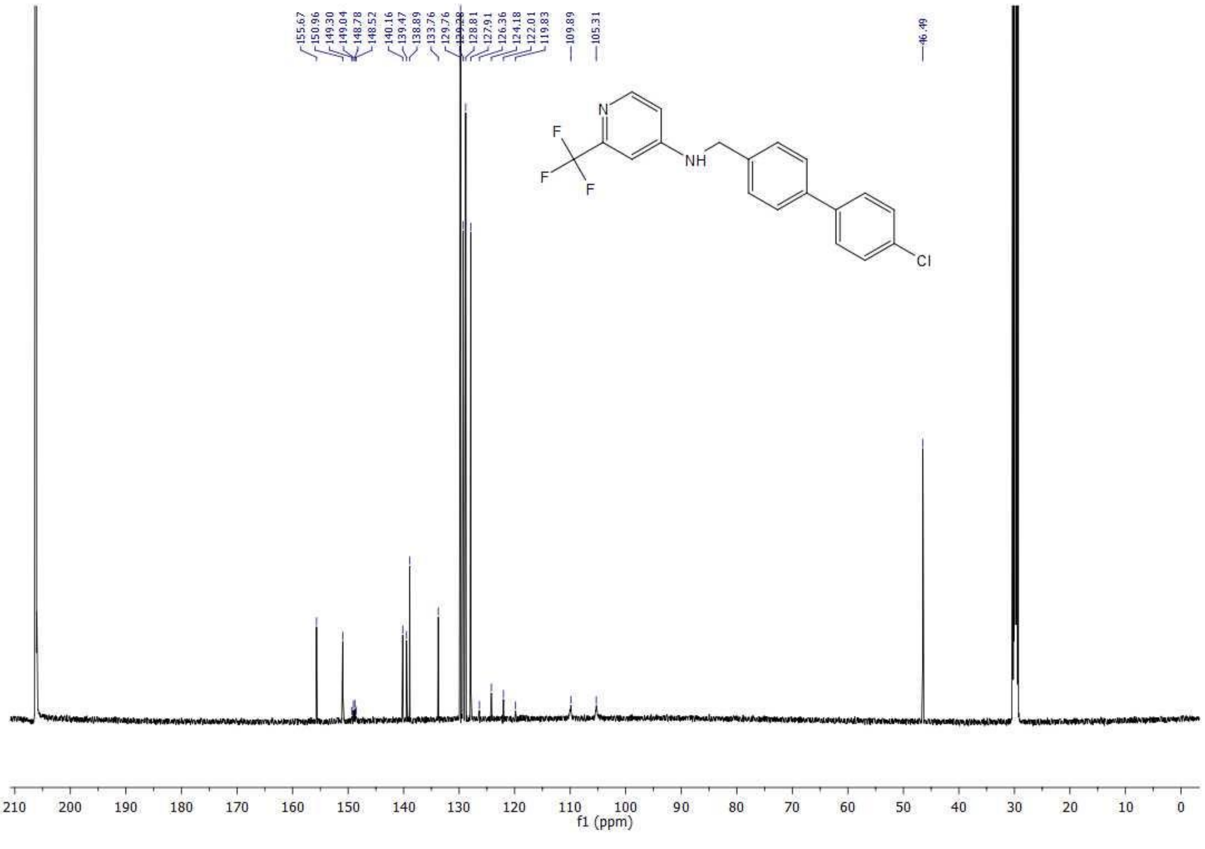

Compound **8**

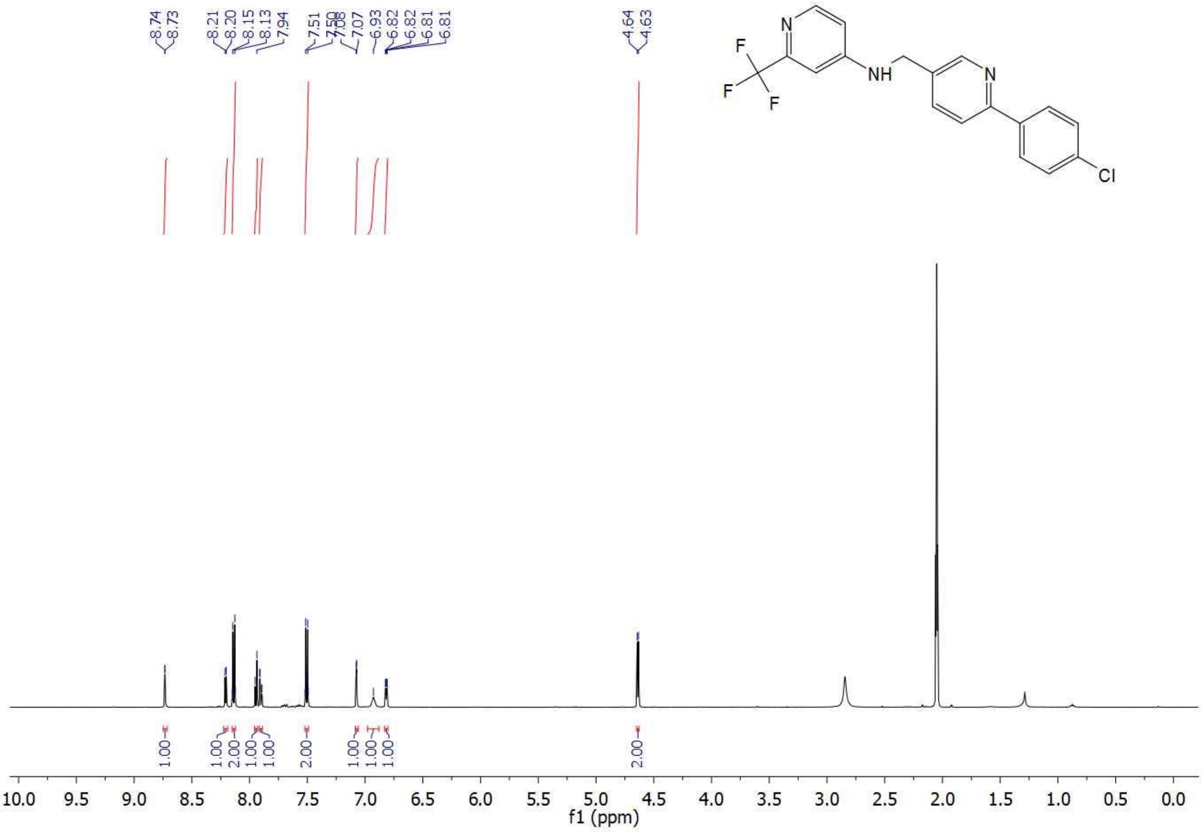

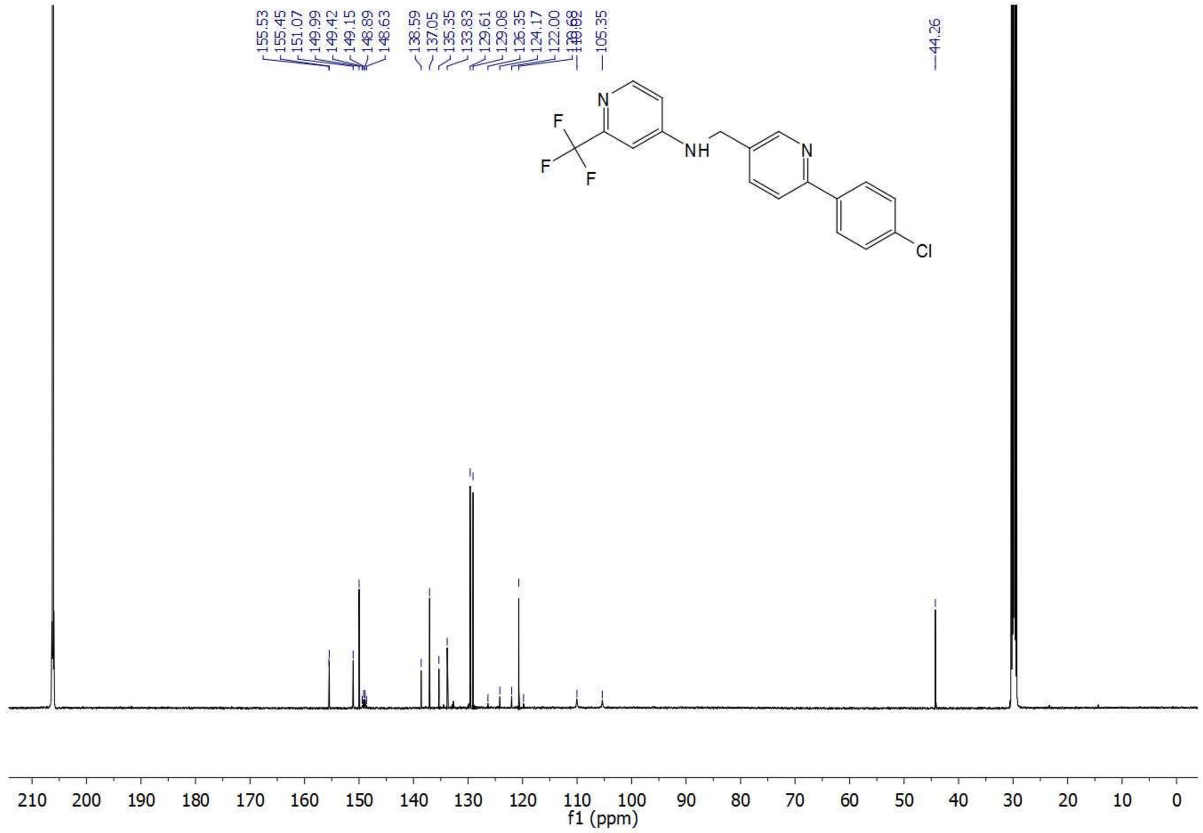

Compound **9**

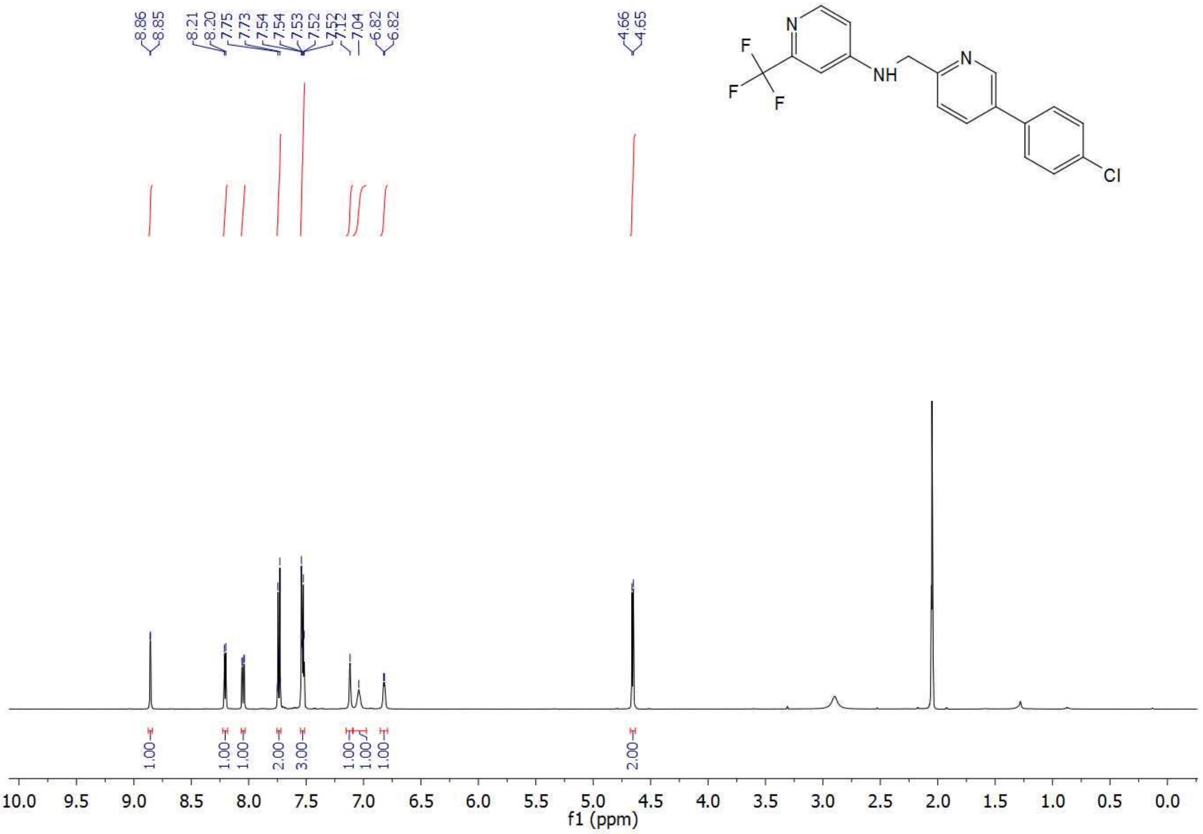

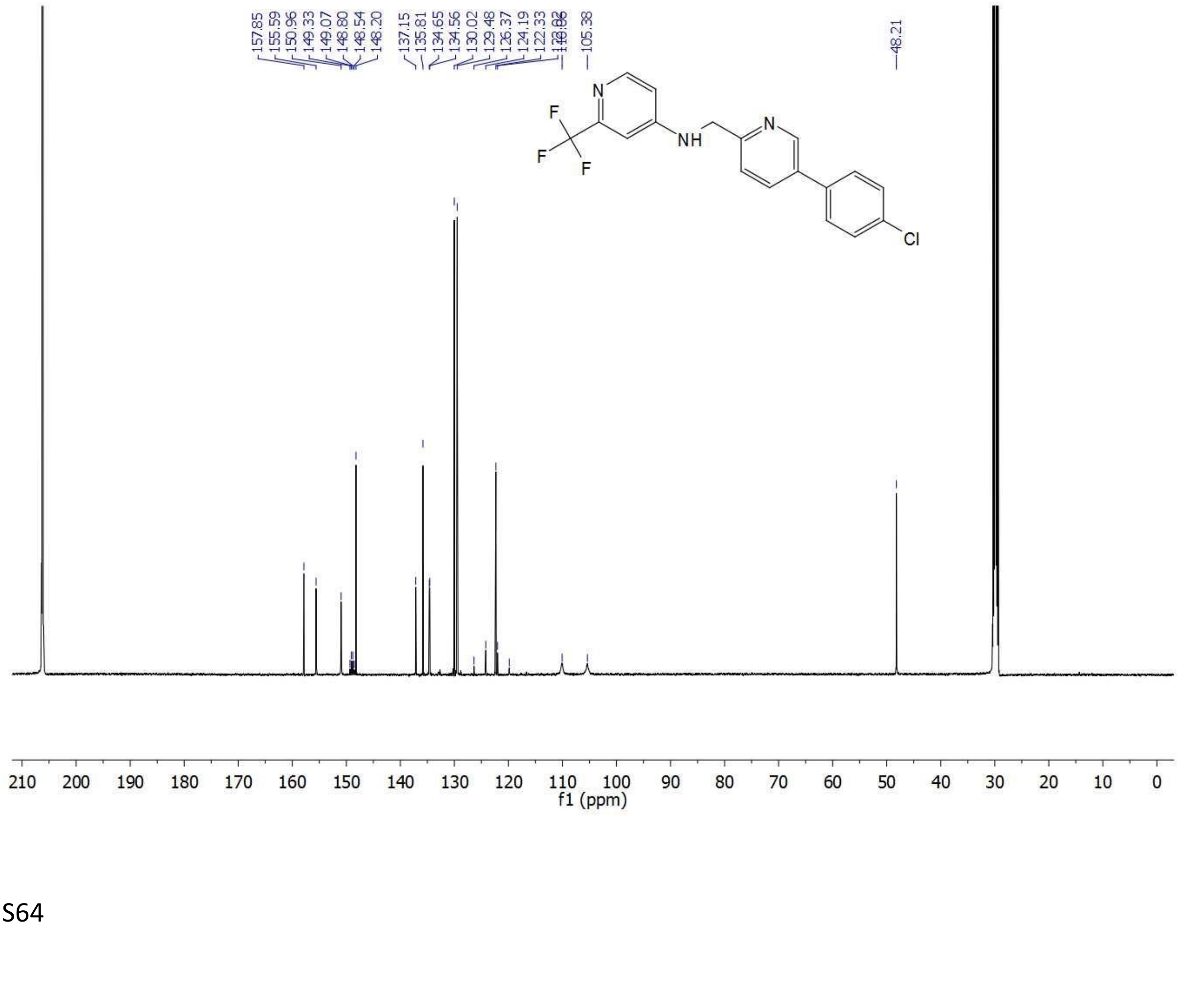

Compound **10**

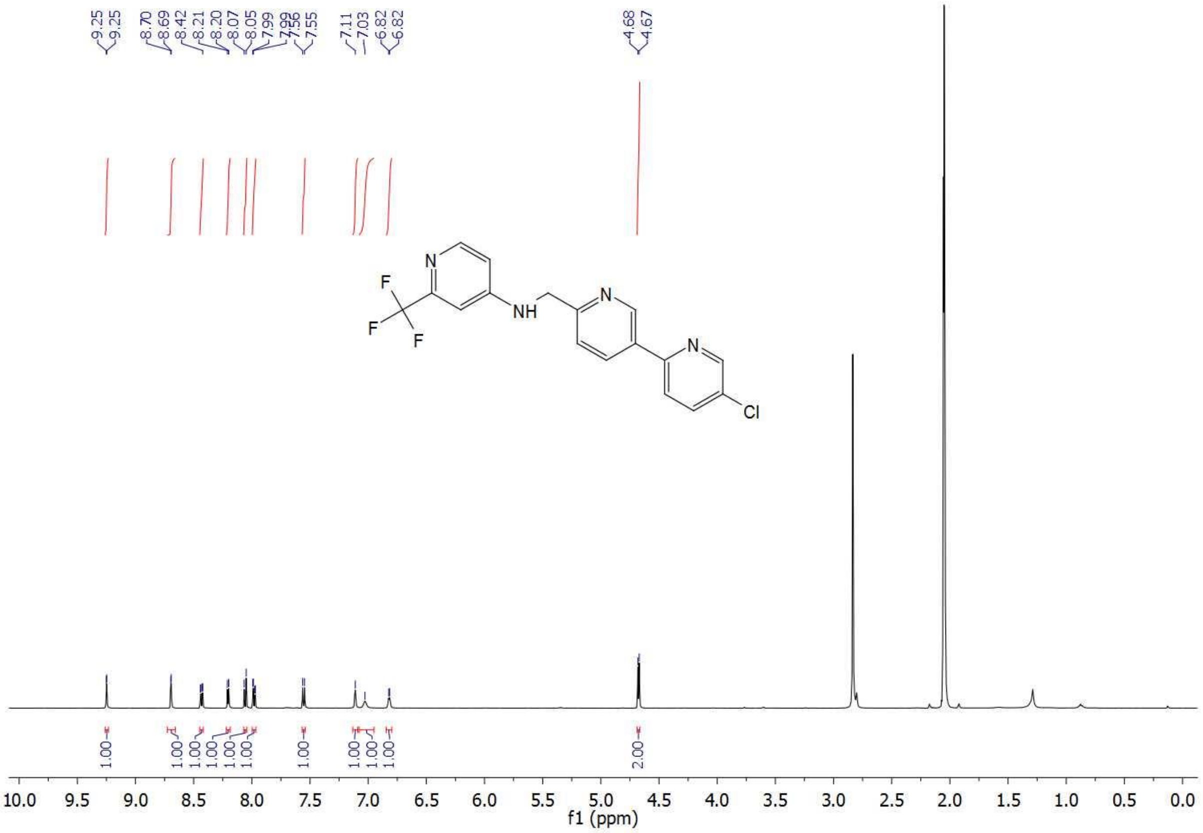
9.25
9.25
8.70
8.69
8.42
8.21
8.20
8.07
8.05
7.99
7.56
7.55
7.11
7.03
6.82
6.82
4.68
4.67
1.00
1.00
1.00
1.00
1.00
1.00
1.00
1.00
1.00
1.00
2.00
f1 (ppm)

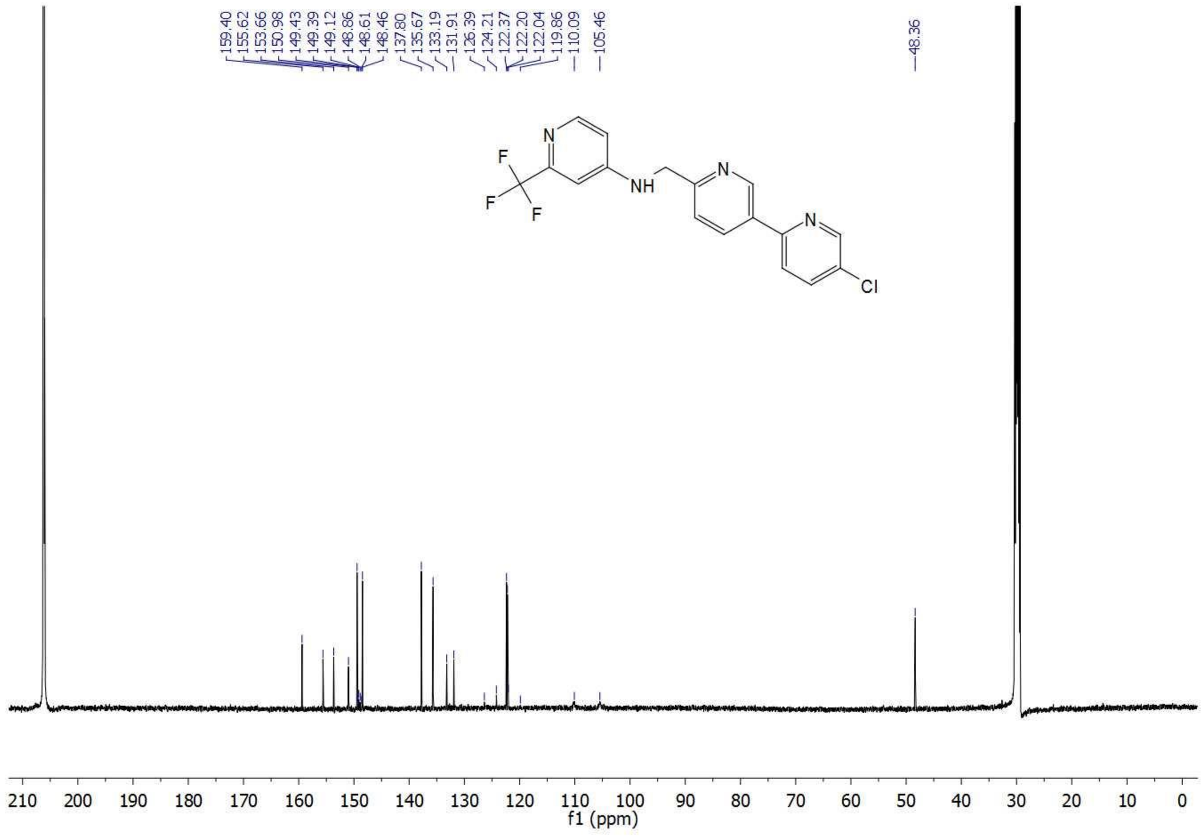
159.40
155.62
153.66
150.98
149.43
149.39
149.12
148.86
148.61
148.46
137.80
135.67
133.19
131.91
126.39
124.21
122.37
122.20
122.04
119.86
110.09
105.46
48.36
f1 (ppm)

Compound **11**

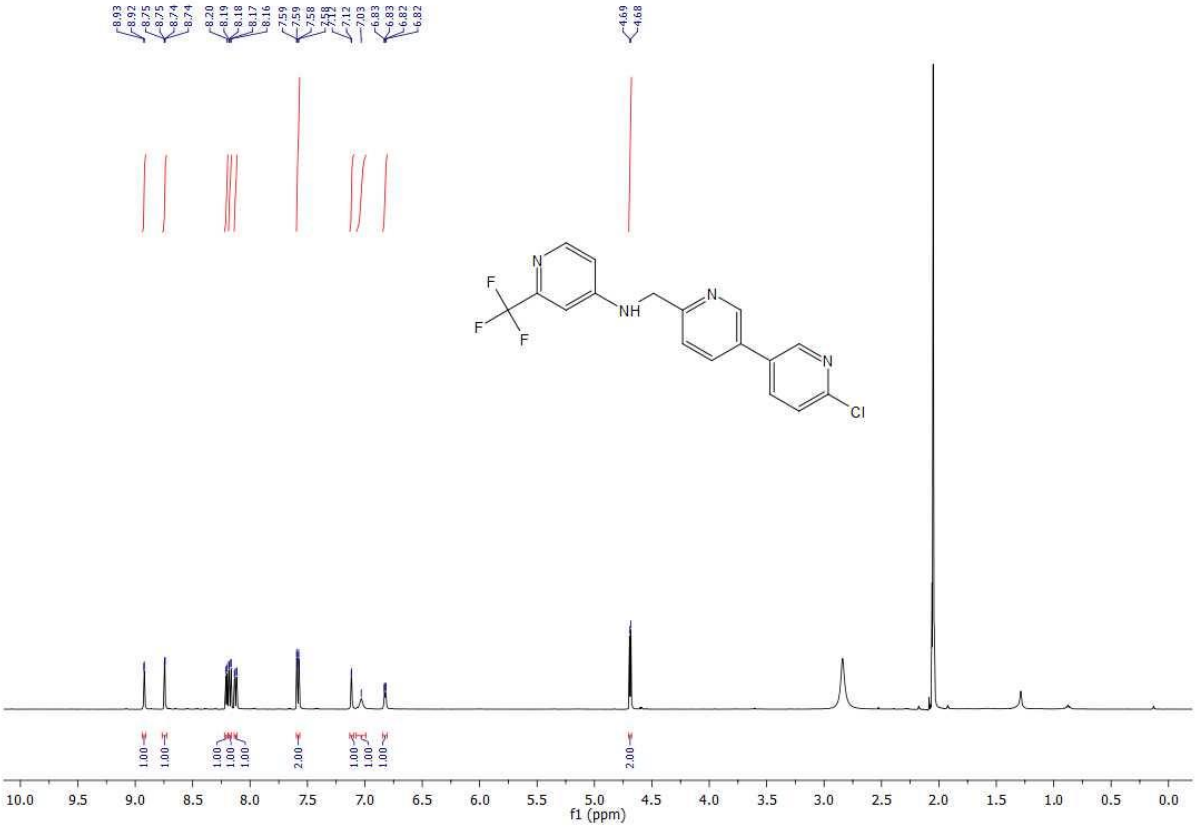

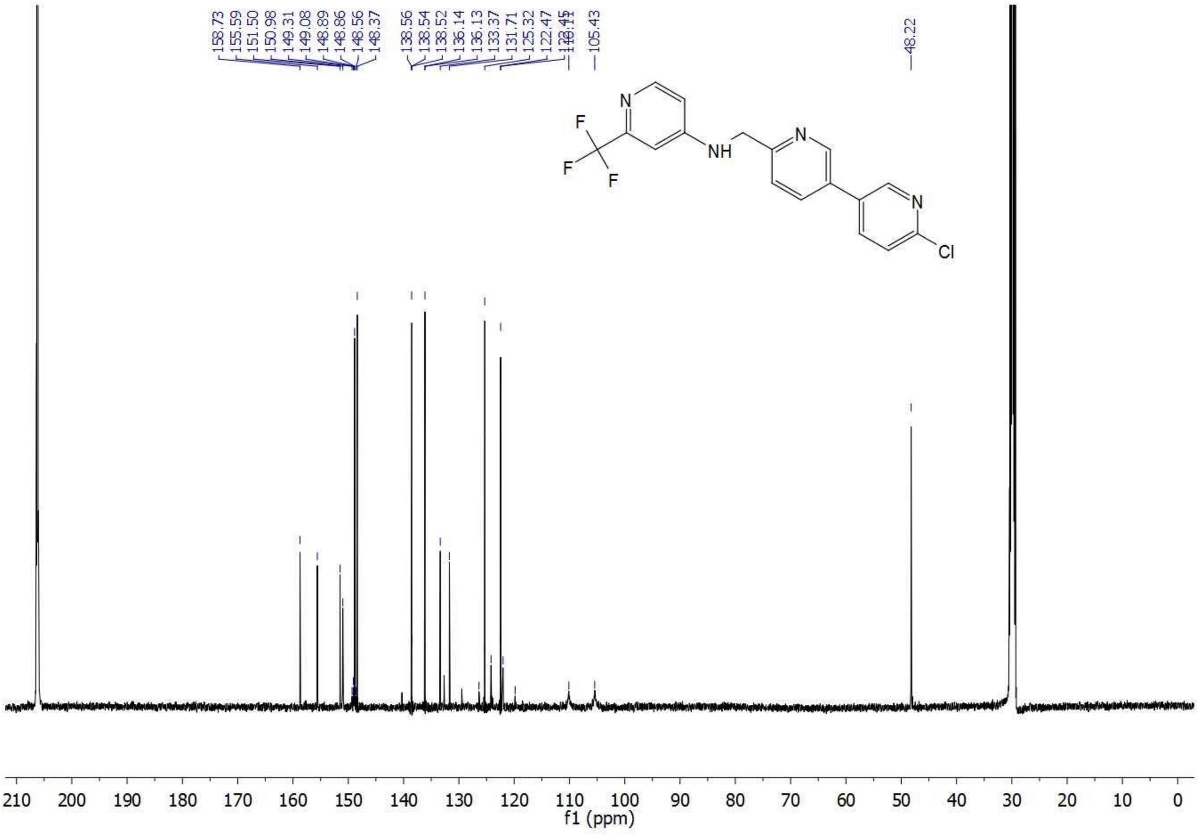

Compound **12**

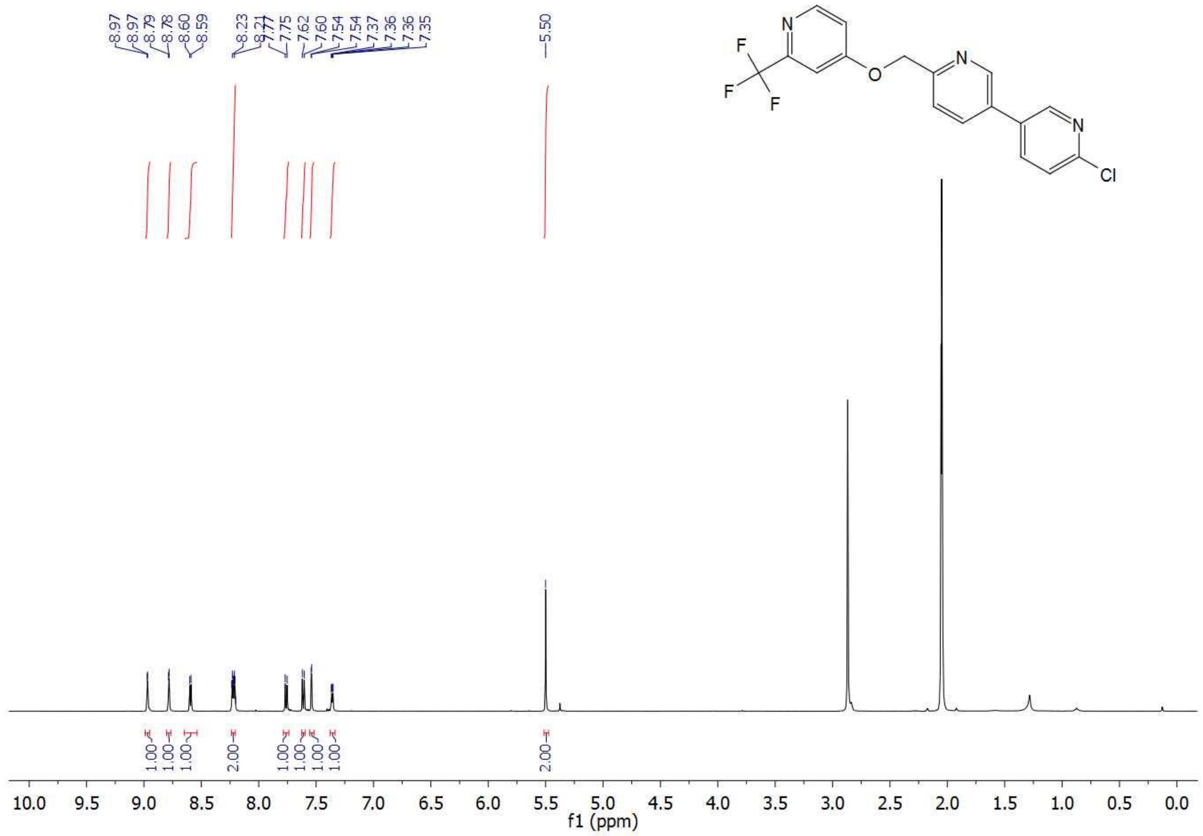
8.97
8.97
8.79
8.78
8.60
8.59
8.23
8.21
7.77
7.75
7.62
7.60
7.54
7.54
7.37
7.36
7.36
7.35
5.50
F
F
F
N
O
N
N
Cl
1.00
1.00
1.00
2.00
1.00
1.00
1.00
1.00
2.00
10.0 9.5 9.0 8.5 8.0 7.5 7.0 6.5 6.0 5.5 5.0 4.5 4.0 3.5 3.0 2.5 2.0 1.5 1.0 0.5 0.0
f1 (ppm)

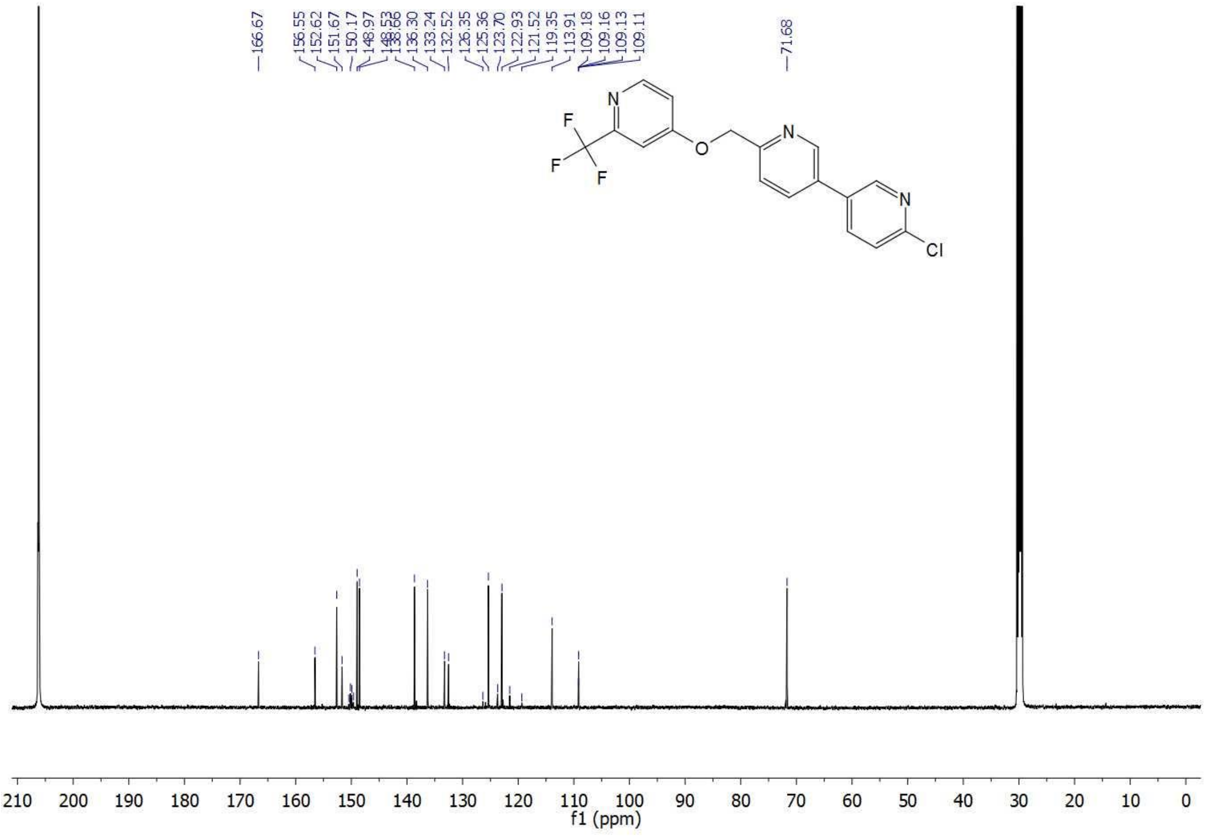
166.67
156.55
152.62
151.67
150.17
148.97
148.53
138.66
136.30
133.24
132.52
126.35
125.36
123.70
122.93
121.52
119.35
113.91
109.18
109.16
109.13
109.11
71.68
F
F
F
N
O
N
N
Cl
210 200 190 180 170 160 150 140 130 120 110 100 90 80 70 60 50 40 30 20 10 0
f1 (ppm)

Compound **13**

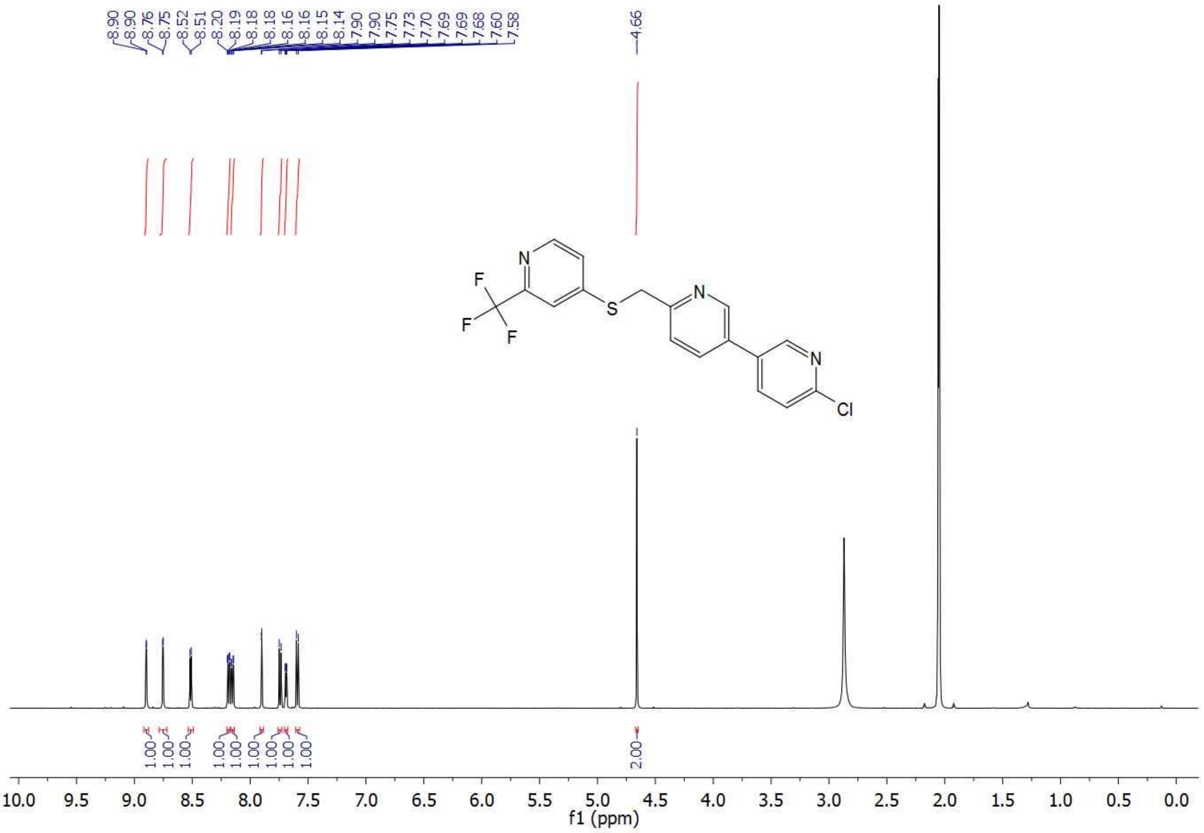
8.90
8.90
8.76
8.75
8.52
8.51
8.20
8.19
8.18
8.18
8.16
8.16
8.15
8.14
7.90
7.90
7.75
7.73
7.70
7.69
7.69
7.68
7.60
7.58
4.66
1.00
1.00
1.00
1.00
1.00
1.00
1.00
1.00
2.00
F
F
F
N
S
N
N
Cl
10.0 9.5 9.0 8.5 8.0 7.5 7.0 6.5 6.0 5.5 5.0 4.5 4.0 3.5 3.0 2.5 2.0 1.5 1.0 0.5 0.0
f1 (ppm)

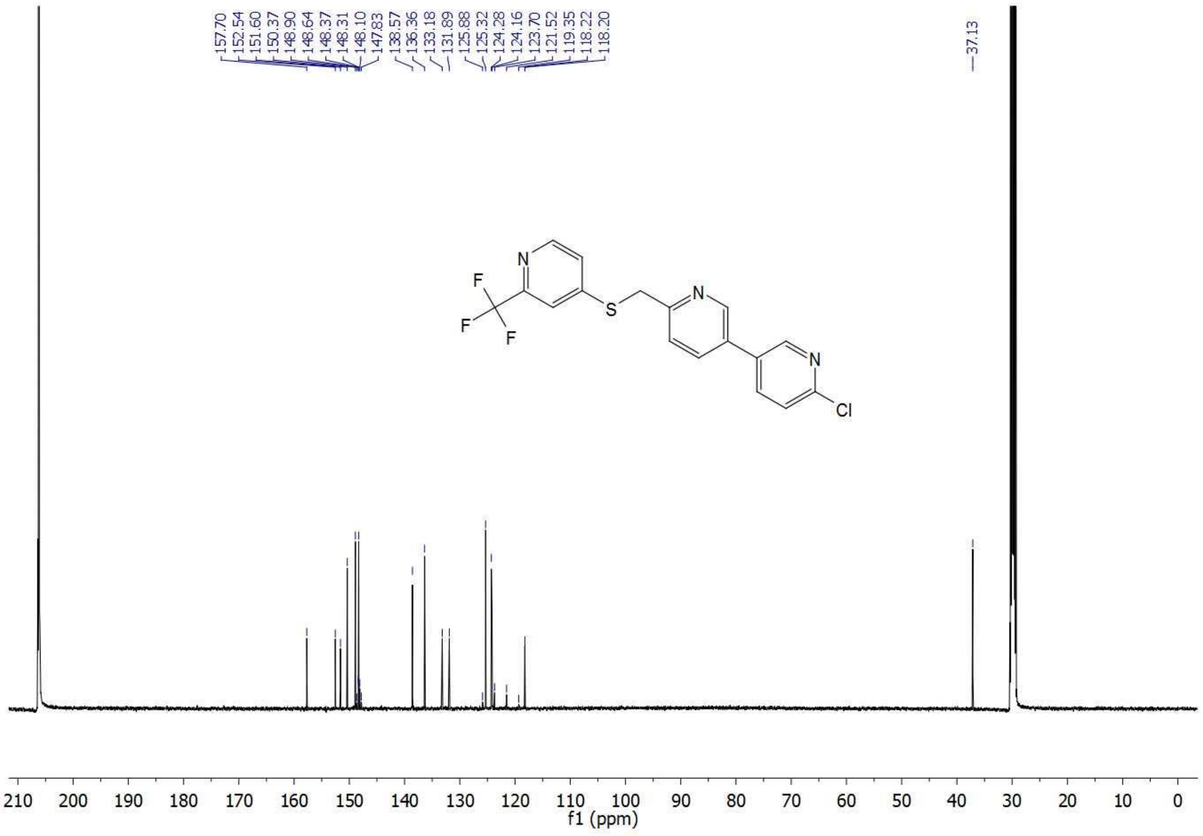
157.70
152.54
151.60
150.37
148.90
148.64
148.37
148.31
148.10
147.83
138.57
136.36
133.18
131.89
125.88
125.32
124.28
124.16
123.70
121.52
119.35
118.22
118.20
37.13
F
F
F
N
S
N
N
Cl
210 200 190 180 170 160 150 140 130 120 110 100 90 80 70 60 50 40 30 20 10 0
f1 (ppm)

Compound **14**

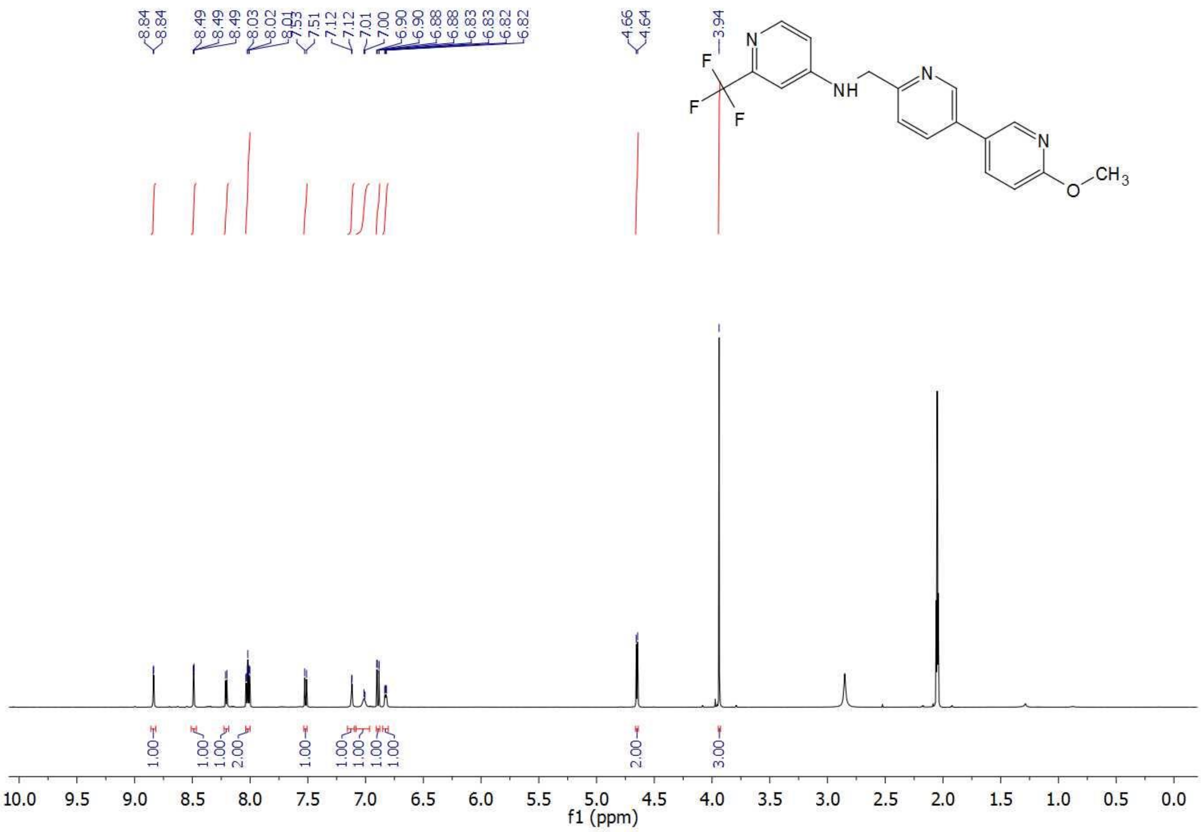

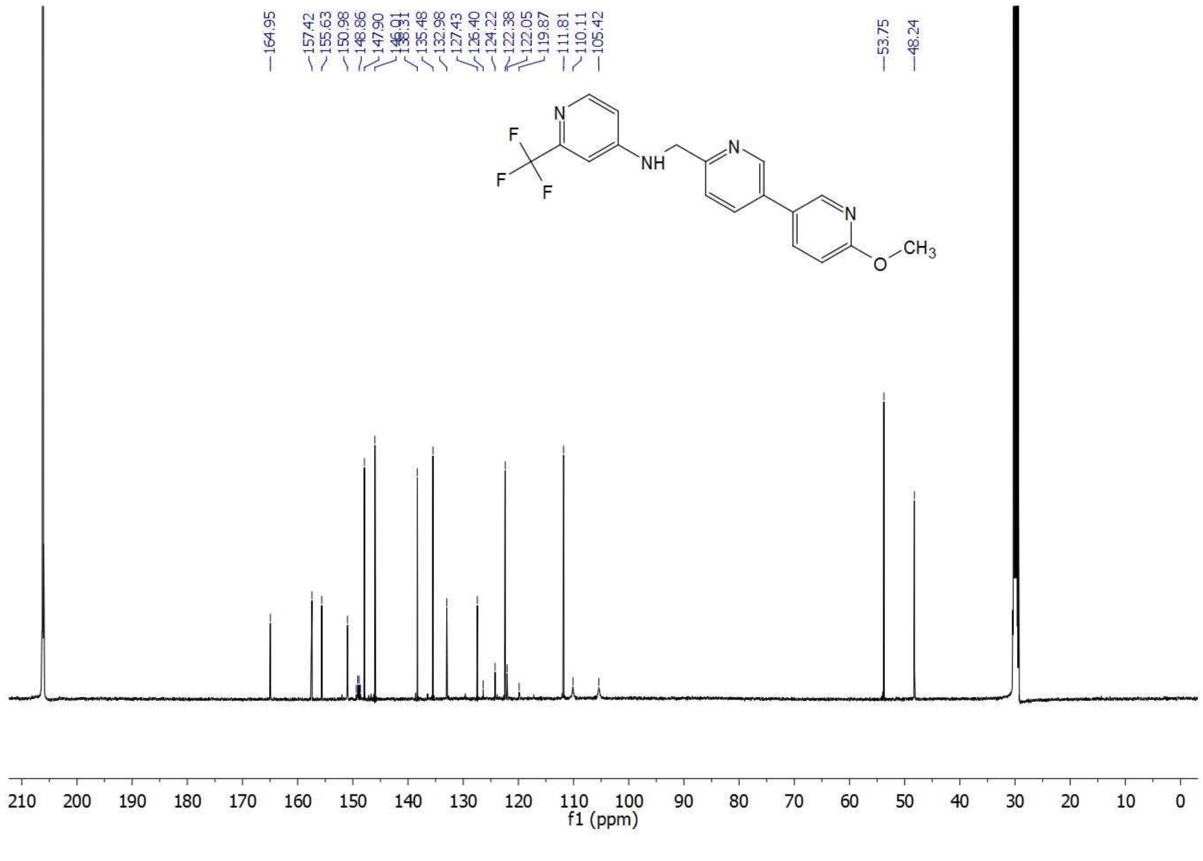

Compound **15**

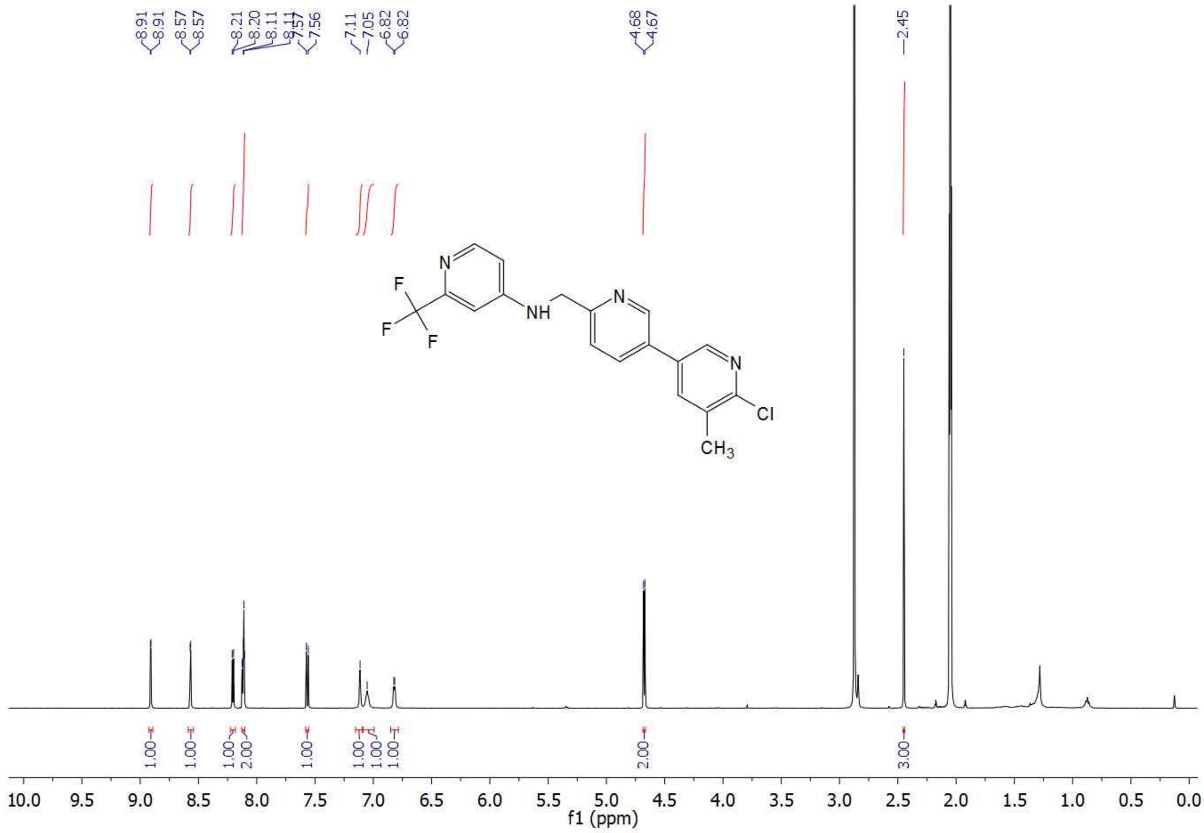

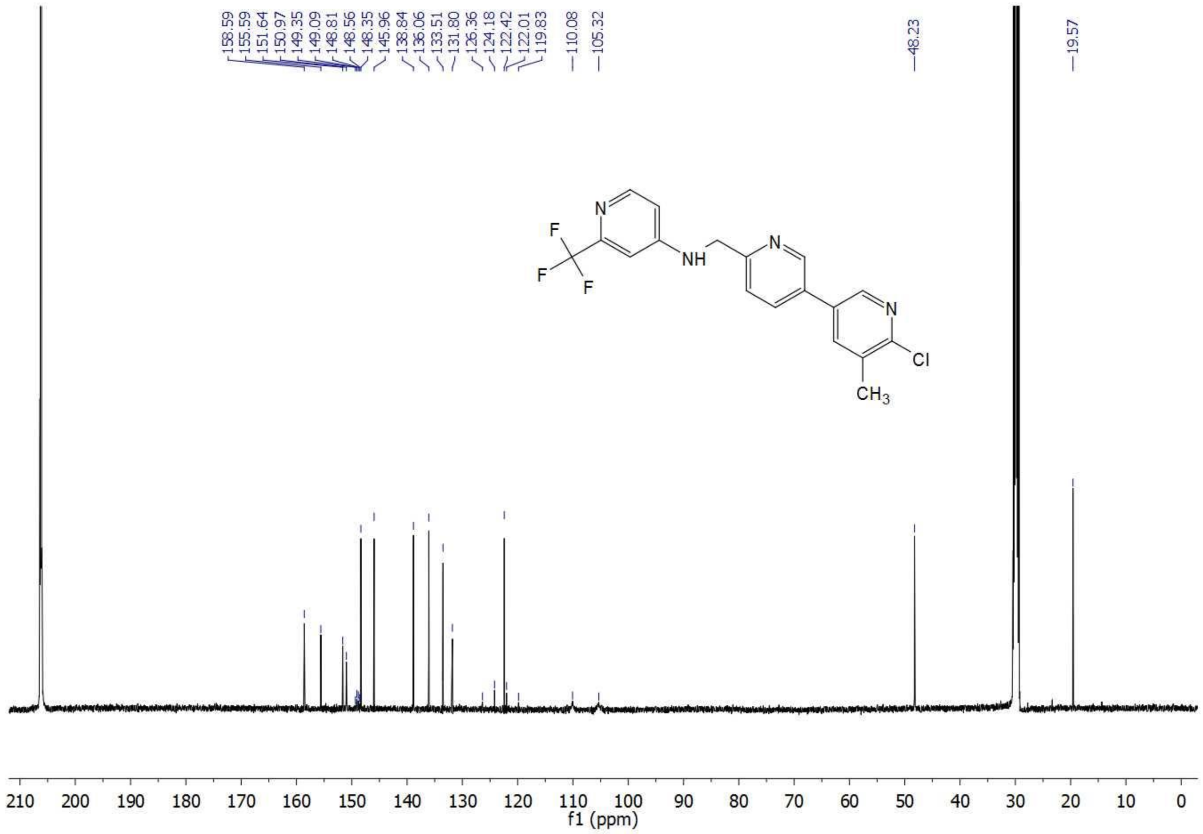

Compound **16**

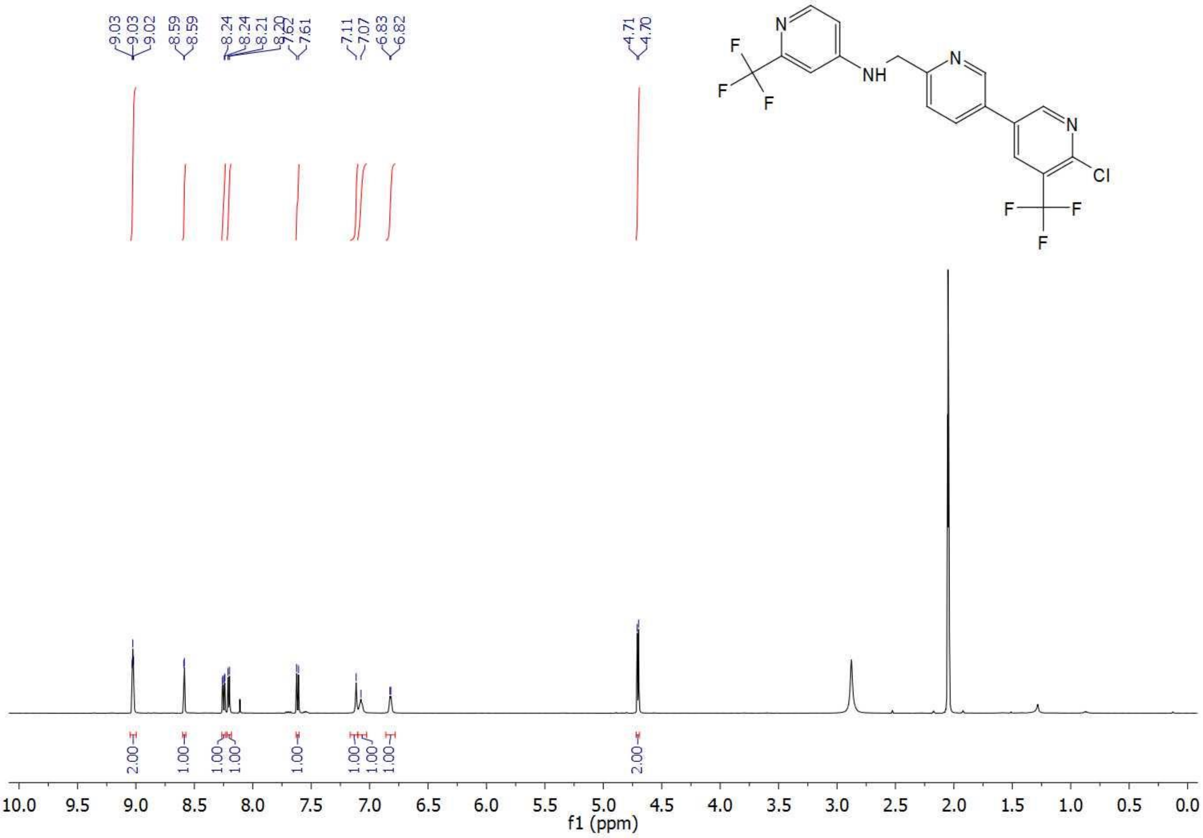
9.03
9.03
9.02
8.59
8.59
8.24
8.24
8.21
7.61
7.11
7.07
6.83
6.82
4.71
4.70
NH
Cl
2.00
1.00
1.00
1.00
1.00
1.00
1.00
1.00
2.00
f1 (ppm)

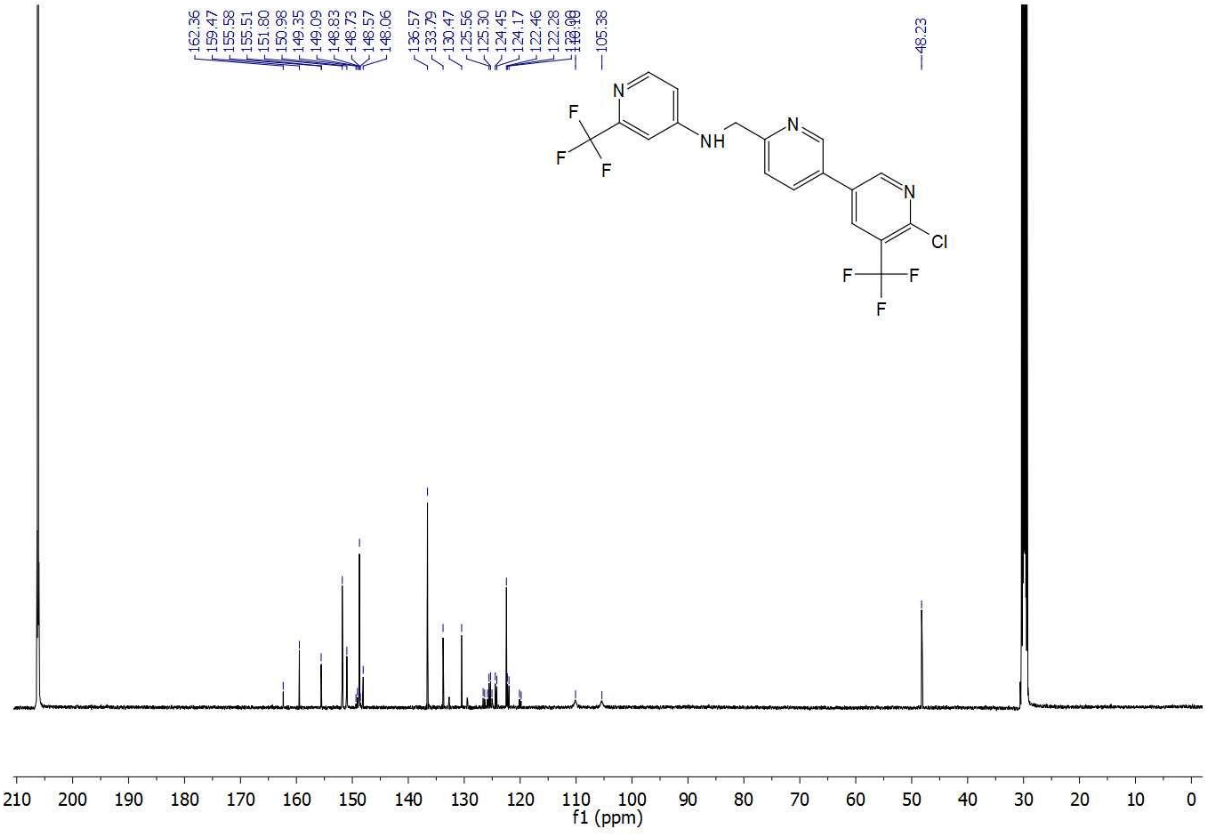
162.36
159.47
155.58
155.51
151.80
150.98
149.35
149.09
148.83
148.73
148.57
148.06
136.57
133.79
130.47
125.56
125.30
124.45
124.17
122.46
122.28
105.38
48.23
NH
Cl
f1 (ppm)

Compound **17**

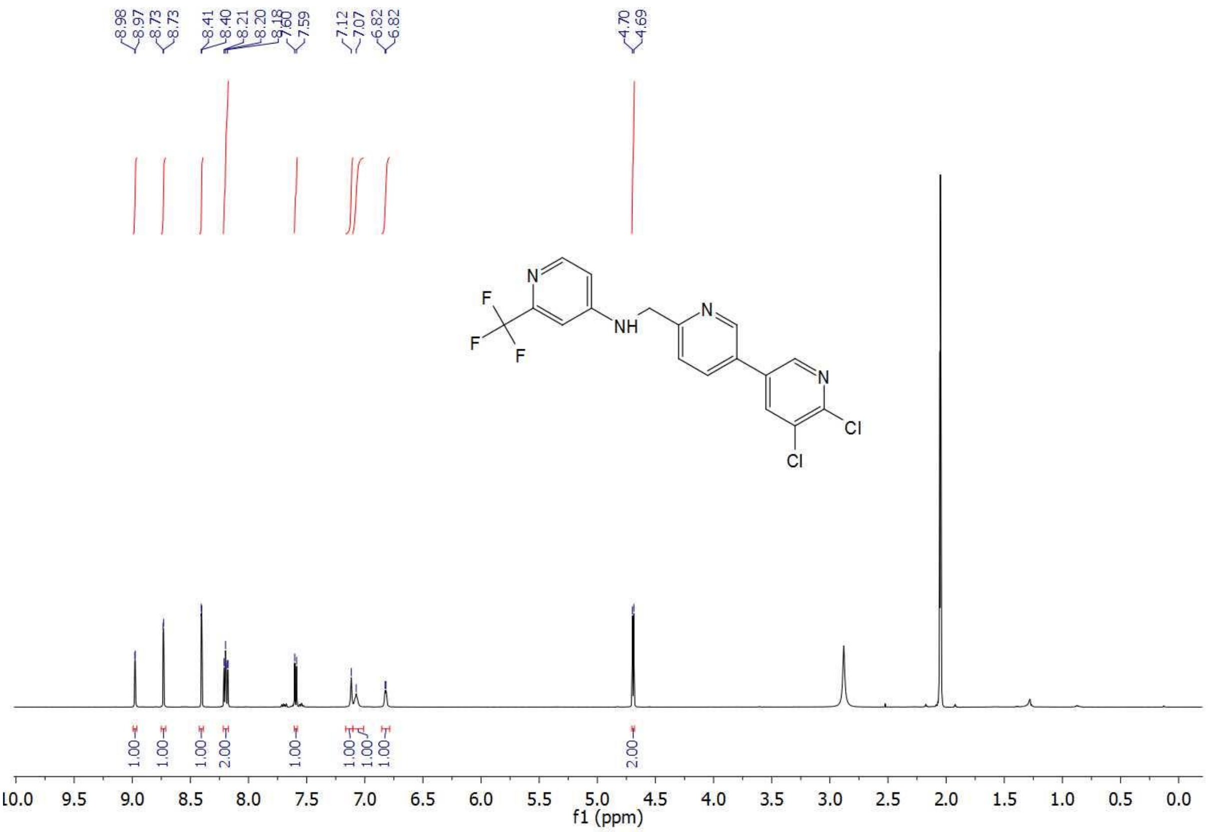
8.98
8.97
8.73
8.73
8.41
8.40
8.21
8.20
8.18
7.60
7.59
7.12
7.07
6.82
6.82
4.70
4.69
N
NH
F
F
F
N
N
Cl
Cl
1.00
1.00
1.00
2.00
1.00
1.00
1.00
1.00
2.00
f1 (ppm)

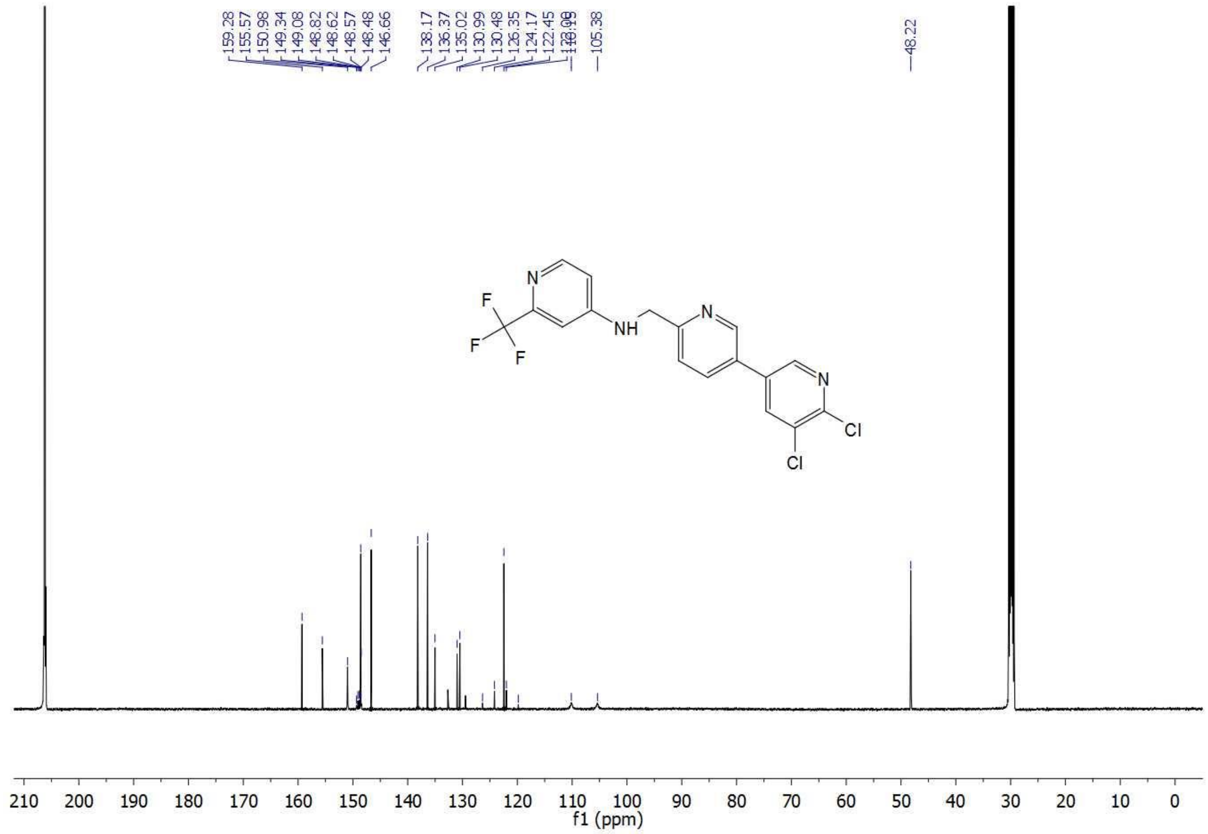
159.28
155.57
150.98
149.34
149.08
148.82
148.62
148.57
148.48
146.66
138.17
136.37
135.02
130.99
130.48
126.35
124.17
122.45
105.38
48.22
N
NH
F
F
F
N
N
Cl
Cl
f1 (ppm)

Compound **18**

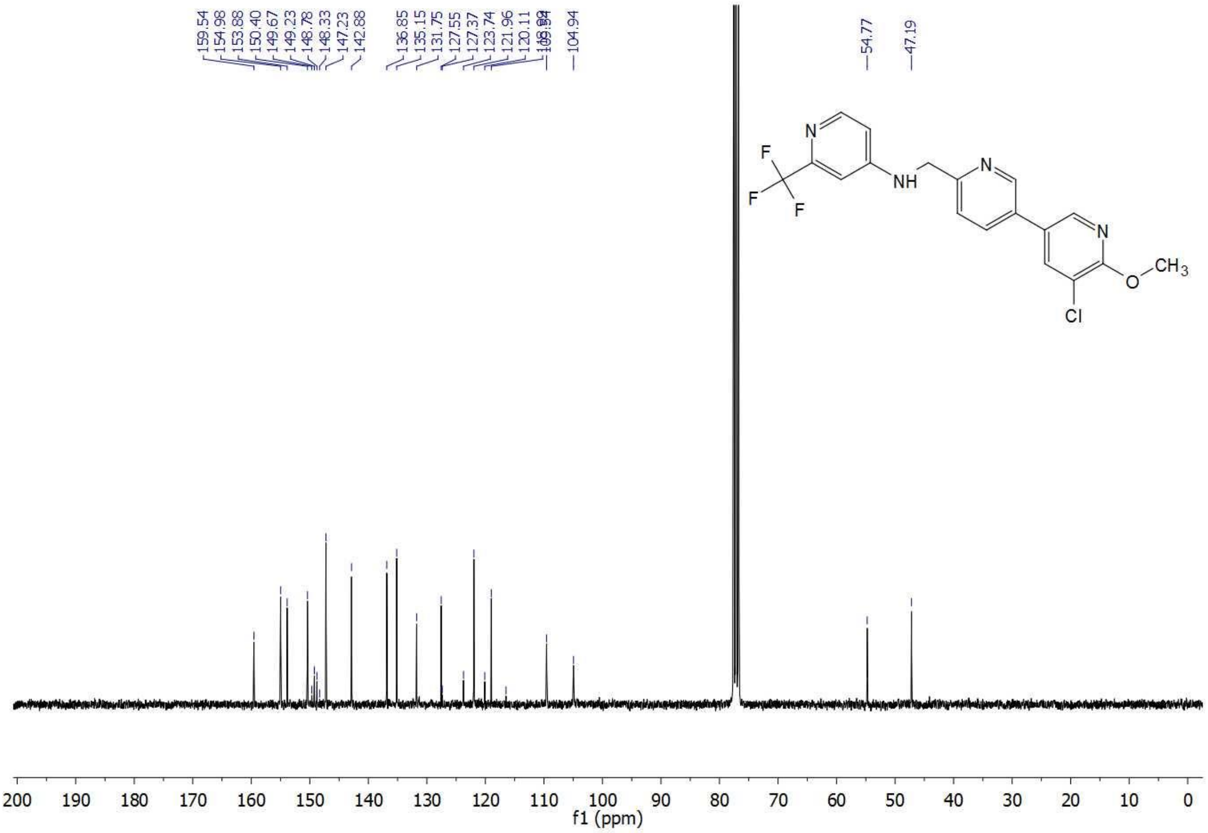
159.54
154.98
153.88
150.40
149.67
149.23
148.78
148.33
147.23
142.88
136.85
135.15
131.75
127.55
127.37
123.74
121.96
120.11
104.94
54.77
47.19
N
F
F
F
NH
N
N
O
CH3
Cl
200 190 180 170 160 150 140 130 120 110 100 90 80 70 60 50 40 30 20 10 0
f1 (ppm)

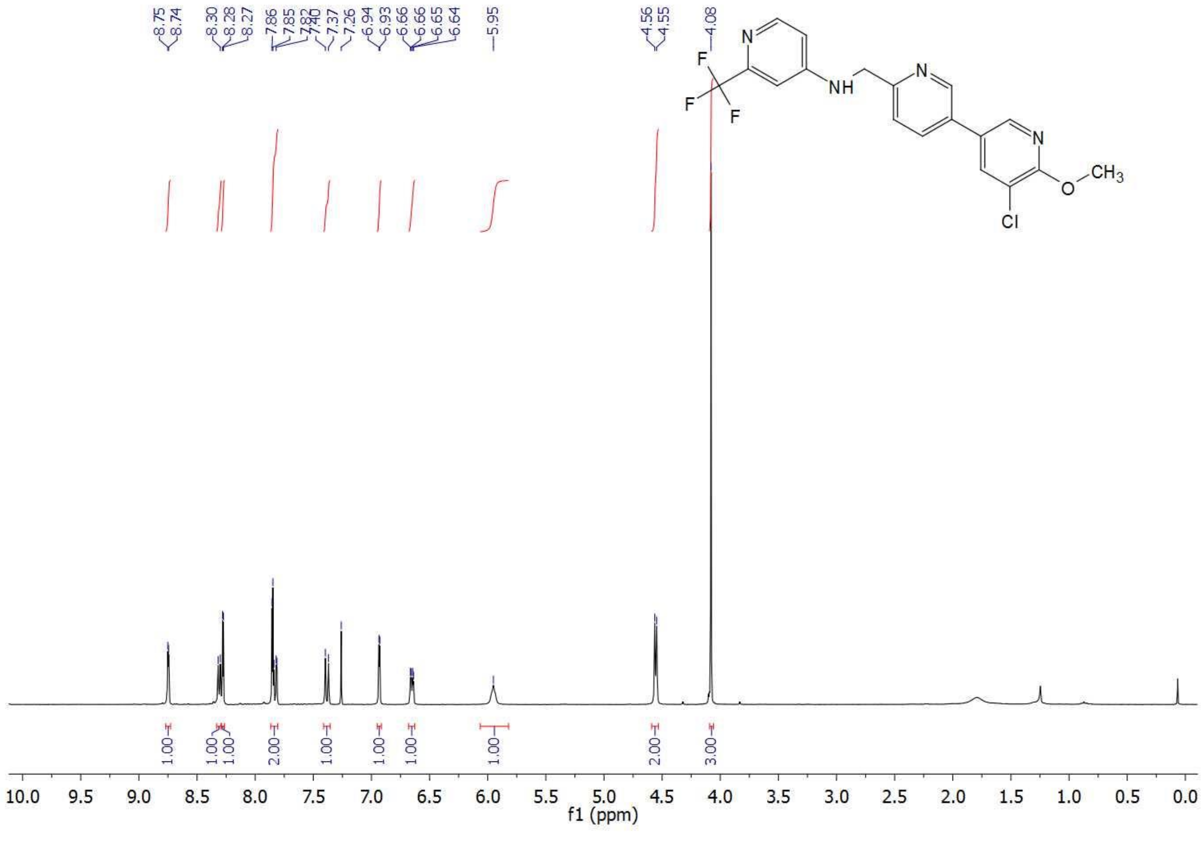
8.75
8.74
8.30
8.28
8.27
7.86
7.85
7.37
7.26
6.94
6.93
6.66
6.66
6.65
6.64
5.95
4.56
4.55
4.08
N
F
F
F
NH
N
N
O
CH3
Cl
1.00
1.00
1.00
2.00
1.00
1.00
1.00
1.00
2.00
3.00
10.0 9.5 9.0 8.5 8.0 7.5 7.0 6.5 6.0 5.5 5.0 4.5 4.0 3.5 3.0 2.5 2.0 1.5 1.0 0.5 0.0
f1 (ppm)

Compound **19**

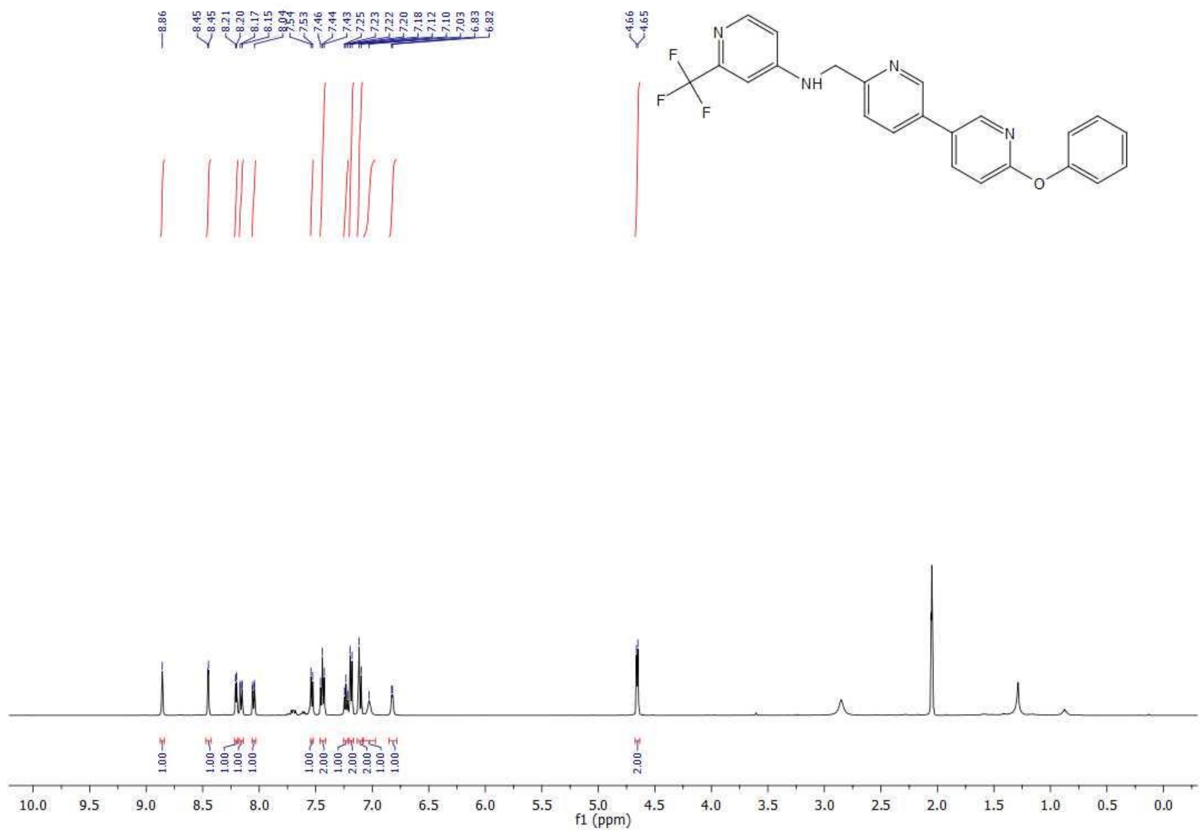

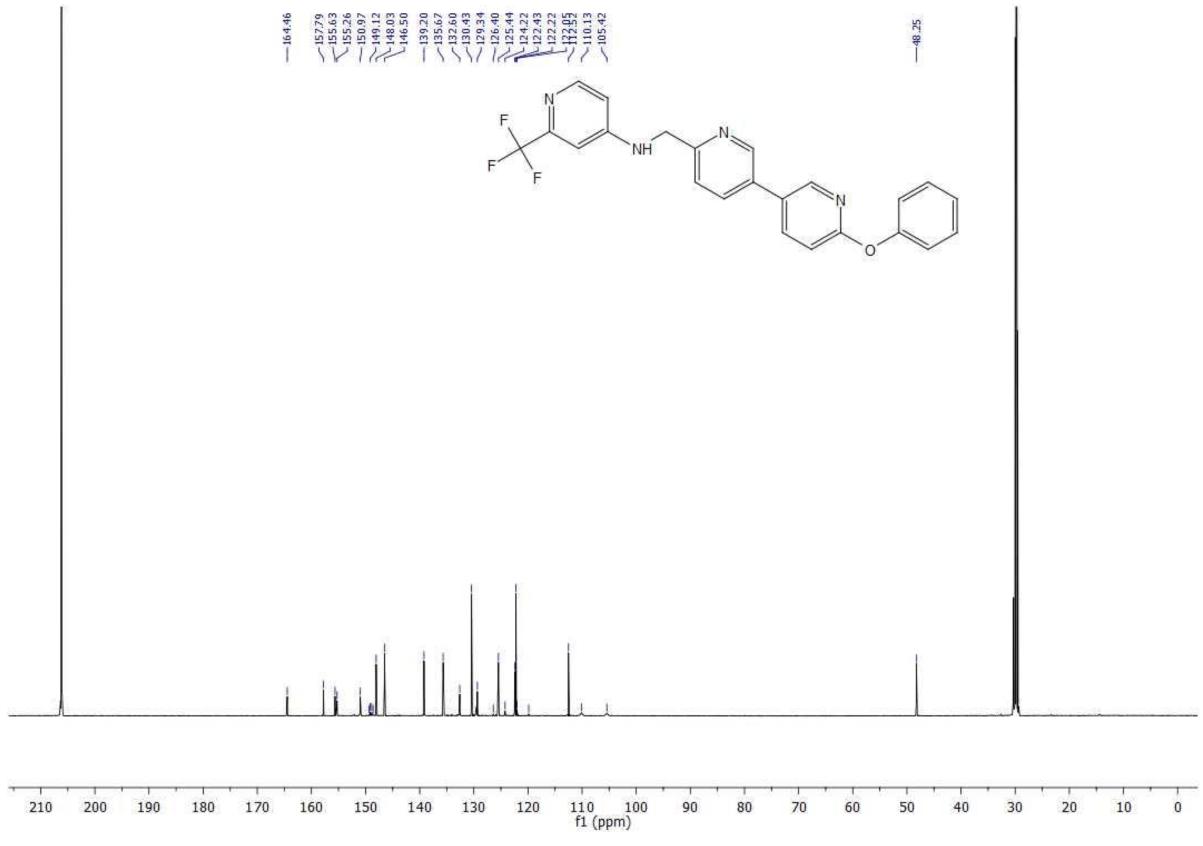

Compound **20**

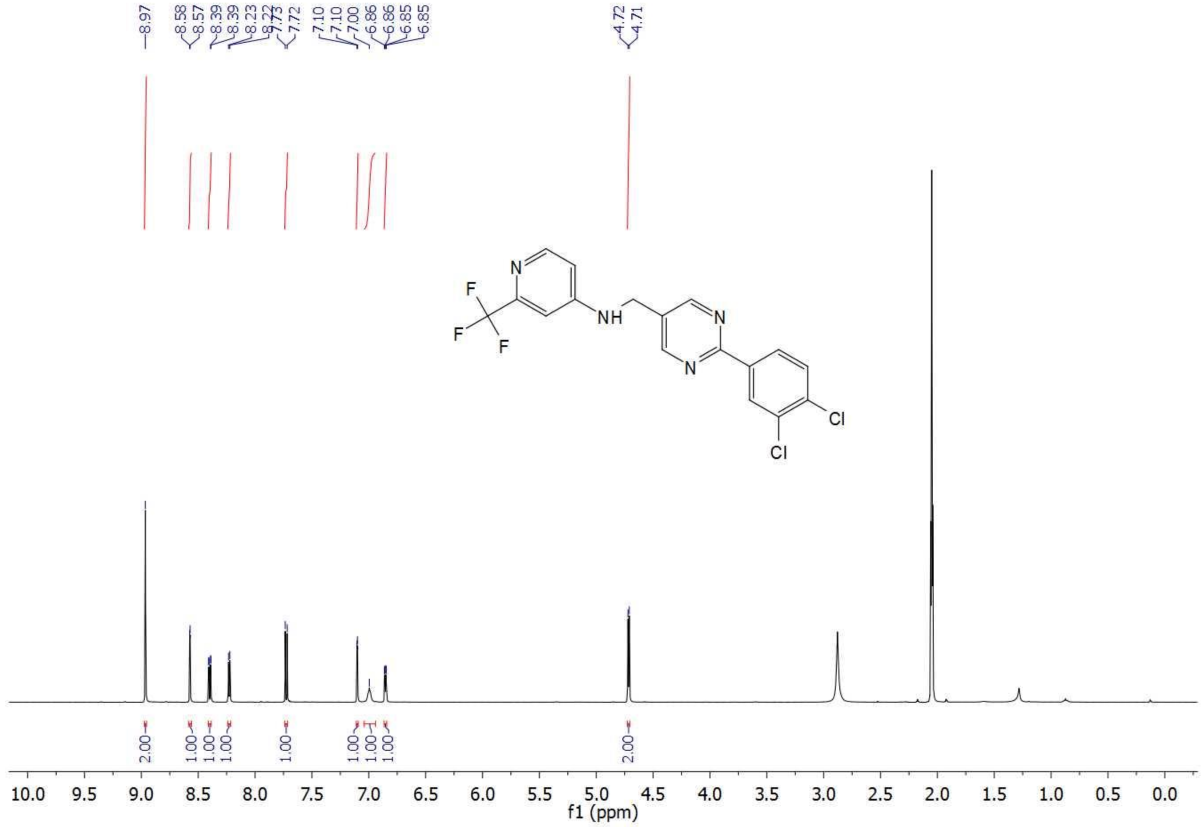
8.97
8.58
8.57
8.39
8.39
8.23
8.22
7.73
7.72
7.10
7.10
7.00
6.86
6.86
6.85
6.85
4.72
4.71
N
F
F
F
NH
N
N
Cl
Cl
2.00
1.00
1.00
1.00
1.00
1.00
1.00
1.00
2.00
10.0 9.5 9.0 8.5 8.0 7.5 7.0 6.5 6.0 5.5 5.0 4.5 4.0 3.5 3.0 2.5 2.0 1.5 1.0 0.5 0.0
f1 (ppm)

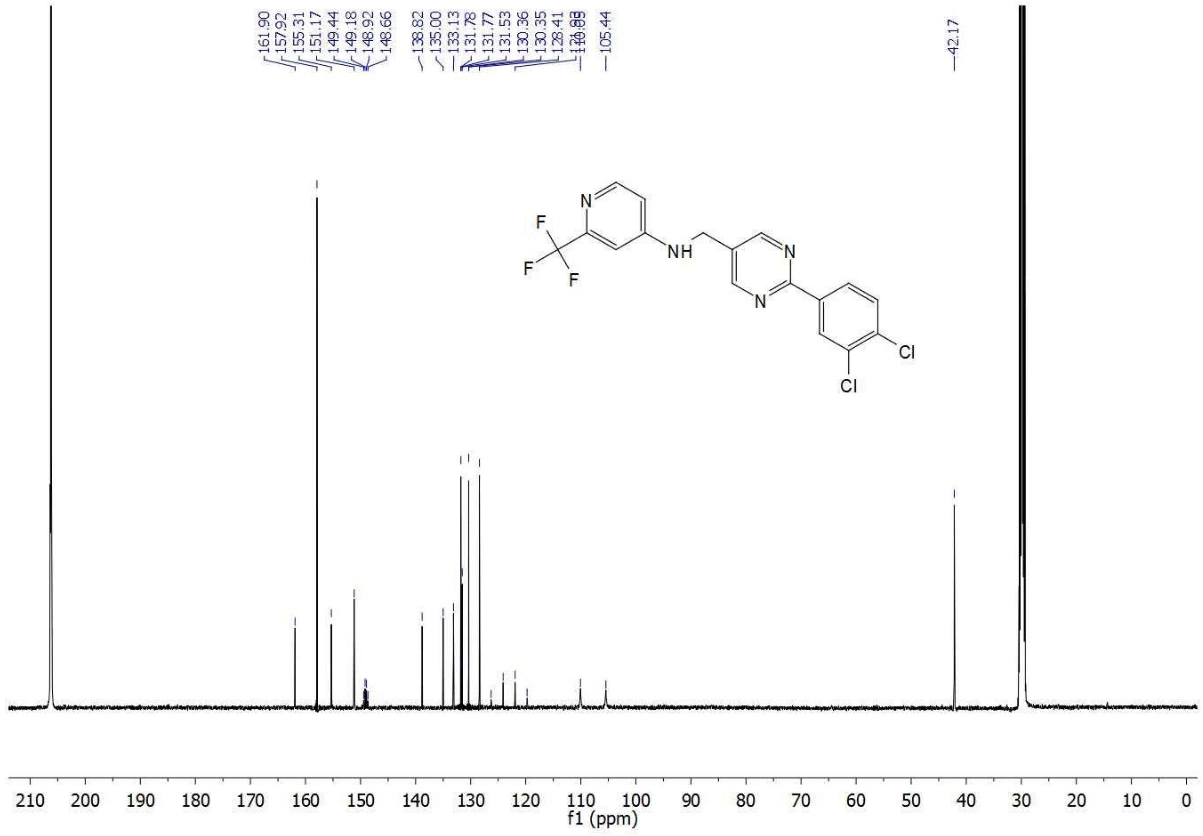
161.90
157.92
155.31
151.17
149.44
149.18
148.92
148.66
138.82
135.00
133.13
131.78
131.77
131.53
130.36
130.35
128.41
105.44
42.17
N
F
F
F
NH
N
N
Cl
Cl
210 200 190 180 170 160 150 140 130 120 110 100 90 80 70 60 50 40 30 20 10 0
f1 (ppm)

Compound **21**

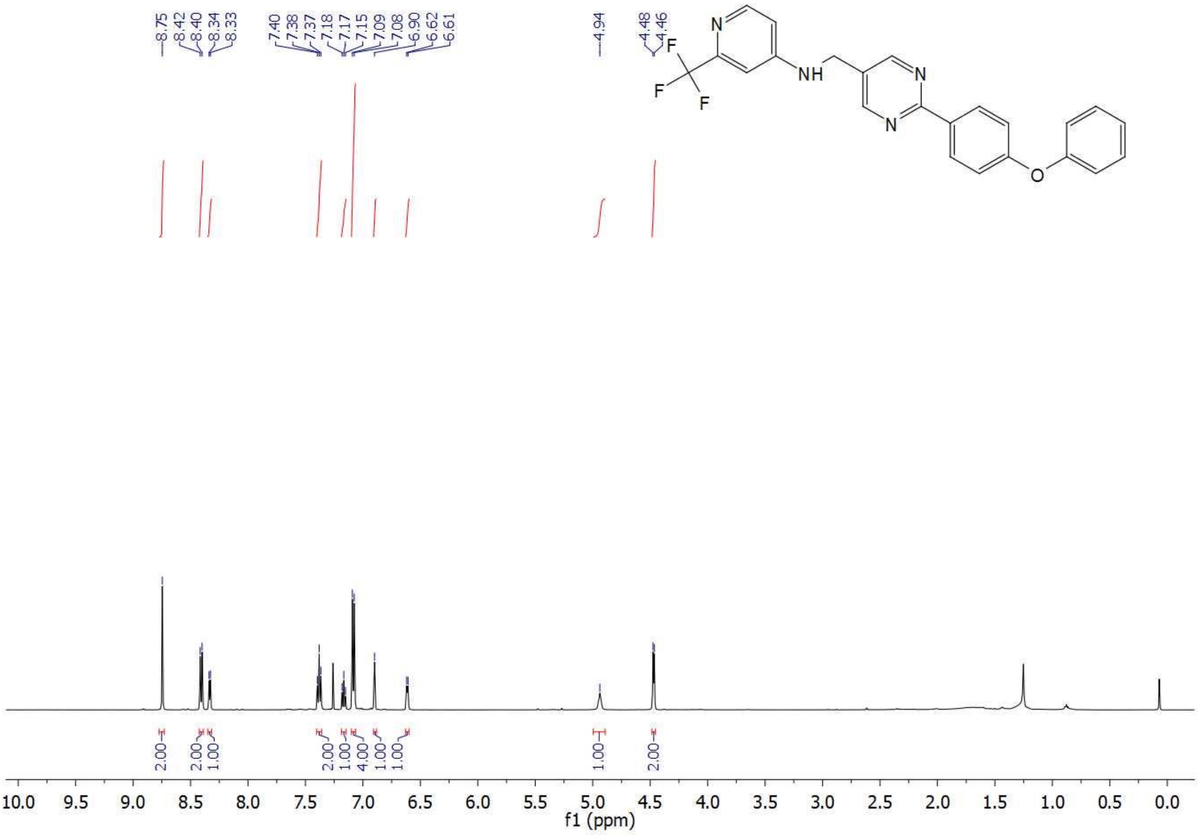

8.75
8.42
8.40
8.34
8.33
7.40
7.38
7.37
7.18
7.17
7.15
7.09
7.08
6.90
6.62
6.61
4.94
4.48
4.46
N
F
F
F
NH
N
N
O
2.00
2.00
1.00
2.00
1.00
4.00
1.00
1.00
1.00
2.00
10.0 9.5 9.0 8.5 8.0 7.5 7.0 6.5 6.0 5.5 5.0 4.5 4.0 3.5 3.0 2.5 2.0 1.5 1.0 0.5 0.0
f1 (ppm)

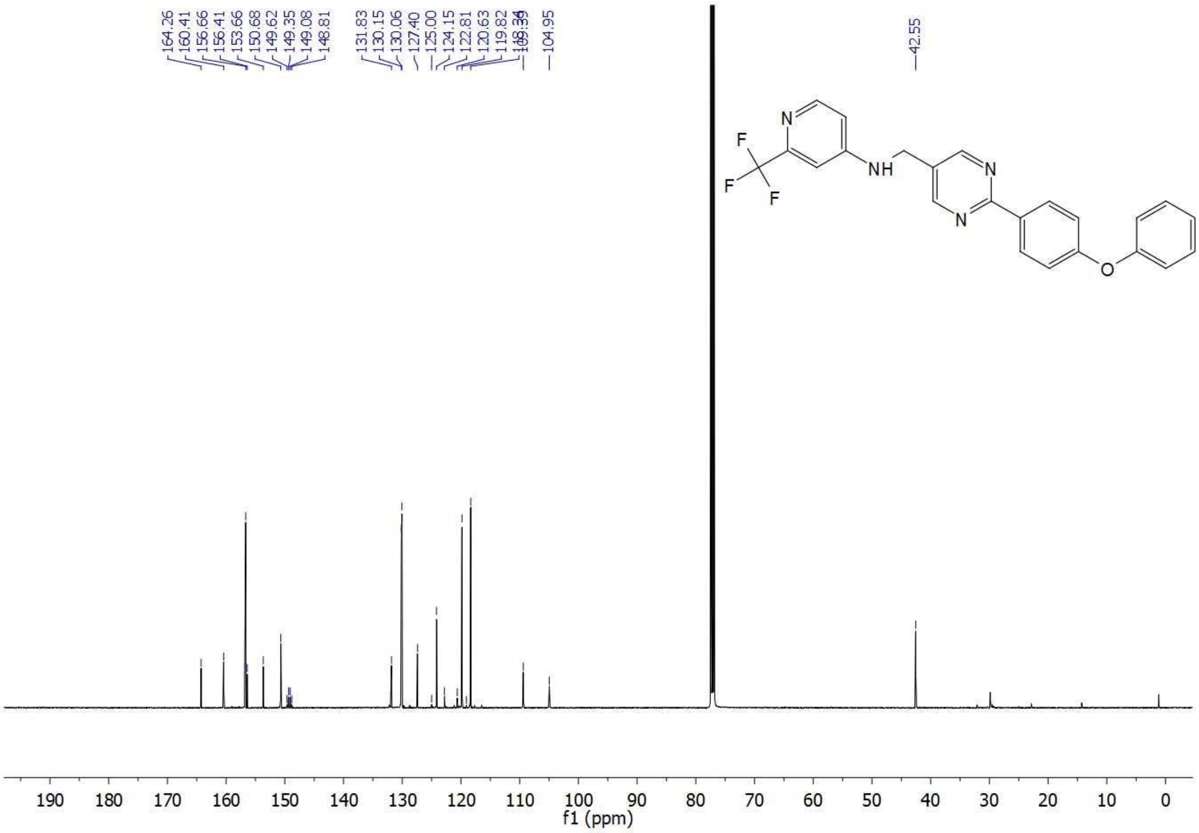

164.26
160.41
156.66
156.41
153.66
150.68
149.62
149.35
149.08
148.81
131.83
130.15
130.06
127.40
125.00
124.15
122.81
120.63
119.82
104.95
42.55
N
F
F
F
NH
N
N
O
190 180 170 160 150 140 130 120 110 100 90 80 70 60 50 40 30 20 10 0
f1 (ppm)

Compound **22**

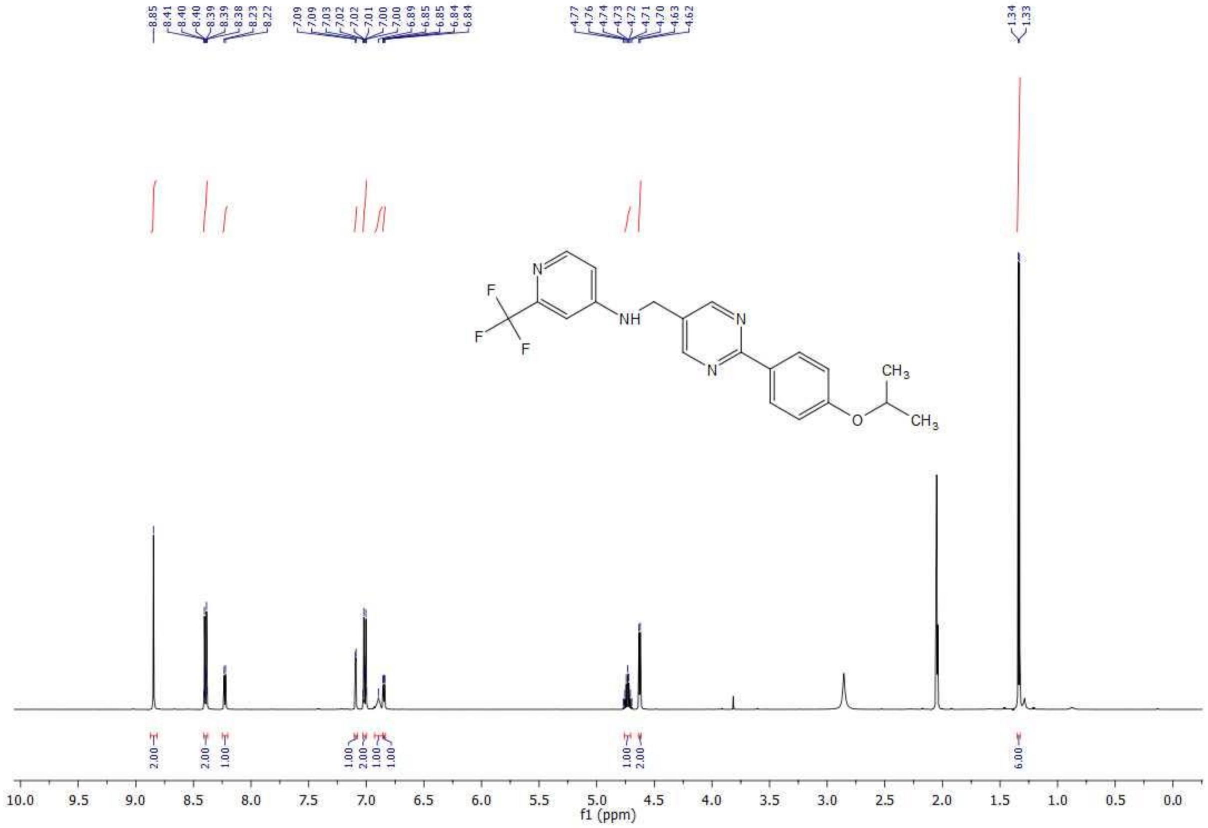

8.85
8.41
8.40
8.39
8.39
8.38
8.23
8.22
7.09
7.09
7.03
7.02
7.02
7.01
7.00
7.00
6.89
6.85
6.85
6.84
6.84
4.77
4.76
4.74
4.73
4.72
4.71
4.70
4.63
4.62
1.34
1.33
N
F
F
F
NH
N
N
CH3
O
CH3
2.00
2.00
1.00
1.00
2.00
1.00
1.00
1.00
2.00
6.00
10.0 9.5 9.0 8.5 8.0 7.5 7.0 6.5 6.0 5.5 5.0 4.5 4.0 3.5 3.0 2.5 2.0 1.5 1.0 0.5 0.0
f1 (ppm)

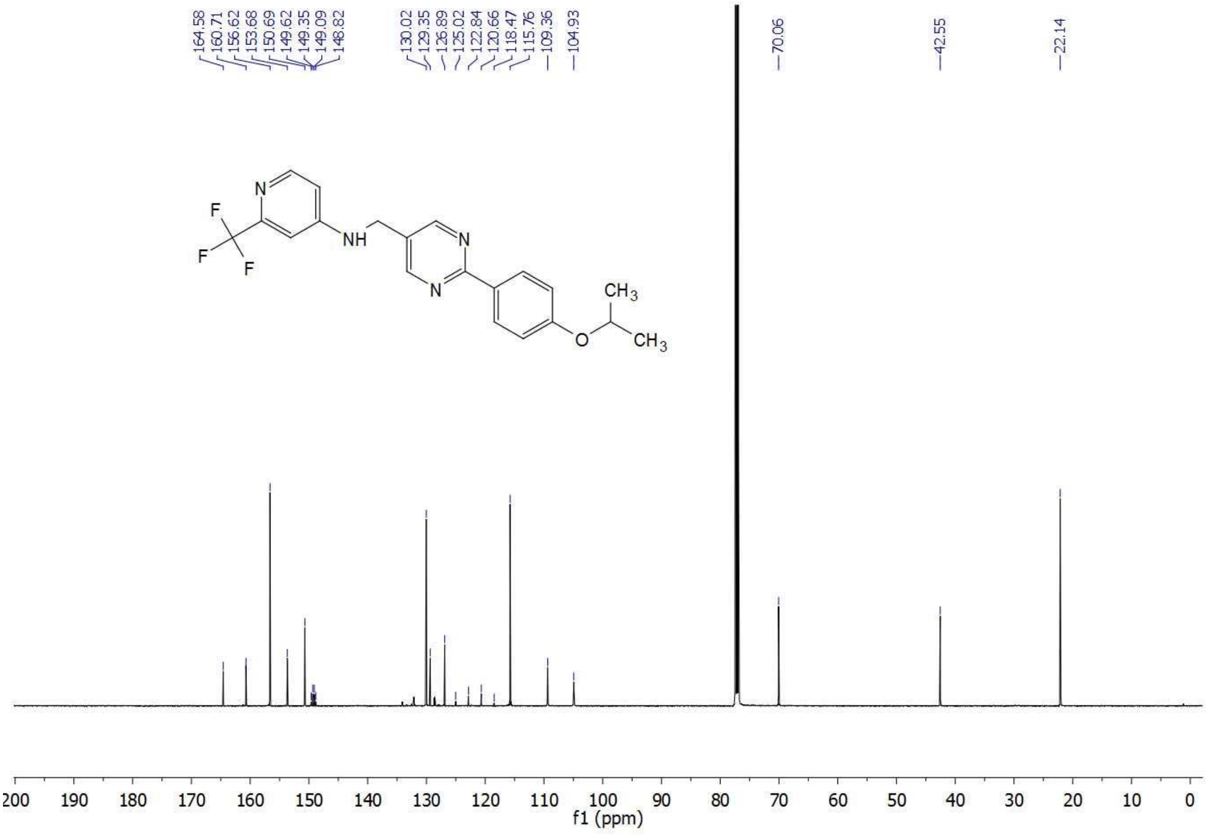

164.58
160.71
156.62
153.68
150.69
149.62
149.35
149.09
148.82
130.02
129.35
126.89
125.02
122.84
120.66
118.47
115.76
109.36
104.93
70.06
42.55
22.14
N
F
F
F
NH
N
N
CH3
O
CH3
200 190 180 170 160 150 140 130 120 110 100 90 80 70 60 50 40 30 20 10 0
f1 (ppm)

Compound **23**

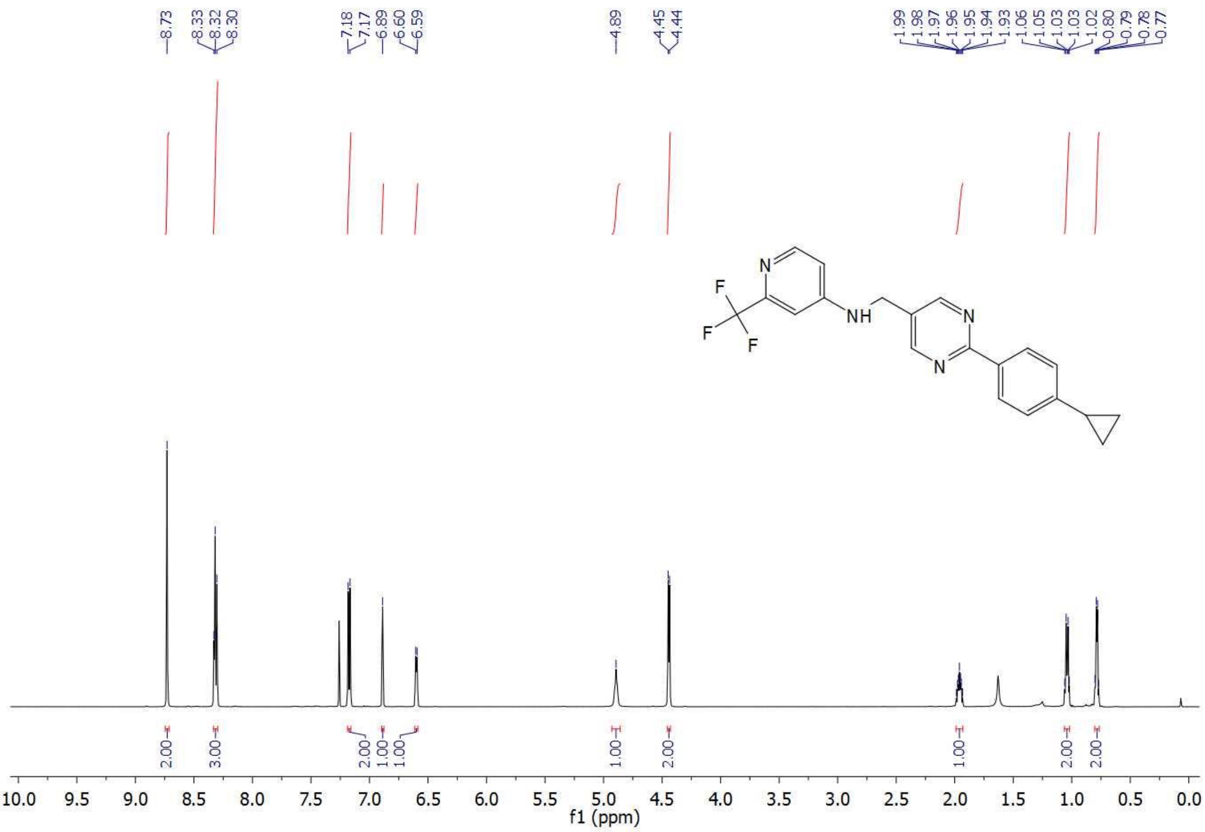
8.73
8.33
8.32
8.30
7.18
7.17
6.89
6.60
6.59
4.89
4.45
4.44
1.99
1.98
1.97
1.96
1.95
1.94
1.93
1.06
1.05
1.03
1.03
1.02
0.80
0.79
0.78
0.77
2.00
3.00
2.00
1.00
1.00
1.00
2.00
1.00
2.00
2.00
N
F
F
F
NH
N
N
f1 (ppm)

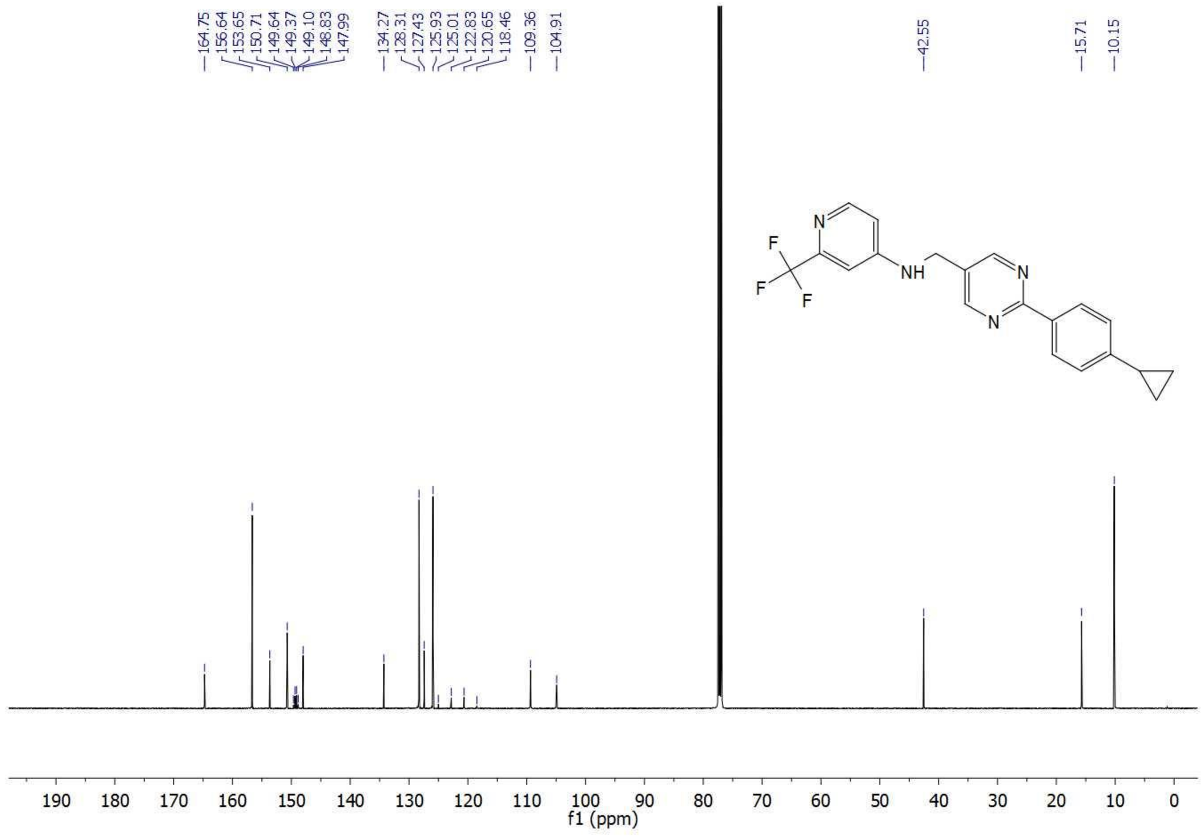
164.75
156.64
153.65
150.71
149.64
149.37
149.10
148.83
147.99
134.27
128.31
127.43
125.93
125.01
122.83
120.65
118.46
109.36
104.91
42.55
15.71
10.15
N
F
F
F
NH
N
N
f1 (ppm)

Compound **24**

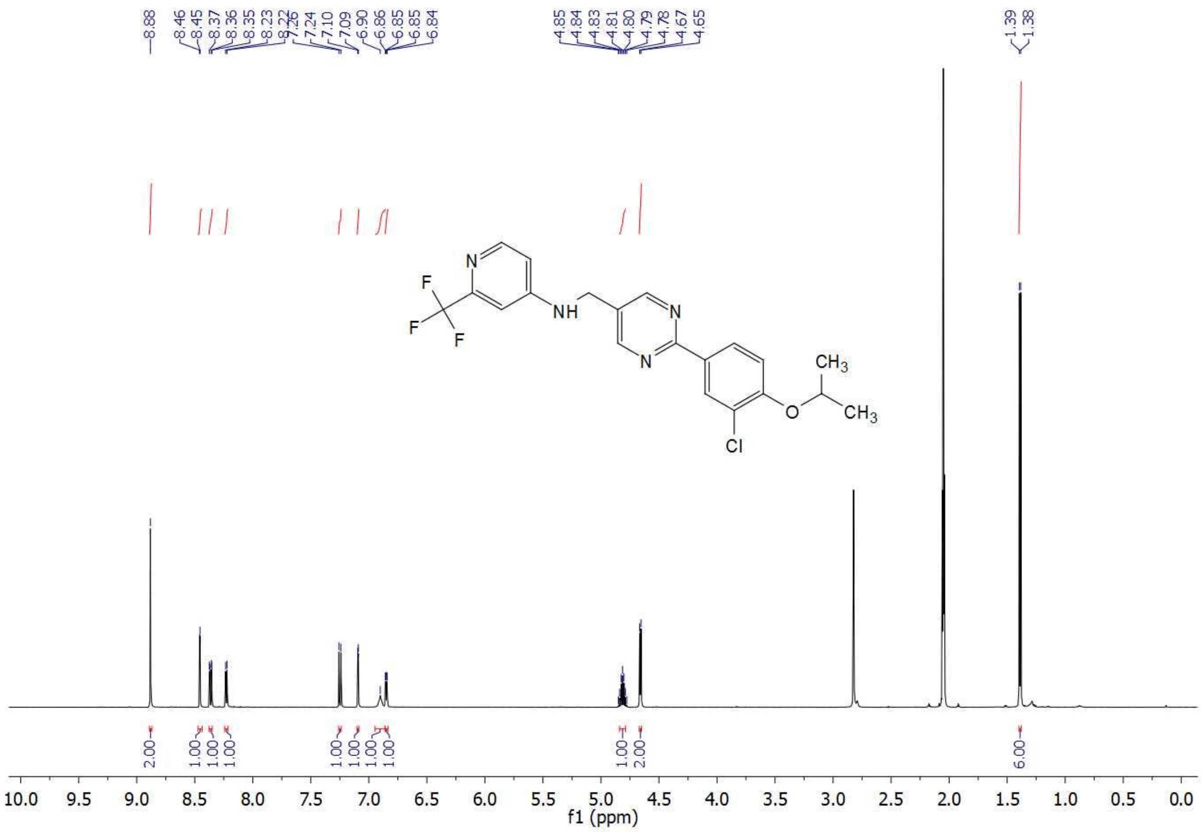
8.88
8.46 8.45 8.37 8.36 8.35 8.23 8.22 7.26 7.24 7.10 7.09 6.90 6.86 6.85 6.85 6.84
4.85 4.84 4.83 4.81 4.80 4.79 4.78 4.67 4.65
1.39 1.38
N
F
F
F
NH
N
N
CH3
O
CH3
Cl
2.00
1.00 1.00 1.00
1.00 1.00 1.00 1.00
1.00 2.00
6.00
10.0 9.5 9.0 8.5 8.0 7.5 7.0 6.5 6.0 5.5 5.0 4.5 4.0 3.5 3.0 2.5 2.0 1.5 1.0 0.5 0.0
f1 (ppm)

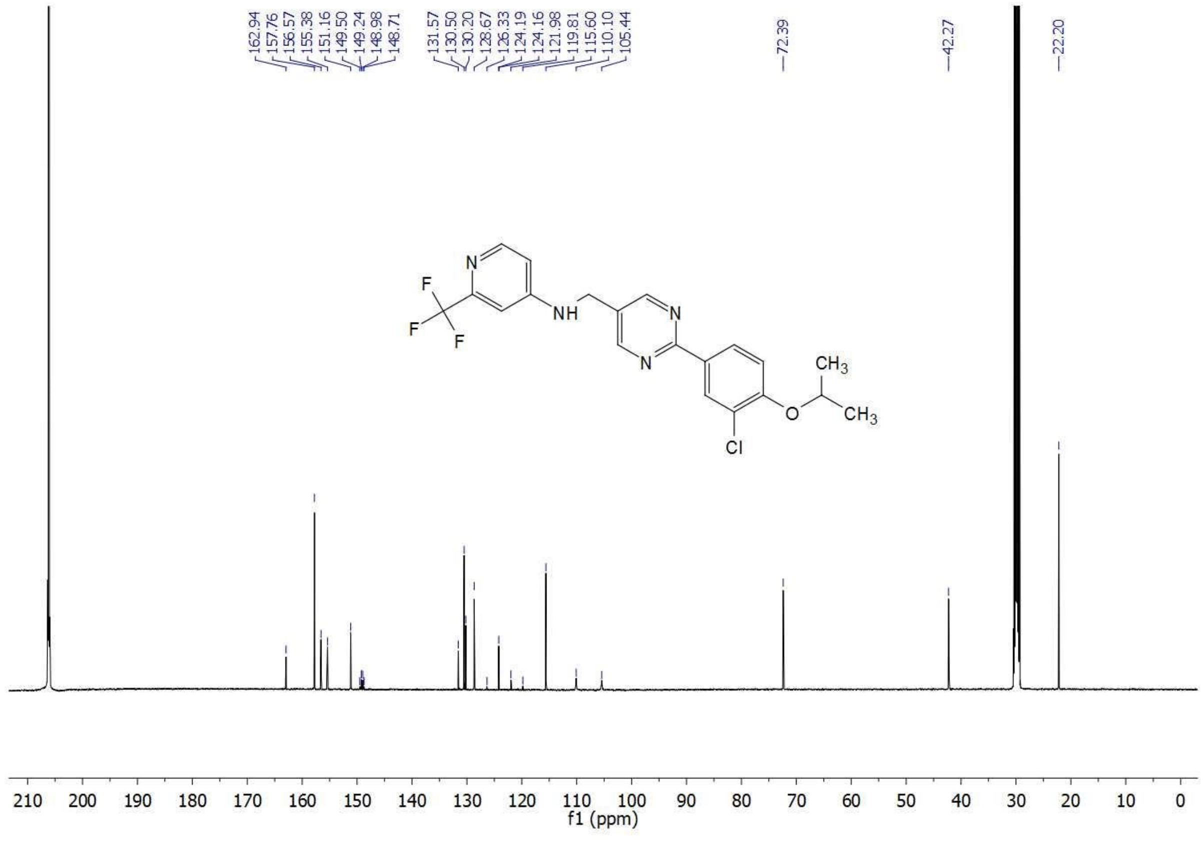
162.94 157.76 156.57 155.38 151.16 149.50 149.24 148.98 148.71
131.57 130.50 130.20 128.67 126.33 124.19 124.16 121.98 119.81 115.60 110.10 105.44
72.39
42.27
22.20
N
F
F
F
NH
N
N
CH3
O
CH3
Cl
210 200 190 180 170 160 150 140 130 120 110 100 90 80 70 60 50 40 30 20 10 0
f1 (ppm)

Compound **25**

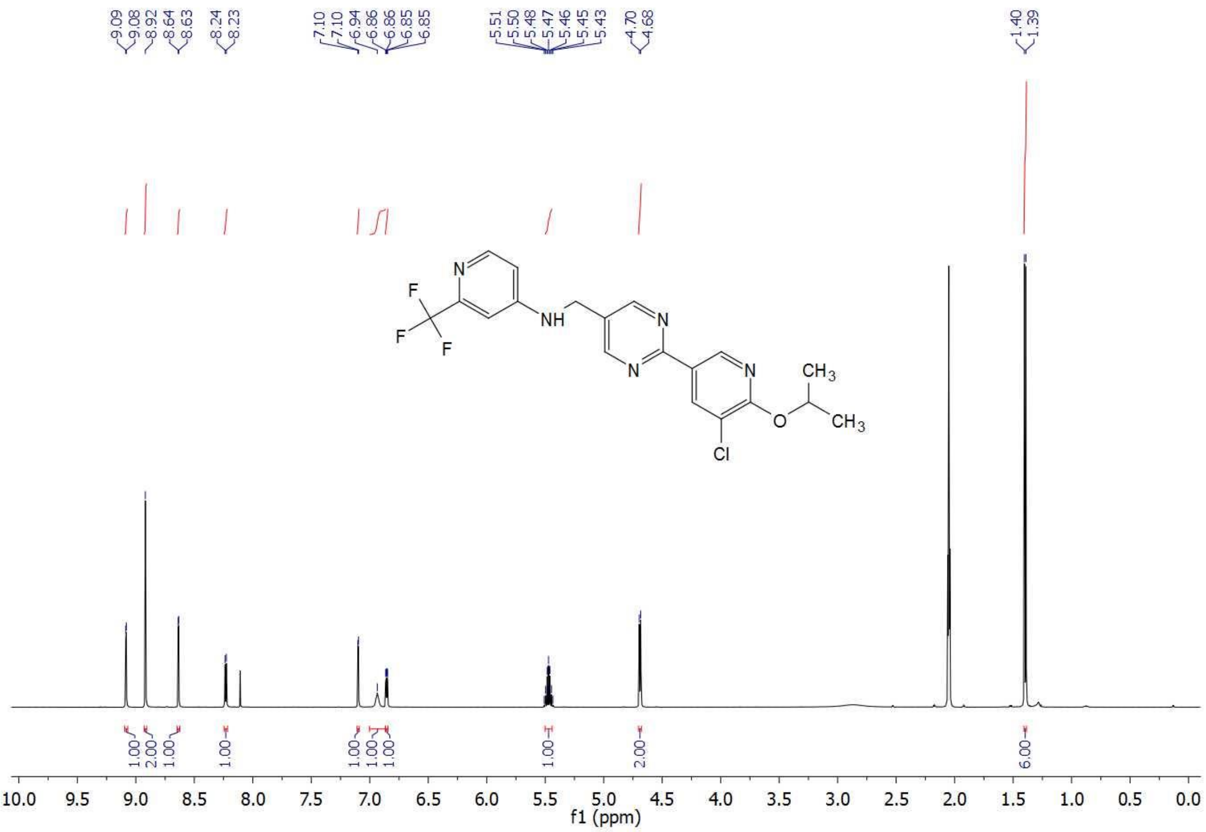
9.09
9.08
8.92
8.64
8.63
8.24
8.23
7.10
7.10
6.94
6.86
6.86
6.85
6.85
5.51
5.50
5.48
5.47
5.46
5.45
5.43
4.70
4.68
1.40
1.39
1.00
2.00
1.00
1.00
1.00
1.00
1.00
1.00
2.00
6.00
10.0 9.5 9.0 8.5 8.0 7.5 7.0 6.5 6.0 5.5 5.0 4.5 4.0 3.5 3.0 2.5 2.0 1.5 1.0 0.5 0.0
f1 (ppm)

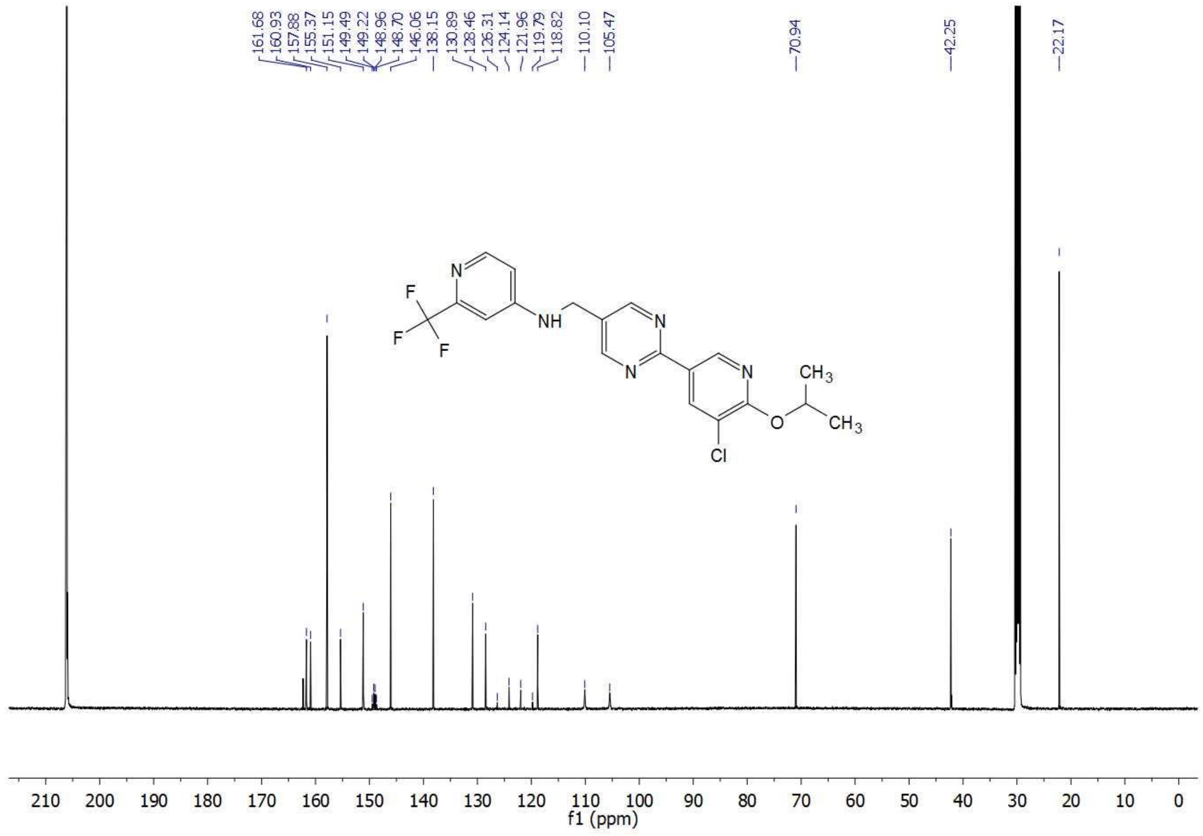
161.68
160.93
157.88
155.37
151.15
149.49
149.22
148.96
148.70
146.06
138.15
130.89
128.46
126.31
124.14
121.96
119.79
118.82
110.10
105.47
70.94
42.25
22.17
210 200 190 180 170 160 150 140 130 120 110 100 90 80 70 60 50 40 30 20 10 0
f1 (ppm)

3 HRMS SPECTRA OF THE DESCRIBED COMPOUNDS

Compound **2**

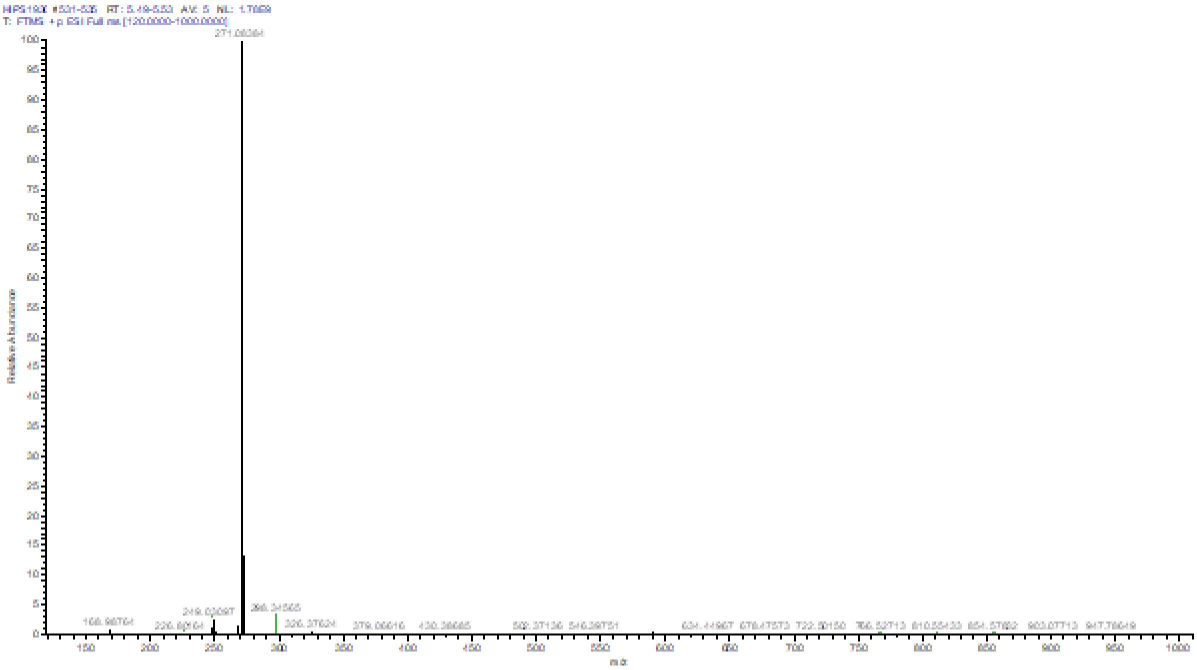

Compound **3**

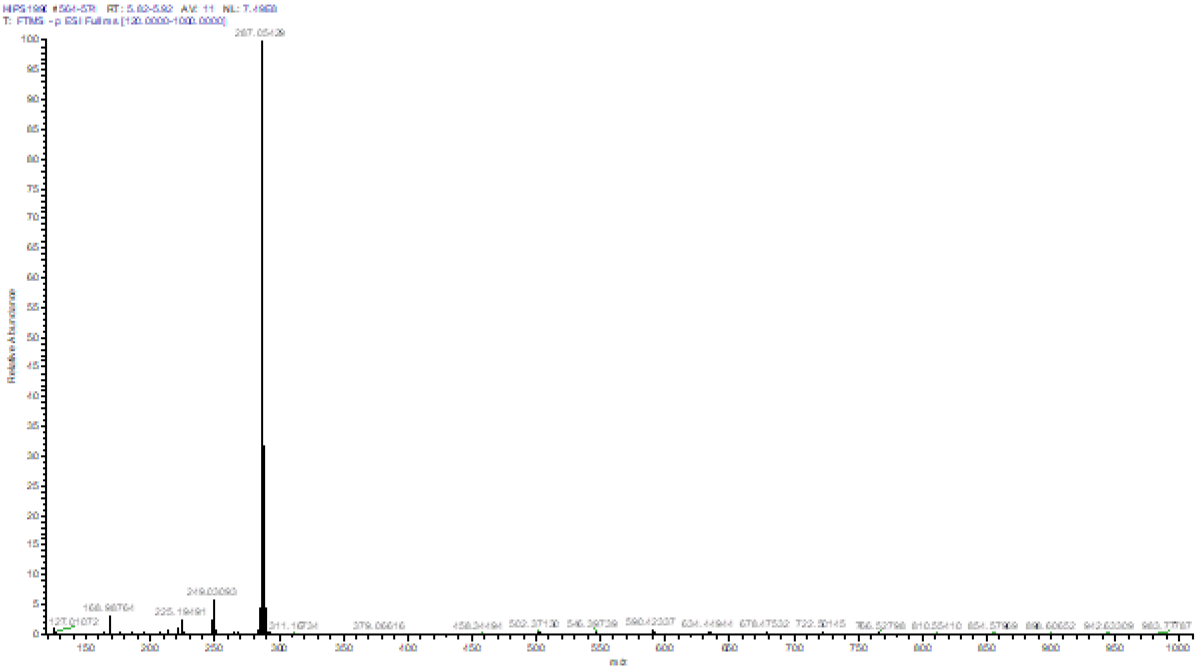

Compound **4**

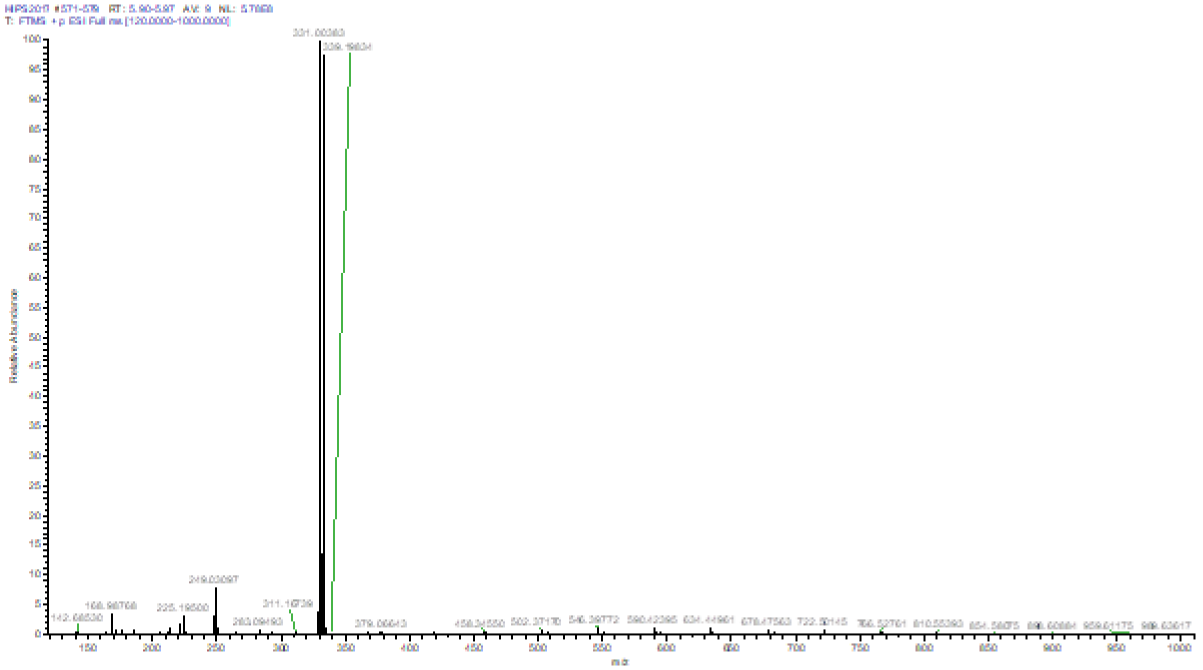

Compound **5**

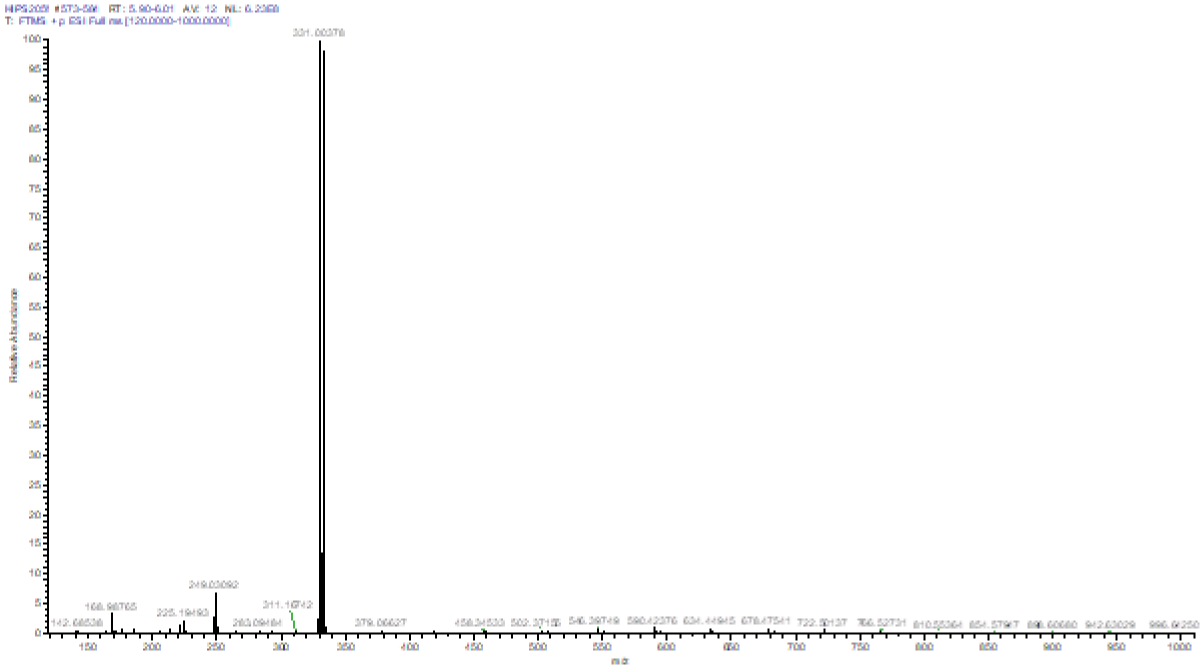

Compound **6**

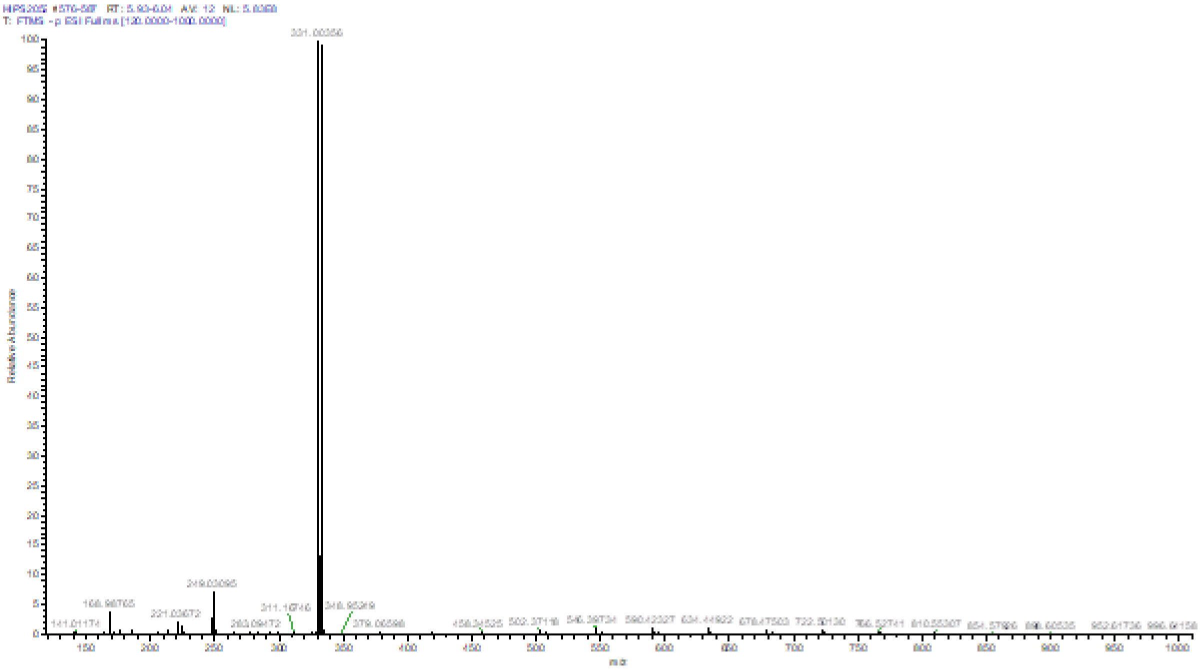

Compound **7**

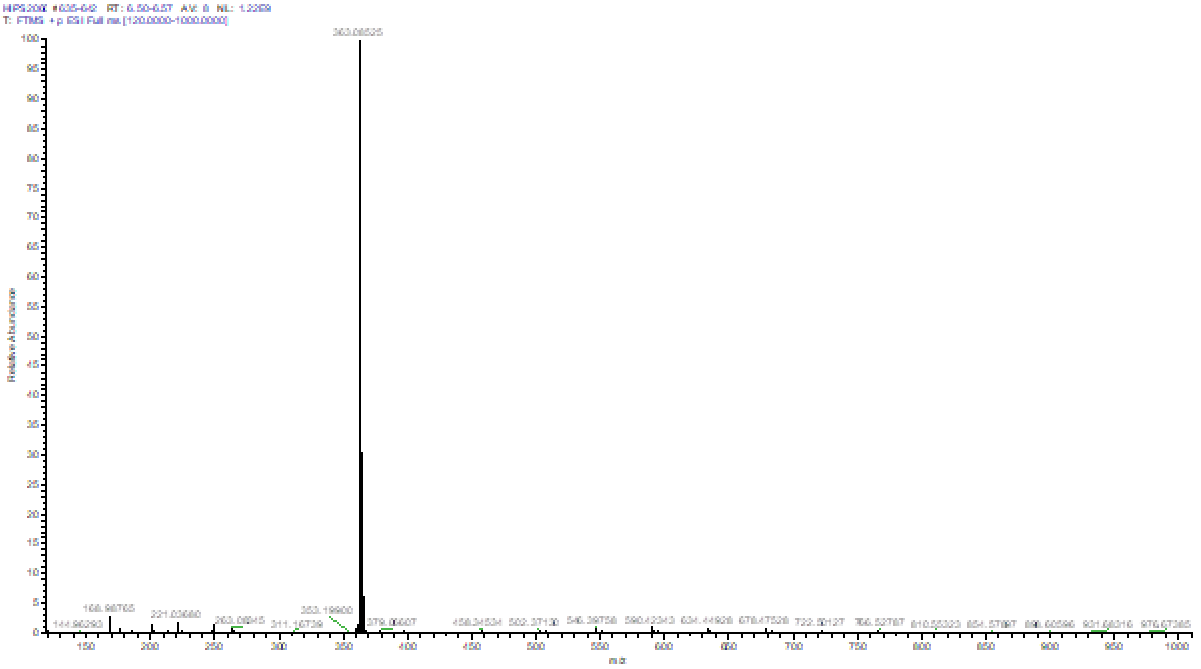

Compound **8**

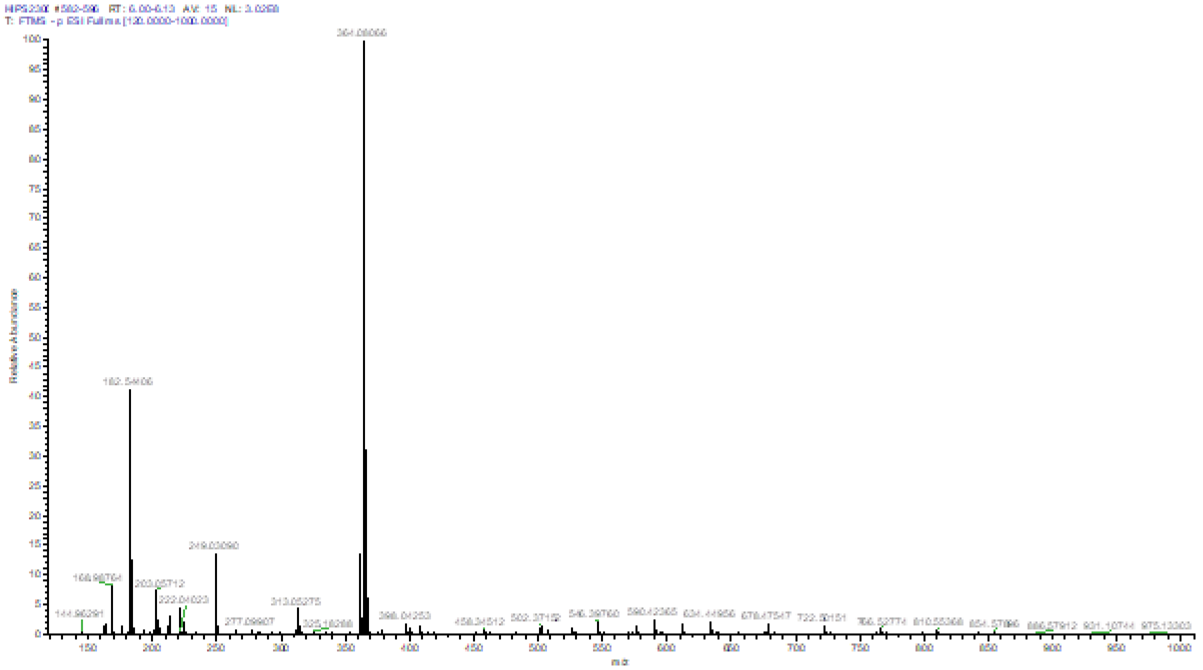

Compound **9**

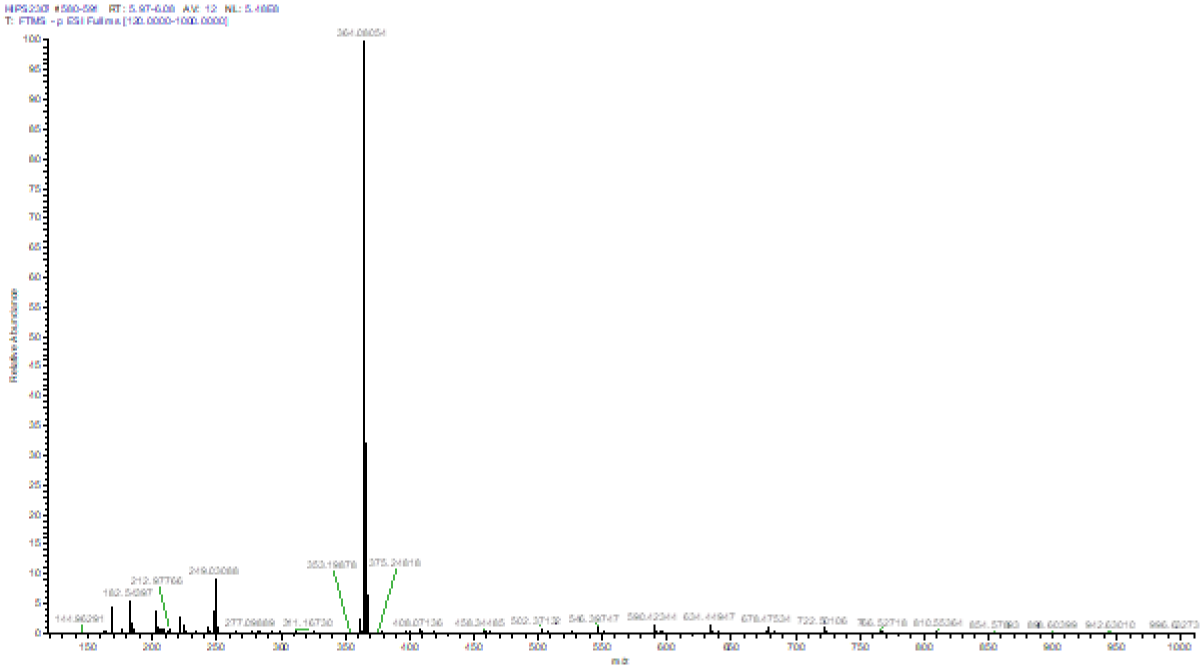

Compound **10**

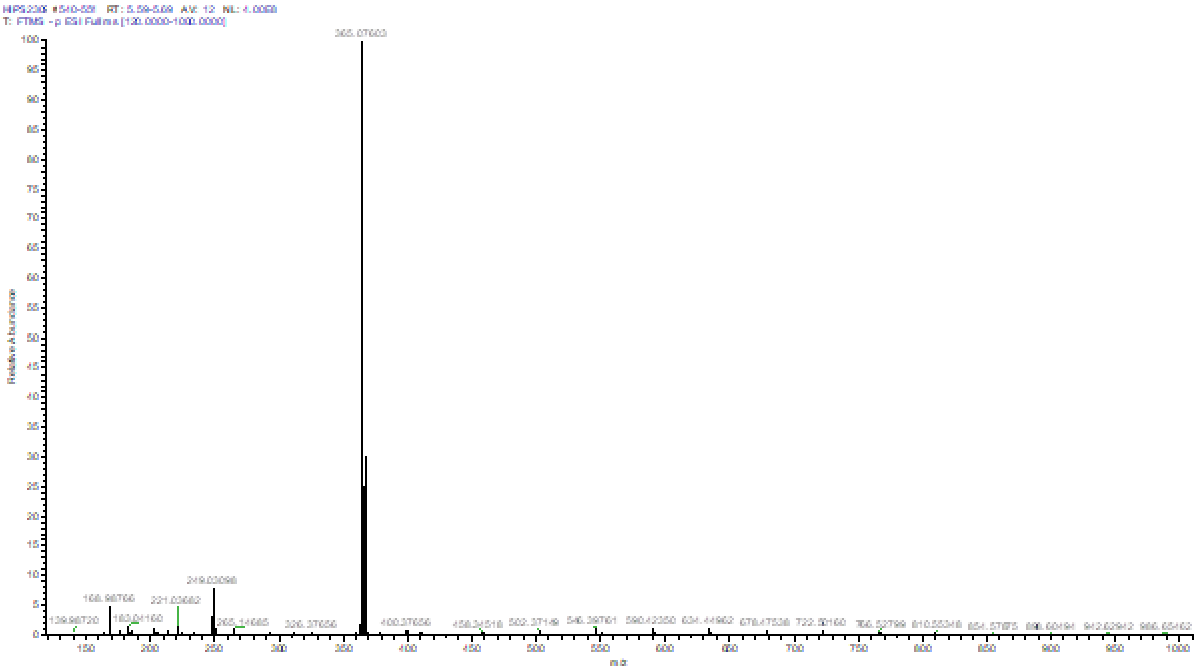

Compound **11**

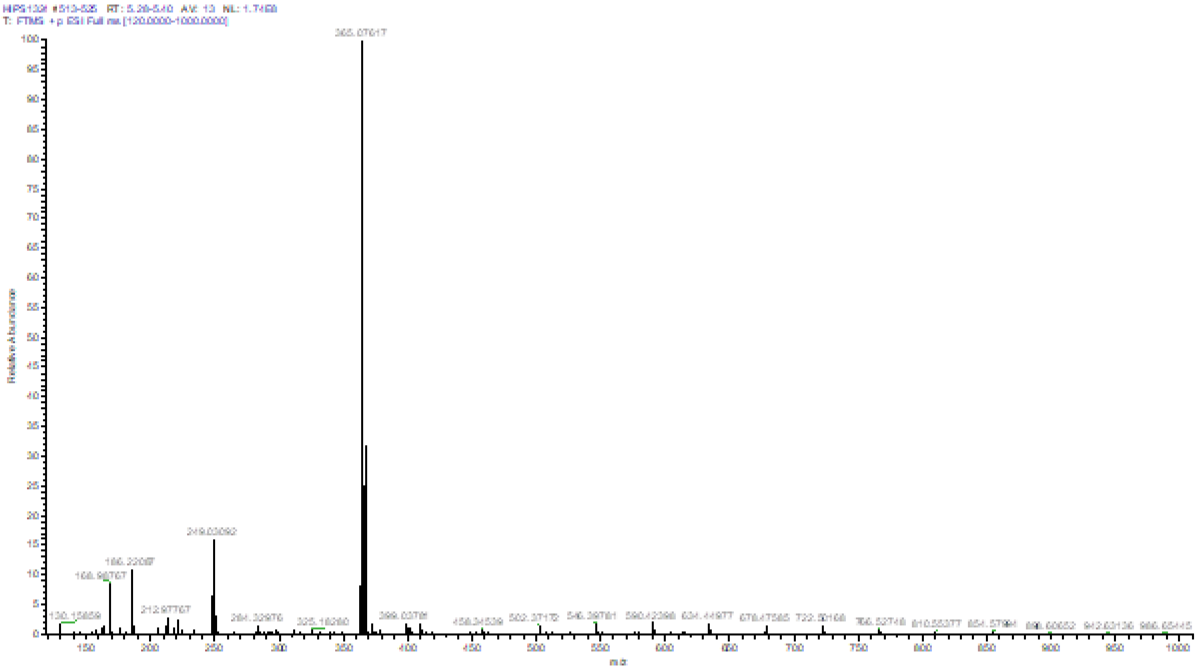

Compound **12**

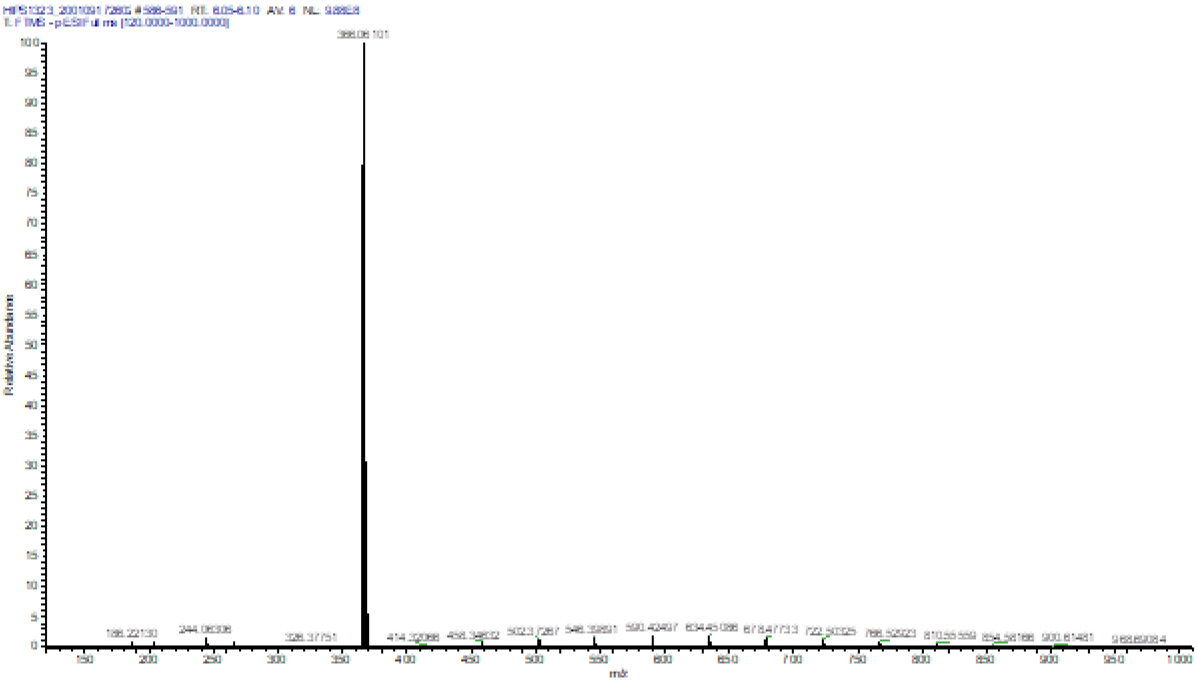

Compound **13**

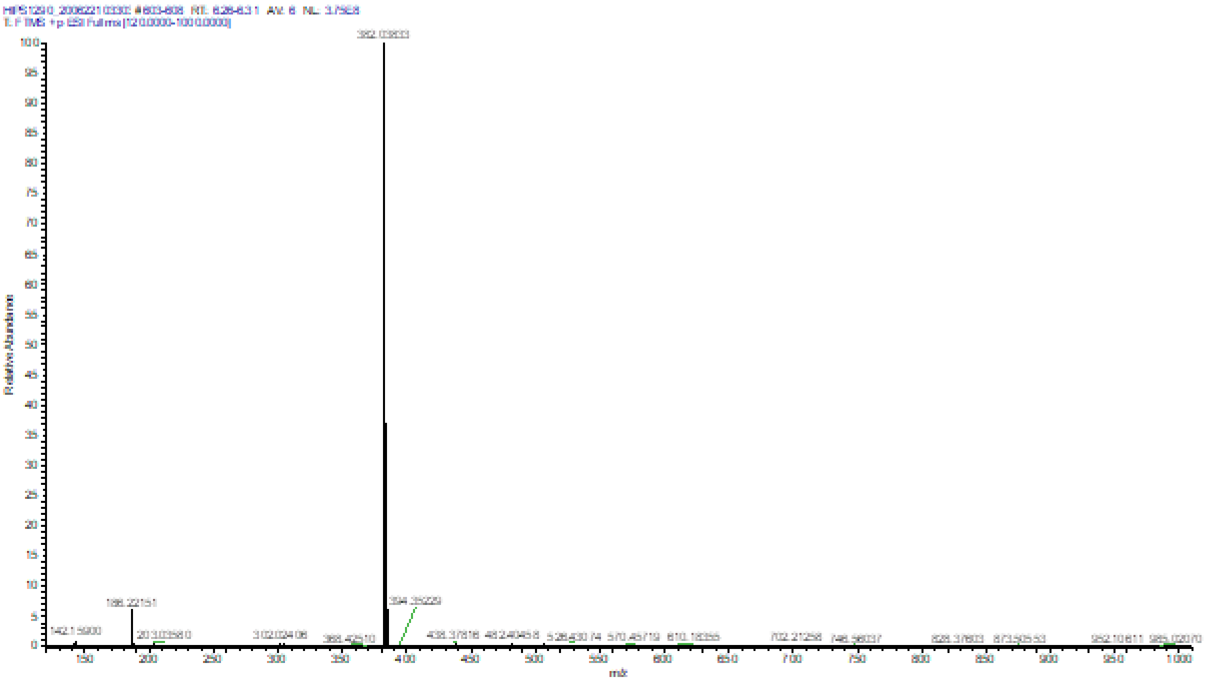

Compound **14**

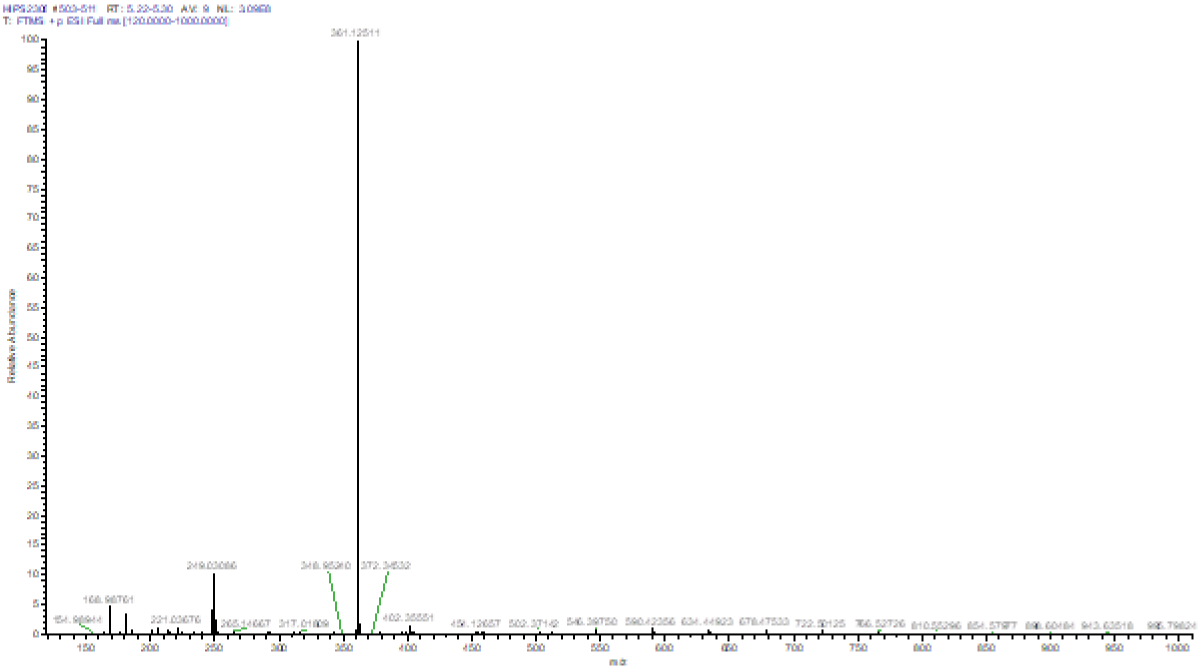

Compound **15**

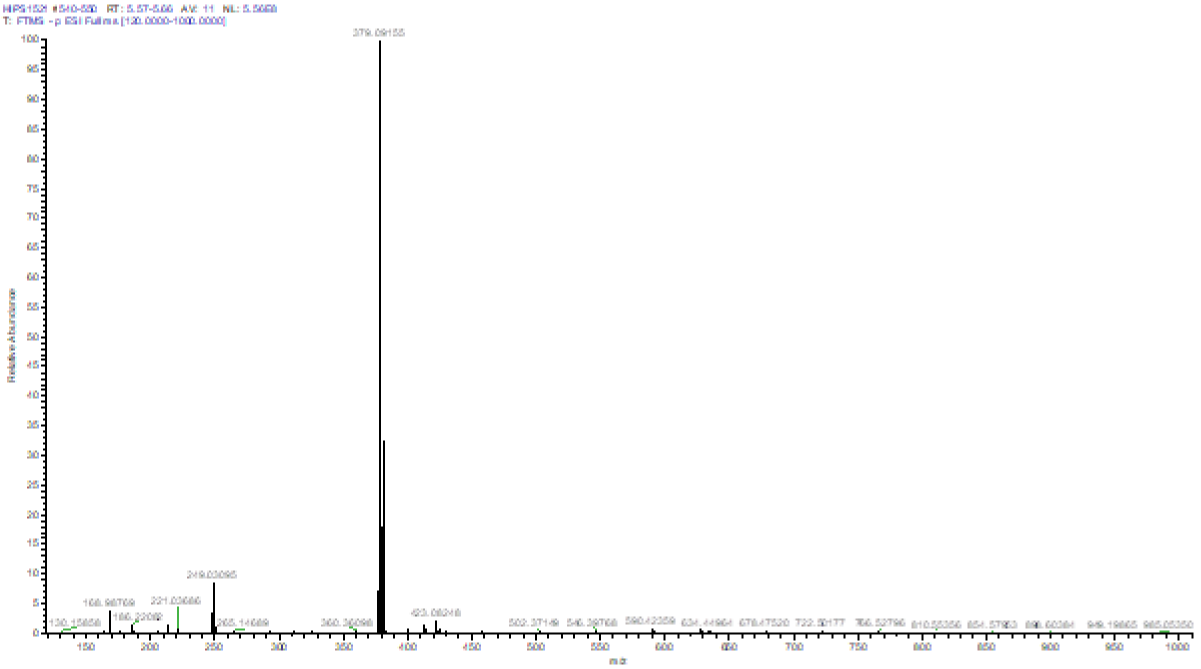

Compound **16**

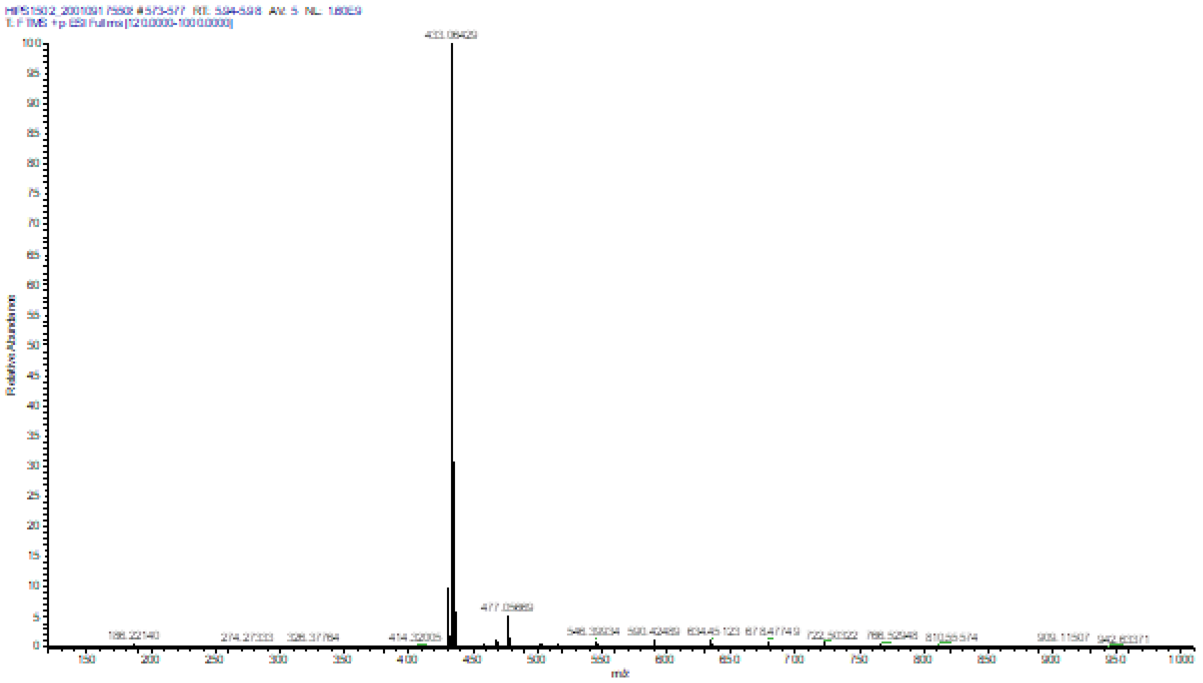

Compound **17**

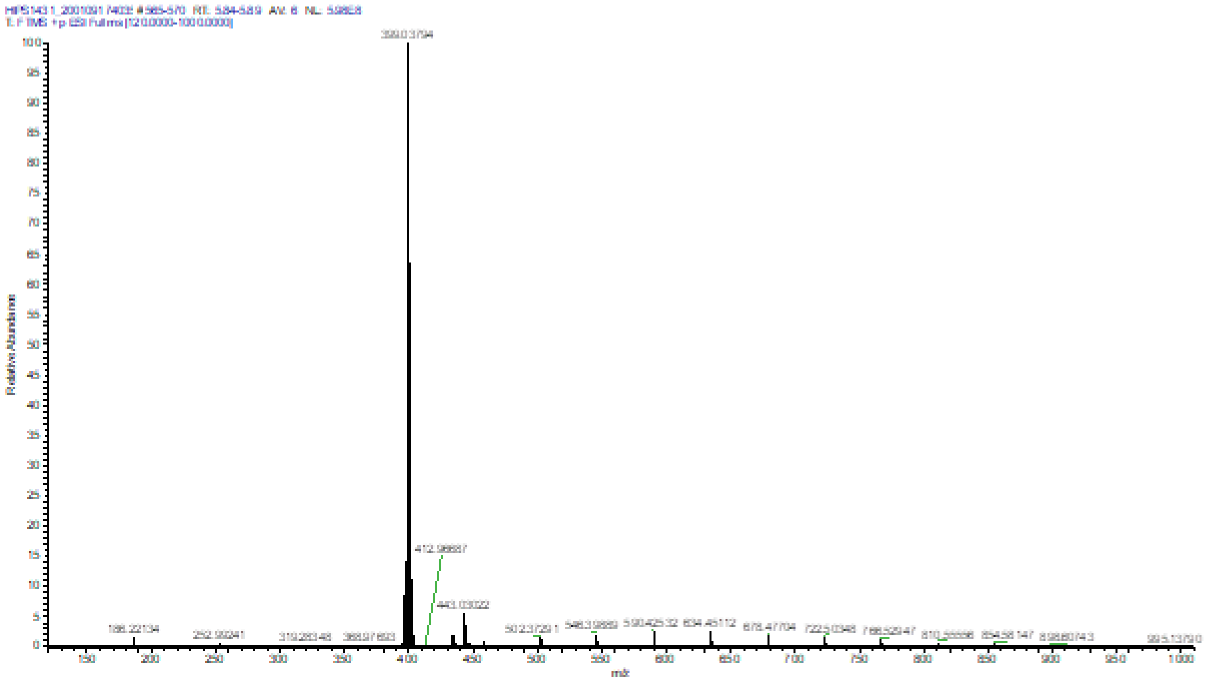

Compound **18**

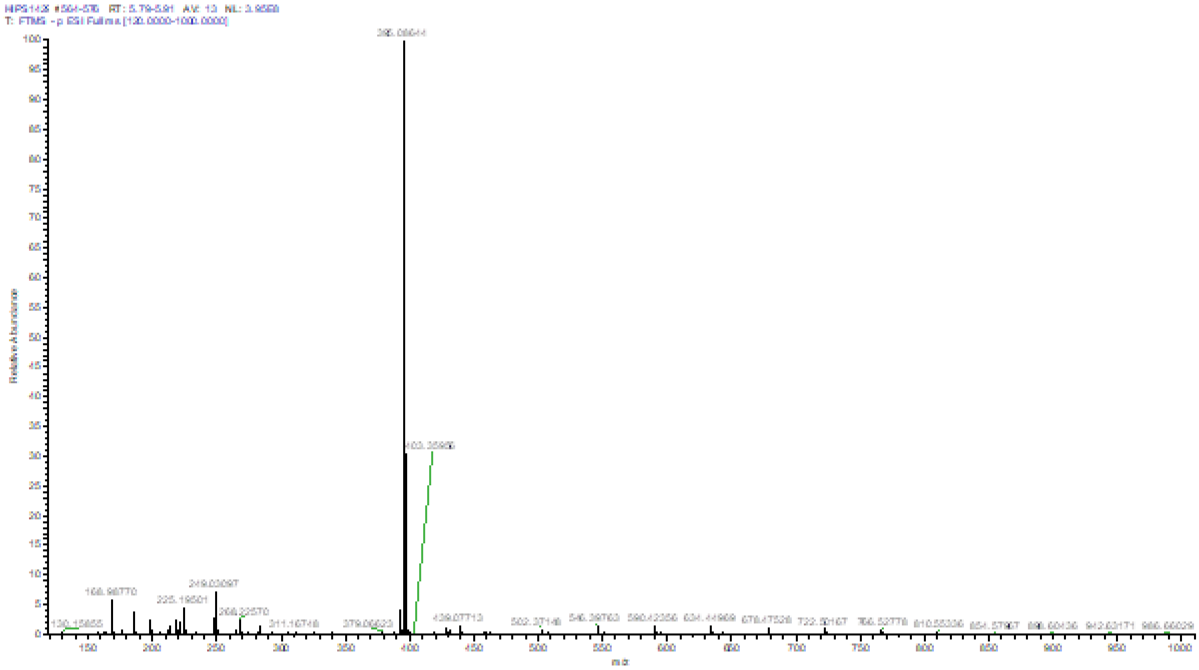

Compound **19**

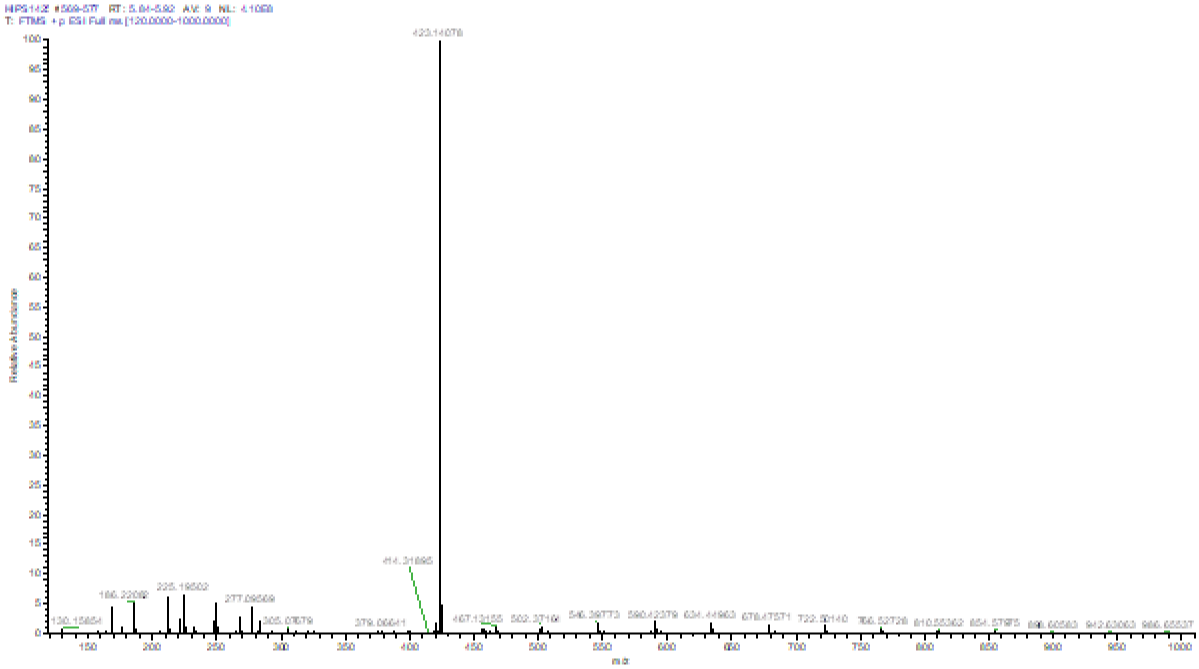

Compound **20**

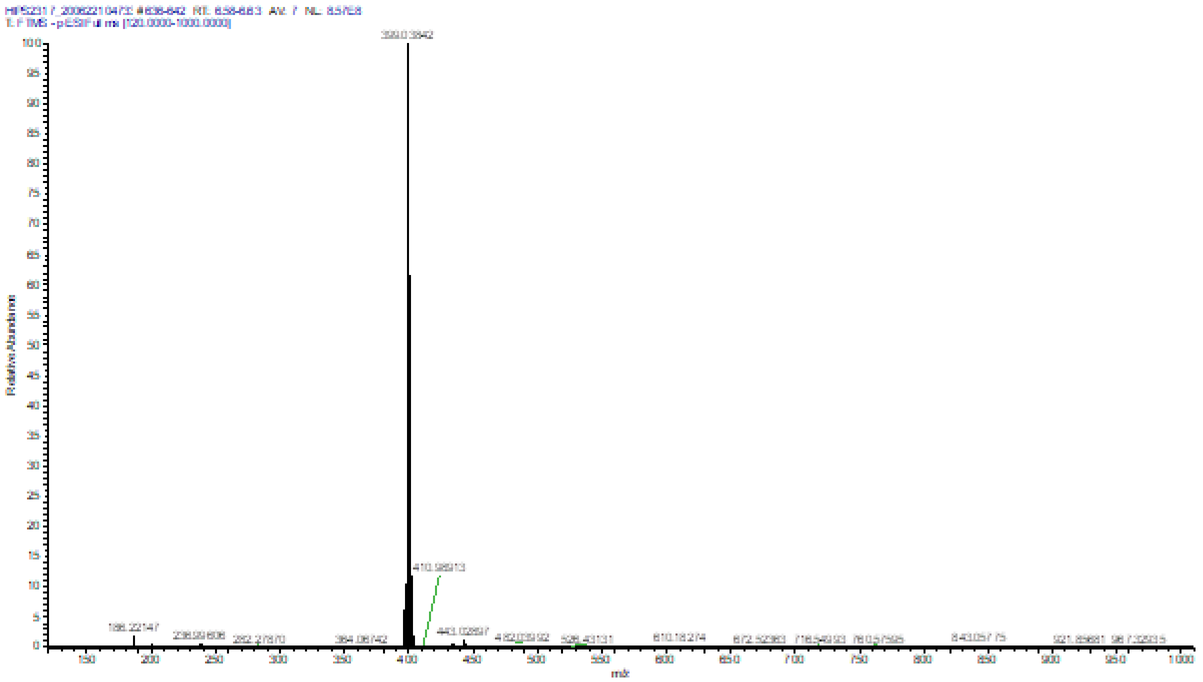

Compound **21**

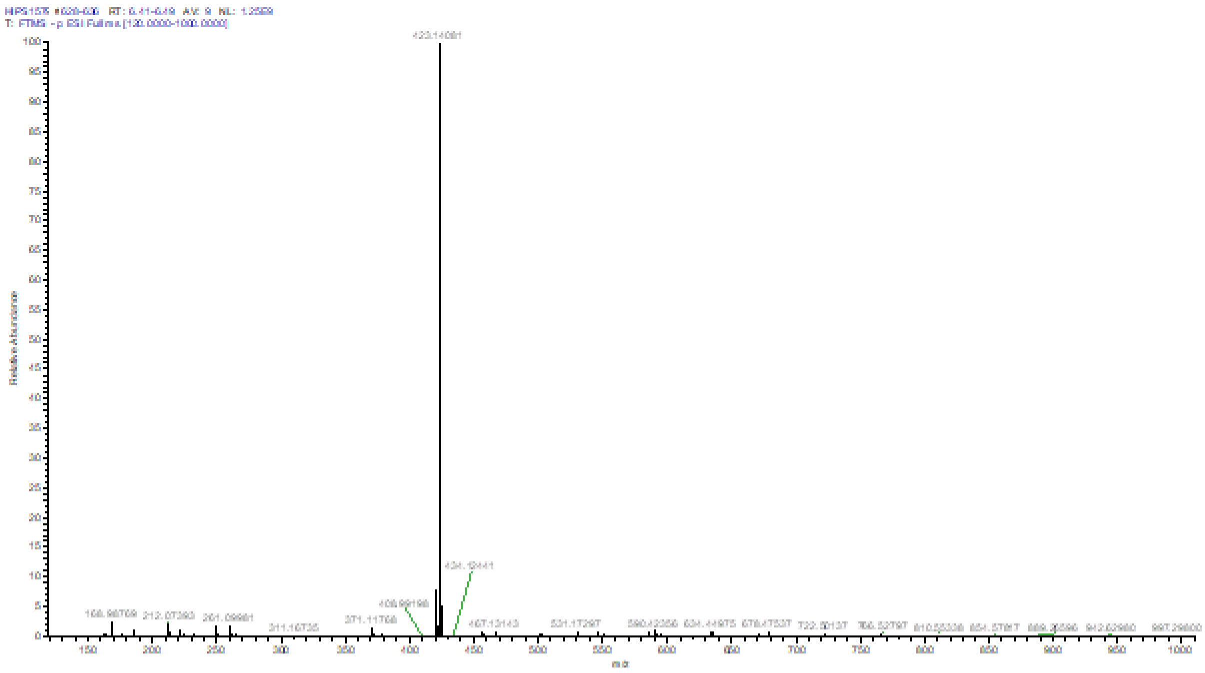

Compound **22**

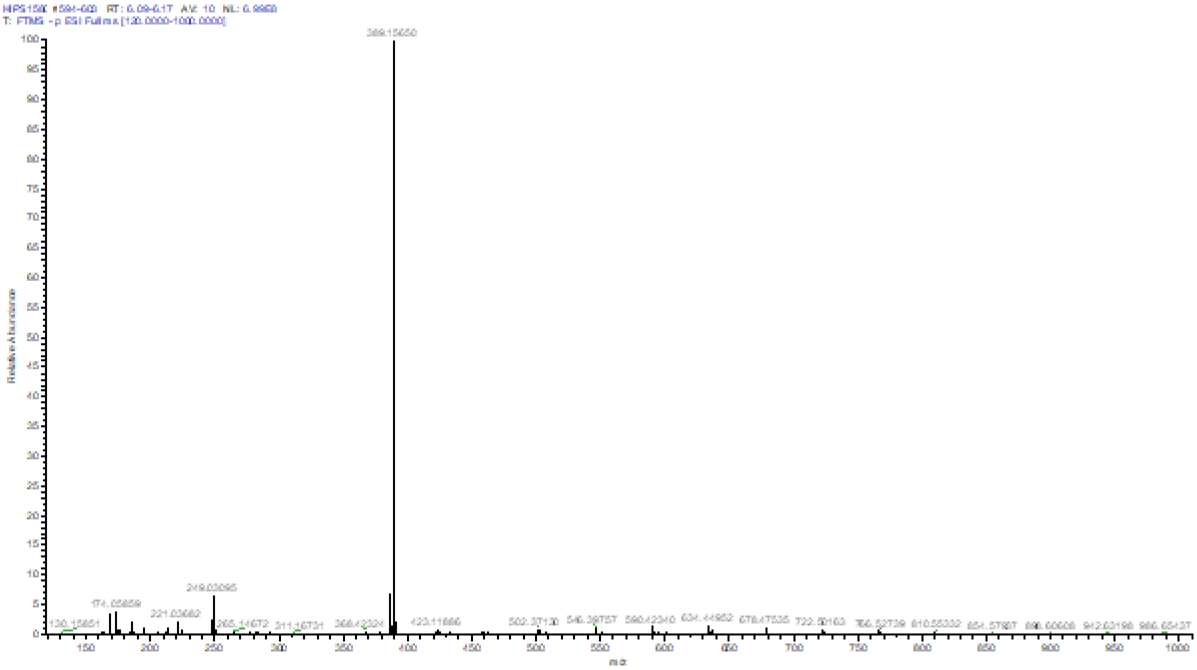

Compound **23**

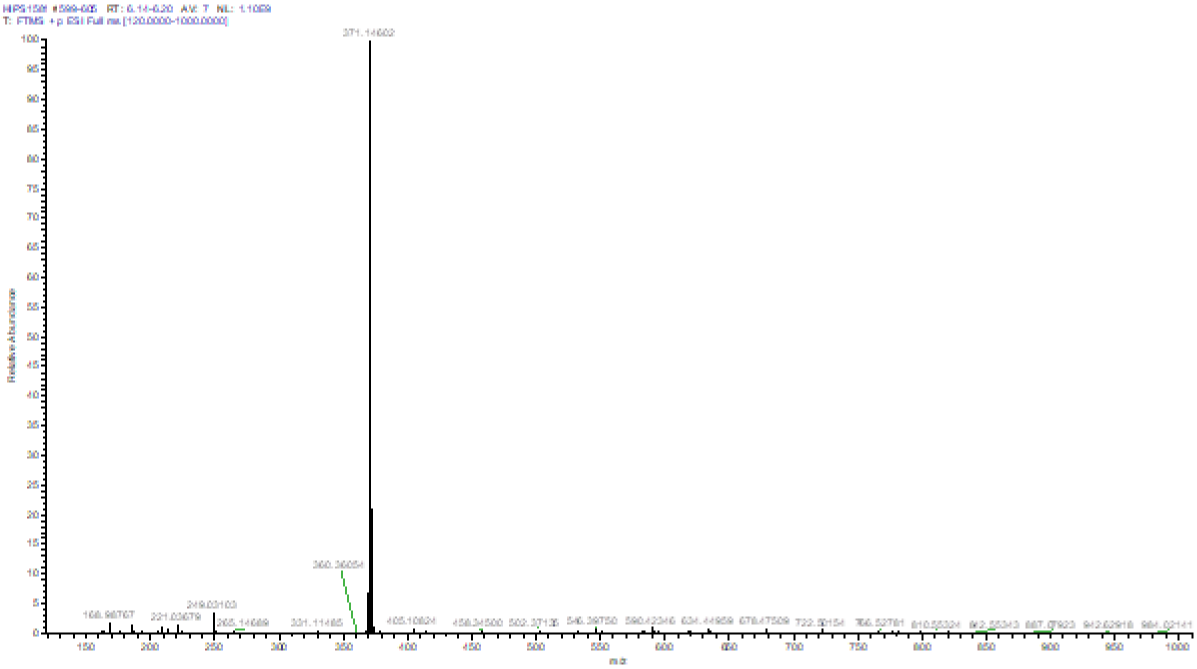

Compound **24**

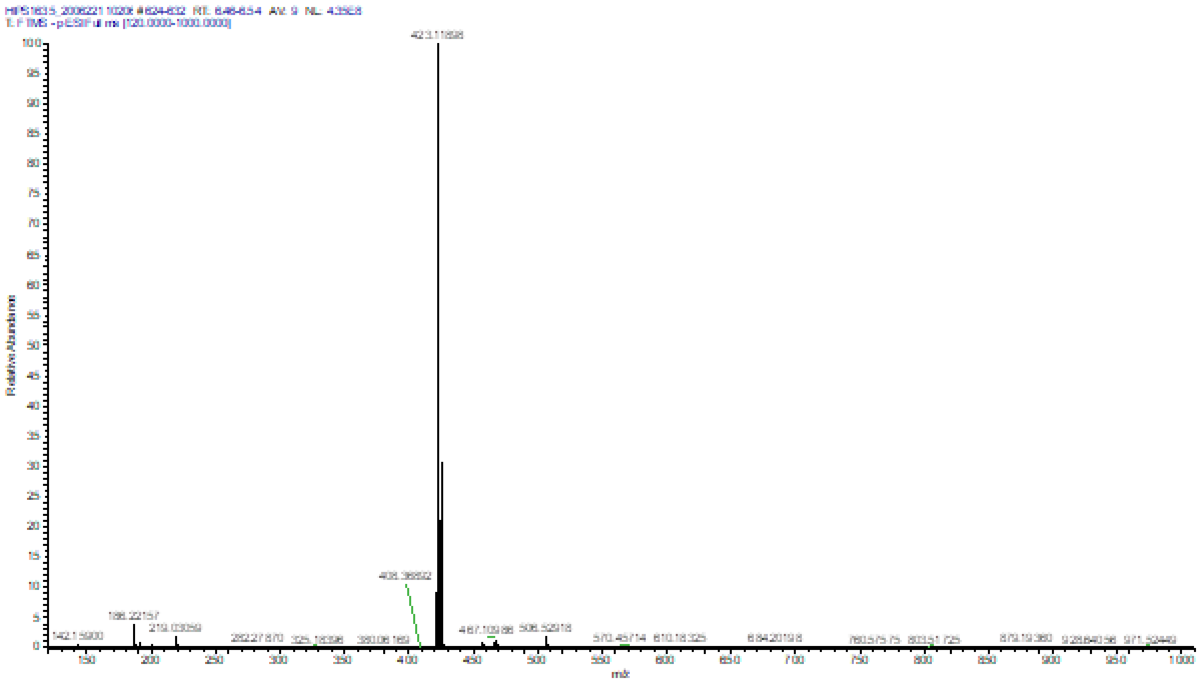

Compound **25**

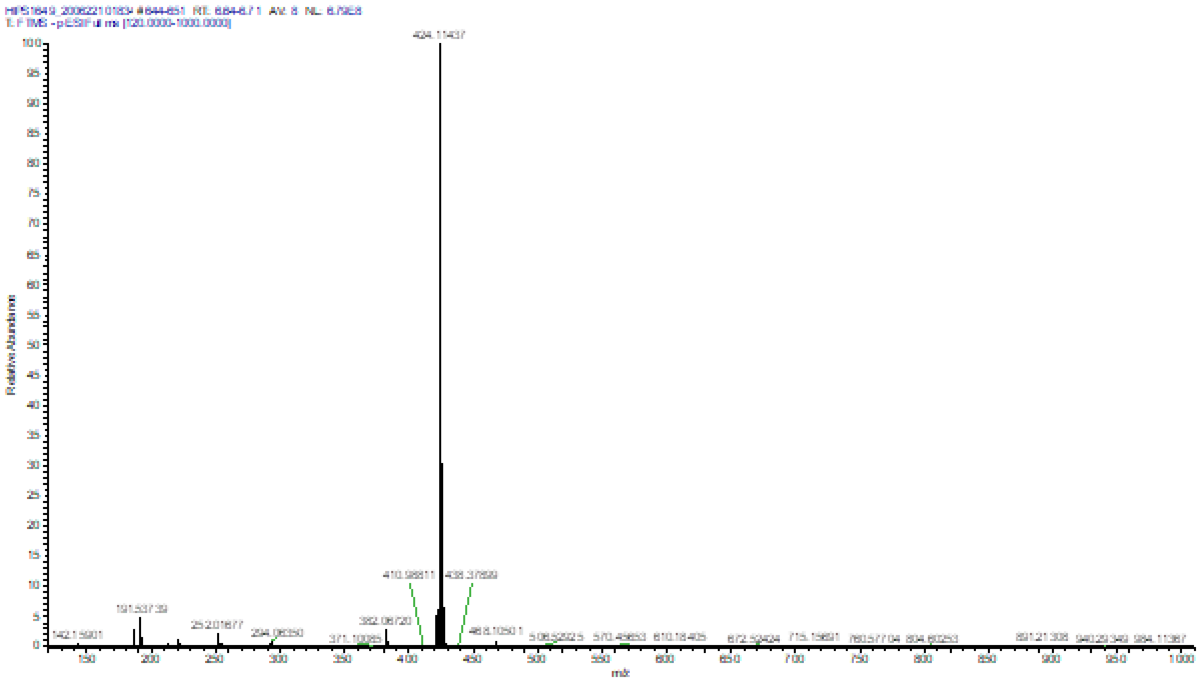

4 HPLC traces

Compound **2**

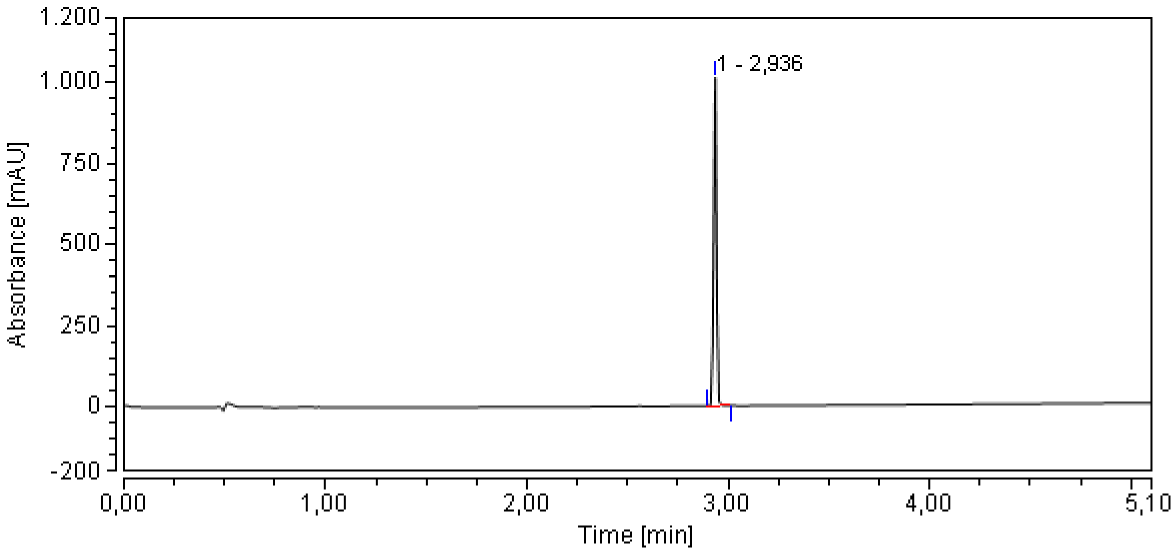

Compound **3**

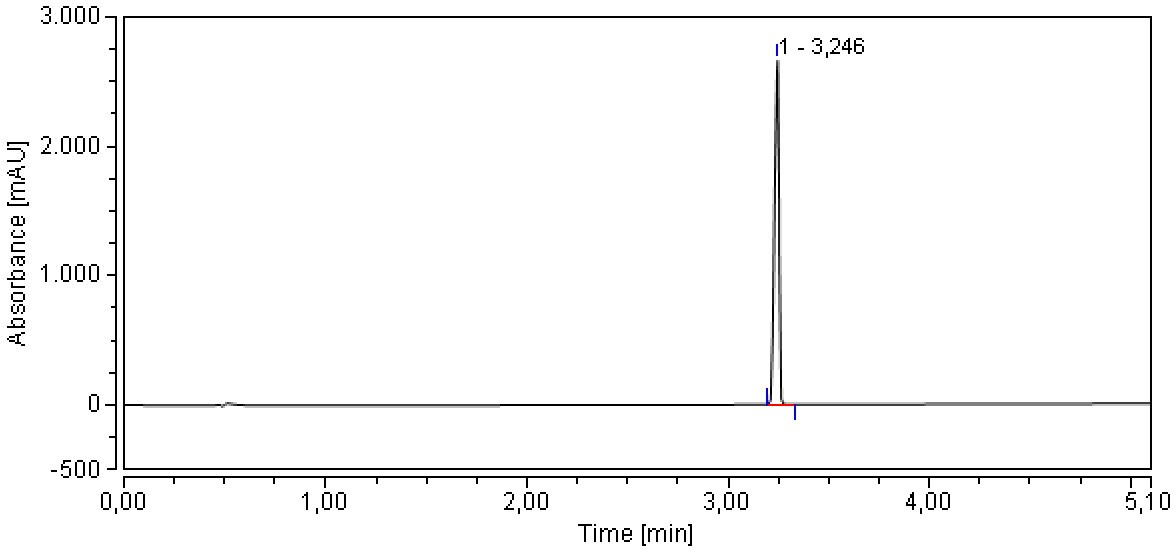

Compound **4**

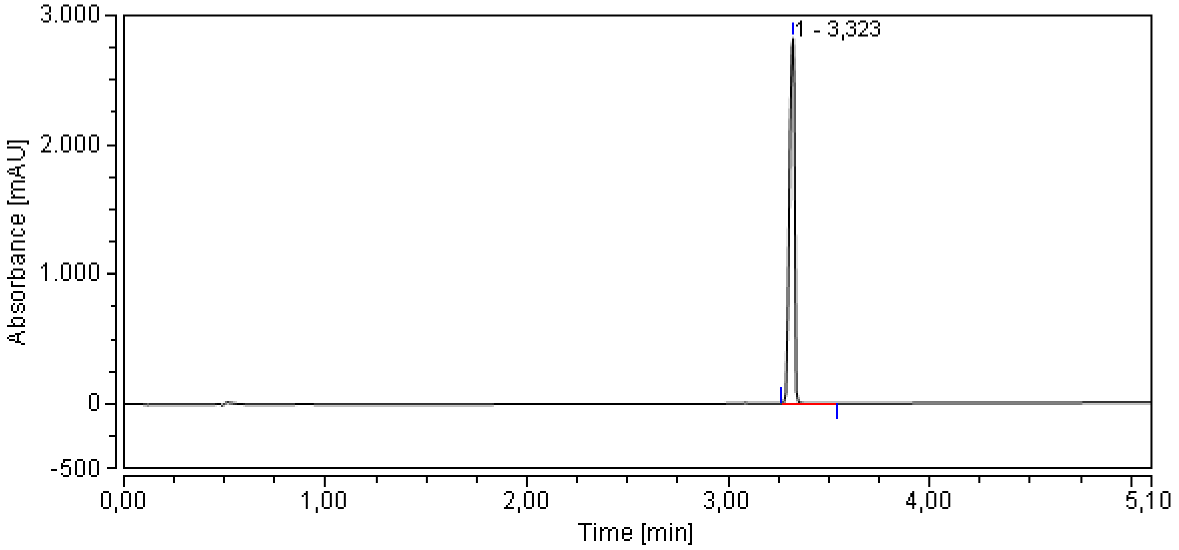

Compound **5**

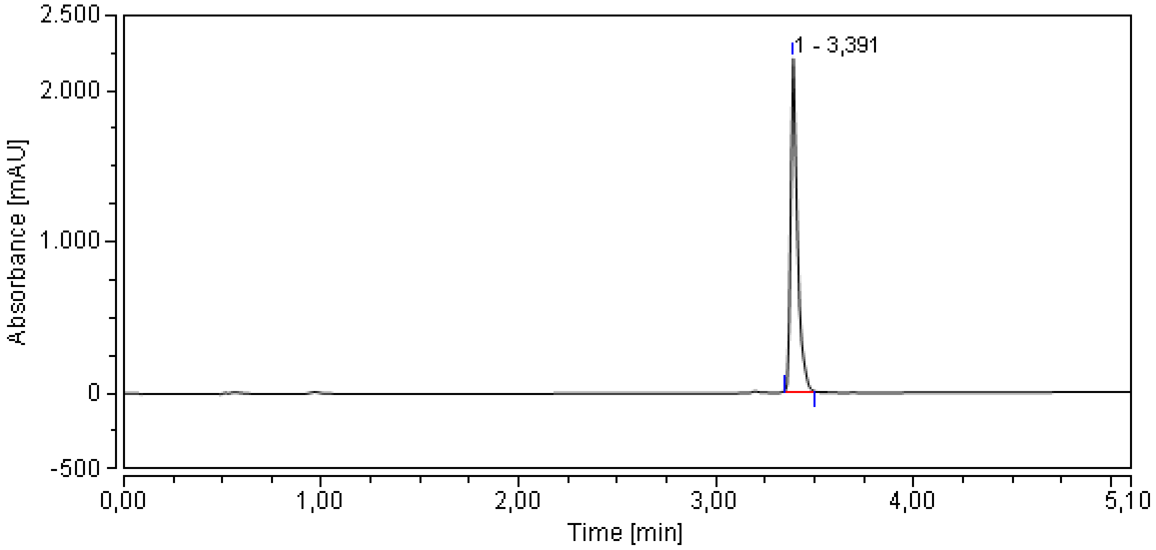

Compound **6**

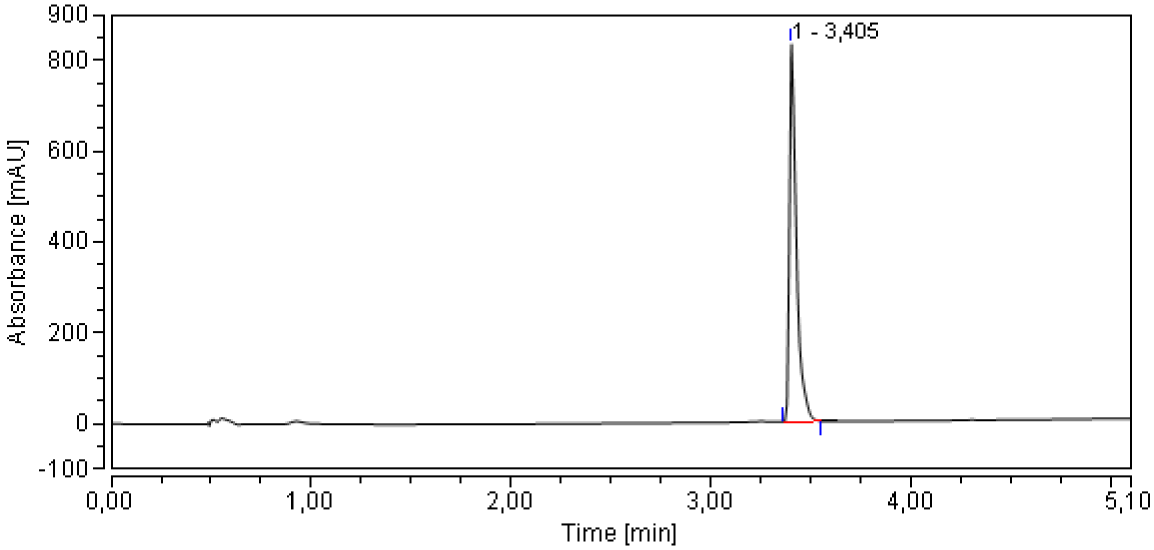

Compound **7**

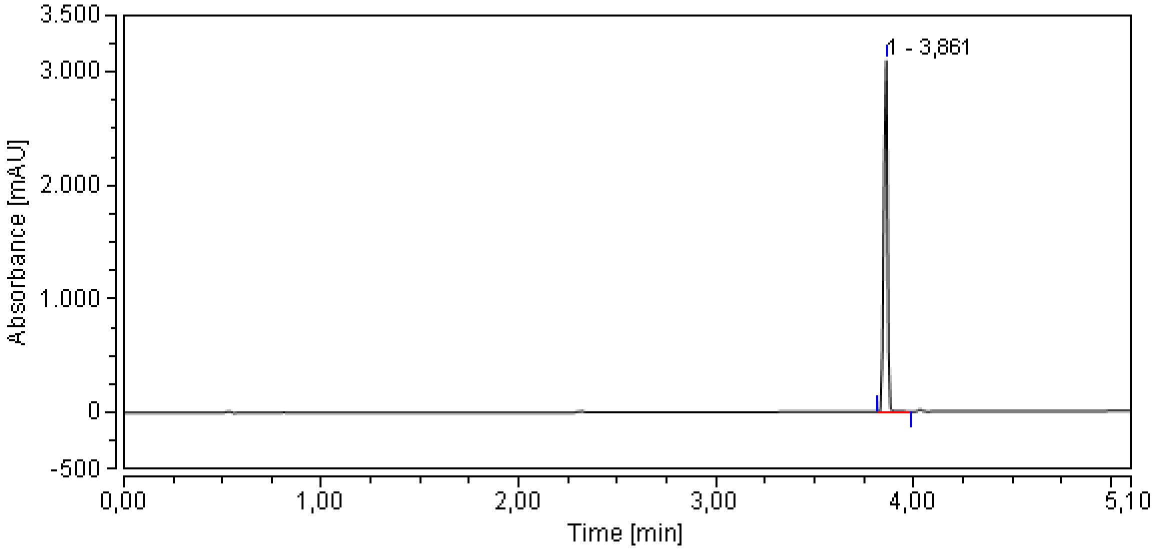

Compound **8**

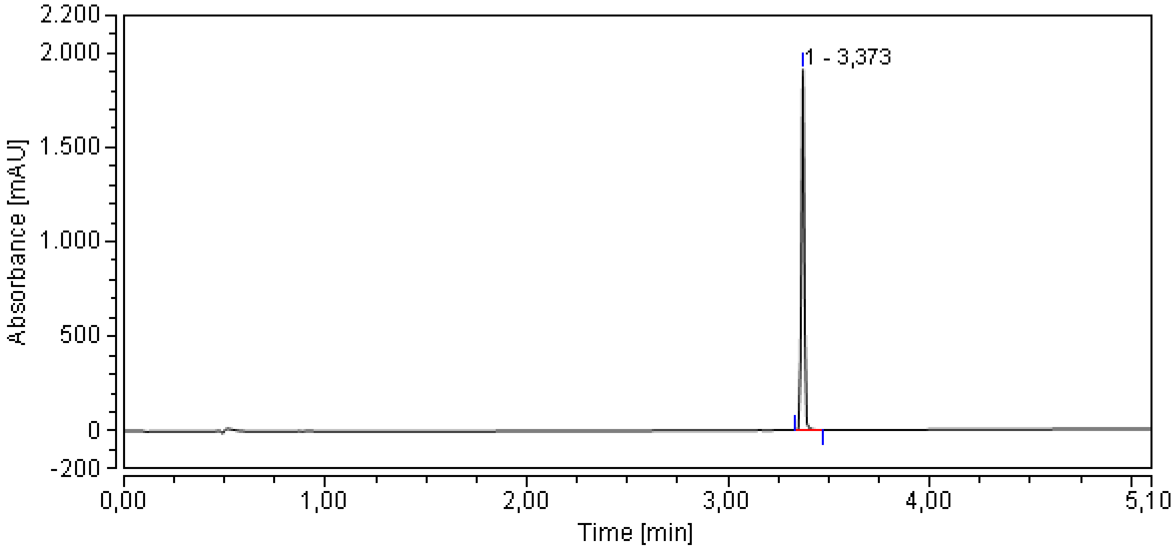

Compound **9**

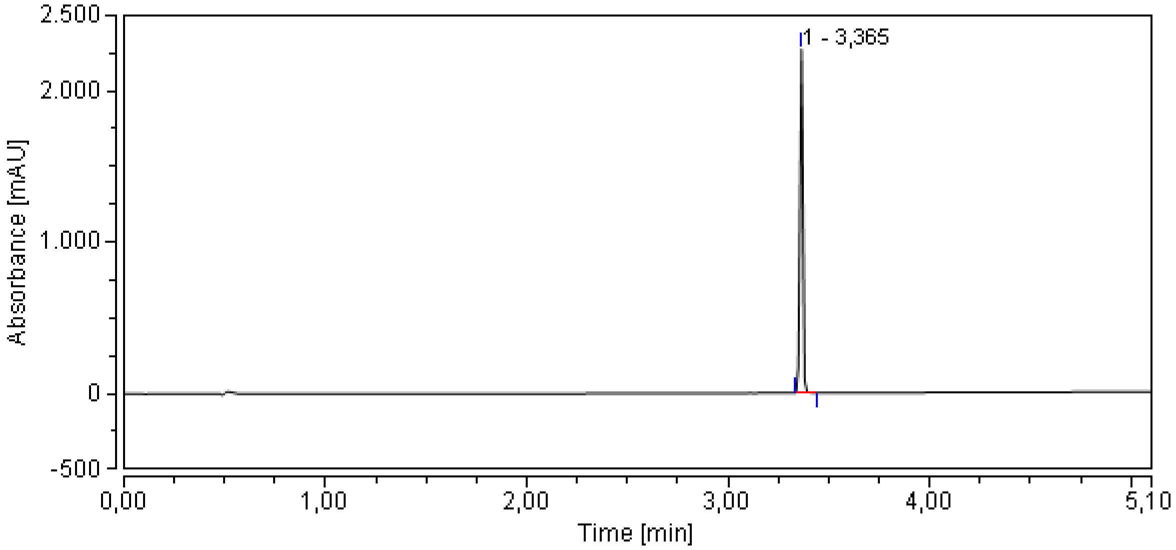

Compound **10**

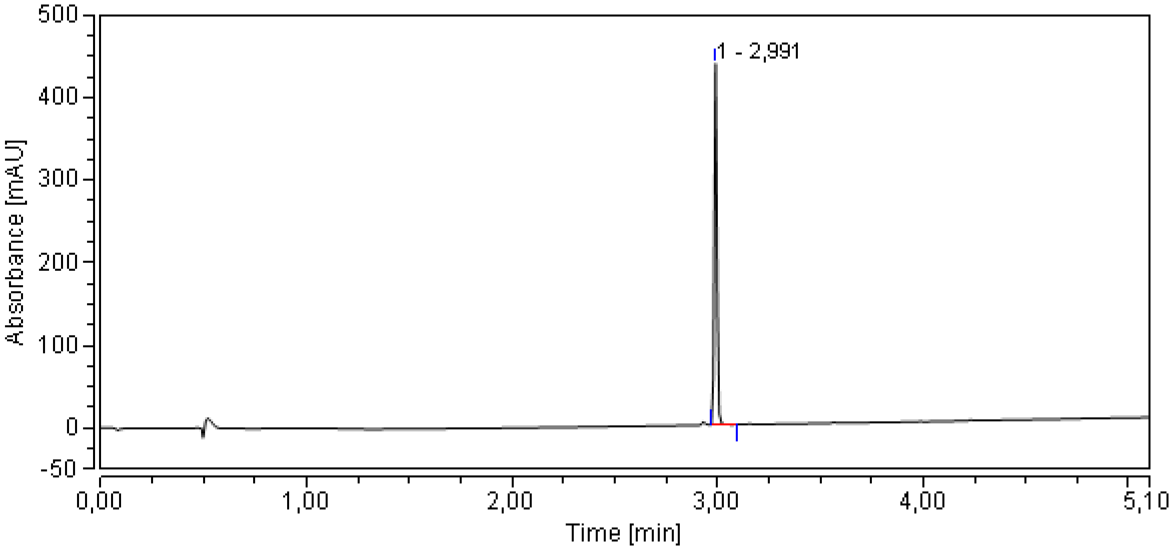

Compound **11**

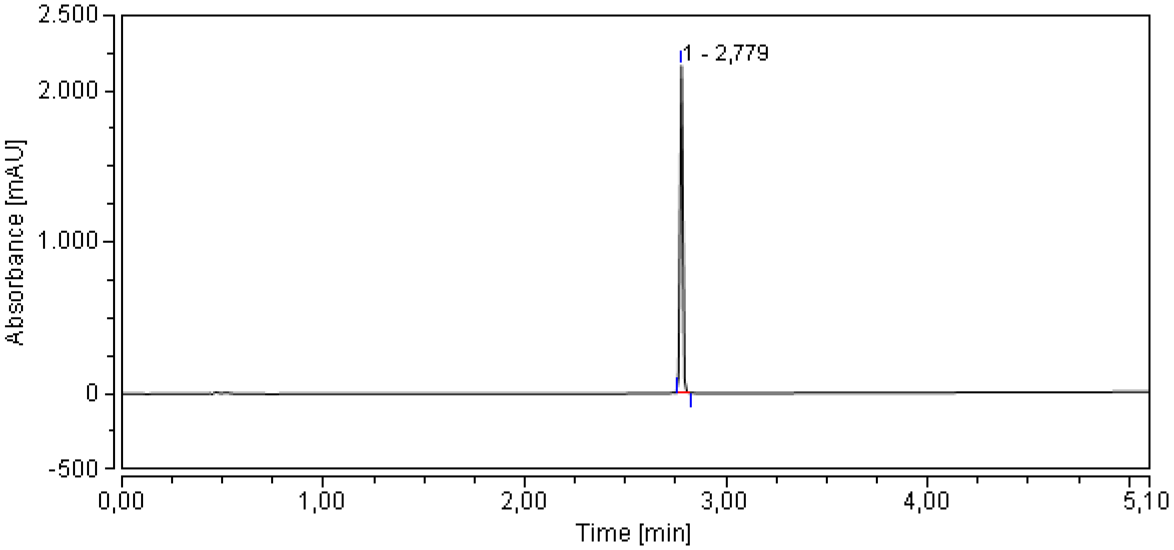

Compound **12**

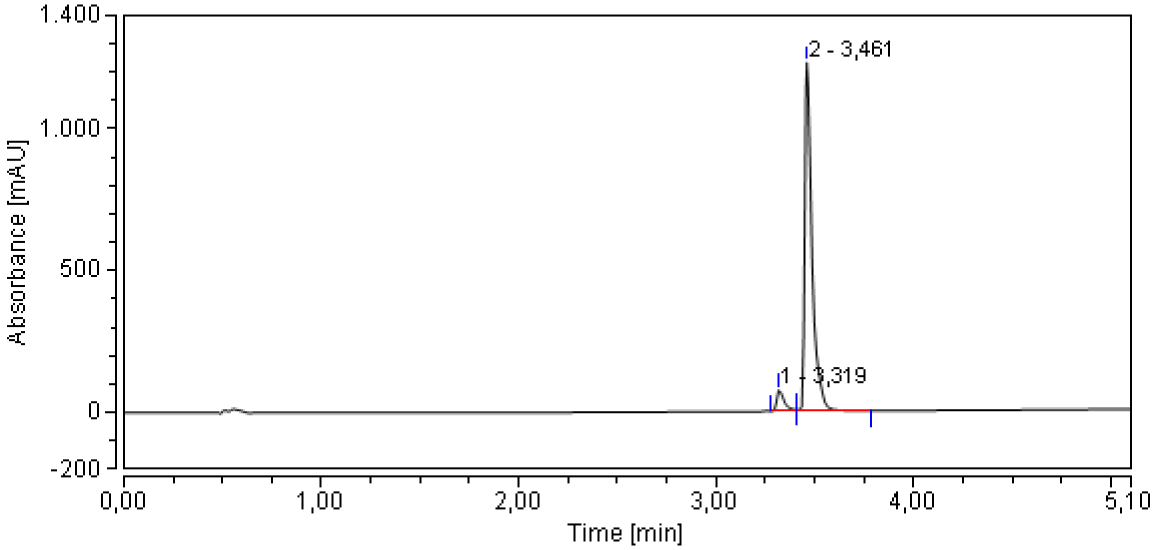

Compound **13**

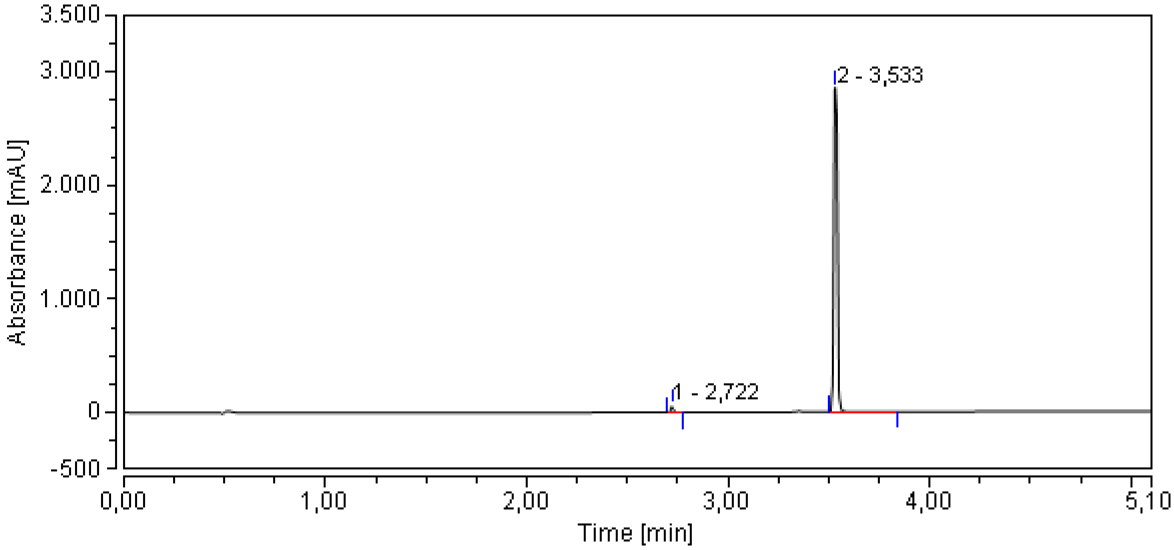

Compound **14**

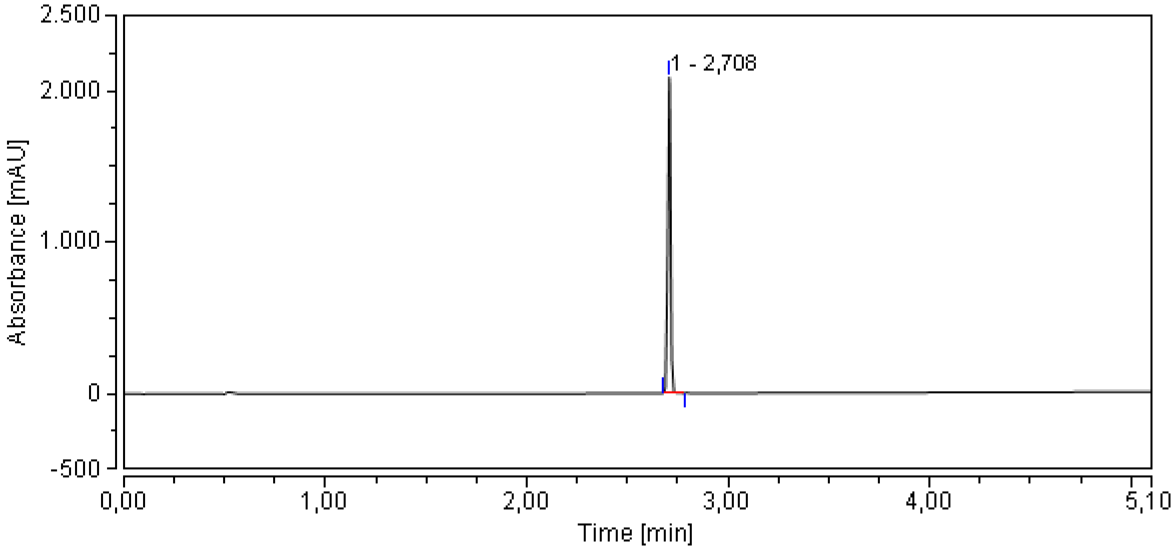

Compound **15**

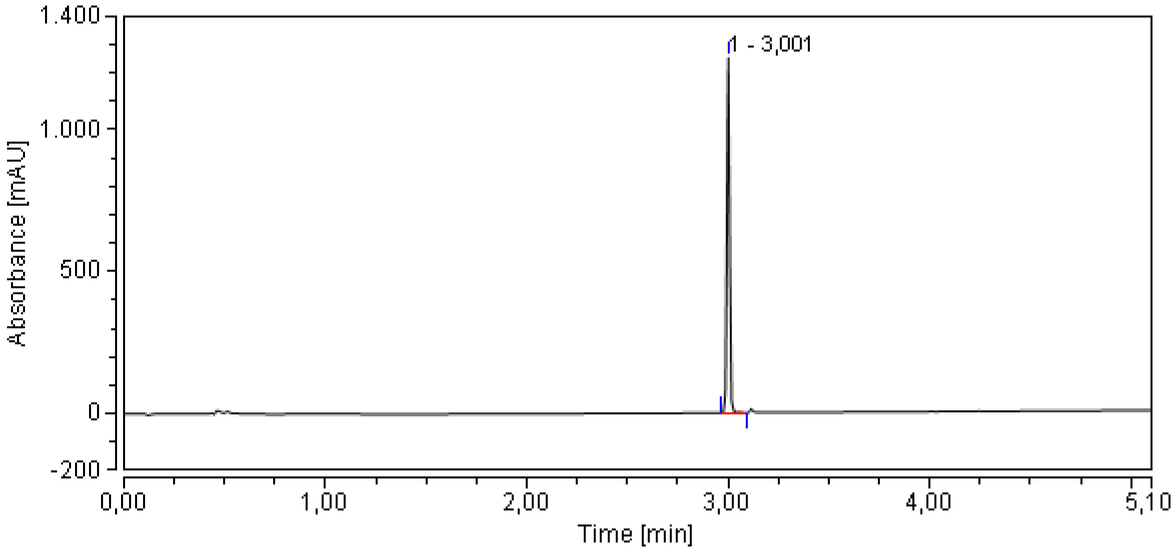

Compound **16**

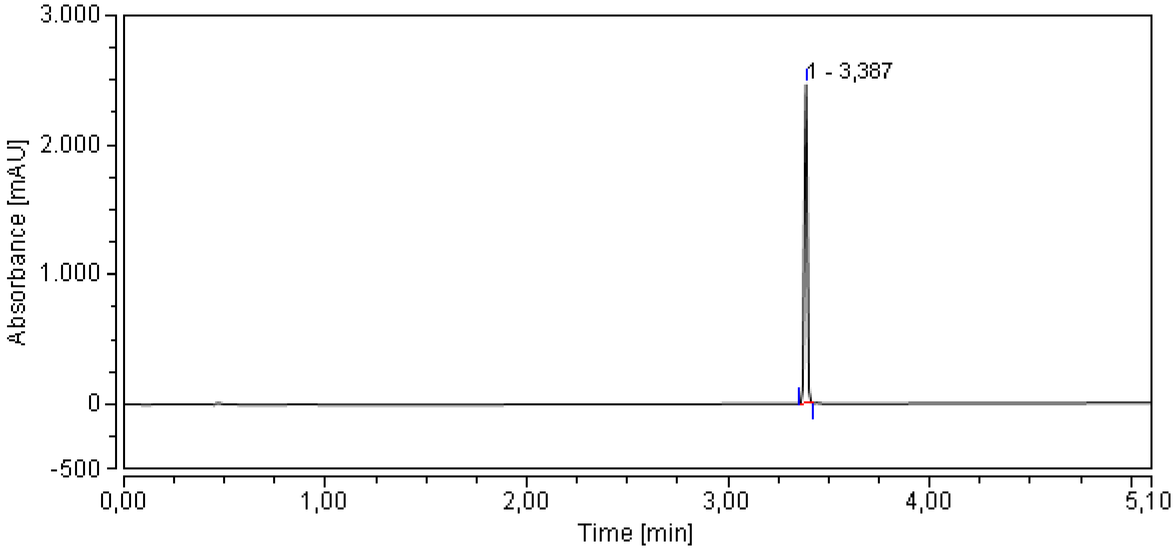

Compound **17**

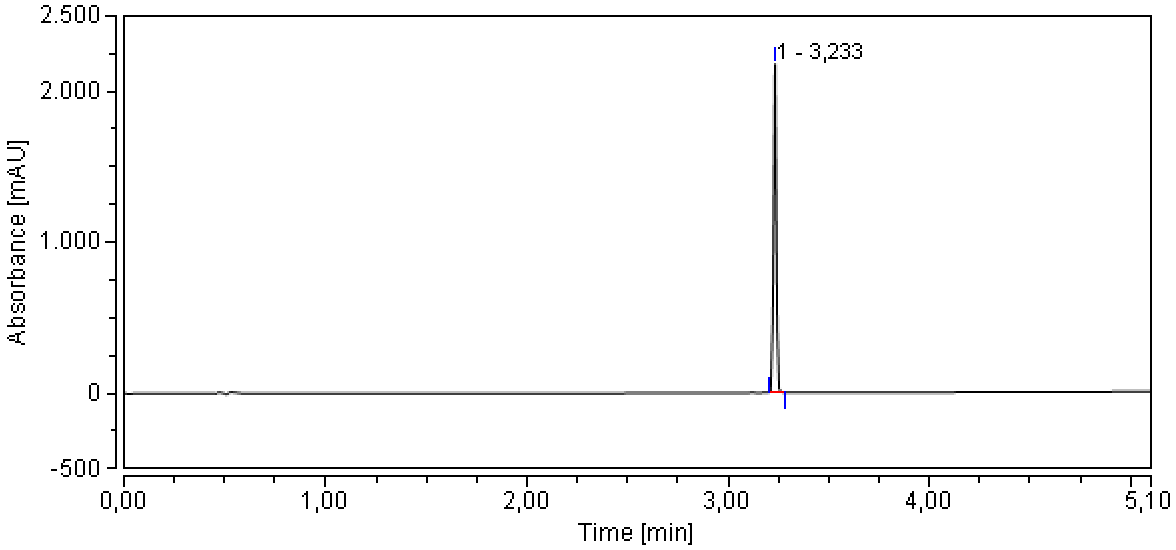

Compound **18**

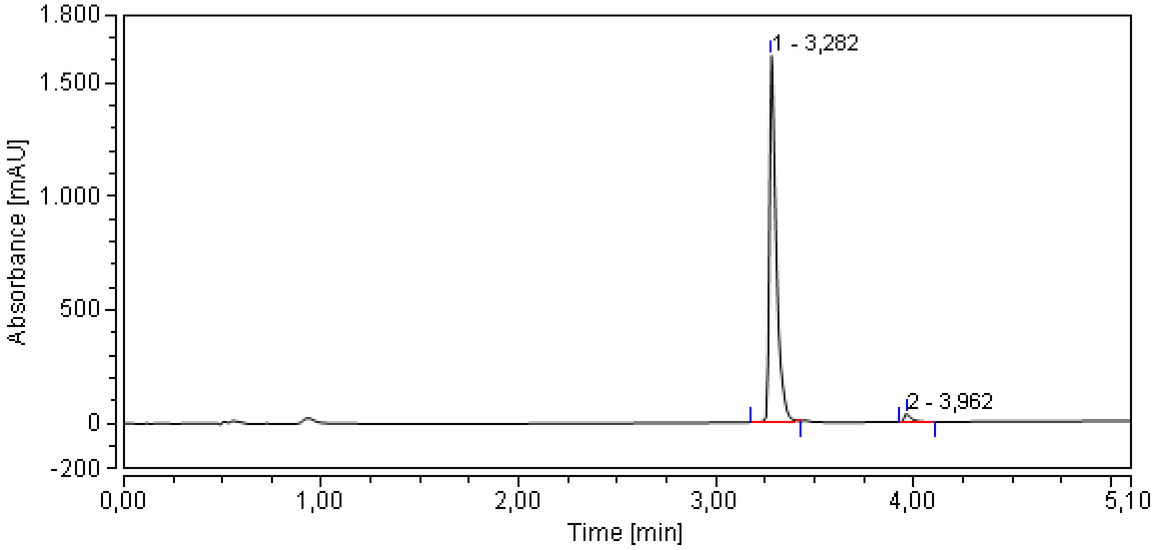

Compound **19**

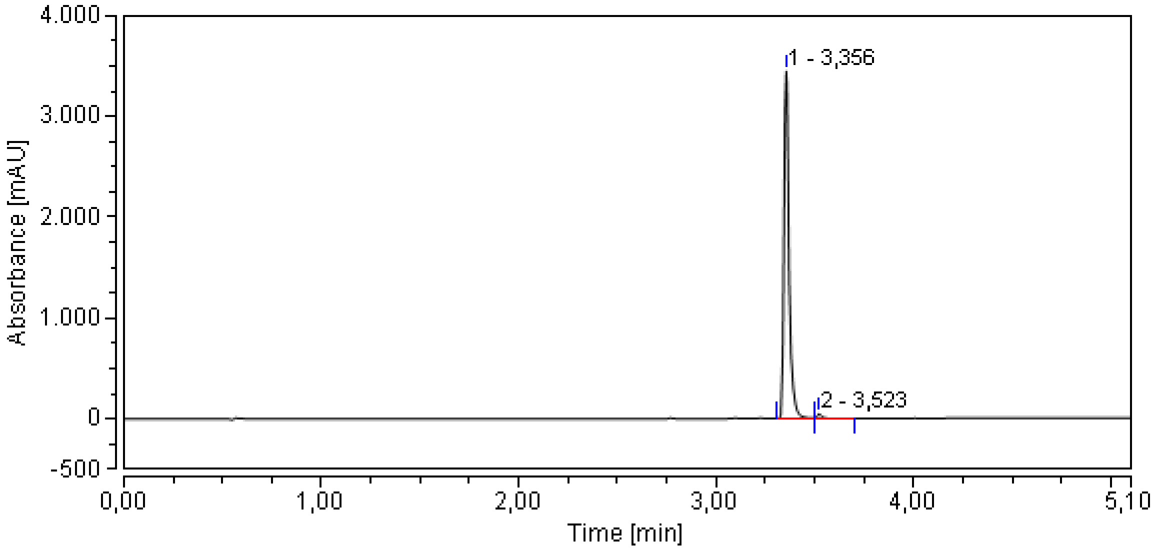

Compound **20**

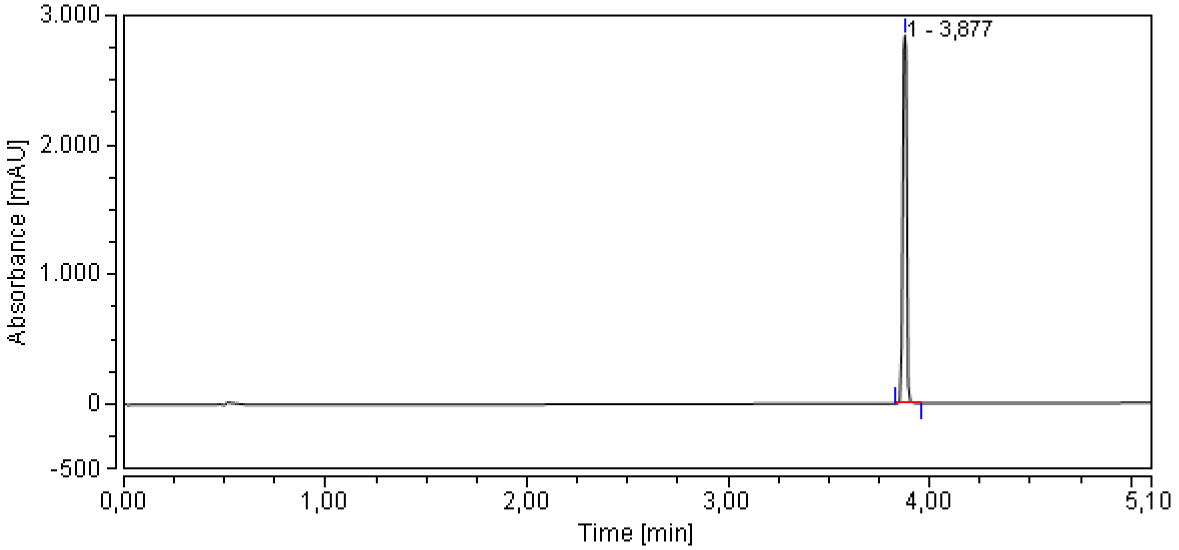

Compound **21**

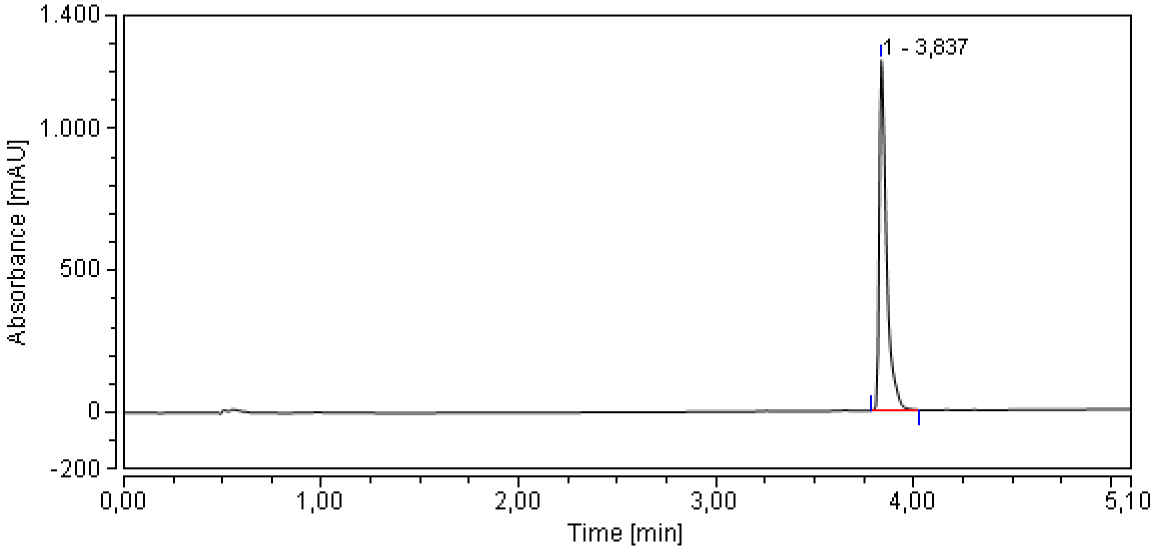

Compound **22**

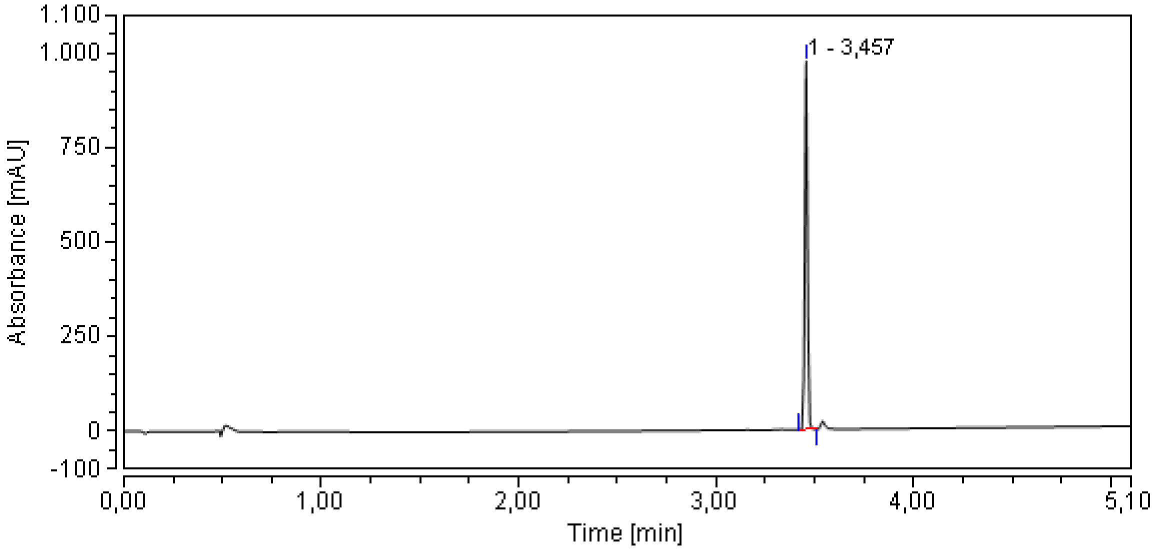

Compound **23**

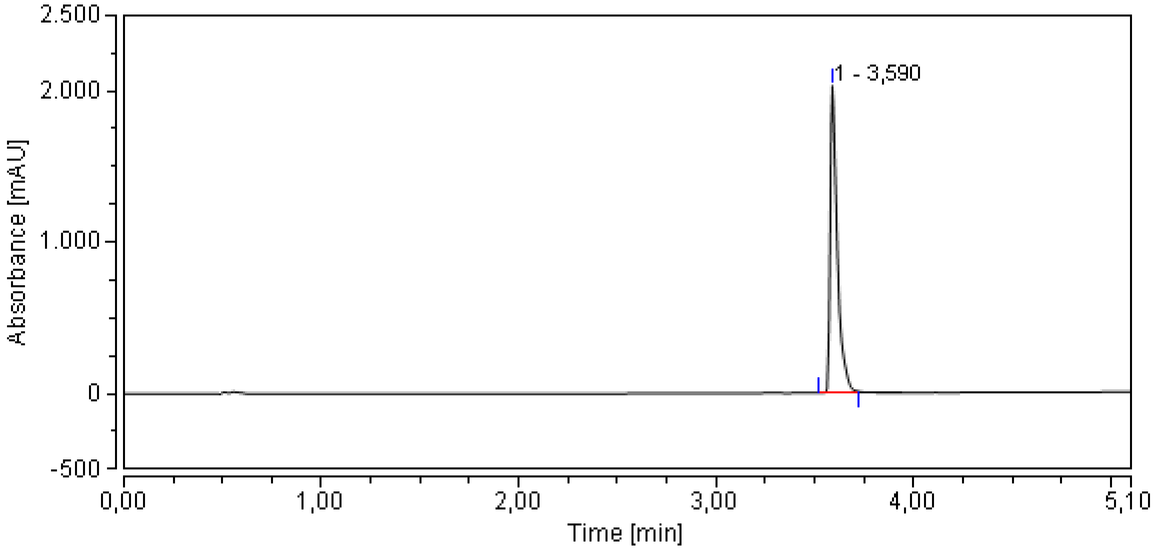

Compound **24**

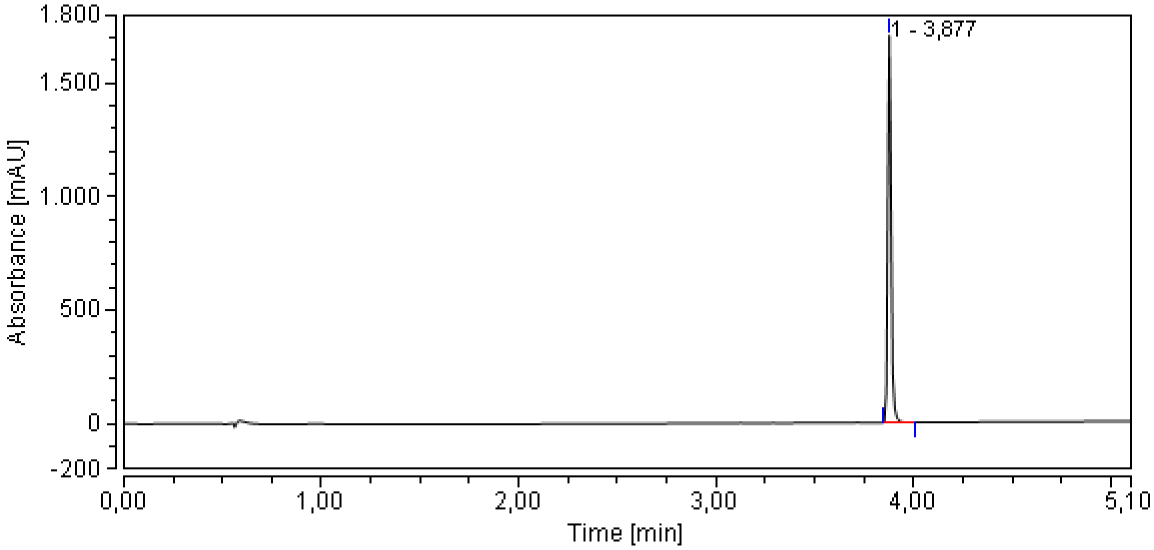

Compound **25**

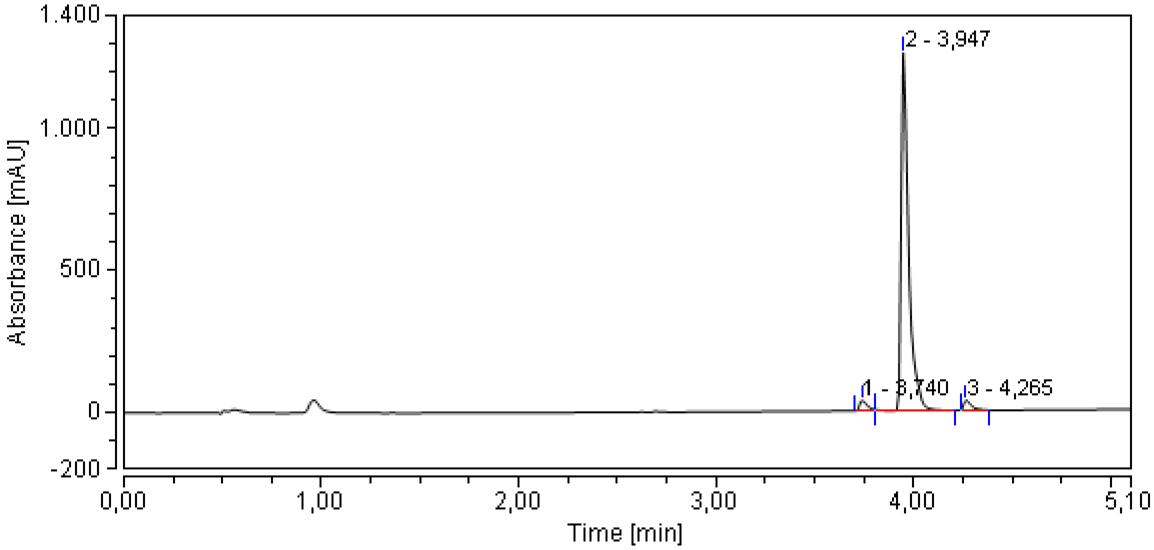

5 REFERENCES

[1] C. Lu, B. Kirsch, C. Zimmer, J. C. de Jong, C. Henn, C. K. Maurer, M. Müsken, S. Häussler, A. Steinbach, R. W. Hartmann, *Chemistry & biology* **2012**, *19*, 381.

[2] C. Lu, C. K. Maurer, B. Kirsch, A. Steinbach, R. W. Hartmann, *Angewandte Chemie (International ed. in English)* **2014**, *53*, 1109.

[3] A. Thomann, A. G. G. de Mello Martins, C. Brengel, M. Empting, R. W. Hartmann, *ACS chemical biology* **2016**, *11*, 1279.

[4] L. Wiehlmann, G. Wagner, N. Cramer, B. Siebert, P. Gudowius, G. Morales, T. Köhler, C. van Delden, C. Weinel, P. Slickers et al., *Proceedings of the National Academy of Sciences of the United States of America* **2007**, *104*, 8101.

[5] B. Curran, D. Jonas, H. Grundmann, T. Pitt, C. G. Dowson, *Journal of clinical microbiology* **2004**, *42*, 5644.

[6] R. Hilker, A. Munder, J. Klockgether, P. M. Losada, P. Chouvarine, N. Cramer, C. F. Davenport, S. Dethlefsen, S. Fischer, H. Peng et al., *Environmental microbiology* **2015**, *17*, 29.

[7] G. L. Winsor, E. J. Griffiths, R. Lo, B. K. Dhillon, J. A. Shay, F. S. L. Brinkman, *Nucleic acids research* **2016**, *44*, D646-53.

[8] B. M. Coffey, G. G. Anderson, *Methods in molecular biology (Clifton, N.J.)* **2014**, *1149*, 631.

[9] J. Schindelin, I. Arganda-Carreras, E. Frise, V. Kaynig, M. Longair, T. Pietzsch, S. Preibisch, C. Rueden, S. Saalfeld, B. Schmid et al., *Nature methods* **2012**, *9*, 676.

[10] M. A. Abril, C. Michan, K. N. Timmis, J. L. Ramos, *Journal of bacteriology* **1989**, *171*, 6782.

[11] Y. Zhang, M. Huo, J. Zhou, S. Xie, *Computer methods and programs in biomedicine* **2010**, *99*, 306.

[12] C. Vonrhein, C. Flensburg, P. Keller, A. Sharff, O. Smart, W. Paciorek, T. Womack, G. Bricogne, *Acta crystallographica. Section D, Biological crystallography* **2011**, *67*, 293.

[13] Tickle, I.J., Flensburg, C., Keller, P., Paciorek, W., Sharff, A., Vonrhein, C., Bricogne, G., *STARANISO. Cambridge, United Ltd., Kingdom: Global Phasing 2018*, **2018**.

[14] A. J. McCoy, R. W. Grosse-Kunstleve, P. D. Adams, M. D. Winn, L. C. Storoni, R. J. Read, *Journal of applied crystallography* **2007**, *40*, 658.

[15] P. V. Afonine, R. W. Grosse-Kunstleve, N. Echols, J. J. Headd, N. W. Moriarty, M. Mustyakimov, T. C. Terwilliger, A. Urzhumtsev, P. H. Zwart, P. D. Adams, *Acta crystallographica. Section D, Biological crystallography* **2012**, *68*, 352.

[16] P. D. Adams, P. V. Afonine, G. Bunkóczi, V. B. Chen, I. W. Davis, N. Echols, J. J. Headd, L.-W. Hung, G. J. Kapral, R. W. Grosse-Kunstleve et al., *Acta crystallographica. Section D, Biological crystallography* **2010**, *66*, 213.

[17] Chemical Computing Group ULC 2018., *Molecular Operating Environment (MOE),. Chemical Computing Group ULC 2018.*, Chemical Computing Group ULC 2018.

[18] E. Krieger, G. Koraimann, G. Vriend, *Proteins* **2002**, *47*, 393.
